# Benzothiazole heterogeneous photodegradation in nano α-Fe_2_O_3_/oxalate system under UV light irradiation

**DOI:** 10.1098/rsos.180322

**Published:** 2018-06-27

**Authors:** Xiangyun Han, Xi Zhang, Lei Zhang, Mei Pan, Jinlong Yan

**Affiliations:** 1 School of Environmental Science and Engineering, Yancheng Institute of Technology, Yancheng 224003, China; 2 College of Life and Environmental Sciences, Shanghai Normal University, Shanghai 201418, China

**Keywords:** benzothiazole, photodegradation, transformation products, degradation pathway

## Abstract

The photodegradation of benzothiazole (BTH) in wastewater with the coexistence of iron oxides and oxalic acid under UV light irradiation was investigated. Results revealed that an effective heterogeneous photo-Fenton-like system could be set up for BTH abatement in wastewater under UV irradiation without additional H_2_O_2_, and 88.1% BTH was removed with the addition of 2.0 mmol l^−1^ oxalic acid and 0.2 g l^−1^ α-Fe_2_O_3_ using a 500 W high-pressure mercury lamp (365 nm). The degradation of BTH in the photo-Fenton-like system followed the first-order kinetic model. The photoproduction of hydroxyl radicals (·OH) in different systems was determined by high-performance liquid chromatography. Identification of transformation products by using liquid chromatography coupled with high resolution tandem mass spectrometry provided information about six transformation products formed during the photodegradation of BTH. Further insight was obtained by monitoring concentrations of the sulfate ion $\left({{\mathrm{SO}}_{4}}^{2-}\right)$ and nitrate ion $\left({{\mathrm{NO}}_{3}}^{-}\right)$, which demonstrated that the intermediate products of BTH could be decomposed ultimately. Based on the results, the potential photodegradation pathway of BTH was also proposed.

## 1. Introduction

Benzothiazoles (BTHs), a group of xenobiotic compounds consisting of a five-membered 1,3-thiazole ring attached to a benzene ring by a common C─C bond, are used in a variety of industry products and processes. For example, BTHs are used as slimicides in the paper and pulp industry [1], as fungicides in lumber and leather production [2], as vulcanization accelerators in the manufacture of rubber products and tyres [3], and as stabilizers in the photo industry [4].

Owing to the widespread use and poor elimination of BTHs by conventional wastewater treatment processes [5], sewage is considered as their main pathway to the aquatic environment [6]. An additional source of BTHs in water includes street runoff containing abrasion residues of tyres [7]. An average concentration of BTHs in an effluent from a Greek wastewater treatment plant was 254 ng l^−1^ [6], and a survey done in China revealed that the occurrence of BTHs in river water was in the range of 158–473 ng l^−1^ [8].

It was found that most of BTHs not only inhibited the activity of microorganisms [9] but also showed toxic effects to mammals. The advanced oxidation processes (AOP), such as H_2_O_2_/UV, photo-Fenton and ozone, have been used to oxidize benzothiazole compounds [10–14], suggesting that AOP can be efficient for elimination of BTHs.

Oxalic acid is ubiquitous in water and soil [15]. Iron is the fourth most abundant element of the Earth's crust (5.1 mass%). Major iron oxides in the natural environment include goethite (α-FeOOH), hematite (α-Fe_2_O_3_), maghemite (γ-Fe_2_O_3_), lepidocrocite (γ-FeOOH) and magnetite (Fe_3_O_4_). In recent years, development of heterogeneous photo-Fenton process has caused increasing research interest. And solid iron hydroxides/oxides such as hydroxyl-Fe [16], hematite [17], maghemite [18], goethite [19], magnetite [20,21], Fe_3_O_4_@γ-Fe_2_O_3_ [22], and Fe_3_O_4_/multiwall carbon nanotubes/polyhydroquinone [23] have been used as catalysts in heterogeneous photo-Fenton process. A so-called photo-Fenton-like system under light irradiation can be set up when iron oxides and oxalic acid coexist [15,24,25]. It has been reported that 2-mercaptobenzothiazole can be oxidized by photo-Fenton-like techniques, and degradation efficiency could be greatly accelerated with the co-presence of iron oxides and oxalate [26].

Here, we aim to investigate the photodegradation behaviour of BTH and define the best conditions to improve the BTH degradation in a heterogeneous system composed of iron oxides and oxalic acid. Meanwhile, based on the data obtained from high-performance liquid chromatography coupled with quadrupole time-of-flight mass spectrometry (HPLC-QTOF-MS) analysis and the calculation of the frontier electron density of BTH, the initial steps of degradation of BTH and its resulting transformation products were proposed.

## 2. Material and methods

### 2.1. Reagents

BTH (technical grade, 96%) and oxalic acid (AR, 98%) were purchased from Shanghai Aladdin Biochemical Technology Co., Ltd, China. α-Fe_2_O_3_ (99.5%, 30 nm) was obtained from Shanghai Ziyi Reagent Co., Ltd, China. Other analytical-grade chemicals were purchased from Sinopharm Chemical Reagent Co., Ltd, China. Methyl alcohol (HPLC grade) was used for HPLC analysis. Chromatographic-grade methyl alcohol was purchased from Tedia Company, USA. All chemicals were used without further purification and all solutions were prepared using double-distilled water.

### 2.2. Experiments of benzothiazole photodegradation

The photodegradation experiments of BTH were carried out in an XPA-7 photochemical reactor (Xujiang Electromechanical Plant, Nanjing, China). Throughout the experiments, the experimental solution temperature was maintained at 20 ± 1°C by cooling water circulation. The irradiation source was a 500 W high-pressure mercury lamp with a maximum light intensity output at 365 nm. The lamp was placed into a hollow quartz trap located at the centre of the reactor. The light intensity at quartz tube positions was measured to be 8.96 × 10^2^ mW cm^−2^ by a UV irradiation meter (UV-A, Beijing Normal University, China), and illumination to be 7.9 × 10^4^ lx by a lux meter (AS-813, Smart Sensor, China). Before irradiation, the suspension was sealed and agitated for 30 min to reach adsorption equilibrium. The initial pH of reaction solutions was regulated with sulfuric acid solution (with hydrochloric acid when acid ions were measured) and sodium hydroxide solution, and the final solution volume was adjusted to 50 ml with double-distilled water. Then, the solution was placed into the photochemical reactor and stirred with magnetic stirrers. At fixed time points, analytical samples were withdrawn from the suspension with a pipette, immediately centrifuged at 10 000 r.p.m. and then filtered by using a syringe equipped with a 0.45 µm membrane filter for further analysis.

### 2.3. Analysis methods

The concentrations of BTH during the experiments were quantified by a PerkinElmer HPLC equipped with a SPHERI-5RP-18 column (4.6 × 150 mm, 5 µm) at a wavelength of 254 nm, and the retention time of BTH was 6.4 min. The mobile phase was methanol–water (90 : 10, v/v), and the flow rate was set as 0.6 ml min^−1^.

Identification of transformation products (TPs) in the solution was performed by employing a Waters Acquity G2 Q-TOF LC-MS instrument, which was composed of a Waters Acquity ultra-performance liquid chromatography (UPLC) system coupled to a QTOF mass spectrometer. Analytes were eluted with a gradient programme using MeOH (A) and water (B), both acidified with 0.1% formic acid. And the gradient programme was: held at 15% A for 0–2 min; 2.0–16.0 min, linear increase 15–95% A; 16.0–21.0 min, held at 95% A; 21.0–21.1 min, immediately reduced to 15% A to equilibrate the column [11]. All samples were kept refrigerated at 10°C in the UPLC auto sampler, and a 1.0 µl injection volume was used with a total flow rate of 0.2 ml min^−1^ over a total run time of 12 min. Mass spectrometry was performed on a Waters Synapt G2S Q-TOF (Micro mass MS Technologies, Manchester, UK) equipped with an electrospray ionization source operating both in positive and negative modes. The high-purity nitrogen as the nebulization gas was set at 800 l h^−1^ at a temperature of 500°C, and the cone gas was set at 50 l h^−1^. The capillary voltages under positive and negative modes were set at 5.0 kV and −4.5 kV, respectively. Argon was used as the collision gas. The cone voltages were both set at 35 V, but the energies for collision-induced dissociation in positive and negative ion modes were set at 5.0 eV and 7.0 eV respectively for the fragmentation information.

Scavenging of ·OH by excess benzene was introduced into different reaction systems to determine the ·OH quantum yield under irradiation of a 500 W Hg lamp. Phenol produced from the reaction of benzene and ·OH was detected at 254 nm by HPLC, in which 25% (v/v) acetonitrile was used as a mobile phase at a flowing rate of 1.0 ml min^−1^ under isocratic conditions at 25°C. Samples of 10 µl were injected into the column through the sample loop for analysis [25].

Analyses of sulfate ion and nitrate ion were performed according to standard methods proposed by PRC State Environmental Protection Administration [27].

### 2.4. Kinetic study

The kinetic description of BTH degradation processes through the pseudo-first-order approach was made, and the first-order rate constants of phototransformation (*k*[s^−1^]) of the investigated compound were obtained by linear regression of the natural logarithmic relative residual concentration over irradiation time *t*[s], which is described by the following equation:

2.1 $$kt=\ln\;\left(\frac{C_{0}}{C_{t}}\right),$$

where *C_t_* is the concentration of BTH at given time, *C*_0_ is the initial concentration, and *k* is the rate constant.

### 2.5. Calculation of the frontier electron density of benzothiazole

By means of the calculation of BTH at the B3LYP/6-311G** level with the density functional theory method, the frontier electron densities (FEDs) of the highest occupied molecular orbital (HOMO) and the lowest unoccupied molecular orbital (LUMO) were both obtained. For the purpose of predicting the reaction sites for hydroxyl addition, values of $\text{FE}{{\text{D}}^{2}}_{\mathrm{HOMO}}+\text{FE}{{\text{D}}^{2}}_{\mathrm{LUMO}}$ were also calculated.

## 3. Results and discussion

### 3.1. Photodegradation of benzothiazole in different systems under UV light irradiation

**Figure 1.**
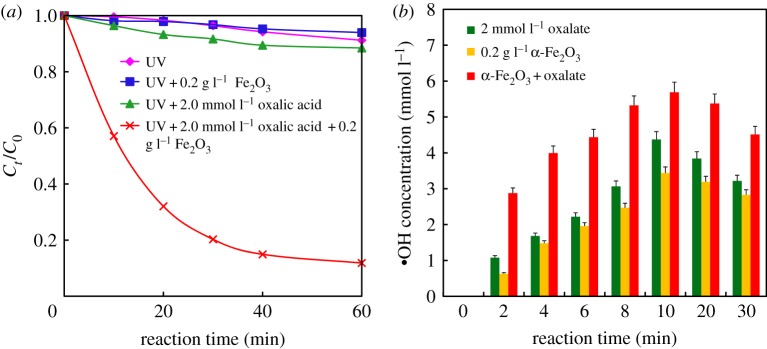
Photodegradation of 100 mg l^−1^ benzothiazole under UV irradiation in 50 ml solutions (pH = 2 ) (*a*) and the production of hydroxyl radicals (·OH) in different reaction systems under UV irradiation (500 W, Hg lamp, pH = 2) (*b*).

The photodegradation of BTH in different systems is shown in figure 1*a*. With the absence of oxalic acid and α-Fe_2_O_3_, the photodegradation rate was only 8.8% under UV light (500 W, Hg lamp) in 60 min. While the removal percentage of BTH dropped to 3.0% when 0.2 g l^−1^ α-Fe_2_O_3_ was added under the otherwise same conditions, and the removal of BTH increased up to 11.5% when just oxalic acid (2.0 mmol l^−1^) was added under UV light. However, when 2.0 mmol l^−1^ oxalic acid and 0.2 g l^−1^ α-Fe_2_O_3_ were simultaneously added into the reaction system under UV irradiation, the removal percentage of BTH significantly increased up to 88.1%. Therefore, with only α-Fe_2_O_3_ or only oxalic acid, the reaction system shows low photocatalytic activities for BTH degradation. While BTH can be efficiently degraded with the synergistic effect of iron oxides and oxalate under UV light irradiation, for the reason of a heterogeneous photochemical Fenton-like system being set up. It is reported that fenuron [17] and mesotrione [25] can be efficiently photodegraded in such a system.

### 3.2. Production of hydroxyl radicals in different reaction systems

The generation of hydroxyl radicals (·OH) in photochemical reactions with high oxidation potential is critical to the degradation of organic pollutants. Particularly, yield of ·OH could be an indicator for photochemical degradation in the α-Fe_2_O_3_/oxalate system. Therefore, the concentration of ·OH was detected during the photochemical reaction process in the present study. The concentration of ·OH in the reaction system depends on the rates of generation and consumption. As shown in figure 1*b*, the yield of ·OH produced in the system of α-Fe_2_O_3_ or oxalate alone is much lower than that with coexistence of α-Fe_2_O_3_ and oxalate system. The ·OH was generated quickly in the initial 10 min, and the maximum ·OH concentration detected was about 6 μmol l^−1^ after 10 min.

**Figure 2.**
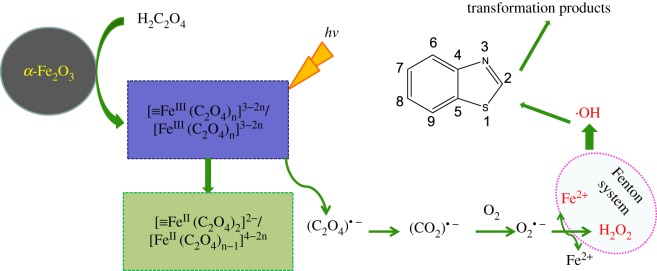
Potential pathway of the photochemical reaction in α-Fe_2_O_3_/oxalate complex system.

To understand the photochemical reaction process of BTH degradation in such a α-Fe_2_O_3_/oxalate complex system, the interaction of α-Fe_2_O_3_ and oxalate under UV light irradiation was discussed in detail [26] (figure 2). Firstly, oxalic acid was adsorbed on the surface of α-Fe_2_O_3_ particles, which accelerates the formation of α-Fe_2_O_3_/oxalate complexes, [≡Fe^III^(C_2_O_4_)_*n*_]^3−2*n*^, and a part of these complexes are dissolved in the solution. [≡Fe^III^(C_2_O_4_)_*n*_]^3−2*n*^ on the α-Fe_2_O_3_ particle surface and in the solution both possessed high photochemical activity, which is easy to be excited to generate oxalate radicals (C_2_O_4_)^• −^ and transferred into carbon-centred radicals (CO_2_)^• −^. The excited electrons were transferred from CO_2_^• −^ to the adsorbed oxygen forming superoxide ion (O_2_^• −^), which reacted with Fe^3+^ to form O_2_ and Fe^2+^. In the acidic solution, O_2_^• −^ reacted with Fe^2+^ to form H_2_O_2_ and Fe^3+^. Thus, ·OH could be formed through the reaction of H_2_O_2_ with Fe^2+^. At the same time, Fe^3+^ also formed. Finally, BTH was oxidized by ·OH with strong oxidation potential. As reported by Balmer & Sulzberger [28], when the oxalate concentration was more than 0.18 mmol l^−1^ in the Fe^3+^/oxalate system, Fe(III) mainly existed as $\text{Fe}{{\left({\text{C}}_{2}{\text{O}}_{4}\right)}_{2}}^{\_}$ and $\text{Fe}{{\left({\text{C}}_{2}{\text{O}}_{4}\right)}_{3}}^{3-}$, both of which could be more efficiently photolysed than other Fe(III) species. Therefore, the BTH photodegradation was improved significantly in the system of oxalic acid and α-Fe_2_O_3_.

Radical quenching experiments are very useful methods for proving the effect of hydroxyl radical. Chen *et al*. [25] selected benzene as the hydroxyl radical scavenger to show that ·OH produced from the photocatalysis was the key to lead the degradation of organics.

### 3.3. Effect of the dosage of α-Fe_2_O_3_ on benzothiazole photodegradation

**Figure 3.**
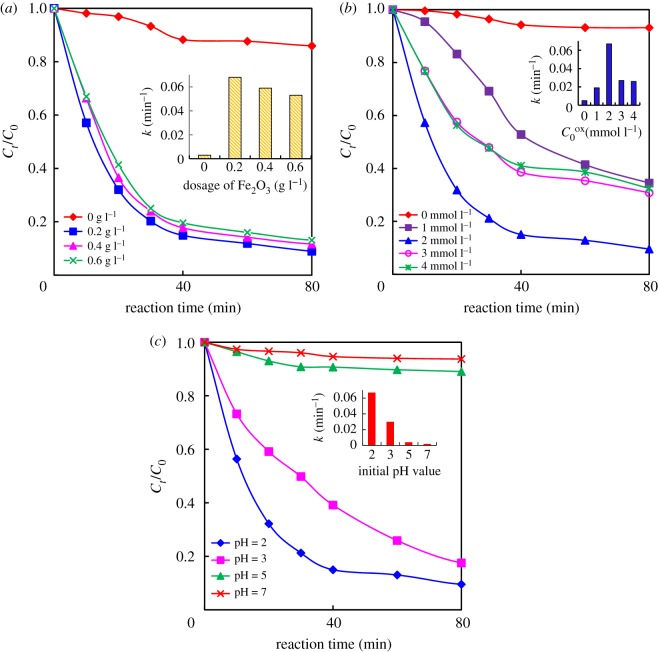
Effect of α-Fe_2_O_3_ dosage (*a*), initial concentration of oxalate (*b*) and pH value (*c*) on the photodegradation of 100 mg l^−1^ BTH under UV irradiation (500 W, Hg lamp). (Insets present the dependence of *k*.)

As shown in figure 3*a*, the effect of α-Fe_2_O_3_ dosage on BTH photodegradation was investigated in the presence of oxalic acid with an initial concentration of 2.0 mmol l^−1^ under irradiation of a 500 W high-pressure mercury lamp.

With the absence of α-Fe_2_O_3_, the degradation of BTH was very slow, and the degradation rate was only 12.3% (curve 0.0 g l^−1^). Nevertheless, the degradation of BTH was obviously accelerated after adding α-Fe_2_O_3_ in the reaction system, indicating that α-Fe_2_O_3_ was an effective photocatalyst for BTH degradation with the assistance of oxalic acid. The removal percentage of BTH rose up to 92.88% when the concentration of α-Fe_2_O_3_ was increased to 0.2 g l^−1^. However, the removal percentage declined slightly when the dosage of α-Fe_2_O_3_ increased from 0.2 to 0.6 g l^−1^, because the excessive amount of α-Fe_2_O_3_ might restrain the UV light scattering in the reaction suspension and reduce the generation of ·OH.

The kinetics of the reaction process was also studied. The photodegradation of BTH in the α-Fe_2_O_3_/oxalate system under UV irradiation was in accordance with first-order kinetics. The first-order kinetic constants (*k*) were calculated to be 0.5 × 10^−2^, 6.8 × 10^−2^, 5.9 × 10^−2^ and 5.3 × 10^−2^ min^−1^ with 0.0, 0.2, 0.4 and 0.6 g l^−1^ α-Fe_2_O_3_, respectively. The changes of *k* versus α-Fe_2_O_3_ dosage (figure 3*a* inset) reveal that the optimum concentration of α-Fe_2_O_3_ was 0.2 g l^−1^ in the proposed α-Fe_2_O_3_/oxalate system for the best BTH photodegradation performance. As a heterogeneous photocatalyst, α-Fe_2_O_3_ could remarkably accelerate the generation of [≡Fe(C_2_O_4_)_*n*_]^3−2*n*^. Under UV irradiation, ·OH could be produced more with more [≡Fe(C_2_O_4_)_*n*_]^3−2*n*^ generated during the photochemical reaction. However, excessive dosage of α-Fe_2_O_3_ might restrict the penetration of UV light in the solution and decrease UV light intensity, which is confirmed by Wu *et al*. [29].

### 3.4. Dependence of the benzothiazole photodegradation on the oxalate initial concentration

In order to survey the effect of the oxalate initial concentration (${C_{0}}^{\mathrm{ox}}$) on the photodegradation of BTH, experiments were carried out with the initial BTH of 100 mg l^−1^ and α-Fe_2_O_3_ dosage of 0.2 g l^−1^ under UV irradiation (500 W Hg lamp). The results are shown in figure 3*b*. In the absence of oxalate, BTH was degraded slowly and the concentration of BTH almost unchanged under the irradiation for 60 min (curve 0.0 mmol l^−1^). However, The rate of BTH photodegradation was improved markedly as a consequence of oxalate increase in the suspension of α-Fe_2_O_3_/oxalate. However, the degradation rate of BTH is not always increased with the initial oxalate concentration, which means excessive oxalate could inhibit the degradation of BTH. The excessive oxalate would lead to the occupation of the adsorbed sites on the iron oxide surface. Besides, the excessive oxalate also can result in a lower pH at the beginning, so a large amount of Fe^3+^ would form [26,30].

The photodegradation of BTH in the α-Fe_2_O_3_/oxalate system was fitted with first-order kinetics and the first-order kinetic constants (*k*) versus ${C_{0}}^{\mathrm{ox}}$ are shown in figure 3*b* (inset). When the initial concentrations of oxalic acid were 0.0, 1.0, 2.0, 3.0 and 4.0 mmol l^−1^, the *k* values of BTH degradation were calculated to be 0.5 × 10^−2^, 1.9 × 10^−2^, 6.7 × 10^−2^, 2.7 × 10^−2^ and 2.6 × 10^−2^, respectively. The results revealed that the BTH photodegradation rate increased with initial oxalic acid concentration increase firstly, but reached maximum value when the initial concentration of oxalic acid was increased to 2.0 mmol l^−1^. Therefore, it is necessary to control the concentrations of α-Fe_2_O_3_ and oxalate for BTH photodegradation, because excessive oxalic acid would overwhelmingly occupy the active sites on the surface of α-Fe_2_O_3_ and facilitate the competitive reaction with the generated ·OH, while less oxalic acid would lead to incomplete reaction.

### 3.5. Effect of the initial pH value on benzothiazole photodegradation

To study the effect of the initial pH value on BTH photodegradation, a series of experiments were carried out in this study. Initial pH of the solution was adjusted by NaOH or H_2_SO_4_ before reaction. And the initial concentration of BTH is 100 mg l^−1^ with the presence of 0.2 g l^−1^ α-Fe_2_O_3_ and 2.0 mmol l^−1^ oxalic acid under UV irradiation (500 W Hg lamp). At pH = 7.0, the degradation efficiency of BTH changes less. When the pH value was decreased, the degradation efficiency is gradually improved. Especially, when pH value reaches 2.0, the degradation efficiency is increased to maximum value of 90.57% (figure 3*c*). The first-order kinetic constants (*k*) were 6.7 × 10^−2^, 2.1 × 10^−2^, 0.4 × 10^−2^, 0.2 × 10^−2^ when the initial pH values were 2.0, 3.0, 5.0, 7.0, respectively. In system of α-Fe_2_O_3_/oxalate/UV, a high concentration of [≡Fe(C_2_O_4_)_*n*_]^3−2*n*^ with high photocatalytic activity might appear at a lower pH value.

### 3.6. Identification of the photodegradation intermediates and products

**Figure 4.**
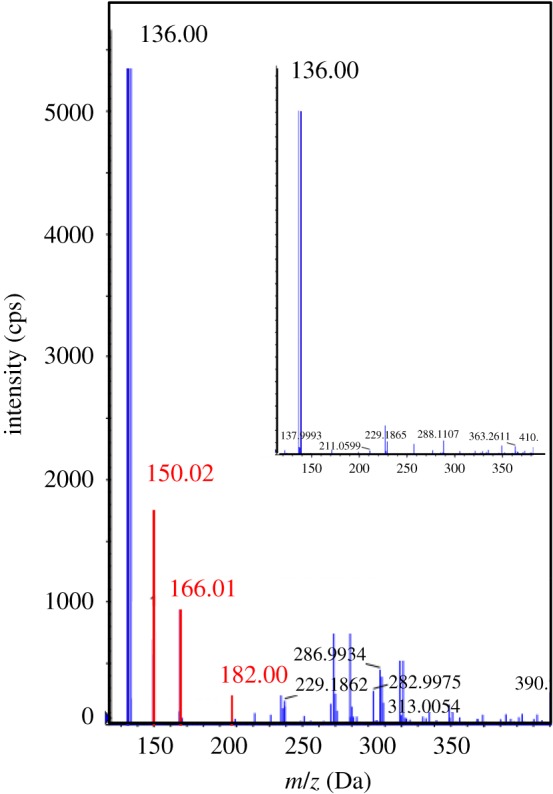
Mass spectra of benzothiazole photodegradation solution after 50 min and 0 min (inset). (Reaction conditions: 100 mg l^−1^ BTH under UV irradiation in presence of 0.2 g l^−1^ Fe_2_O_3_, 2 mmol l^−1^ oxalate and pH = 2.)

Various TPs are often produced in advanced oxidation processes, because the reaction between ·OH and organic pollutants is non-selective. Degradation intermediates were determined by UPLC and QTOF analysis. And the chromatographic retention time, relative molecular weight and ion information of the intermediates were comprehensively analysed using the method of extracting mass spectrometry. Based on the comparison of the mass spectra of the photodegradation solution at 0 min and 50 min during the reaction process, a host of new peaks appeared (figure 4). The major TPs included such hydroxylation products as the mono-hydroxylated BTH with mass–charge ratio (*m/z*) of 150.02, di-hydroxylated BTH at *m/z* 166.01 and tri-hydroxylated BTH at *m/z* 182.00, among which the peaks at *m/z* 150.02 might also correspond to benzothiazol-2(3H)-one.

To correctly characterize the positions of hydroxylation in mono-hydroxylated compounds, the FEDs of BTH were calculated to predict the reaction sites for ·OH attack. The results are summarized in table 1. According to the frontier orbital theory, the prior ·OH addition probably occurs on the atom with the highest $\text{FE}{{\text{D}}^{2}}_{\mathrm{HOMO}}+\text{FE}{{\text{D}}^{2}}_{\mathrm{LUMO}}$ value [31], which has been testified to be reasonable by published work [32]. As shown in table 1, 6C, 8C and 9C sites in phenyl ring and 2C in thiazole had the highest $\text{FE}{{\text{D}}^{2}}_{\mathrm{HOMO}}+\text{FE}{{\text{D}}^{2}}_{\mathrm{LUMO}}$ value, suggesting benzene was likely to be attacked by ·OH, thus resulting in the generation of mono-hydroxylation products. However, it should be noted that the possibility for ·OH addition to thiazole moiety is much higher than addition to phenyl moiety.

**Table 1.** $\text{FE}{{\text{D}}^{2}}_{\mathrm{HOMO}}+\text{FE}{{\text{D}}^{2}}_{\mathrm{LUMO}}$ values of BTH atoms calculated at the B3LYP/6-311G** level using Gaussian 09 program.

number (atom)^a^	$\text{FE}{{\text{D}}^{2}}_{\mathrm{HOMO}}+\text{FE}{{\text{D}}^{2}}_{\mathrm{LUMO}}$	number (atom)	$\text{FE}{{\text{D}}^{2}}_{\mathrm{HOMO}}+\text{FE}{{\text{D}}^{2}}_{\mathrm{LUMO}}$
1S	0.294443	*6C*^b^	*0.205303*
*2C*^b^	*0.315164*	7C	0.0045209
3N	0.148739	*8C*^b^	*0.167837*
4C	0.076377	*9C*^b^	*0.184021*
5C	0.032246		

^a^See figure 2 for atom numbering.

^b^Atoms in italics means these atoms have the highest $\text{FE}{{\text{D}}^{2}}_{\mathrm{HOMO}}+\text{FE}{{\text{D}}^{2}}_{\mathrm{LUMO}}$ values, thus are more likely to be attacked by hydroxyl radical.

**Figure 5.**
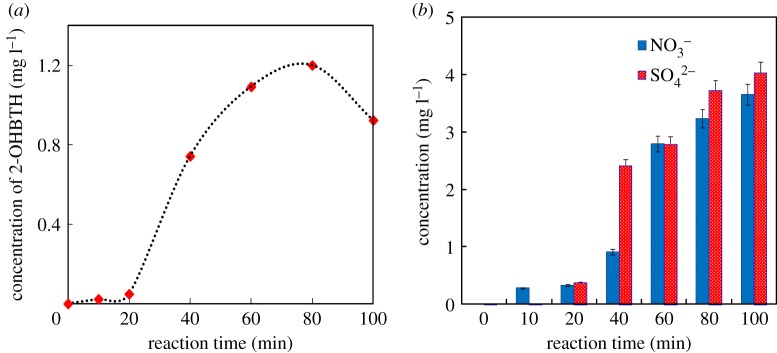
Concentration curve of benzothiazol-2(3H)-one (*a*), and concentrations of ${{\text{NO}}_{3}}^{-}$ and ${{\mathrm{SO}}_{4}}^{2-}$ (*b*). (Reaction conditions: 100 mg l^−1^ BTH under UV irradiation in presence of 0.2 g l^−1^ Fe_2_O_3_, 2 mmol l^−1^ oxalate and pH = 2.)

The concentration change of benzothiazol-2(3H)-one, one of the intermediates, was determined by liquid chromatography (LC), as shown in figure 5*a*. As seen, the concentration of benzothiazol-2(3H)-one increased with time during 20–70 min followed by a gradual decay, indicating that the formation and transformation of benzothiazol-2(3H)-one were accompanied with the degradation of BTH.

The concentration change of inorganic ions during the BTH photocatalysis process is depicted in figure 5*b*. As clearly seen, the sulfur atom and nitrogen atom in the thiazole structure could be converted to sulfate ions $\left({{\mathrm{SO}}_{4}}^{2-}\right)$ and nitrate $\left({{\text{NO}}_{3}}^{-}\right)$, respectively. Thus it was illustrated that intermediates can be decomposed ultimately.

**Figure 6.**
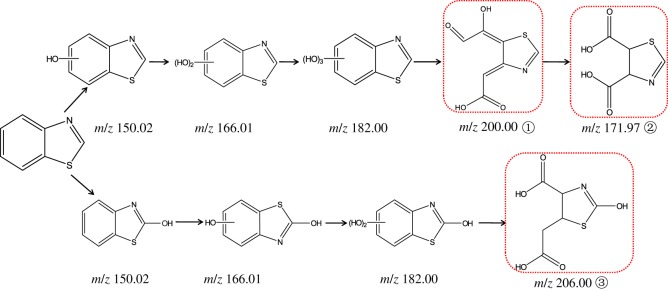
Possible photodegradation pathway of BTH in UV irradiated α-Fe_2_O_3_/oxalate system (TPs marked with dashed frame were detected in none of the samples, but Borowska *et al.* [11] had detected ① and ②).

Data obtained above were used to propose a schematic pathway of BTH degradation by α-Fe_2_O_3_/oxalate (figure 6). The degradation of BTH starts with the hydroxylation, and then produces mono-, di- or tri-hydroxylated BTH. However, hydroxylation of the aromatic ring makes it more unstable and prone to ring opening. The oxidation products of tri-hydroxylated BTH may be found at *m/z* 200.00 or 171.97 (neither of them has been detected in the samples, but reported in the literature [11]), and the latter corresponds to the loss of one atom of carbon and gain of four atoms of oxygen, which suggests the benzene ring opening and subsequent decarboxylation [33].

## 4. Conclusion

The photocatalytic degradation of BTH has been investigated in UV irradiated α-Fe_2_O_3_/oxalate system in this study, as a photo-Fenton-like system without additional H_2_O_2_. The optimum degradation conditions were found to be: initial pH 2.0, α-Fe_2_O_3_ dosage 0.2 g l^−1^ and initial oxalate concentration 2.0 mmol l^−1^ under 500 W of UV light irradiation (Hg lamp). Photocatalysis reactions followed pseudo-first-order kinetics. Organic transformation products were identified by LC–MS analysis, and the major photoproducts included hydroxylated products, benzene ring cleavage compounds and phenylimidazolecarboxylic derivatives. The calculation of FEDs predicted that the benzene in BTH was likely to be attacked by ·OH, resulting in the formation of mono-hydroxylation products. Sulfur atom of BTH was converted to a sulfate ion while nitrogen atom was released as nitrate, implying that intermediates can be decomposed further after a certain irradiation time. The results obtained in this study are helpful to understand the environmental fates of BTH, and also can provide a viable technology for BTH removal from water.

## Supplementary Material

Q-chem output file

## Data accessibility

The data supporting the findings of this study are available in the electronic supplementary material.
