# Supplementary material for: Benzothiazole heterogeneous photodegradation in nano α-Fe2O3/oxalate system under UV light irradiation
Source: R Soc Open Sci. 2018 Jun 27;5(6):180322. doi: 10.1098/rsos.180322 (PMC6030283; doi:10.1098/rsos.180322)
Supplement: Q-chem output file [file rsos180322supp1.doc]

Entering Link 1 = C:\G09W\l1.exe PID= 5964.

Copyright (c) 1988,1990,1992,1993,1995,1998,2003,2009, Gaussian, Inc.

All Rights Reserved.

This is part of the Gaussian(R) 09 program. It is based on

the Gaussian(R) 03 system (copyright 2003, Gaussian, Inc.),

the Gaussian(R) 98 system (copyright 1998, Gaussian, Inc.),

the Gaussian(R) 94 system (copyright 1995, Gaussian, Inc.),

the Gaussian 92(TM) system (copyright 1992, Gaussian, Inc.),

the Gaussian 90(TM) system (copyright 1990, Gaussian, Inc.),

the Gaussian 88(TM) system (copyright 1988, Gaussian, Inc.),

the Gaussian 86(TM) system (copyright 1986, Carnegie Mellon

University), and the Gaussian 82(TM) system (copyright 1983,

Carnegie Mellon University). Gaussian is a federally registered

trademark of Gaussian, Inc.

This software contains proprietary and confidential information,

including trade secrets, belonging to Gaussian, Inc.

This software is provided under written license and may be

used, copied, transmitted, or stored only in accord with that

written license.

The following legend is applicable only to US Government

contracts under FAR:

RESTRICTED RIGHTS LEGEND

Use, reproduction and disclosure by the US Government is

subject to restrictions as set forth in subparagraphs (a)

and (c) of the Commercial Computer Software - Restricted

Rights clause in FAR 52.227-19.

Gaussian, Inc.

340 Quinnipiac St., Bldg. 40, Wallingford CT 06492

---------------------------------------------------------------

Warning -- This program may not be used in any manner that

competes with the business of Gaussian, Inc. or will provide

assistance to any competitor of Gaussian, Inc. The licensee

of this program is prohibited from giving any competitor of

Gaussian, Inc. access to this program. By using this program,

the user acknowledges that Gaussian, Inc. is engaged in the

business of creating and licensing software in the field of

computational chemistry and represents and warrants to the

licensee that it is not a competitor of Gaussian, Inc. and that

it will not use this program in any manner prohibited above.

---------------------------------------------------------------

Cite this work as:

Gaussian 09, Revision A.02,

M. J. Frisch, G. W. Trucks, H. B. Schlegel, G. E. Scuseria,

M. A. Robb, J. R. Cheeseman, G. Scalmani, V. Barone, B. Mennucci,

G. A. Petersson, H. Nakatsuji, M. Caricato, X. Li, H. P. Hratchian,

A. F. Izmaylov, J. Bloino, G. Zheng, J. L. Sonnenberg, M. Hada,

M. Ehara, K. Toyota, R. Fukuda, J. Hasegawa, M. Ishida, T. Nakajima,

Y. Honda, O. Kitao, H. Nakai, T. Vreven, J. A. Montgomery, Jr.,

J. E. Peralta, F. Ogliaro, M. Bearpark, J. J. Heyd, E. Brothers,

K. N. Kudin, V. N. Staroverov, R. Kobayashi, J. Normand,

K. Raghavachari, A. Rendell, J. C. Burant, S. S. Iyengar, J. Tomasi,

M. Cossi, N. Rega, J. M. Millam, M. Klene, J. E. Knox, J. B. Cross,

V. Bakken, C. Adamo, J. Jaramillo, R. Gomperts, R. E. Stratmann,

O. Yazyev, A. J. Austin, R. Cammi, C. Pomelli, J. W. Ochterski,

R. L. Martin, K. Morokuma, V. G. Zakrzewski, G. A. Voth,

P. Salvador, J. J. Dannenberg, S. Dapprich, A. D. Daniels,

O. Farkas, J. B. Foresman, J. V. Ortiz, J. Cioslowski,

and D. J. Fox, Gaussian, Inc., Wallingford CT, 2009.

******************************************

Gaussian 09: IA32W-G09RevA.02 11-Jun-2009

28-Jul-2017

******************************************

%chk=d7.chk

%nproc=3

Will use up to 3 processors via shared memory.

----------------------------------

#n b3lyp/6-311G** opt freq pop=reg

----------------------------------

1/14=-1,18=20,19=15,26=3,38=1/1,3;

2/9=110,12=2,17=6,18=5,40=1/2;

3/5=4,6=6,7=101,11=2,16=1,25=1,30=1,71=1,74=-5/1,2,3;

4//1;

5/5=2,38=5/2;

6/28=1/1;

7//1,2,3,16;

1/14=-1,18=20,19=15/3(2);

2/9=110/2;

99//99;

2/9=110/2;

3/5=4,6=6,7=101,11=2,16=1,25=1,30=1,71=1,74=-5/1,2,3;

4/5=5,16=3/1;

5/5=2,38=5/2;

7//1,2,3,16;

1/14=-1,18=20,19=15/3(-5);

2/9=110/2;

6/19=2,28=1/1;

99/9=1/99;

----

ssss

----

Symbolic Z-matrix:

Charge = 0 Multiplicity = 1

S 0 -1.83557 1.37513 -0.84518

C 0 -2.18485 -0.19963 -0.84518

N 0 -1.46215 -1.23176 -0.84518

C 0 -0.10286 -0.70998 -0.84518

C 0 -0.10286 0.71001 -0.84518

C 0 0.93019 -1.41997 -0.17804

C 0 1.96323 -0.70998 0.48911

C 0 2.35648 0.70995 -0.8717

C 0 1.12683 1.41997 -0.85844

H 0 -3.26369 -0.41417 -0.85329

H 0 0.92502 -2.51992 -0.17004

H 0 2.42647 -0.89481 1.46953

H 0 3.26369 0.88187 -1.46953

H 0 1.12675 2.51992 -0.86797

GradGradGradGradGradGradGradGradGradGradGradGradGradGradGradGradGradGrad

Berny optimization.

Initialization pass.

----------------------------

! Initial Parameters !

! (Angstroms and Degrees) !

-------------------------- --------------------------

! Name Definition Value Derivative Info. !

--------------------------------------------------------------------------------

! R1 R(1,2) 1.613 estimate D2E/DX2 !

! R2 R(1,5) 1.856 estimate D2E/DX2 !

! R3 R(2,3) 1.26 estimate D2E/DX2 !

! R4 R(2,10) 1.1 estimate D2E/DX2 !

! R5 R(3,4) 1.456 estimate D2E/DX2 !

! R6 R(4,5) 1.42 estimate D2E/DX2 !

! R7 R(4,6) 1.42 estimate D2E/DX2 !

! R8 R(5,9) 1.42 estimate D2E/DX2 !

! R9 R(6,7) 1.42 estimate D2E/DX2 !

! R10 R(6,11) 1.1 estimate D2E/DX2 !

! R11 R(7,12) 1.1 estimate D2E/DX2 !

! R12 R(8,9) 1.42 estimate D2E/DX2 !

! R13 R(8,13) 1.1 estimate D2E/DX2 !

! R14 R(9,14) 1.1 estimate D2E/DX2 !

! A1 A(2,1,5) 81.5056 estimate D2E/DX2 !

! A2 A(1,2,3) 132.4944 estimate D2E/DX2 !

! A3 A(1,2,10) 113.7521 estimate D2E/DX2 !

! A4 A(3,2,10) 113.7521 estimate D2E/DX2 !

! A5 A(2,3,4) 104.0 estimate D2E/DX2 !

! A6 A(3,4,5) 111.0 estimate D2E/DX2 !

! A7 A(3,4,6) 120.0 estimate D2E/DX2 !

! A8 A(5,4,6) 120.0 estimate D2E/DX2 !

! A9 A(1,5,4) 111.0 estimate D2E/DX2 !

! A10 A(1,5,9) 128.998 estimate D2E/DX2 !

! A11 A(4,5,9) 119.9986 estimate D2E/DX2 !

! A12 A(4,6,7) 120.0 estimate D2E/DX2 !

! A13 A(4,6,11) 119.9987 estimate D2E/DX2 !

! A14 A(7,6,11) 119.9988 estimate D2E/DX2 !

! A15 A(6,7,12) 129.8753 estimate D2E/DX2 !

! A16 A(9,8,13) 129.8748 estimate D2E/DX2 !

! A17 A(5,9,8) 120.0 estimate D2E/DX2 !

! A18 A(5,9,14) 119.9988 estimate D2E/DX2 !

! A19 A(8,9,14) 119.9987 estimate D2E/DX2 !

! D1 D(5,1,2,3) 0.0 estimate D2E/DX2 !

! D2 D(5,1,2,10) -179.5385 estimate D2E/DX2 !

! D3 D(2,1,5,4) 0.0 estimate D2E/DX2 !

! D4 D(2,1,5,9) 179.3117 estimate D2E/DX2 !

! D5 D(1,2,3,4) 0.0 estimate D2E/DX2 !

! D6 D(10,2,3,4) 179.5385 estimate D2E/DX2 !

! D7 D(2,3,4,5) 0.0 estimate D2E/DX2 !

! D8 D(2,3,4,6) 147.1454 estimate D2E/DX2 !

! D9 D(3,4,5,1) 0.0 estimate D2E/DX2 !

! D10 D(3,4,5,9) -179.3824 estimate D2E/DX2 !

! D11 D(6,4,5,1) -147.1454 estimate D2E/DX2 !

! D12 D(6,4,5,9) 33.4722 estimate D2E/DX2 !

! D13 D(3,4,6,7) -144.2091 estimate D2E/DX2 !

! D14 D(3,4,6,11) 35.2179 estimate D2E/DX2 !

! D15 D(5,4,6,7) 0.0 estimate D2E/DX2 !

! D16 D(5,4,6,11) 179.4271 estimate D2E/DX2 !

! D17 D(1,5,9,8) -179.258 estimate D2E/DX2 !

! D18 D(1,5,9,14) 0.169 estimate D2E/DX2 !

! D19 D(4,5,9,8) 0.0 estimate D2E/DX2 !

! D20 D(4,5,9,14) 179.427 estimate D2E/DX2 !

! D21 D(4,6,7,12) 137.3185 estimate D2E/DX2 !

! D22 D(11,6,7,12) -42.1085 estimate D2E/DX2 !

! D23 D(13,8,9,5) 135.8491 estimate D2E/DX2 !

! D24 D(13,8,9,14) -43.578 estimate D2E/DX2 !

--------------------------------------------------------------------------------

Trust Radius=3.00D-01 FncErr=1.00D-07 GrdErr=1.00D-06

Number of steps in this run= 67 maximum allowed number of steps= 100.

GradGradGradGradGradGradGradGradGradGradGradGradGradGradGradGradGradGrad

Input orientation:

---------------------------------------------------------------------

Center Atomic Atomic Coordinates (Angstroms)

Number Number Type X Y Z

---------------------------------------------------------------------

1 16 0 -1.835572 1.375133 -0.845184

2 6 0 -2.184849 -0.199630 -0.845184

3 7 0 -1.462146 -1.231757 -0.845184

4 6 0 -0.102858 -0.709976 -0.845184

5 6 0 -0.102858 0.710007 -0.845184

6 6 0 0.930186 -1.419967 -0.178038

7 6 0 1.963230 -0.709976 0.489108

8 6 0 2.356481 0.709945 -0.871696

9 6 0 1.126829 1.419967 -0.858440

10 1 0 -3.263685 -0.414170 -0.853294

11 1 0 0.925018 -2.519917 -0.170036

12 1 0 2.426474 -0.894806 1.469528

13 1 0 3.263685 0.881873 -1.469528

14 1 0 1.126754 2.519917 -0.867966

---------------------------------------------------------------------

Distance matrix (angstroms):

1 2 3 4 5

1 S 0.000000

2 C 1.613032 0.000000

3 N 2.633500 1.259994 0.000000

4 C 2.711084 2.143628 1.455994 0.000000

5 C 1.855988 2.272031 2.370256 1.419983 0.000000

6 C 3.988369 3.411415 2.490735 1.419983 2.459482

7 C 4.534194 4.387180 3.712922 2.459483 2.839966

8 C 4.244583 4.631599 4.284019 2.839934 2.459482

9 C 2.962770 3.686527 3.706023 2.459464 1.419982

10 H 2.289362 1.099991 1.978398 3.174649 3.354798

11 H 4.821628 3.938379 2.795306 2.188205 3.456120

12 H 5.354956 5.206291 4.537928 3.433595 3.785609

13 H 5.160962 5.589810 5.214472 3.775897 3.428258

14 H 3.175913 4.285227 4.558287 3.456107 2.188204

6 7 8 9 10

6 C 0.000000

7 C 1.419983 0.000000

8 C 2.655561 2.005644 0.000000

9 C 2.926917 2.655581 1.419983 0.000000

10 H 4.365335 5.404645 5.731513 4.758224 0.000000

11 H 1.099991 2.188205 3.601864 4.004661 4.737747

12 H 2.286733 1.099991 2.839270 3.530819 6.164773

13 H 3.523015 2.839264 1.099992 2.286729 6.683265

14 H 4.004663 3.601882 2.188205 1.099991 5.280628

11 12 13 14

11 H 0.000000

12 H 2.753820 0.000000

13 H 4.327842 3.534906 0.000000

14 H 5.091928 4.337451 2.758902 0.000000

Stoichiometry C7H5NS

Framework group C1[X(C7H5NS)]

Deg. of freedom 36

Full point group C1 NOp 1

Largest Abelian subgroup C1 NOp 1

Largest concise Abelian subgroup C1 NOp 1

Standard orientation:

---------------------------------------------------------------------

Center Atomic Atomic Coordinates (Angstroms)

Number Number Type X Y Z

---------------------------------------------------------------------

1 16 0 1.872776 -0.861207 0.236834

2 6 0 1.935113 0.727536 -0.034977

3 7 0 1.050366 1.577117 -0.323110

4 6 0 -0.179843 0.800184 -0.376771

5 6 0 0.070282 -0.568958 -0.095276

6 6 0 -1.410689 1.391700 0.012432

7 6 0 -2.391409 0.614074 0.683130

8 6 0 -2.317806 -1.068914 -0.405329

9 6 0 -0.998716 -1.503510 -0.109557

10 1 0 2.947886 1.150332 0.039284

11 1 0 -1.600626 2.454688 -0.197235

12 1 0 -3.022951 0.868048 1.547210

13 1 0 -3.079423 -1.520548 -1.057974

14 1 0 -0.803449 -2.565705 0.099232

---------------------------------------------------------------------

Rotational constants (GHZ): 2.8308641 1.2996419 0.9451992

Standard basis: 6-311G(d,p) (5D, 7F)

There are 200 symmetry adapted basis functions of A symmetry.

Integral buffers will be 262144 words long.

Raffenetti 2 integral format.

Two-electron integral symmetry is turned on.

200 basis functions, 342 primitive gaussians, 209 cartesian basis functions

35 alpha electrons 35 beta electrons

nuclear repulsion energy 460.9643240241 Hartrees.

NAtoms= 14 NActive= 14 NUniq= 14 SFac= 7.50D-01 NAtFMM= 80 NAOKFM=F Big=F

One-electron integrals computed using PRISM.

NBasis= 200 RedAO= T NBF= 200

NBsUse= 200 1.00D-06 NBFU= 200

Harris functional with IExCor= 402 diagonalized for initial guess.

ExpMin= 7.71D-02 ExpMax= 9.34D+04 ExpMxC= 3.17D+03 IAcc=2 IRadAn= 0 AccDes= 0.00D+00

HarFok: IExCor= 402 AccDes= 0.00D+00 IRadAn= 0 IDoV= 1

ScaDFX= 1.000000 1.000000 1.000000 1.000000

FoFCou: FMM=F IPFlag= 0 FMFlag= 100000 FMFlg1= 0

NFxFlg= 0 DoJE=T BraDBF=F KetDBF=T FulRan=T

Omega= 0.000000 0.000000 1.000000 0.000000 0.000000 ICntrl= 500 IOpCl= 0

NMat0= 1 NMatS0= 1 NMatT0= 0 NMatD0= 1 NMtDS0= 0 NMtDT0= 0

I1Cent= 4 NGrid= 0.

Petite list used in FoFCou.

Initial guess orbital symmetries:

Occupied (A) (A) (A) (A) (A) (A) (A) (A) (A) (A) (A) (A)

(A) (A) (A) (A) (A) (A) (A) (A) (A) (A) (A) (A)

(A) (A) (A) (A) (A) (A) (A) (A) (A) (A) (A)

Virtual (A) (A) (A) (A) (A) (A) (A) (A) (A) (A) (A) (A)

(A) (A) (A) (A) (A) (A) (A) (A) (A) (A) (A) (A)

(A) (A) (A) (A) (A) (A) (A) (A) (A) (A) (A) (A)

(A) (A) (A) (A) (A) (A) (A) (A) (A) (A) (A) (A)

(A) (A) (A) (A) (A) (A) (A) (A) (A) (A) (A) (A)

(A) (A) (A) (A) (A) (A) (A) (A) (A) (A) (A) (A)

(A) (A) (A) (A) (A) (A) (A) (A) (A) (A) (A) (A)

(A) (A) (A) (A) (A) (A) (A) (A) (A) (A) (A) (A)

(A) (A) (A) (A) (A) (A) (A) (A) (A) (A) (A) (A)

(A) (A) (A) (A) (A) (A) (A) (A) (A) (A) (A) (A)

(A) (A) (A) (A) (A) (A) (A) (A) (A) (A) (A) (A)

(A) (A) (A) (A) (A) (A) (A) (A) (A) (A) (A) (A)

(A) (A) (A) (A) (A) (A) (A) (A) (A) (A) (A) (A)

(A) (A) (A) (A) (A) (A) (A) (A) (A)

The electronic state of the initial guess is 1-A.

Requested convergence on RMS density matrix=1.00D-08 within 128 cycles.

Requested convergence on MAX density matrix=1.00D-06.

Requested convergence on energy=1.00D-06.

No special actions if energy rises.

Integral accuracy reduced to 1.0D-05 until final iterations.

Initial convergence to 1.0D-05 achieved. Increase integral accuracy.

SCF Done: E(RB3LYP) = -722.548221925 A.U. after 18 cycles

Convg = 0.5148D-08 -V/T = 2.0031

**********************************************************************

Population analysis using the SCF density.

**********************************************************************

Orbital symmetries:

Occupied (A) (A) (A) (A) (A) (A) (A) (A) (A) (A) (A) (A)

(A) (A) (A) (A) (A) (A) (A) (A) (A) (A) (A) (A)

(A) (A) (A) (A) (A) (A) (A) (A) (A) (A) (A)

Virtual (A) (A) (A) (A) (A) (A) (A) (A) (A) (A) (A) (A)

(A) (A) (A) (A) (A) (A) (A) (A) (A) (A) (A) (A)

(A) (A) (A) (A) (A) (A) (A) (A) (A) (A) (A) (A)

(A) (A) (A) (A) (A) (A) (A) (A) (A) (A) (A) (A)

(A) (A) (A) (A) (A) (A) (A) (A) (A) (A) (A) (A)

(A) (A) (A) (A) (A) (A) (A) (A) (A) (A) (A) (A)

(A) (A) (A) (A) (A) (A) (A) (A) (A) (A) (A) (A)

(A) (A) (A) (A) (A) (A) (A) (A) (A) (A) (A) (A)

(A) (A) (A) (A) (A) (A) (A) (A) (A) (A) (A) (A)

(A) (A) (A) (A) (A) (A) (A) (A) (A) (A) (A) (A)

(A) (A) (A) (A) (A) (A) (A) (A) (A) (A) (A) (A)

(A) (A) (A) (A) (A) (A) (A) (A) (A) (A) (A) (A)

(A) (A) (A) (A) (A) (A) (A) (A) (A) (A) (A) (A)

(A) (A) (A) (A) (A) (A) (A) (A) (A)

The electronic state is 1-A.

Alpha occ. eigenvalues -- -88.89372 -14.31406 -10.22773 -10.22421 -10.22419

Alpha occ. eigenvalues -- -10.21541 -10.21360 -10.20085 -10.20028 -7.98163

Alpha occ. eigenvalues -- -5.94425 -5.94113 -5.93718 -0.97222 -0.83180

Alpha occ. eigenvalues -- -0.81686 -0.75213 -0.71040 -0.61825 -0.59504

Alpha occ. eigenvalues -- -0.57371 -0.51021 -0.48054 -0.46996 -0.44997

Alpha occ. eigenvalues -- -0.42591 -0.41839 -0.39446 -0.37472 -0.34311

Alpha occ. eigenvalues -- -0.32552 -0.29259 -0.28436 -0.21755 -0.20490

Alpha virt. eigenvalues -- -0.09584 -0.05577 -0.01799 -0.00203 0.02913

Alpha virt. eigenvalues -- 0.05265 0.06787 0.07580 0.07965 0.08649

Alpha virt. eigenvalues -- 0.10578 0.11174 0.16755 0.18214 0.18787

Alpha virt. eigenvalues -- 0.23495 0.26551 0.26979 0.28204 0.30812

Alpha virt. eigenvalues -- 0.32167 0.34112 0.35824 0.37622 0.38743

Alpha virt. eigenvalues -- 0.39487 0.42087 0.42464 0.44573 0.45158

Alpha virt. eigenvalues -- 0.46117 0.47992 0.48324 0.49276 0.51578

Alpha virt. eigenvalues -- 0.52951 0.53243 0.56107 0.56588 0.58505

Alpha virt. eigenvalues -- 0.60328 0.61149 0.62391 0.62908 0.64816

Alpha virt. eigenvalues -- 0.66246 0.66490 0.68574 0.69580 0.71085

Alpha virt. eigenvalues -- 0.72872 0.75430 0.77512 0.77819 0.81057

Alpha virt. eigenvalues -- 0.82079 0.85768 0.87600 0.90687 0.92608

Alpha virt. eigenvalues -- 0.93047 1.00376 1.03495 1.06441 1.08537

Alpha virt. eigenvalues -- 1.13207 1.15820 1.19399 1.20264 1.25567

Alpha virt. eigenvalues -- 1.29093 1.30279 1.34099 1.35240 1.39629

Alpha virt. eigenvalues -- 1.40483 1.41840 1.44909 1.50142 1.51600

Alpha virt. eigenvalues -- 1.52072 1.56149 1.57245 1.57868 1.59385

Alpha virt. eigenvalues -- 1.61989 1.64248 1.66861 1.68289 1.71859

Alpha virt. eigenvalues -- 1.72981 1.74665 1.78106 1.80560 1.85457

Alpha virt. eigenvalues -- 1.86719 1.88079 1.89328 1.91552 1.94871

Alpha virt. eigenvalues -- 1.96481 1.99463 2.00056 2.02654 2.05565

Alpha virt. eigenvalues -- 2.08491 2.10002 2.17475 2.19308 2.20806

Alpha virt. eigenvalues -- 2.28999 2.31010 2.36265 2.37088 2.41762

Alpha virt. eigenvalues -- 2.45888 2.52277 2.53161 2.54856 2.55200

Alpha virt. eigenvalues -- 2.57534 2.60011 2.60797 2.63181 2.64732

Alpha virt. eigenvalues -- 2.69262 2.71119 2.75988 2.79965 2.81891

Alpha virt. eigenvalues -- 2.84537 2.85651 2.87012 2.94562 2.97932

Alpha virt. eigenvalues -- 3.03095 3.14754 3.17418 3.25542 3.31163

Alpha virt. eigenvalues -- 3.34252 3.44326 3.47028 3.58839 3.64025

Alpha virt. eigenvalues -- 3.82662 3.84901 3.87646 3.99732 4.38228

Alpha virt. eigenvalues -- 4.40434 4.88755 7.81699 17.17879 17.37325

Alpha virt. eigenvalues -- 17.45944 23.43952 23.69600 23.84175 23.88054

Alpha virt. eigenvalues -- 23.89597 23.99070 23.99330 35.60683 188.93347

Molecular Orbital Coefficients:

31 32 33 34 35

O O O O O

Eigenvalues -- -0.32552 -0.29259 -0.28436 -0.21755 -0.20490

1 1 S 1S 0.00067 0.00070 0.00130 0.00000 -0.00005

2 2S 0.00194 0.00200 0.00371 0.00002 -0.00014

3 3S -0.00454 -0.00434 -0.00822 -0.00023 0.00023

4 4S -0.00625 -0.00796 -0.01394 0.00067 0.00085

5 5S 0.00750 0.02095 0.03256 -0.00405 -0.00167

6 6S 0.02984 0.03570 0.06100 0.01678 -0.00259

7 7PX -0.02192 0.01243 -0.01305 0.00250 -0.00122

8 7PY 0.01139 -0.03086 -0.02298 -0.00677 0.00039

9 7PZ 0.02375 -0.05359 0.04163 -0.03266 0.00531

10 8PX -0.04174 0.02348 -0.02466 0.00467 -0.00224

11 8PY 0.02161 -0.05829 -0.04335 -0.01274 0.00061

12 8PZ 0.04502 -0.10105 0.07849 -0.06153 0.00977

13 9PX 0.06104 -0.03344 0.03577 -0.00669 0.00294

14 9PY -0.03147 0.08415 0.06282 0.01843 0.00017

15 9PZ -0.06494 0.14379 -0.11207 0.08947 -0.01270

16 10PX 0.11980 -0.07689 0.07224 -0.01504 0.00954

17 10PY -0.06297 0.17857 0.13199 0.04137 -0.00703

18 10PZ -0.13220 0.31542 -0.24668 0.19901 -0.03897

19 11PX 0.04054 -0.03993 0.01979 -0.01853 0.00278

20 11PY -0.02389 0.07744 0.05555 0.01946 0.00841

21 11PZ -0.04261 0.13397 -0.10894 0.10409 -0.02136

22 12D 0 0.00721 -0.00484 0.00188 0.00436 0.00056

23 12D+1 -0.01669 -0.01217 0.00508 0.01719 0.02176

24 12D-1 -0.02384 -0.00126 -0.00379 0.00417 0.01914

25 12D+2 0.00297 -0.00298 -0.00735 -0.00479 -0.00803

26 12D-2 0.00292 -0.00604 -0.00573 0.00060 0.00470

27 2 C 1S 0.00046 0.00743 0.01681 0.00040 -0.00391

28 2S 0.00079 0.01192 0.02710 0.00069 -0.00629

29 2PX 0.00963 -0.00060 -0.00323 -0.00325 -0.02155

30 2PY -0.01159 -0.03898 -0.04601 0.00055 0.01668

31 2PZ -0.09843 -0.03167 0.02779 0.00029 0.08298

32 3S -0.00268 -0.03454 -0.08285 -0.00218 0.02003

33 3PX 0.01478 0.00180 0.00059 -0.00322 -0.03151

34 3PY -0.01720 -0.05713 -0.06640 0.00286 0.02401

35 3PZ -0.15481 -0.04352 0.03900 0.00244 0.12842

36 4S -0.01291 -0.00094 -0.02340 0.00043 0.01823

37 4PX 0.02768 -0.03394 -0.06437 -0.00630 -0.03351

38 4PY -0.03475 0.01042 0.03319 0.01237 0.02400

39 4PZ -0.14091 -0.06336 0.01384 0.00350 0.15442

40 5D 0 -0.00065 0.01193 -0.00610 0.00662 0.00032

41 5D+1 0.00678 0.00884 -0.01234 0.00747 0.00060

42 5D-1 0.00588 -0.02634 0.00768 -0.01289 0.00198

43 5D+2 0.00269 -0.02095 -0.03547 -0.00187 -0.00234

44 5D-2 -0.00454 0.00397 -0.00819 0.00308 -0.00326

45 3 N 1S 0.00414 -0.02299 -0.03877 0.00165 0.00307

46 2S 0.00667 -0.03707 -0.06245 0.00265 0.00493

47 2PX 0.00807 0.04936 0.03348 0.02268 -0.00009

48 2PY -0.03714 0.08665 0.19735 0.00230 -0.00755

49 2PZ -0.05058 -0.14108 0.05057 -0.04940 0.04245

50 3S -0.01933 0.10859 0.18147 -0.00889 -0.01391

51 3PX 0.01022 0.07473 0.05083 0.03221 -0.00139

52 3PY -0.05450 0.12003 0.27729 0.00198 -0.00895

53 3PZ -0.07897 -0.20796 0.07749 -0.07124 0.06408

54 4S -0.02896 0.11724 0.21391 -0.01708 -0.02708

55 4PX 0.01498 0.05290 0.03068 0.02640 0.00007

56 4PY -0.04373 0.12922 0.29104 0.00790 -0.01568

57 4PZ -0.05249 -0.20042 0.06997 -0.08306 0.05457

58 5D 0 -0.00636 0.00294 0.01330 -0.00084 0.00395

59 5D+1 -0.01032 0.00065 -0.00205 0.00018 0.01358

60 5D-1 0.00650 0.01487 -0.00344 0.00487 -0.00321

61 5D+2 -0.00331 0.00307 0.00838 -0.00103 -0.00110

62 5D-2 -0.00105 -0.00635 -0.00284 -0.00124 0.00074

63 4 C 1S 0.00643 0.00453 0.01570 0.00745 -0.00729

64 2S 0.01027 0.00719 0.02532 0.01182 -0.01190

65 2PX 0.03003 -0.04596 -0.04063 0.01881 -0.01088

66 2PY -0.00873 -0.02947 -0.07225 -0.01769 0.00065

67 2PZ 0.07978 -0.05058 0.03223 -0.00670 -0.12393

68 3S -0.02851 -0.01845 -0.07537 -0.03502 0.03654

69 3PX 0.04269 -0.07568 -0.06753 0.02164 -0.01807

70 3PY -0.00961 -0.04753 -0.11299 -0.02140 0.00070

71 3PZ 0.12506 -0.07496 0.04677 -0.00829 -0.19282

72 4S -0.03962 -0.09456 -0.14554 -0.11054 0.04461

73 4PX 0.04697 0.00568 -0.02247 0.04995 -0.00888

74 4PY -0.00565 -0.00310 0.01269 -0.03382 0.00843

75 4PZ 0.09096 -0.11013 0.04146 -0.03093 -0.18964

76 5D 0 0.00710 0.00148 0.00246 0.00232 -0.00411

77 5D+1 -0.01019 -0.01199 0.00447 -0.01576 -0.00186

78 5D-1 -0.00852 -0.01540 0.00096 0.01270 0.01063

79 5D+2 -0.00215 -0.00541 -0.02599 -0.00861 0.00217

80 5D-2 0.00023 0.00153 -0.00033 -0.00871 -0.00160

81 5 C 1S -0.00532 -0.00530 -0.01132 0.00317 -0.00333

82 2S -0.00834 -0.00844 -0.01818 0.00516 -0.00507

83 2PX -0.04561 -0.01511 -0.00568 0.02137 -0.00084

84 2PY 0.02345 0.02475 0.06096 0.00064 -0.04852

85 2PZ 0.09006 0.05645 -0.03152 -0.09430 -0.09326

86 3S 0.02351 0.02475 0.05551 -0.01844 0.01322

87 3PX -0.07072 -0.01863 -0.00349 0.02970 -0.00033

88 3PY 0.03694 0.03887 0.08636 -0.00244 -0.06637

89 3PZ 0.13886 0.08905 -0.04755 -0.14196 -0.14222

90 4S 0.01238 0.07840 0.11658 -0.03358 0.06345

91 4PX -0.05367 -0.08035 -0.06319 0.03028 -0.00072

92 4PY 0.03019 0.04079 0.09812 0.02578 -0.08752

93 4PZ 0.11812 0.09564 -0.06760 -0.15335 -0.18375

94 5D 0 -0.00319 0.00550 -0.00173 0.00199 0.00505

95 5D+1 -0.01516 0.00896 -0.00643 0.00584 -0.00982

96 5D-1 -0.00091 -0.00813 0.00349 0.00540 -0.01604

97 5D+2 -0.00470 0.00593 0.00066 -0.00557 0.00525

98 5D-2 0.00249 -0.00113 -0.00430 0.00075 -0.00311

99 6 C 1S -0.00215 -0.00253 0.00534 0.00082 -0.00561

100 2S -0.00343 -0.00408 0.00846 0.00147 -0.00898

101 2PX 0.02923 -0.01429 -0.00362 0.06721 -0.01942

102 2PY 0.04651 -0.02066 0.01958 0.05638 -0.01571

103 2PZ 0.07137 -0.00769 -0.00088 0.10144 0.00789

104 3S 0.00908 0.01264 -0.02780 -0.00851 0.02755

105 3PX 0.04714 -0.02059 -0.00766 0.09688 -0.03111

106 3PY 0.06954 -0.03181 0.02676 0.08270 -0.02395

107 3PZ 0.11009 -0.01249 -0.00457 0.15534 0.01061

108 4S 0.00735 0.05633 -0.02806 0.05003 0.06105

109 4PX 0.03696 0.00910 0.00357 0.14040 -0.01815

110 4PY 0.04976 -0.05211 -0.00059 0.07972 -0.02419

111 4PZ 0.09262 -0.01631 0.01431 0.16878 0.01213

112 5D 0 -0.00128 0.00490 -0.00496 0.00483 0.01268

113 5D+1 0.00769 -0.00750 0.00596 0.00567 -0.01507

114 5D-1 -0.01029 0.00213 0.00159 -0.01167 -0.00201

115 5D+2 -0.00637 -0.00047 -0.00957 -0.02273 -0.00433

116 5D-2 -0.00435 -0.00415 -0.00552 0.00243 0.00277

117 7 C 1S 0.00867 0.00600 -0.00049 -0.00785 0.00092

118 2S 0.01402 0.00962 -0.00089 -0.01265 0.00147

119 2PX 0.03931 0.00997 -0.01181 0.09328 0.07477

120 2PY -0.02151 0.04645 -0.03017 -0.11755 0.01582

121 2PZ 0.03997 0.02396 -0.00804 0.06642 0.04764

122 3S -0.04551 -0.02566 0.00167 0.03353 0.00036

123 3PX 0.05826 0.01614 -0.01656 0.13640 0.10601

124 3PY -0.03211 0.06984 -0.04241 -0.17486 0.02300

125 3PZ 0.05969 0.03694 -0.01335 0.09512 0.07185

126 4S -0.02220 -0.05949 -0.00234 0.09800 -0.03706

127 4PX 0.06710 0.00273 -0.01740 0.15705 0.11188

128 4PY -0.03560 0.06731 -0.04414 -0.18499 0.00833

129 4PZ 0.04415 0.04087 -0.01686 0.10524 0.10682

130 5D 0 -0.01081 0.00053 -0.00115 -0.00925 -0.00571

131 5D+1 0.00748 -0.00310 0.00199 0.01244 0.00149

132 5D-1 0.00256 -0.00434 0.00007 0.01246 -0.00490

133 5D+2 -0.00197 0.00341 -0.00305 -0.00198 -0.00312

134 5D-2 0.00335 -0.00071 0.00031 0.00853 0.00412

135 8 C 1S -0.00508 0.00599 0.00090 -0.00838 0.00177

136 2S -0.00833 0.00966 0.00142 -0.01351 0.00291

137 2PX -0.04960 0.00384 -0.02415 -0.01836 -0.05864

138 2PY -0.01841 -0.05361 0.01745 0.07815 -0.10542

139 2PZ 0.05487 -0.01177 0.02074 0.00660 0.12038

140 3S 0.02775 -0.02726 -0.00647 0.03537 -0.00914

141 3PX -0.07158 0.00638 -0.03425 -0.02579 -0.08617

142 3PY -0.02684 -0.08168 0.02447 0.11757 -0.15440

143 3PZ 0.08315 -0.01832 0.03191 0.01208 0.17529

144 4S 0.00062 -0.04640 0.01427 0.10573 -0.02726

145 4PX -0.07522 0.01115 -0.01771 -0.01540 -0.09993

146 4PY -0.03932 -0.06573 0.02550 0.11263 -0.18463

147 4PZ 0.06786 -0.00895 0.03271 0.02360 0.20439

148 5D 0 0.00642 0.00004 0.00026 0.00359 0.00331

149 5D+1 0.01387 0.00570 -0.00030 -0.01008 0.01416

150 5D-1 -0.00067 -0.00375 0.00036 0.00960 -0.00412

151 5D+2 0.00085 0.00339 -0.00391 -0.00289 0.00177

152 5D-2 0.00561 0.00134 -0.00168 -0.00010 0.00796

153 9 C 1S 0.00057 -0.00078 0.00532 0.00812 0.00108

154 2S 0.00097 -0.00125 0.00856 0.01322 0.00171

155 2PX 0.00440 -0.02396 -0.00046 0.03732 -0.01255

156 2PY 0.02758 0.00741 -0.03289 -0.03593 0.02899

157 2PZ 0.10873 0.03187 0.01029 -0.07913 0.08071

158 3S -0.00339 0.00440 -0.02663 -0.04466 -0.00399

159 3PX 0.00287 -0.03308 -0.00016 0.05267 -0.01531

160 3PY 0.04052 0.00925 -0.04895 -0.05448 0.04039

161 3PZ 0.16511 0.04990 0.01509 -0.11955 0.11989

162 4S 0.02181 -0.04270 -0.05305 -0.02011 -0.03612

163 4PX -0.00991 -0.03848 -0.00064 0.07917 -0.05056

164 4PY 0.04238 -0.00644 -0.05241 -0.04351 0.05058

165 4PZ 0.13836 0.03811 0.01842 -0.13224 0.15660

166 5D 0 -0.00242 0.00063 -0.00157 0.00043 -0.00488

167 5D+1 0.00054 0.00644 -0.00578 -0.01071 -0.01922

168 5D-1 0.01151 0.00394 -0.00187 -0.00742 0.00243

169 5D+2 -0.00142 0.00019 -0.00948 -0.00716 0.01750

170 5D-2 0.00523 0.00623 -0.00116 -0.01019 0.01459

171 10 H 1S -0.00179 -0.03849 -0.05886 -0.00508 -0.00510

172 2S -0.00229 -0.06710 -0.10458 -0.01294 -0.00571

173 3S -0.00273 -0.04100 -0.05905 -0.00453 -0.01515

174 4PX 0.00123 0.00239 0.00407 0.00050 -0.00193

175 4PY -0.00100 -0.00144 -0.00162 0.00017 0.00105

176 4PZ -0.00644 -0.00160 0.00197 0.00030 0.00651

177 11 H 1S 0.03287 -0.01196 0.02227 0.03968 -0.01197

178 2S 0.05575 -0.01538 0.03655 0.06632 -0.01884

179 3S 0.02804 0.00604 0.06566 0.05973 -0.02883

180 4PX 0.00234 -0.00117 0.00176 0.00601 -0.00112

181 4PY -0.00020 -0.00017 0.00067 0.00389 -0.00022

182 4PZ 0.00603 -0.00098 -0.00033 0.00841 0.00026

183 12 H 1S -0.01328 0.01793 -0.00714 -0.03225 -0.00689

184 2S -0.02194 0.02663 -0.00713 -0.05814 -0.01692

185 3S -0.00352 0.01251 -0.00417 -0.04795 -0.01264

186 4PX 0.00344 0.00257 -0.00066 0.00761 0.00687

187 4PY -0.00210 0.00292 -0.00222 -0.00979 0.00054

188 4PZ 0.00237 -0.00005 -0.00073 0.00763 0.00397

189 13 H 1S 0.02225 0.02104 -0.00522 -0.01998 0.02234

190 2S 0.03558 0.03773 -0.00589 -0.03437 0.03719

191 3S 0.01321 0.02363 0.00019 -0.03217 0.03257

192 4PX -0.00303 0.00248 -0.00202 -0.00272 -0.00601

193 4PY -0.00151 -0.00315 0.00133 0.00548 -0.01013

194 4PZ 0.00455 0.00088 0.00182 -0.00104 0.01076

195 14 H 1S -0.00797 -0.00668 0.03162 0.02768 -0.03309

196 2S -0.01321 -0.01048 0.04781 0.04091 -0.05704

197 3S -0.00379 -0.00968 0.03911 0.05402 -0.04520

198 4PX 0.00016 -0.00208 -0.00099 0.00305 -0.00217

199 4PY 0.00130 0.00033 0.00064 -0.00205 0.00219

200 4PZ 0.00838 0.00324 -0.00013 -0.00698 0.00652

36 37 38 39 40

V V V V V

Eigenvalues -- -0.09584 -0.05577 -0.01799 -0.00203 0.02913

1 1 S 1S -0.00062 0.00138 -0.00313 0.00071 0.00191

2 2S -0.00179 0.00398 -0.00902 0.00206 0.00554

3 3S 0.00403 -0.00902 0.02046 -0.00480 -0.01277

4 4S 0.00667 -0.01476 0.03369 -0.00719 -0.01991

5 5S -0.01372 0.03159 -0.07676 0.01391 0.02966

6 6S -0.01841 0.05139 -0.10151 0.04904 0.24377

7 7PX -0.00859 0.01501 -0.05090 0.00142 0.00137

8 7PY -0.00818 0.00282 -0.02375 0.00957 -0.00210

9 7PZ -0.02569 0.00063 0.00416 0.03343 -0.00462

10 8PX -0.01612 0.02795 -0.09376 0.00237 0.00252

11 8PY -0.01511 0.00517 -0.04323 0.01762 -0.00353

12 8PZ -0.04794 0.00111 0.00764 0.06221 -0.00886

13 9PX 0.02311 -0.03962 0.12718 -0.00126 -0.00311

14 9PY 0.02051 -0.00612 0.05338 -0.02389 0.00192

15 9PZ 0.06889 -0.00103 -0.01038 -0.09072 0.01492

16 10PX 0.05871 -0.10457 0.39923 -0.01999 -0.02009

17 10PY 0.06111 -0.02325 0.21014 -0.07634 0.03925

18 10PZ 0.17537 -0.00698 -0.03380 -0.23830 0.02045

19 11PX 0.07976 -0.15733 0.71022 -0.07247 -0.06824

20 11PY 0.06777 -0.05767 0.39909 -0.11370 0.18962

21 11PZ 0.12272 0.01243 -0.02023 -0.37706 0.06874

22 12D 0 0.00700 0.00801 -0.01834 -0.02710 0.01997

23 12D+1 0.01867 0.00783 0.03240 -0.01360 0.00944

24 12D-1 -0.01216 0.00252 0.03340 0.08574 -0.01596

25 12D+2 0.01394 -0.03075 0.13135 -0.03610 0.01071

26 12D-2 0.00381 0.00513 -0.02827 -0.01127 -0.00156

27 2 C 1S 0.00266 -0.00736 0.02751 -0.00634 0.00029

28 2S 0.00438 -0.01183 0.04514 -0.01039 0.00036

29 2PX 0.00603 -0.00569 -0.02265 -0.02442 0.00183

30 2PY -0.00403 -0.00137 0.07193 0.02652 0.01876

31 2PZ -0.05535 0.02482 0.03065 0.17891 -0.02523

32 3S -0.01342 0.03261 -0.12941 0.02980 0.00731

33 3PX 0.00879 -0.00452 -0.02900 -0.03965 0.00054

34 3PY -0.00489 -0.00441 0.12692 0.03740 0.03120

35 3PZ -0.08330 0.03938 0.04464 0.27175 -0.03747

36 4S -0.03720 0.16895 -0.66240 0.12664 -0.09635

37 4PX 0.01949 -0.04409 0.00440 -0.08626 0.02030

38 4PY -0.00096 0.02985 0.14434 0.09805 0.11882

39 4PZ -0.12596 0.04172 0.11140 0.58157 -0.13053

40 5D 0 0.00273 0.00291 0.00182 0.01346 0.00113

41 5D+1 0.00377 0.00291 0.00808 0.01281 -0.00048

42 5D-1 -0.00744 -0.00186 -0.00699 -0.02703 0.00810

43 5D+2 -0.00141 -0.00790 0.00345 -0.00100 -0.00754

44 5D-2 -0.00325 0.00562 -0.01432 0.00721 -0.00167

45 3 N 1S -0.00030 0.00672 0.00045 0.00394 0.00953

46 2S -0.00038 0.01094 0.00103 0.00624 0.01535

47 2PX 0.01693 0.01841 0.05368 0.03253 0.02080

48 2PY -0.00041 0.01097 -0.02152 -0.02558 0.00741

49 2PZ -0.01964 -0.00802 -0.02151 -0.16873 0.04651

50 3S -0.00102 -0.03451 -0.01072 -0.01450 -0.04424

51 3PX 0.02433 0.03071 0.07240 0.04357 0.03210

52 3PY 0.00011 0.01603 -0.02558 -0.03376 0.01197

53 3PZ -0.02718 -0.01171 -0.03108 -0.23118 0.06104

54 4S 0.00765 -0.07515 0.08841 -0.11232 -0.17869

55 4PX 0.01820 -0.00977 0.12623 0.10086 0.01259

56 4PY -0.00414 0.03259 -0.09143 -0.05723 0.03354

57 4PZ -0.03524 -0.02012 -0.04724 -0.44070 0.13501

58 5D 0 -0.00354 0.00110 -0.00116 0.00479 -0.00122

59 5D+1 -0.01067 0.00391 0.00598 0.01480 0.00216

60 5D-1 0.00175 0.00175 0.00035 0.00078 0.00210

61 5D+2 0.00199 0.00197 0.00257 -0.00169 0.00414

62 5D-2 -0.00171 -0.00185 0.00099 0.00194 -0.00158

63 4 C 1S -0.00052 -0.01356 0.00231 -0.00426 -0.00659

64 2S -0.00076 -0.02185 0.00407 -0.00701 -0.01109

65 2PX -0.01307 -0.02200 0.00713 -0.00484 0.00367

66 2PY 0.01779 -0.00112 0.03494 0.00210 0.01339

67 2PZ 0.09560 -0.03698 -0.02236 0.00014 -0.05587

68 3S 0.00731 0.06432 -0.01665 0.02394 0.04308

69 3PX -0.01704 -0.03035 0.00532 -0.00630 0.00173

70 3PY 0.02696 -0.00049 0.05897 -0.00341 0.02305

71 3PZ 0.14388 -0.05232 -0.03137 0.00307 -0.08777

72 4S 0.02924 0.16820 -0.02983 0.01131 -0.20558

73 4PX -0.02998 -0.06703 -0.01720 0.03270 0.16194

74 4PY 0.02181 -0.08856 0.05847 0.12774 0.05466

75 4PZ 0.20968 -0.09647 -0.07902 -0.03274 -0.20011

76 5D 0 0.00107 0.00613 0.00344 0.00769 0.00639

77 5D+1 -0.00770 -0.01194 -0.00341 -0.01826 -0.00552

78 5D-1 0.01195 0.01381 -0.00039 -0.01847 0.01362

79 5D+2 -0.00538 -0.00952 0.00577 0.00006 -0.01442

80 5D-2 -0.00802 -0.00095 -0.02238 0.00174 -0.00819

81 5 C 1S -0.01574 0.01625 -0.03495 0.00355 0.00162

82 2S -0.02543 0.02597 -0.05642 0.00592 0.00306

83 2PX 0.00847 -0.01217 0.13697 -0.03767 0.00270

84 2PY -0.03030 0.00708 -0.04071 0.01596 -0.00123

85 2PZ -0.07392 -0.05217 0.03127 0.06830 -0.03471

86 3S 0.07787 -0.06411 0.13499 -0.01631 -0.02000

87 3PX 0.01774 -0.02296 0.21835 -0.05749 0.00863

88 3PY -0.04041 0.01090 -0.05991 0.02333 -0.00581

89 3PZ -0.10752 -0.07462 0.05068 0.09791 -0.05251

90 4S 0.14260 -0.33842 0.70466 0.02072 0.17299

91 4PX 0.05045 -0.00741 0.47562 -0.16685 -0.15197

92 4PY -0.10148 -0.03530 -0.05623 0.08639 0.03747

93 4PZ -0.16696 -0.14725 0.08279 0.19826 -0.13116

94 5D 0 -0.00219 0.00124 -0.00094 -0.00266 0.00464

95 5D+1 0.00396 -0.00698 -0.00398 0.00080 -0.01026

96 5D-1 0.01475 -0.01437 -0.00185 0.01104 -0.01646

97 5D+2 -0.00597 0.00240 0.00508 -0.00394 0.00884

98 5D-2 0.00112 -0.00207 0.00689 -0.00291 0.00334

99 6 C 1S -0.01025 0.00082 -0.00929 0.00175 0.00776

100 2S -0.01662 0.00127 -0.01477 0.00281 0.01316

101 2PX 0.03838 0.04892 0.02784 0.02960 0.05552

102 2PY -0.00493 0.02852 0.00128 0.01800 0.04535

103 2PZ 0.04024 0.12036 0.00629 0.04825 0.07245

104 3S 0.05599 -0.00697 0.04129 -0.00194 -0.04722

105 3PX 0.05888 0.06831 0.03770 0.05137 0.08880

106 3PY -0.00775 0.04169 0.00314 0.02827 0.06627

107 3PZ 0.05856 0.17508 0.01062 0.07210 0.10030

108 4S 0.05161 -0.01915 0.09467 -0.05733 0.21809

109 4PX 0.03182 0.13473 0.07013 0.02467 0.24042

110 4PY 0.01175 0.08658 0.03266 -0.03951 0.03956

111 4PZ 0.11717 0.28287 0.00596 0.15504 0.21322

112 5D 0 -0.01457 0.00126 0.00662 -0.00407 -0.00883

113 5D+1 0.00742 -0.00976 -0.00381 0.00068 0.00040

114 5D-1 0.00550 0.01477 0.00049 -0.00031 0.00221

115 5D+2 0.02212 0.01822 0.00579 0.00345 0.00452

116 5D-2 -0.00016 -0.00091 0.00591 -0.00271 0.00890

117 7 C 1S -0.00290 0.02933 0.00421 -0.01204 -0.04748

118 2S -0.00465 0.04710 0.00707 -0.01910 -0.07614

119 2PX -0.10626 -0.05636 0.00533 -0.04779 -0.08185

120 2PY 0.03560 0.14275 0.02253 -0.02201 -0.07540

121 2PZ -0.09936 -0.02353 0.00820 -0.04006 -0.05816

122 3S 0.01441 -0.13220 -0.02596 0.04610 0.20869

123 3PX -0.14928 -0.08255 0.00188 -0.06552 -0.10082

124 3PY 0.05085 0.19222 0.02987 -0.03165 -0.10422

125 3PZ -0.14049 -0.03066 0.01083 -0.05650 -0.09093

126 4S 0.00946 -0.34365 0.00091 0.17623 0.80730

127 4PX -0.25508 -0.12555 0.04827 -0.15420 -0.35912

128 4PY 0.05595 0.37587 0.06090 -0.05775 -0.17681

129 4PZ -0.21685 -0.08543 0.00465 -0.08173 -0.04549

130 5D 0 -0.01154 -0.01078 0.00156 -0.00965 -0.00995

131 5D+1 0.00209 0.00748 0.00030 0.00353 0.00960

132 5D-1 -0.00294 0.01897 0.00668 -0.00075 -0.00888

133 5D+2 -0.00163 -0.00793 -0.00059 0.00371 0.00965

134 5D-2 0.00506 0.00312 0.00101 0.00242 -0.00175

135 8 C 1S -0.00286 -0.02997 -0.01645 0.00767 0.04262

136 2S -0.00472 -0.04819 -0.02637 0.01209 0.06837

137 2PX -0.04657 -0.01168 0.02095 -0.00021 0.07644

138 2PY -0.07962 0.13612 0.04163 -0.03271 -0.05333

139 2PZ 0.12474 0.00106 -0.02817 0.00552 -0.08908

140 3S 0.01902 0.13664 0.07619 -0.02705 -0.18748

141 3PX -0.06592 -0.01000 0.02910 0.00047 0.08979

142 3PY -0.11042 0.18662 0.05918 -0.04081 -0.07505

143 3PZ 0.17527 0.00249 -0.03659 0.00537 -0.12376

144 4S -0.03715 0.33864 0.24345 -0.14771 -0.67807

145 4PX -0.15846 -0.07897 0.02311 -0.02031 0.33022

146 4PY -0.17660 0.34569 0.10163 -0.09378 -0.08250

147 4PZ 0.26269 -0.02140 -0.11514 0.04472 -0.21427

148 5D 0 -0.00901 0.00132 0.00427 -0.00328 0.00553

149 5D+1 -0.01002 0.01002 0.00156 -0.00779 0.01063

150 5D-1 -0.00143 -0.01584 -0.00375 0.00669 0.01422

151 5D+2 -0.00305 0.00921 -0.00273 -0.00263 -0.00634

152 5D-2 -0.00809 0.00051 -0.00334 -0.00611 -0.00467

153 9 C 1S -0.00425 -0.00090 -0.00082 -0.00231 -0.01101

154 2S -0.00690 -0.00146 -0.00073 -0.00385 -0.01806

155 2PX 0.00730 -0.00974 -0.00938 0.00496 -0.03619

156 2PY -0.00396 0.02031 -0.03262 -0.00255 0.01462

157 2PZ -0.07053 0.09929 0.03382 -0.04873 0.08646

158 3S 0.02407 0.00724 -0.00637 0.01218 0.05158

159 3PX 0.01221 -0.00804 -0.02349 0.00316 -0.06131

160 3PY -0.00431 0.03161 -0.04529 -0.00181 0.02170

161 3PZ -0.10384 0.14191 0.04802 -0.06287 0.11941

162 4S 0.05729 0.04946 0.21185 -0.00685 -0.00773

163 4PX -0.03482 -0.04201 0.03388 -0.00084 -0.10695

164 4PY -0.00480 0.07662 -0.07460 -0.03760 -0.08437

165 4PZ -0.16585 0.23627 0.10484 -0.17729 0.30821

166 5D 0 -0.00707 -0.00120 0.00183 -0.00151 0.00803

167 5D+1 -0.01884 -0.00367 0.00627 0.00551 0.01071

168 5D-1 0.00112 -0.01362 0.00010 0.00969 -0.00224

169 5D+2 0.01995 -0.01173 0.01370 -0.00039 0.00057

170 5D-2 0.01353 -0.01292 -0.00499 0.00346 0.00442

171 10 H 1S -0.00835 -0.00290 -0.01645 -0.00266 -0.00467

172 2S -0.01321 -0.01606 -0.00633 -0.00904 -0.00459

173 3S -0.03903 -0.00791 -0.03614 -0.04137 -0.09316

174 4PX 0.00071 -0.00113 0.00224 -0.00461 -0.00284

175 4PY -0.00160 0.00168 -0.00129 0.00364 -0.00002

176 4PZ -0.00454 0.00259 0.00549 0.01721 -0.00166

177 11 H 1S -0.02640 -0.03226 -0.01770 0.00397 -0.00036

178 2S -0.03848 -0.07807 -0.04266 0.04822 0.00409

179 3S -0.08898 -0.08139 -0.12903 0.04212 -0.02694

180 4PX 0.00245 0.00301 0.00202 0.00308 0.00796

181 4PY -0.00207 0.00122 -0.00181 -0.00147 0.00291

182 4PZ 0.00456 0.01479 0.00116 0.00525 0.00878

183 12 H 1S -0.01708 0.01612 0.00696 -0.02135 -0.05206

184 2S -0.03241 0.03071 0.01736 -0.05783 -0.13874

185 3S -0.03080 0.09671 0.02065 -0.14800 -0.68753

186 4PX -0.01103 -0.00261 0.00060 -0.00601 -0.00569

187 4PY 0.00306 0.01451 0.00281 -0.00226 -0.00950

188 4PZ -0.01120 -0.00762 0.00094 -0.00573 -0.01381

189 13 H 1S -0.02168 -0.02234 -0.01557 -0.00209 0.04706

190 2S -0.04691 -0.04856 -0.04216 0.00239 0.11305

191 3S -0.05476 -0.11770 -0.15405 0.02875 0.55722

192 4PX -0.00439 -0.00543 0.00184 0.00280 0.00466

193 4PY -0.00720 0.01289 0.00526 -0.00247 -0.00736

194 4PZ 0.01399 -0.00327 -0.00406 0.00249 -0.01489

195 14 H 1S -0.03092 0.02685 -0.00170 -0.00731 -0.00747

196 2S -0.05052 0.07579 -0.02396 -0.01503 -0.03713

197 3S -0.08236 0.07897 -0.15018 -0.02971 -0.16264

198 4PX -0.00130 0.00230 -0.00417 0.00050 -0.00653

199 4PY 0.00249 0.00063 0.00535 -0.00071 0.00331

200 4PZ -0.00771 0.01218 0.00173 -0.00646 0.00999

Density Matrix:

1 2 3 4 5

1 1 S 1S 0.36887

2 2S 0.62174 1.08247

3 3S -0.12709 -0.35658 0.63370

4 4S -0.17295 -0.46365 0.78158 0.99054

5 5S 0.03912 0.11210 -0.25589 -0.46500 0.97706

6 6S 0.03920 0.11211 -0.24067 -0.39751 0.69418

7 7PX -0.00082 -0.00231 0.00515 0.00761 -0.01332

8 7PY 0.00091 0.00255 -0.00552 -0.00928 0.01507

9 7PZ -0.00030 -0.00084 0.00186 0.00291 -0.00477

10 8PX -0.00130 -0.00382 0.00909 0.01353 -0.02475

11 8PY 0.00145 0.00424 -0.00983 -0.01672 0.02792

12 8PZ -0.00047 -0.00139 0.00328 0.00520 -0.00883

13 9PX 0.00188 0.00547 -0.01293 -0.01914 0.03353

14 9PY -0.00220 -0.00640 0.01486 0.02417 -0.03937

15 9PZ 0.00070 0.00203 -0.00476 -0.00740 0.01218

16 10PX 0.00472 0.01365 -0.03078 -0.04575 0.07521

17 10PY -0.00599 -0.01730 0.03818 0.06082 -0.09380

18 10PZ 0.00188 0.00543 -0.01210 -0.01862 0.02873

19 11PX 0.00089 0.00255 -0.00640 -0.01110 0.02425

20 11PY -0.00052 -0.00145 0.00399 0.00797 -0.01868

21 11PZ 0.00028 0.00079 -0.00201 -0.00356 0.00739

22 12D 0 -0.00021 -0.00058 0.00125 0.00311 -0.00851

23 12D+1 0.00007 0.00018 -0.00039 -0.00096 0.00230

24 12D-1 -0.00003 -0.00005 0.00012 0.00061 -0.00269

25 12D+2 0.00011 0.00035 -0.00066 -0.00107 -0.00080

26 12D-2 0.00015 0.00045 -0.00102 -0.00148 0.00202

27 2 C 1S -0.00061 -0.00170 0.00326 0.00622 -0.01077

28 2S -0.00095 -0.00264 0.00512 0.00917 -0.01548

29 2PX -0.00073 -0.00211 0.00475 0.00760 -0.01585

30 2PY -0.00278 -0.00781 0.01645 0.02927 -0.06070

31 2PZ 0.00038 0.00106 -0.00221 -0.00396 0.00811

32 3S 0.00217 0.00601 -0.01150 -0.01953 0.03326

33 3PX -0.00105 -0.00302 0.00684 0.01127 -0.02454

34 3PY -0.00553 -0.01584 0.03286 0.05461 -0.09777

35 3PZ 0.00083 0.00238 -0.00487 -0.00794 0.01326

36 4S -0.00520 -0.01503 0.03225 0.05109 -0.07888

37 4PX -0.00076 -0.00218 0.00518 0.00879 -0.02207

38 4PY 0.00096 0.00277 -0.00648 -0.01133 0.02111

39 4PZ -0.00031 -0.00089 0.00203 0.00363 -0.00761

40 5D 0 -0.00041 -0.00116 0.00238 0.00385 -0.00599

41 5D+1 -0.00008 -0.00024 0.00049 0.00077 -0.00118

42 5D-1 -0.00043 -0.00123 0.00265 0.00425 -0.00720

43 5D+2 -0.00153 -0.00447 0.00951 0.01521 -0.02543

44 5D-2 -0.00014 -0.00041 0.00112 0.00181 -0.00437

45 3 N 1S -0.00013 -0.00037 0.00086 0.00146 -0.00269

46 2S -0.00021 -0.00062 0.00147 0.00240 -0.00460

47 2PX 0.00028 0.00083 -0.00205 -0.00269 0.00453

48 2PY 0.00128 0.00366 -0.00796 -0.01401 0.02937

49 2PZ -0.00022 -0.00063 0.00134 0.00251 -0.00532

50 3S 0.00058 0.00169 -0.00421 -0.00682 0.01493

51 3PX 0.00046 0.00135 -0.00322 -0.00419 0.00654

52 3PY 0.00150 0.00423 -0.00936 -0.01722 0.03934

53 3PZ -0.00025 -0.00069 0.00149 0.00303 -0.00721

54 4S 0.00371 0.01080 -0.02239 -0.03375 0.04754

55 4PX 0.00076 0.00221 -0.00482 -0.00689 0.00993

56 4PY 0.00099 0.00277 -0.00646 -0.01272 0.03399

57 4PZ -0.00013 -0.00037 0.00083 0.00185 -0.00525

58 5D 0 0.00006 0.00016 -0.00040 -0.00079 0.00227

59 5D+1 0.00000 0.00000 0.00002 0.00013 -0.00059

60 5D-1 0.00000 -0.00001 0.00001 -0.00007 0.00046

61 5D+2 -0.00006 -0.00017 0.00039 0.00058 -0.00095

62 5D-2 -0.00011 -0.00033 0.00069 0.00089 -0.00098

63 4 C 1S 0.00028 0.00080 -0.00183 -0.00275 0.00606

64 2S 0.00042 0.00120 -0.00269 -0.00429 0.00973

65 2PX -0.00088 -0.00252 0.00562 0.00934 -0.01968

66 2PY 0.00023 0.00065 -0.00128 -0.00268 0.00707

67 2PZ -0.00016 -0.00046 0.00103 0.00173 -0.00385

68 3S -0.00098 -0.00278 0.00631 0.01101 -0.02720

69 3PX -0.00124 -0.00355 0.00803 0.01358 -0.03002

70 3PY -0.00020 -0.00062 0.00134 0.00099 0.00359

71 3PZ -0.00020 -0.00057 0.00130 0.00221 -0.00519

72 4S -0.00146 -0.00436 0.00865 0.01238 -0.01744

73 4PX -0.00257 -0.00747 0.01460 0.02057 -0.02151

74 4PY 0.00112 0.00327 -0.00622 -0.00915 0.01054

75 4PZ -0.00045 -0.00130 0.00275 0.00426 -0.00729

76 5D 0 0.00007 0.00020 -0.00042 -0.00055 0.00058

77 5D+1 -0.00006 -0.00019 0.00040 0.00055 -0.00072

78 5D-1 -0.00002 -0.00007 0.00012 0.00026 -0.00042

79 5D+2 -0.00022 -0.00066 0.00134 0.00200 -0.00240

80 5D-2 -0.00008 -0.00021 0.00041 0.00083 -0.00157

81 5 C 1S -0.00020 -0.00053 0.00081 0.00231 -0.00358

82 2S -0.00031 -0.00081 0.00121 0.00339 -0.00489

83 2PX 0.00129 0.00360 -0.00734 -0.01468 0.02990

84 2PY -0.00028 -0.00079 0.00154 0.00328 -0.00824

85 2PZ 0.00023 0.00063 -0.00124 -0.00277 0.00628

86 3S 0.00050 0.00126 -0.00131 -0.00557 0.00624

87 3PX 0.00311 0.00879 -0.01776 -0.03235 0.05801

88 3PY -0.00062 -0.00175 0.00345 0.00665 -0.01431

89 3PZ 0.00059 0.00165 -0.00326 -0.00627 0.01193

90 4S -0.00229 -0.00667 0.01511 0.02077 -0.03310

91 4PX -0.00025 -0.00075 0.00347 0.00654 -0.02383

92 4PY 0.00104 0.00316 -0.00597 -0.00609 -0.00392

93 4PZ -0.00037 -0.00111 0.00247 0.00294 -0.00279

94 5D 0 -0.00032 -0.00090 0.00179 0.00302 -0.00477

95 5D+1 0.00035 0.00101 -0.00209 -0.00345 0.00561

96 5D-1 -0.00003 -0.00008 0.00016 0.00025 -0.00025

97 5D+2 0.00101 0.00288 -0.00593 -0.00974 0.01610

98 5D-2 -0.00058 -0.00167 0.00359 0.00587 -0.00973

99 6 C 1S -0.00007 -0.00021 0.00044 0.00080 -0.00188

100 2S -0.00012 -0.00033 0.00071 0.00128 -0.00291

101 2PX 0.00020 0.00056 -0.00126 -0.00213 0.00459

102 2PY -0.00025 -0.00071 0.00154 0.00285 -0.00651

103 2PZ -0.00017 -0.00048 0.00100 0.00202 -0.00508

104 3S 0.00032 0.00090 -0.00193 -0.00343 0.00782

105 3PX 0.00033 0.00096 -0.00209 -0.00330 0.00600

106 3PY -0.00039 -0.00110 0.00237 0.00435 -0.00974

107 3PZ -0.00033 -0.00095 0.00200 0.00373 -0.00844

108 4S -0.00109 -0.00317 0.00580 0.00729 -0.00106

109 4PX -0.00034 -0.00100 0.00179 0.00210 0.00104

110 4PY -0.00029 -0.00082 0.00199 0.00397 -0.01088

111 4PZ 0.00050 0.00148 -0.00296 -0.00343 0.00029

112 5D 0 -0.00001 -0.00002 0.00004 0.00005 0.00006

113 5D+1 -0.00003 -0.00008 0.00018 0.00032 -0.00079

114 5D-1 0.00006 0.00017 -0.00037 -0.00061 0.00112

115 5D+2 0.00001 0.00004 -0.00004 -0.00010 0.00008

116 5D-2 -0.00008 -0.00022 0.00048 0.00070 -0.00095

117 7 C 1S 0.00002 0.00006 -0.00015 -0.00024 0.00042

118 2S 0.00004 0.00011 -0.00024 -0.00038 0.00067

119 2PX 0.00001 0.00002 -0.00008 0.00009 -0.00060

120 2PY 0.00001 0.00002 -0.00003 -0.00022 0.00093

121 2PZ 0.00008 0.00022 -0.00055 -0.00062 0.00075

122 3S -0.00012 -0.00035 0.00080 0.00114 -0.00186

123 3PX 0.00002 0.00006 -0.00017 0.00004 -0.00084

124 3PY 0.00003 0.00009 -0.00016 -0.00049 0.00153

125 3PZ 0.00009 0.00027 -0.00070 -0.00076 0.00102

126 4S -0.00047 -0.00136 0.00311 0.00501 -0.00977

127 4PX 0.00000 -0.00001 0.00006 0.00067 -0.00307

128 4PY -0.00019 -0.00057 0.00112 0.00104 0.00132

129 4PZ -0.00015 -0.00043 0.00060 0.00089 0.00031

130 5D 0 0.00001 0.00003 -0.00007 -0.00016 0.00043

131 5D+1 -0.00003 -0.00008 0.00018 0.00033 -0.00082

132 5D-1 0.00000 -0.00002 0.00003 0.00004 -0.00003

133 5D+2 0.00001 0.00003 -0.00008 -0.00016 0.00048

134 5D-2 0.00001 0.00003 -0.00005 -0.00001 -0.00032

135 8 C 1S -0.00010 -0.00027 0.00056 0.00109 -0.00229

136 2S -0.00015 -0.00044 0.00094 0.00172 -0.00349

137 2PX 0.00043 0.00122 -0.00267 -0.00469 0.00978

138 2PY -0.00013 -0.00038 0.00083 0.00146 -0.00318

139 2PZ 0.00005 0.00015 -0.00033 -0.00050 0.00090

140 3S 0.00052 0.00149 -0.00317 -0.00540 0.01012

141 3PX 0.00057 0.00162 -0.00357 -0.00633 0.01340

142 3PY -0.00015 -0.00043 0.00097 0.00180 -0.00440

143 3PZ 0.00007 0.00021 -0.00046 -0.00065 0.00098

144 4S -0.00101 -0.00290 0.00555 0.00705 -0.00479

145 4PX -0.00018 -0.00055 0.00080 -0.00014 0.00611

146 4PY -0.00027 -0.00080 0.00158 0.00222 -0.00254

147 4PZ -0.00025 -0.00073 0.00137 0.00198 -0.00177

148 5D 0 -0.00002 -0.00004 0.00010 0.00017 -0.00040

149 5D+1 -0.00001 -0.00003 0.00006 0.00010 -0.00017

150 5D-1 -0.00003 -0.00008 0.00017 0.00026 -0.00039

151 5D+2 0.00001 0.00003 -0.00007 -0.00011 0.00029

152 5D-2 -0.00002 -0.00006 0.00012 0.00022 -0.00046

153 9 C 1S 0.00023 0.00067 -0.00149 -0.00245 0.00443

154 2S 0.00036 0.00103 -0.00227 -0.00382 0.00700

155 2PX -0.00087 -0.00250 0.00547 0.00926 -0.01780

156 2PY -0.00009 -0.00026 0.00053 0.00091 -0.00102

157 2PZ -0.00017 -0.00049 0.00109 0.00182 -0.00349

158 3S -0.00104 -0.00297 0.00646 0.01098 -0.01959

159 3PX -0.00083 -0.00237 0.00545 0.00988 -0.02213

160 3PY 0.00016 0.00046 -0.00089 -0.00107 0.00085

161 3PZ -0.00025 -0.00071 0.00158 0.00263 -0.00499

162 4S 0.00056 0.00175 -0.00205 0.00193 -0.02460

163 4PX -0.00114 -0.00318 0.00674 0.01178 -0.02316

164 4PY 0.00086 0.00250 -0.00464 -0.00566 0.00206

165 4PZ -0.00029 -0.00082 0.00171 0.00275 -0.00448

166 5D 0 -0.00001 -0.00003 0.00006 0.00007 0.00011

167 5D+1 0.00004 0.00010 -0.00021 -0.00040 0.00077

168 5D-1 0.00004 0.00010 -0.00019 -0.00031 0.00038

169 5D+2 0.00024 0.00069 -0.00135 -0.00225 0.00341

170 5D-2 0.00005 0.00015 -0.00027 -0.00029 -0.00036

171 10 H 1S -0.00156 -0.00449 0.01002 0.01670 -0.03462

172 2S -0.00209 -0.00595 0.01354 0.02323 -0.05150

173 3S -0.00168 -0.00481 0.01037 0.01681 -0.03180

174 4PX -0.00008 -0.00027 0.00051 0.00058 0.00035

175 4PY -0.00006 -0.00017 0.00041 0.00081 -0.00190

176 4PZ 0.00000 -0.00001 0.00000 -0.00005 0.00036

177 11 H 1S -0.00011 -0.00030 0.00063 0.00129 -0.00317

178 2S -0.00005 -0.00012 0.00024 0.00098 -0.00371

179 3S 0.00020 0.00059 -0.00137 -0.00200 0.00331

180 4PX 0.00002 0.00006 -0.00014 -0.00021 0.00034

181 4PY -0.00001 -0.00003 0.00005 0.00005 0.00006

182 4PZ -0.00004 -0.00012 0.00024 0.00039 -0.00063

183 12 H 1S 0.00005 0.00016 -0.00037 -0.00066 0.00167

184 2S 0.00031 0.00091 -0.00187 -0.00283 0.00432

185 3S 0.00018 0.00052 -0.00108 -0.00173 0.00305

186 4PX 0.00002 0.00006 -0.00013 -0.00015 0.00006

187 4PY 0.00001 0.00002 -0.00004 -0.00008 0.00016

188 4PZ -0.00001 -0.00004 0.00006 0.00009 -0.00002

189 13 H 1S -0.00013 -0.00038 0.00083 0.00148 -0.00304

190 2S -0.00024 -0.00070 0.00148 0.00248 -0.00426

191 3S -0.00022 -0.00063 0.00126 0.00194 -0.00244

192 4PX 0.00002 0.00007 -0.00014 -0.00021 0.00031

193 4PY -0.00002 -0.00006 0.00012 0.00022 -0.00047

194 4PZ -0.00002 -0.00005 0.00011 0.00018 -0.00029

195 14 H 1S -0.00028 -0.00079 0.00168 0.00294 -0.00591

196 2S 0.00017 0.00050 -0.00082 -0.00056 -0.00260

197 3S 0.00026 0.00075 -0.00167 -0.00225 0.00248

198 4PX -0.00002 -0.00005 0.00010 0.00019 -0.00046

199 4PY 0.00007 0.00020 -0.00036 -0.00046 0.00006

200 4PZ -0.00002 -0.00007 0.00014 0.00019 -0.00015

6 7 8 9 10

6 6S 0.64407

7 7PX -0.02522 0.29970

8 7PY 0.03953 -0.00149 0.30190

9 7PZ -0.01276 -0.00091 0.00030 0.30497

10 8PX -0.04791 0.56166 -0.00267 -0.00163 1.05261

11 8PY 0.07559 -0.00265 0.56570 0.00052 -0.00473

12 8PZ -0.02429 -0.00163 0.00052 0.57105 -0.00290

13 9PX 0.08333 0.00627 0.00374 0.00230 0.01006

14 9PY -0.12346 0.00362 0.00012 -0.00065 0.00683

15 9PZ 0.04013 0.00233 -0.00066 -0.00693 0.00424

16 10PX 0.15956 -0.07934 0.00853 0.00621 -0.15154

17 10PY -0.20844 0.00945 -0.08216 -0.00436 0.01783

18 10PZ 0.07361 0.00584 -0.00432 -0.11391 0.01074

19 11PX 0.04509 -0.01492 0.00653 0.00495 -0.02852

20 11PY -0.03287 0.00633 -0.01578 -0.00351 0.01204

21 11PZ 0.01852 0.00408 -0.00348 -0.04075 0.00763

22 12D 0 0.01141 -0.00338 0.00425 0.00064 -0.00646

23 12D+1 -0.00183 0.00250 0.00025 0.00165 0.00481

24 12D-1 0.00643 0.00054 0.00217 -0.00515 0.00103

25 12D+2 0.01113 0.00732 0.00887 0.00039 0.01406

26 12D-2 0.00388 -0.00302 0.00107 0.00038 -0.00580

27 2 C 1S 0.02761 0.00069 0.00834 -0.00125 0.00134

28 2S 0.04249 0.00106 0.01334 -0.00200 0.00207

29 2PX -0.01912 -0.00200 0.00282 0.00106 -0.00385

30 2PY 0.01430 0.00395 0.02703 -0.00634 0.00753

31 2PZ -0.00610 0.00053 -0.00647 -0.01031 0.00103

32 3S -0.11626 -0.00241 -0.03644 0.00535 -0.00479

33 3PX -0.02227 -0.00240 0.00600 0.00165 -0.00464

34 3PY 0.02373 0.00500 0.04297 -0.01055 0.00951

35 3PZ -0.00850 0.00140 -0.01021 -0.01733 0.00268

36 4S -0.15935 -0.00614 -0.03441 0.00414 -0.01186

37 4PX -0.02583 0.00144 0.01111 0.00060 0.00267

38 4PY 0.06362 0.00761 0.01447 -0.00457 0.01460

39 4PZ -0.01611 0.00069 -0.00355 -0.01303 0.00132

40 5D 0 0.00129 0.00035 0.00092 -0.00229 0.00064

41 5D+1 0.00040 -0.00039 0.00062 -0.00081 -0.00077

42 5D-1 -0.00555 -0.00022 0.00222 0.00556 -0.00040

43 5D+2 -0.01566 0.00105 0.00565 -0.00176 0.00200

44 5D-2 -0.01353 0.00408 -0.00145 -0.00031 0.00781

45 3 N 1S -0.01679 0.00093 -0.00039 0.00002 0.00179

46 2S -0.02713 0.00146 -0.00062 0.00002 0.00281

47 2PX 0.02879 0.00187 0.00197 -0.00209 0.00355

48 2PY 0.04515 0.00132 -0.00919 0.00400 0.00260

49 2PZ -0.00802 -0.00241 0.00395 0.01027 -0.00450

50 3S 0.08162 -0.00456 0.00209 -0.00017 -0.00871

51 3PX 0.04058 0.00340 0.00215 -0.00279 0.00647

52 3PY 0.06424 0.00183 -0.01183 0.00541 0.00360

53 3PZ -0.01192 -0.00331 0.00522 0.01450 -0.00617

54 4S 0.09234 -0.00419 -0.00214 0.00186 -0.00797

55 4PX 0.03365 0.00100 0.00291 -0.00357 0.00185

56 4PY 0.06098 -0.00038 -0.01515 0.00635 -0.00062

57 4PZ -0.01064 -0.00439 0.00715 0.02041 -0.00826

58 5D 0 0.00387 -0.00032 -0.00054 -0.00015 -0.00061

59 5D+1 -0.00018 0.00041 0.00009 -0.00101 0.00079

60 5D-1 0.00195 -0.00002 0.00008 -0.00035 -0.00005

61 5D+2 0.00314 0.00052 0.00129 0.00002 0.00099

62 5D-2 -0.00194 -0.00066 0.00033 -0.00023 -0.00125

63 4 C 1S 0.00268 0.00148 -0.00162 0.00071 0.00284

64 2S 0.00392 0.00231 -0.00260 0.00113 0.00442

65 2PX -0.02524 -0.00217 0.00342 -0.00138 -0.00408

66 2PY -0.02059 0.00607 0.00039 0.00154 0.01150

67 2PZ -0.00354 -0.00147 0.00151 0.00605 -0.00276

68 3S -0.00931 -0.00640 0.00775 -0.00316 -0.01227

69 3PX -0.04037 -0.00267 0.00558 -0.00172 -0.00499

70 3PY -0.03276 0.00790 0.00156 0.00167 0.01495

71 3PZ -0.00551 -0.00200 0.00215 0.00863 -0.00374

72 4S 0.00282 -0.01736 0.01969 -0.00505 -0.03309

73 4PX -0.00247 -0.00545 0.00308 -0.00523 -0.01051

74 4PY -0.01392 0.01020 -0.00202 0.00663 0.01941

75 4PZ -0.00873 -0.00456 0.00433 0.01339 -0.00861

76 5D 0 0.00155 -0.00001 0.00011 -0.00024 -0.00002

77 5D+1 -0.00252 0.00022 0.00010 0.00062 0.00044

78 5D-1 -0.00028 -0.00030 0.00053 0.00102 -0.00057

79 5D+2 -0.00516 0.00051 0.00081 -0.00031 0.00095

80 5D-2 0.00301 -0.00258 0.00043 -0.00056 -0.00495

81 5 C 1S 0.01008 -0.00600 0.00005 -0.00140 -0.01146

82 2S 0.01518 -0.00944 0.00008 -0.00222 -0.01813

83 2PX -0.01384 0.02365 0.00114 0.00397 0.04534

84 2PY 0.01888 -0.00698 -0.00093 -0.00223 -0.01334

85 2PZ -0.00651 0.00519 -0.00073 -0.00522 0.00992

86 3S -0.04113 0.02512 -0.00040 0.00645 0.04856

87 3PX -0.01596 0.03836 0.00137 0.00653 0.07354

88 3PY 0.02774 -0.01159 -0.00112 -0.00342 -0.02214

89 3PZ -0.00879 0.00854 -0.00127 -0.00839 0.01632

90 4S -0.06574 0.03495 -0.00365 0.01001 0.06716

91 4PX -0.07189 0.02558 0.00747 0.00600 0.04921

92 4PY 0.01625 0.00088 -0.00469 -0.00127 0.00157

93 4PZ -0.01407 0.00441 -0.00015 -0.01047 0.00844

94 5D 0 0.00056 -0.00089 0.00015 -0.00133 -0.00172

95 5D+1 0.00337 0.00162 -0.00036 -0.00313 0.00309

96 5D-1 -0.00172 0.00023 -0.00044 0.00068 0.00043

97 5D+2 0.00372 0.00367 -0.00121 0.00116 0.00704

98 5D-2 -0.01331 -0.00111 -0.00264 -0.00034 -0.00212

99 6 C 1S 0.00176 -0.00095 0.00008 -0.00039 -0.00182

100 2S 0.00196 -0.00154 0.00016 -0.00061 -0.00293

101 2PX 0.00401 0.00235 -0.00040 0.00029 0.00444

102 2PY -0.00386 -0.00206 0.00025 -0.00161 -0.00386

103 2PZ -0.00002 -0.00182 0.00031 -0.00140 -0.00344

104 3S -0.00453 0.00493 -0.00064 0.00170 0.00935

105 3PX 0.00480 0.00344 -0.00055 0.00029 0.00650

106 3PY -0.00545 -0.00338 0.00056 -0.00203 -0.00636

107 3PZ -0.00161 -0.00252 0.00041 -0.00231 -0.00476

108 4S 0.00303 0.00482 -0.00164 -0.00351 0.00905

109 4PX 0.00385 0.00278 -0.00332 -0.00332 0.00524

110 4PY -0.01410 -0.00235 0.00041 -0.00295 -0.00438

111 4PZ 0.00994 -0.00257 0.00087 -0.00221 -0.00486

112 5D 0 -0.00014 0.00030 -0.00014 -0.00057 0.00057

113 5D+1 -0.00042 -0.00028 0.00011 0.00035 -0.00052

114 5D-1 0.00120 0.00011 -0.00006 0.00024 0.00020

115 5D+2 -0.00272 0.00046 0.00034 0.00083 0.00087

116 5D-2 -0.00312 0.00043 0.00005 -0.00012 0.00081

117 7 C 1S 0.00289 -0.00029 0.00003 0.00019 -0.00057

118 2S 0.00380 -0.00048 0.00006 0.00032 -0.00093

119 2PX 0.00156 0.00046 -0.00083 -0.00322 0.00087

120 2PY -0.00199 -0.00045 0.00068 0.00225 -0.00084

121 2PZ 0.00603 -0.00024 -0.00085 -0.00291 -0.00045

122 3S -0.00858 0.00164 -0.00030 -0.00116 0.00312

123 3PX 0.00209 0.00089 -0.00142 -0.00492 0.00169

124 3PY -0.00233 -0.00086 0.00093 0.00347 -0.00162

125 3PZ 0.00889 -0.00040 -0.00114 -0.00409 -0.00076

126 4S -0.02074 0.00157 -0.00115 -0.00262 0.00303

127 4PX -0.00086 -0.00004 -0.00130 -0.00532 -0.00006

128 4PY -0.00365 0.00003 0.00052 0.00317 0.00005

129 4PZ 0.00892 0.00054 -0.00150 -0.00630 0.00099

130 5D 0 -0.00020 0.00022 -0.00005 -0.00006 0.00042

131 5D+1 -0.00037 -0.00015 0.00001 -0.00026 -0.00028

132 5D-1 0.00005 0.00010 -0.00001 -0.00029 0.00019

133 5D+2 0.00046 0.00017 -0.00009 -0.00006 0.00032

134 5D-2 -0.00027 -0.00005 -0.00003 -0.00013 -0.00010

135 8 C 1S -0.00009 -0.00114 -0.00035 -0.00008 -0.00218

136 2S -0.00037 -0.00180 -0.00056 -0.00014 -0.00344

137 2PX 0.00576 0.00404 0.00066 -0.00075 0.00766

138 2PY -0.00188 -0.00101 -0.00049 -0.00276 -0.00196

139 2PZ 0.00050 0.00035 0.00088 0.00361 0.00070

140 3S 0.00172 0.00516 0.00169 0.00077 0.00987

141 3PX 0.00860 0.00556 0.00098 -0.00116 0.01055

142 3PY -0.00370 -0.00126 -0.00078 -0.00397 -0.00245

143 3PZ 0.00075 0.00035 0.00132 0.00515 0.00071

144 4S -0.00593 0.01206 0.00250 -0.00274 0.02295

145 4PX 0.00436 0.00764 0.00064 -0.00273 0.01449

146 4PY -0.00002 -0.00123 -0.00131 -0.00584 -0.00239

147 4PZ 0.00118 0.00006 0.00068 0.00400 0.00016

148 5D 0 -0.00056 0.00003 -0.00005 -0.00005 0.00007

149 5D+1 -0.00011 -0.00026 0.00005 0.00038 -0.00050

150 5D-1 -0.00054 0.00011 -0.00008 -0.00028 0.00022

151 5D+2 0.00064 -0.00025 -0.00003 -0.00007 -0.00048

152 5D-2 -0.00059 -0.00029 -0.00014 -0.00001 -0.00055

153 9 C 1S 0.00796 0.00162 0.00057 -0.00005 0.00308

154 2S 0.01113 0.00257 0.00088 -0.00009 0.00492

155 2PX -0.01787 -0.00514 -0.00168 -0.00137 -0.00979

156 2PY -0.00090 -0.00211 -0.00013 0.00085 -0.00404

157 2PZ -0.00425 -0.00226 0.00023 0.00309 -0.00429

158 3S -0.02732 -0.00736 -0.00268 0.00053 -0.01408

159 3PX -0.02320 -0.00697 -0.00222 -0.00191 -0.01328

160 3PY -0.00002 -0.00266 0.00057 0.00136 -0.00508

161 3PZ -0.00603 -0.00331 0.00021 0.00428 -0.00627

162 4S -0.04047 -0.00772 -0.00087 0.00246 -0.01470

163 4PX -0.01716 0.00045 -0.00020 -0.00322 0.00074

164 4PY -0.00430 -0.00416 0.00232 0.00225 -0.00786

165 4PZ -0.00494 -0.00250 0.00118 0.00916 -0.00474

166 5D 0 -0.00015 0.00002 -0.00026 -0.00028 0.00004

167 5D+1 -0.00053 0.00065 -0.00008 -0.00076 0.00123

168 5D-1 -0.00066 0.00034 0.00002 0.00007 0.00065

169 5D+2 -0.00289 0.00293 0.00039 0.00097 0.00561

170 5D-2 0.00048 -0.00051 0.00030 0.00026 -0.00095

171 10 H 1S -0.04718 0.00173 0.00519 -0.00072 0.00324

172 2S -0.07400 0.00384 0.00772 -0.00071 0.00728

173 3S -0.02674 0.00337 0.01016 -0.00153 0.00647

174 4PX 0.00386 0.00034 0.00059 0.00008 0.00066

175 4PY 0.00291 0.00067 0.00190 -0.00041 0.00128

176 4PZ 0.00002 0.00005 -0.00039 -0.00077 0.00010

177 11 H 1S -0.00053 -0.00164 -0.00019 -0.00138 -0.00309

178 2S 0.00367 -0.00315 0.00023 -0.00215 -0.00595

179 3S 0.01610 -0.00349 -0.00190 -0.00114 -0.00663

180 4PX 0.00031 0.00016 -0.00010 0.00001 0.00031

181 4PY 0.00077 -0.00007 -0.00004 -0.00001 -0.00015

182 4PZ -0.00028 -0.00014 0.00002 -0.00015 -0.00027

183 12 H 1S 0.00160 0.00011 -0.00009 0.00022 0.00020

184 2S 0.00389 0.00003 -0.00011 0.00105 0.00007

185 3S 0.00243 -0.00028 0.00028 0.00193 -0.00053

186 4PX 0.00049 0.00000 -0.00010 -0.00034 -0.00001

187 4PY -0.00012 -0.00001 0.00008 0.00024 -0.00002

188 4PZ 0.00025 0.00007 -0.00007 -0.00028 0.00013

189 13 H 1S -0.00229 -0.00168 -0.00040 -0.00001 -0.00319

190 2S -0.00138 -0.00349 -0.00099 -0.00025 -0.00664

191 3S -0.00005 -0.00230 -0.00090 0.00021 -0.00438

192 4PX 0.00024 0.00003 -0.00003 -0.00017 0.00006

193 4PY -0.00026 -0.00016 -0.00005 -0.00025 -0.00030

194 4PZ -0.00008 -0.00017 0.00002 0.00027 -0.00032

195 14 H 1S -0.00343 -0.00246 -0.00094 -0.00123 -0.00471

196 2S 0.00058 -0.00492 -0.00109 -0.00205 -0.00936

197 3S 0.01423 -0.00452 0.00054 -0.00288 -0.00863

198 4PX -0.00001 -0.00051 0.00005 -0.00019 -0.00097

199 4PY -0.00114 0.00001 0.00010 0.00010 0.00002

200 4PZ 0.00011 -0.00023 0.00001 0.00014 -0.00044

11 12 13 14 15

11 8PY 1.06002

12 8PZ 0.00088 1.06928

13 9PX 0.00702 0.00419 0.11652

14 9PY -0.00180 -0.00103 -0.00988 0.13460

15 9PZ -0.00104 -0.01399 -0.00515 0.00033 0.14598

16 10PX 0.01597 0.01146 0.19821 -0.02177 -0.01731

17 10PY -0.15689 -0.00794 -0.02429 0.20931 0.01171

18 10PZ -0.00786 -0.21525 -0.01616 0.01136 0.29245

19 11PX 0.01240 0.00928 0.04411 -0.01719 -0.01351

20 11PY -0.03006 -0.00657 -0.01557 0.04611 0.00988

21 11PZ -0.00651 -0.07699 -0.01127 0.00954 0.11379

22 12D 0 0.00818 0.00118 0.00908 -0.01232 -0.00189

23 12D+1 0.00049 0.00312 -0.00700 -0.00079 -0.00461

24 12D-1 0.00419 -0.00981 -0.00164 -0.00649 0.01474

25 12D+2 0.01709 0.00072 -0.02062 -0.02655 -0.00082

26 12D-2 0.00203 0.00071 0.00894 -0.00329 -0.00099

27 2 C 1S 0.01614 -0.00242 -0.00178 -0.02319 0.00350

28 2S 0.02582 -0.00387 -0.00279 -0.03709 0.00559

29 2PX 0.00542 0.00202 0.00422 -0.00898 -0.00290

30 2PY 0.05190 -0.01216 -0.01083 -0.07692 0.01788

31 2PZ -0.01242 -0.01970 -0.00174 0.01816 0.02813

32 3S -0.07057 0.01038 0.00708 0.10280 -0.01504

33 3PX 0.01154 0.00311 0.00557 -0.01789 -0.00449

34 3PY 0.08250 -0.02022 -0.01358 -0.11996 0.02943

35 3PZ -0.01957 -0.03308 -0.00427 0.02842 0.04789

36 4S -0.06628 0.00800 0.00867 0.09983 -0.01375

37 4PX 0.02112 0.00116 0.00035 -0.02939 -0.00106

38 4PY 0.02808 -0.00878 -0.01961 -0.04832 0.01455

39 4PZ -0.00690 -0.02493 -0.00170 0.01162 0.03579

40 5D 0 0.00181 -0.00433 -0.00068 -0.00245 0.00631

41 5D+1 0.00117 -0.00151 0.00115 -0.00150 0.00215

42 5D-1 0.00422 0.01049 0.00067 -0.00588 -0.01529

43 5D+2 0.01076 -0.00334 -0.00245 -0.01432 0.00467

44 5D-2 -0.00278 -0.00055 -0.01148 0.00382 0.00082

45 3 N 1S -0.00081 0.00005 -0.00298 0.00128 -0.00016

46 2S -0.00130 0.00007 -0.00458 0.00210 -0.00022

47 2PX 0.00393 -0.00394 -0.00461 -0.00584 0.00581

48 2PY -0.01711 0.00748 -0.00441 0.02446 -0.01080

49 2PZ 0.00740 0.01916 0.00659 -0.01060 -0.02749

50 3S 0.00444 -0.00044 0.01347 -0.00720 0.00087

51 3PX 0.00439 -0.00525 -0.00875 -0.00717 0.00784

52 3PY -0.02196 0.01010 -0.00642 0.03178 -0.01470

53 3PZ 0.00976 0.02701 0.00891 -0.01415 -0.03862

54 4S -0.00369 0.00342 0.01422 0.00792 -0.00487

55 4PX 0.00569 -0.00673 0.00000 -0.00580 0.00940

56 4PY -0.02832 0.01189 -0.00140 0.04114 -0.01744

57 4PZ 0.01343 0.03825 0.01296 -0.01893 -0.05492

58 5D 0 -0.00101 -0.00029 0.00075 0.00159 0.00035

59 5D+1 0.00018 -0.00192 -0.00111 -0.00025 0.00277

60 5D-1 0.00017 -0.00064 -0.00004 -0.00012 0.00094

61 5D+2 0.00251 0.00002 -0.00161 -0.00335 -0.00010

62 5D-2 0.00061 -0.00044 0.00165 -0.00083 0.00058

63 4 C 1S -0.00309 0.00133 -0.00438 0.00497 -0.00201

64 2S -0.00495 0.00213 -0.00683 0.00787 -0.00324

65 2PX 0.00638 -0.00260 0.00607 -0.00968 0.00395

66 2PY 0.00059 0.00295 -0.01735 0.00023 -0.00467

67 2PZ 0.00287 0.01142 0.00421 -0.00455 -0.01697

68 3S 0.01470 -0.00599 0.01903 -0.02266 0.00924

69 3PX 0.01041 -0.00323 0.00731 -0.01558 0.00468

70 3PY 0.00274 0.00320 -0.02294 -0.00217 -0.00510

71 3PZ 0.00410 0.01628 0.00561 -0.00663 -0.02404

72 4S 0.03742 -0.00971 0.03498 -0.05956 0.01153

73 4PX 0.00595 -0.00990 0.02105 -0.01219 0.01754

74 4PY -0.00394 0.01255 -0.02143 0.01122 -0.01984

75 4PZ 0.00822 0.02528 0.01130 -0.01343 -0.03787

76 5D 0 0.00021 -0.00047 0.00003 -0.00027 0.00063

77 5D+1 0.00017 0.00114 -0.00040 -0.00033 -0.00161

78 5D-1 0.00100 0.00192 0.00070 -0.00121 -0.00266

79 5D+2 0.00150 -0.00057 -0.00144 -0.00196 0.00072

80 5D-2 0.00082 -0.00107 0.00695 -0.00089 0.00143

81 5 C 1S 0.00004 -0.00264 0.01576 0.00030 0.00363

82 2S 0.00008 -0.00422 0.02531 0.00043 0.00586

83 2PX 0.00219 0.00761 -0.06669 -0.00373 -0.01088

84 2PY -0.00165 -0.00425 0.01973 0.00080 0.00621

85 2PZ -0.00137 -0.00984 -0.01431 0.00176 0.01297

86 3S -0.00057 0.01231 -0.06890 -0.00094 -0.01724

87 3PX 0.00266 0.01251 -0.10719 -0.00511 -0.01771

88 3PY -0.00196 -0.00652 0.03135 0.00074 0.00934

89 3PZ -0.00239 -0.01581 -0.02326 0.00315 0.02125

90 4S -0.00661 0.01898 -0.09198 0.01124 -0.02711

91 4PX 0.01409 0.01151 -0.08415 -0.01340 -0.02042

92 4PY -0.00890 -0.00236 0.01111 0.01927 0.00346

93 4PZ -0.00024 -0.01973 -0.01592 -0.00053 0.02704

94 5D 0 0.00031 -0.00253 0.00248 -0.00056 0.00377

95 5D+1 -0.00067 -0.00590 -0.00457 0.00104 0.00873

96 5D-1 -0.00085 0.00130 -0.00059 0.00122 -0.00192

97 5D+2 -0.00233 0.00222 -0.01032 0.00354 -0.00331

98 5D-2 -0.00508 -0.00063 0.00323 0.00753 0.00094

99 6 C 1S 0.00017 -0.00076 0.00270 -0.00035 0.00115

100 2S 0.00030 -0.00118 0.00432 -0.00058 0.00177

101 2PX -0.00073 0.00059 -0.00655 0.00121 -0.00093

102 2PY 0.00047 -0.00302 0.00562 -0.00085 0.00448

103 2PZ 0.00060 -0.00262 0.00498 -0.00093 0.00396

104 3S -0.00122 0.00326 -0.01364 0.00201 -0.00480

105 3PX -0.00103 0.00061 -0.00932 0.00177 -0.00088

106 3PY 0.00105 -0.00382 0.00893 -0.00182 0.00553

107 3PZ 0.00078 -0.00431 0.00675 -0.00127 0.00652

108 4S -0.00304 -0.00657 -0.00742 0.00341 0.01271

109 4PX -0.00624 -0.00617 -0.00803 0.00789 0.01045

110 4PY 0.00073 -0.00557 0.00350 -0.00224 0.00829

111 4PZ 0.00168 -0.00413 0.00689 -0.00102 0.00548

112 5D 0 -0.00027 -0.00107 -0.00080 0.00038 0.00158

113 5D+1 0.00021 0.00067 0.00074 -0.00034 -0.00098

114 5D-1 -0.00010 0.00045 -0.00019 0.00015 -0.00067

115 5D+2 0.00062 0.00157 -0.00131 -0.00084 -0.00236

116 5D-2 0.00008 -0.00022 -0.00125 -0.00005 0.00031

117 7 C 1S 0.00007 0.00035 0.00090 -0.00016 -0.00053

118 2S 0.00012 0.00060 0.00139 -0.00026 -0.00091

119 2PX -0.00159 -0.00607 -0.00132 0.00248 0.00902

120 2PY 0.00127 0.00423 0.00121 -0.00185 -0.00628

121 2PZ -0.00160 -0.00548 0.00059 0.00239 0.00806

122 3S -0.00060 -0.00219 -0.00429 0.00101 0.00338

123 3PX -0.00270 -0.00926 -0.00259 0.00419 0.01384

124 3PY 0.00174 0.00650 0.00219 -0.00257 -0.00964

125 3PZ -0.00214 -0.00771 0.00101 0.00333 0.01137

126 4S -0.00223 -0.00488 -0.00738 0.00200 0.00722

127 4PX -0.00249 -0.01002 -0.00159 0.00363 0.01474

128 4PY 0.00097 0.00594 -0.00003 -0.00192 -0.00842

129 4PZ -0.00283 -0.01190 0.00014 0.00396 0.01777

130 5D 0 -0.00010 -0.00010 -0.00058 0.00013 0.00013

131 5D+1 0.00002 -0.00049 0.00043 0.00000 0.00076

132 5D-1 -0.00003 -0.00053 -0.00029 0.00003 0.00075

133 5D+2 -0.00017 -0.00012 -0.00047 0.00028 0.00016

134 5D-2 -0.00006 -0.00024 0.00010 0.00010 0.00036

135 8 C 1S -0.00064 -0.00016 0.00332 0.00093 0.00020

136 2S -0.00105 -0.00026 0.00525 0.00157 0.00037

137 2PX 0.00126 -0.00137 -0.01170 -0.00201 0.00175

138 2PY -0.00092 -0.00517 0.00306 0.00134 0.00740

139 2PZ 0.00165 0.00676 -0.00124 -0.00235 -0.00948

140 3S 0.00320 0.00147 -0.01515 -0.00506 -0.00224

141 3PX 0.00187 -0.00214 -0.01567 -0.00313 0.00293

142 3PY -0.00149 -0.00741 0.00374 0.00212 0.01051

143 3PZ 0.00248 0.00964 -0.00118 -0.00356 -0.01360

144 4S 0.00484 -0.00516 -0.02470 -0.00669 0.00910

145 4PX 0.00128 -0.00511 -0.01840 -0.00200 0.00804

146 4PY -0.00244 -0.01096 0.00305 0.00273 0.01628

147 4PZ 0.00129 0.00744 0.00027 -0.00169 -0.00918

148 5D 0 -0.00010 -0.00010 -0.00003 0.00010 0.00015

149 5D+1 0.00010 0.00072 0.00079 -0.00010 -0.00106

150 5D-1 -0.00015 -0.00053 -0.00018 0.00023 0.00077

151 5D+2 -0.00006 -0.00013 0.00069 0.00009 0.00019

152 5D-2 -0.00028 -0.00003 0.00084 0.00049 0.00000

153 9 C 1S 0.00113 -0.00009 -0.00466 -0.00198 0.00017

154 2S 0.00175 -0.00016 -0.00742 -0.00302 0.00029

155 2PX -0.00324 -0.00258 0.01512 0.00543 0.00383

156 2PY -0.00031 0.00159 0.00604 0.00091 -0.00241

157 2PZ 0.00041 0.00579 0.00646 -0.00056 -0.00867

158 3S -0.00525 0.00100 0.02107 0.00856 -0.00157

159 3PX -0.00428 -0.00359 0.02037 0.00741 0.00534

160 3PY 0.00100 0.00254 0.00700 -0.00072 -0.00398

161 3PZ 0.00037 0.00799 0.00949 -0.00047 -0.01170

162 4S -0.00204 0.00473 0.01858 0.01127 -0.00992

163 4PX -0.00044 -0.00605 0.00915 0.00470 0.00960

164 4PY 0.00425 0.00426 0.00499 -0.00247 -0.00854

165 4PZ 0.00220 0.01720 0.00987 -0.00297 -0.02559

166 5D 0 -0.00049 -0.00052 -0.00024 0.00058 0.00072

167 5D+1 -0.00016 -0.00143 -0.00171 0.00023 0.00187

168 5D-1 0.00004 0.00013 -0.00093 -0.00007 -0.00025

169 5D+2 0.00073 0.00184 -0.00802 -0.00088 -0.00263

170 5D-2 0.00058 0.00048 0.00148 -0.00072 -0.00068

171 10 H 1S 0.00980 -0.00136 -0.00579 -0.01563 0.00203

172 2S 0.01454 -0.00133 -0.01290 -0.02439 0.00207

173 3S 0.01938 -0.00291 -0.01245 -0.02916 0.00380

174 4PX 0.00114 0.00016 -0.00071 -0.00113 -0.00026

175 4PY 0.00364 -0.00078 -0.00166 -0.00443 0.00098

176 4PZ -0.00075 -0.00147 -0.00012 0.00101 0.00200

177 11 H 1S -0.00034 -0.00260 0.00453 0.00031 0.00384

178 2S 0.00047 -0.00404 0.00887 -0.00053 0.00556

179 3S -0.00352 -0.00216 0.00997 0.00498 0.00301

180 4PX -0.00019 0.00001 -0.00046 0.00029 -0.00007

181 4PY -0.00007 -0.00003 0.00022 0.00007 0.00008

182 4PZ 0.00004 -0.00028 0.00046 -0.00010 0.00044

183 12 H 1S -0.00016 0.00042 -0.00024 0.00021 -0.00067

184 2S -0.00020 0.00197 -0.00064 0.00065 -0.00308

185 3S 0.00053 0.00365 0.00034 -0.00065 -0.00555

186 4PX -0.00018 -0.00064 -0.00001 0.00033 0.00095

187 4PY 0.00015 0.00046 0.00006 -0.00022 -0.00070

188 4PZ -0.00013 -0.00052 -0.00015 0.00018 0.00077

189 13 H 1S -0.00076 -0.00003 0.00483 0.00121 0.00000

190 2S -0.00188 -0.00050 0.00963 0.00274 0.00117

191 3S -0.00172 0.00037 0.00673 0.00261 -0.00020

192 4PX -0.00005 -0.00033 -0.00018 0.00002 0.00050

193 4PY -0.00010 -0.00046 0.00048 0.00015 0.00068

194 4PZ 0.00004 0.00050 0.00048 -0.00010 -0.00067

195 14 H 1S -0.00176 -0.00232 0.00736 0.00243 0.00345

196 2S -0.00203 -0.00385 0.01112 0.00279 0.00505

197 3S 0.00108 -0.00543 0.01156 -0.00066 0.00748

198 4PX 0.00010 -0.00036 0.00131 -0.00016 0.00051

199 4PY 0.00018 0.00020 -0.00023 -0.00016 -0.00034

200 4PZ 0.00001 0.00025 0.00065 -0.00004 -0.00030

16 17 18 19 20

16 10PX 0.36591

17 10PY -0.05212 0.35687

18 10PZ -0.04607 0.04097 0.62164

19 11PX 0.08294 -0.03336 -0.03198 0.02367

20 11PY -0.03116 0.08520 0.02370 -0.01301 0.02867

21 11PZ -0.02742 0.02425 0.24395 -0.01581 0.01097

22 12D 0 0.01651 -0.01935 -0.00504 0.00423 -0.00376

23 12D+1 -0.01203 -0.00141 -0.00975 -0.00235 0.00065

24 12D-1 -0.00324 -0.00918 0.02832 -0.00078 -0.00045

25 12D+2 -0.03654 -0.04002 -0.00309 -0.00480 -0.00495

26 12D-2 0.01558 -0.00676 -0.00248 0.00199 -0.00212

27 2 C 1S -0.00299 -0.03040 0.00411 0.00215 0.00174

28 2S -0.00406 -0.04836 0.00662 0.00338 0.00158

29 2PX 0.00550 -0.01361 -0.00620 -0.00560 -0.00351

30 2PY -0.02034 -0.12445 0.02984 0.00149 -0.02613

31 2PZ -0.00258 0.02993 0.05134 -0.00242 0.00767

32 3S 0.00789 0.13247 -0.01748 -0.00944 -0.00134

33 3PX 0.00681 -0.02662 -0.01006 -0.00800 -0.00529

34 3PY -0.02574 -0.19253 0.04942 0.00316 -0.03945

35 3PZ -0.00712 0.04708 0.08868 -0.00457 0.01233

36 4S 0.01075 0.14634 -0.01883 -0.01011 0.01527

37 4PX -0.00083 -0.05330 -0.00161 -0.00307 -0.01851

38 4PY -0.03543 -0.06868 0.02373 -0.00465 -0.00400

39 4PZ -0.00196 0.01608 0.06352 -0.00136 0.00328

40 5D 0 -0.00188 -0.00227 0.01340 -0.00106 0.00106

41 5D+1 0.00167 -0.00276 0.00571 -0.00016 -0.00082

42 5D-1 0.00239 -0.01116 -0.03311 0.00241 -0.00388

43 5D+2 -0.00419 -0.02558 0.00915 0.00069 -0.00882

44 5D-2 -0.02084 0.00676 0.00297 -0.00498 0.00158

45 3 N 1S -0.00397 -0.00254 0.00115 -0.00020 -0.00550

46 2S -0.00660 -0.00401 0.00180 -0.00067 -0.00909

47 2PX -0.01049 -0.00623 0.01319 -0.00127 0.00341

48 2PY -0.00962 0.06320 -0.02732 -0.00871 0.03711

49 2PZ 0.01712 -0.02635 -0.06889 0.01225 -0.01387

50 3S 0.02142 0.01018 -0.00462 0.00337 0.02732

51 3PX -0.01901 -0.00645 0.01813 -0.00275 0.00594

52 3PY -0.01404 0.08472 -0.03786 -0.01239 0.05146

53 3PZ 0.02377 -0.03606 -0.09789 0.01760 -0.01951

54 4S 0.02343 0.03760 -0.01631 0.00161 0.03589

55 4PX -0.00207 -0.00554 0.02072 -0.00014 0.00366

56 4PY -0.00426 0.09976 -0.04223 -0.00862 0.05372

57 4PZ 0.03116 -0.04444 -0.12973 0.01900 -0.02161

58 5D 0 0.00132 0.00393 -0.00006 -0.00007 0.00233

59 5D+1 -0.00216 -0.00050 0.00535 -0.00064 0.00022

60 5D-1 -0.00057 0.00093 0.00314 -0.00108 0.00134

61 5D+2 -0.00334 -0.00388 -0.00092 -0.00147 0.00091

62 5D-2 0.00345 -0.00132 0.00079 0.00075 -0.00084

63 4 C 1S -0.00799 0.00774 -0.00398 -0.00285 0.00503

64 2S -0.01250 0.01291 -0.00635 -0.00416 0.00798

65 2PX 0.01568 -0.02136 0.00885 0.00849 -0.01270

66 2PY -0.03486 -0.00544 -0.00836 -0.01396 -0.00118

67 2PZ 0.01008 -0.00866 -0.03645 0.00722 -0.00783

68 3S 0.03484 -0.03932 0.01806 0.01042 -0.02279

69 3PX 0.02032 -0.03477 0.01056 0.01237 -0.02064

70 3PY -0.04653 -0.01257 -0.00865 -0.01935 -0.00382

71 3PZ 0.01385 -0.01264 -0.05156 0.01056 -0.01174

72 4S 0.06503 -0.11146 0.01648 0.01733 -0.04419

73 4PX 0.03879 -0.01840 0.03735 0.00825 -0.00498

74 4PY -0.04069 0.01847 -0.04160 -0.01186 0.00902

75 4PZ 0.02494 -0.02713 -0.08131 0.01390 -0.01767

76 5D 0 0.00000 -0.00018 0.00121 -0.00017 0.00033

77 5D+1 -0.00012 -0.00131 -0.00453 0.00124 -0.00146

78 5D-1 0.00145 -0.00292 -0.00610 -0.00004 -0.00082

79 5D+2 -0.00323 -0.00575 0.00212 -0.00092 -0.00334

80 5D-2 0.01247 -0.00138 0.00230 0.00259 -0.00004

81 5 C 1S 0.02709 -0.00150 0.00736 -0.00025 -0.00221

82 2S 0.04285 -0.00241 0.01180 0.00032 -0.00388

83 2PX -0.11989 -0.00414 -0.01998 -0.02711 0.00204

84 2PY 0.03777 0.00718 0.01205 0.01064 -0.00347

85 2PZ -0.02743 0.00452 0.02889 -0.00399 0.00033

86 3S -0.11446 0.00743 -0.03484 -0.00228 0.01234

87 3PX -0.19239 -0.00478 -0.03267 -0.04264 0.00484

88 3PY 0.05952 0.00963 0.01802 0.01608 -0.00507

89 3PZ -0.04441 0.00796 0.04721 -0.00662 0.00104

90 4S -0.16136 0.03653 -0.05737 -0.02270 0.02986

91 4PX -0.14880 -0.02705 -0.03863 -0.03026 -0.01015

92 4PY 0.02180 0.04021 0.00909 0.00853 0.00613

93 4PZ -0.03175 0.00203 0.06053 -0.00447 -0.00271

94 5D 0 0.00388 -0.00032 0.00760 -0.00023 0.00043

95 5D+1 -0.00886 0.00268 0.01873 -0.00273 0.00135

96 5D-1 -0.00111 0.00188 -0.00387 -0.00050 0.00012

97 5D+2 -0.01883 0.00586 -0.00628 -0.00473 0.00313

98 5D-2 0.00592 0.01133 0.00265 0.00126 0.00132

99 6 C 1S 0.00499 -0.00016 0.00186 0.00142 -0.00006

100 2S 0.00827 -0.00044 0.00297 0.00240 -0.00027

101 2PX -0.01432 0.00282 -0.00011 -0.00787 0.00165

102 2PY 0.01399 -0.00152 0.01039 0.00591 -0.00182

103 2PZ 0.01102 -0.00151 0.00977 0.00200 0.00083

104 3S -0.02688 0.00196 -0.00839 -0.00781 0.00123

105 3PX -0.02023 0.00389 0.00094 -0.01085 0.00191

106 3PY 0.02171 -0.00349 0.01310 0.00885 -0.00319

107 3PZ 0.01522 -0.00230 0.01612 0.00262 0.00092

108 4S -0.01655 0.00836 0.03013 -0.00773 0.00760

109 4PX -0.01752 0.01719 0.02657 -0.01120 0.00889

110 4PY 0.01074 -0.00694 0.01843 0.00566 -0.00686

111 4PZ 0.01487 -0.00052 0.01384 0.00141 0.00277

112 5D 0 -0.00160 0.00057 0.00347 -0.00087 0.00073

113 5D+1 0.00181 -0.00061 -0.00197 0.00111 -0.00090

114 5D-1 -0.00064 0.00050 -0.00165 -0.00023 0.00020

115 5D+2 -0.00271 -0.00218 -0.00518 -0.00062 -0.00115

116 5D-2 -0.00227 -0.00069 0.00085 -0.00065 -0.00029

117 7 C 1S 0.00101 0.00019 -0.00138 0.00014 -0.00003

118 2S 0.00200 0.00013 -0.00224 0.00043 -0.00004

119 2PX -0.00255 0.00448 0.01997 -0.00329 0.00482

120 2PY 0.00236 -0.00436 -0.01476 0.00245 -0.00110

121 2PZ 0.00139 0.00501 0.01800 -0.00253 0.00454

122 3S -0.00772 0.00070 0.00756 -0.00214 0.00044

123 3PX -0.00497 0.00772 0.03062 -0.00518 0.00738

124 3PY 0.00433 -0.00598 -0.02264 0.00392 -0.00142

125 3PZ 0.00221 0.00701 0.02543 -0.00372 0.00669

126 4S -0.01293 0.00154 0.01791 -0.00436 -0.00216

127 4PX -0.00224 0.00571 0.03281 -0.00454 0.00599

128 4PY -0.00087 -0.00458 -0.01997 0.00182 -0.00141

129 4PZ 0.00011 0.00845 0.03841 -0.00447 0.00814

130 5D 0 -0.00129 0.00024 0.00025 -0.00032 -0.00013

131 5D+1 0.00108 0.00004 0.00171 0.00031 0.00005

132 5D-1 -0.00063 0.00006 0.00188 -0.00055 -0.00004

133 5D+2 -0.00126 0.00058 0.00046 -0.00070 0.00032

134 5D-2 0.00037 0.00016 0.00084 0.00014 0.00019

135 8 C 1S 0.00601 0.00197 0.00046 0.00189 0.00068

136 2S 0.00943 0.00309 0.00071 0.00300 0.00108

137 2PX -0.02349 -0.00310 0.00516 -0.00973 -0.00201

138 2PY 0.00477 0.00288 0.01828 0.00062 -0.00201

139 2PZ -0.00056 -0.00545 -0.02293 0.00070 0.00018

140 3S -0.02701 -0.00946 -0.00401 -0.00856 -0.00343

141 3PX -0.03168 -0.00471 0.00825 -0.01327 -0.00288

142 3PY 0.00568 0.00420 0.02625 0.00062 -0.00309

143 3PZ 0.00041 -0.00815 -0.03296 0.00150 0.00013

144 4S -0.04545 -0.00819 0.02036 -0.01167 -0.00038

145 4PX -0.03693 -0.00006 0.01891 -0.01282 0.00025

146 4PY 0.00396 0.00704 0.03864 -0.00108 -0.00242

147 4PZ 0.00298 -0.00414 -0.02444 0.00186 0.00331

148 5D 0 0.00005 0.00014 0.00036 0.00016 0.00013

149 5D+1 0.00165 -0.00036 -0.00264 0.00085 0.00016

150 5D-1 -0.00035 0.00041 0.00187 -0.00018 0.00015

151 5D+2 0.00117 0.00001 0.00040 0.00005 0.00026

152 5D-2 0.00157 0.00060 -0.00008 0.00063 0.00058

153 9 C 1S -0.00893 -0.00184 0.00024 -0.00295 0.00049

154 2S -0.01395 -0.00293 0.00049 -0.00435 0.00055

155 2PX 0.02832 0.00720 0.00890 0.01051 0.00220

156 2PY 0.01037 -0.00123 -0.00583 0.00273 0.00230

157 2PZ 0.01352 -0.00217 -0.02102 0.00789 -0.00015

158 3S 0.03862 0.00876 -0.00318 0.01105 -0.00081

159 3PX 0.03828 0.00984 0.01239 0.01453 0.00341

160 3PY 0.01209 -0.00524 -0.00949 0.00374 0.00242

161 3PZ 0.01980 -0.00247 -0.02887 0.01160 0.00005

162 4S 0.03695 0.00678 -0.01749 0.01253 -0.01184

163 4PX 0.01648 0.00675 0.02244 0.00687 -0.00052

164 4PY 0.01015 -0.01059 -0.01825 0.00334 -0.00284

165 4PZ 0.02087 -0.00799 -0.05890 0.01207 -0.00098

166 5D 0 -0.00059 0.00099 0.00170 -0.00049 0.00005

167 5D+1 -0.00345 0.00063 0.00437 -0.00083 -0.00012

168 5D-1 -0.00164 -0.00018 -0.00059 -0.00004 0.00013

169 5D+2 -0.01455 -0.00212 -0.00556 -0.00340 0.00045

170 5D-2 0.00297 -0.00121 -0.00195 0.00161 0.00066

171 10 H 1S -0.01275 -0.03345 0.00446 -0.00723 -0.01588

172 2S -0.02590 -0.05307 0.00494 -0.01079 -0.02576

173 3S -0.02282 -0.05177 0.00707 -0.00531 -0.01683

174 4PX -0.00092 -0.00067 -0.00068 0.00061 0.00083

175 4PY -0.00281 -0.00682 0.00163 0.00021 -0.00100

176 4PZ -0.00016 0.00177 0.00367 -0.00007 0.00057

177 11 H 1S 0.01105 0.00096 0.00893 0.00507 -0.00079

178 2S 0.02012 0.00030 0.01308 0.00812 -0.00097

179 3S 0.01980 0.01368 0.00684 0.00510 0.00624

180 4PX -0.00088 0.00067 -0.00004 -0.00044 0.00036

181 4PY 0.00024 0.00026 0.00012 -0.00022 0.00021

182 4PZ 0.00103 -0.00020 0.00108 0.00024 -0.00004

183 12 H 1S -0.00052 0.00041 -0.00148 -0.00044 0.00042

184 2S -0.00139 0.00125 -0.00677 -0.00063 0.00068

185 3S 0.00039 -0.00150 -0.01205 0.00053 -0.00084

186 4PX 0.00006 0.00066 0.00208 -0.00025 0.00060

187 4PY 0.00008 -0.00048 -0.00160 0.00018 -0.00017

188 4PZ -0.00039 0.00037 0.00174 -0.00037 0.00031

189 13 H 1S 0.00967 0.00189 -0.00051 0.00416 0.00148

190 2S 0.01856 0.00475 0.00139 0.00688 0.00317

191 3S 0.01252 0.00448 -0.00164 0.00429 0.00261

192 4PX -0.00040 0.00015 0.00118 -0.00019 -0.00003

193 4PY 0.00084 0.00036 0.00169 0.00026 -0.00015

194 4PZ 0.00110 -0.00022 -0.00172 0.00051 0.00014

195 14 H 1S 0.01418 0.00543 0.00789 0.00445 -0.00177

196 2S 0.02150 0.00685 0.01171 0.00628 -0.00259

197 3S 0.02159 0.00184 0.01633 0.00499 -0.00088

198 4PX 0.00240 -0.00040 0.00113 0.00069 -0.00008

199 4PY -0.00024 -0.00040 -0.00076 0.00030 -0.00018

200 4PZ 0.00125 -0.00015 -0.00089 0.00056 0.00006

21 22 23 24 25

21 11PZ 0.10084

22 12D 0 -0.00183 0.00280

23 12D+1 -0.00270 -0.00009 0.00331

24 12D-1 0.00791 -0.00012 0.00085 0.00493

25 12D+2 -0.00129 0.00091 0.00109 0.00121 0.01168

26 12D-2 -0.00116 0.00059 -0.00043 0.00014 -0.00094

27 2 C 1S -0.00038 0.00393 0.00023 0.00248 0.00828

28 2S -0.00042 0.00596 0.00034 0.00381 0.01279

29 2PX -0.00219 -0.00160 -0.00214 -0.00009 0.00595

30 2PY 0.00711 0.00666 0.00170 0.00874 0.01819

31 2PZ 0.00745 -0.00548 0.00430 0.01559 -0.00625

32 3S 0.00124 -0.01530 -0.00081 -0.01016 -0.03457

33 3PX -0.00366 -0.00152 -0.00295 0.00004 0.01010

34 3PY 0.01192 0.01082 0.00229 0.01392 0.02840

35 3PZ 0.01515 -0.00863 0.00657 0.02454 -0.00963

36 4S -0.00202 -0.01050 -0.00150 -0.00707 -0.02928

37 4PX 0.00137 -0.00109 -0.00237 -0.00097 0.00982

38 4PY 0.00468 0.00371 0.00373 0.00822 0.01645

39 4PZ 0.00841 -0.00575 0.00870 0.02272 -0.00754

40 5D 0 0.00589 0.00071 -0.00012 0.00035 0.00085

41 5D+1 0.00439 0.00066 0.00006 -0.00124 0.00003

42 5D-1 -0.01374 0.00054 0.00055 -0.00071 0.00126

43 5D+2 0.00343 0.00062 -0.00010 0.00121 0.00303

44 5D-2 0.00249 -0.00140 0.00068 -0.00042 0.00174

45 3 N 1S 0.00180 -0.00102 0.00026 -0.00032 -0.00069

46 2S 0.00286 -0.00170 0.00045 -0.00060 -0.00127

47 2PX 0.00810 0.00339 0.00072 -0.00167 0.00151

48 2PY -0.01695 -0.00092 0.00044 0.00309 0.00462

49 2PZ -0.04319 -0.00225 0.00309 0.01083 -0.00277

50 3S -0.00826 0.00545 -0.00158 0.00216 0.00428

51 3PX 0.01160 0.00476 0.00113 -0.00265 0.00242

52 3PY -0.02411 -0.00107 0.00066 0.00490 0.00709

53 3PZ -0.06283 -0.00365 0.00468 0.01668 -0.00429

54 4S -0.01386 0.00270 -0.00204 0.00185 0.00439

55 4PX 0.01105 0.00325 0.00018 -0.00143 0.00147

56 4PY -0.02411 -0.00045 0.00047 0.00245 0.00329

57 4PZ -0.07091 -0.00201 0.00412 0.01130 -0.00259

58 5D 0 -0.00113 -0.00008 0.00016 0.00095 -0.00013

59 5D+1 0.00167 -0.00011 0.00084 0.00123 -0.00018

60 5D-1 0.00321 0.00053 -0.00018 -0.00142 0.00065

61 5D+2 -0.00111 0.00016 -0.00006 0.00070 0.00175

62 5D-2 -0.00026 -0.00032 -0.00004 0.00061 0.00024

63 4 C 1S -0.00175 -0.00016 -0.00028 -0.00026 0.00001

64 2S -0.00277 -0.00027 -0.00047 -0.00043 0.00001

65 2PX 0.00535 -0.00105 0.00055 -0.00009 0.00193

66 2PY -0.00398 -0.00185 -0.00138 -0.00001 -0.00063

67 2PZ -0.01887 -0.00122 -0.00798 -0.00408 0.00280

68 3S 0.00802 0.00096 0.00135 0.00109 -0.00006

69 3PX 0.00645 -0.00184 0.00069 0.00000 0.00300

70 3PY -0.00413 -0.00222 -0.00210 0.00004 -0.00118

71 3PZ -0.02714 -0.00204 -0.01251 -0.00626 0.00444

72 4S -0.00343 0.00169 -0.00319 0.00860 0.00210

73 4PX 0.01865 0.00215 -0.00117 -0.00049 0.00167

74 4PY -0.01871 -0.00284 0.00001 -0.00047 0.00184

75 4PZ -0.03912 -0.00107 -0.01090 -0.00642 0.00383

76 5D 0 0.00016 0.00002 -0.00052 0.00002 0.00025

77 5D+1 -0.00390 -0.00066 -0.00019 0.00116 -0.00004

78 5D-1 -0.00242 0.00036 0.00149 0.00083 -0.00066

79 5D+2 0.00129 0.00013 -0.00032 -0.00045 -0.00086

80 5D-2 -0.00026 0.00039 -0.00101 0.00041 -0.00093

81 5 C 1S 0.00195 0.00253 -0.00188 -0.00010 -0.00638

82 2S 0.00330 0.00398 -0.00296 -0.00018 -0.01017

83 2PX -0.00363 -0.00483 0.00707 -0.00007 0.01338

84 2PY 0.00400 0.00154 -0.00306 -0.00195 -0.00129

85 2PZ 0.00648 -0.00408 -0.01332 -0.00393 0.00565

86 3S -0.01093 -0.01071 0.00777 0.00064 0.02812

87 3PX -0.00644 -0.00832 0.01124 -0.00001 0.02225

88 3PY 0.00578 0.00258 -0.00484 -0.00281 -0.00248

89 3PZ 0.01120 -0.00646 -0.02030 -0.00591 0.00899

90 4S -0.02165 -0.01040 0.00905 0.00220 0.02989

91 4PX -0.00894 -0.00569 0.00985 0.00002 0.01676

92 4PY 0.00560 0.00075 -0.00297 -0.00531 -0.00263

93 4PZ 0.01764 -0.00535 -0.02218 -0.00739 0.00858

94 5D 0 0.00280 0.00053 -0.00012 0.00061 -0.00065

95 5D+1 0.00741 -0.00057 0.00001 0.00087 0.00060

96 5D-1 -0.00171 -0.00015 -0.00037 -0.00035 -0.00018

97 5D+2 -0.00220 -0.00112 0.00069 -0.00031 0.00120

98 5D-2 0.00132 -0.00060 -0.00052 -0.00047 -0.00245

99 6 C 1S 0.00034 -0.00006 -0.00009 0.00019 0.00019

100 2S 0.00062 -0.00006 -0.00016 0.00029 0.00029

101 2PX 0.00291 0.00082 0.00016 -0.00184 -0.00063

102 2PY 0.00690 0.00035 0.00081 -0.00050 0.00072

103 2PZ 0.00839 0.00106 0.00199 -0.00096 0.00047

104 3S -0.00210 -0.00011 0.00045 -0.00073 -0.00073

105 3PX 0.00468 0.00107 -0.00006 -0.00285 -0.00086

106 3PY 0.00937 0.00069 0.00110 -0.00099 0.00092

107 3PZ 0.01346 0.00153 0.00294 -0.00160 0.00068

108 4S 0.01951 0.00249 0.00397 -0.00105 -0.00122

109 4PX 0.01936 0.00181 0.00270 -0.00310 -0.00086

110 4PY 0.00982 -0.00060 0.00109 0.00046 0.00013

111 4PZ 0.01296 0.00225 0.00427 -0.00123 0.00022

112 5D 0 0.00190 0.00011 0.00073 0.00032 -0.00021

113 5D+1 -0.00081 -0.00007 -0.00054 -0.00048 0.00031

114 5D-1 -0.00127 -0.00006 -0.00021 0.00014 -0.00005

115 5D+2 -0.00315 -0.00040 -0.00090 -0.00009 0.00018

116 5D-2 0.00068 -0.00013 0.00044 0.00015 0.00003

117 7 C 1S -0.00107 0.00004 -0.00059 -0.00024 -0.00001

118 2S -0.00163 0.00008 -0.00097 -0.00039 -0.00002

119 2PX 0.01264 0.00103 0.00508 0.00147 -0.00113

120 2PY -0.01053 -0.00090 -0.00306 0.00028 0.00065

121 2PZ 0.01082 0.00126 0.00285 0.00050 -0.00123

122 3S 0.00457 -0.00038 0.00310 0.00156 0.00003

123 3PX 0.01891 0.00137 0.00719 0.00215 -0.00157

124 3PY -0.01605 -0.00134 -0.00454 0.00045 0.00096

125 3PZ 0.01549 0.00190 0.00411 0.00065 -0.00179

126 4S 0.01233 -0.00072 0.00339 -0.00022 0.00027

127 4PX 0.02093 0.00151 0.00811 0.00225 -0.00201

128 4PY -0.01552 -0.00163 -0.00594 0.00000 0.00102

129 4PZ 0.02095 0.00193 0.00633 0.00269 -0.00235

130 5D 0 -0.00027 -0.00012 -0.00023 0.00005 -0.00002

131 5D+1 0.00118 0.00006 0.00033 0.00002 0.00006

132 5D-1 0.00135 0.00012 0.00016 -0.00020 -0.00005

133 5D+2 0.00015 0.00001 -0.00026 -0.00017 -0.00008

134 5D-2 0.00073 0.00005 0.00046 0.00010 0.00000

135 8 C 1S -0.00022 -0.00007 -0.00027 0.00009 -0.00051

136 2S -0.00036 -0.00010 -0.00040 0.00015 -0.00081

137 2PX 0.00193 0.00029 -0.00149 -0.00126 0.00089

138 2PY 0.01062 0.00035 -0.00089 -0.00224 -0.00023

139 2PZ -0.01029 0.00013 0.00343 0.00272 -0.00033

140 3S 0.00022 0.00019 0.00081 -0.00073 0.00244

141 3PX 0.00313 0.00048 -0.00220 -0.00181 0.00129

142 3PY 0.01562 0.00046 -0.00111 -0.00338 -0.00034

143 3PZ -0.01476 0.00019 0.00496 0.00395 -0.00045

144 4S 0.01272 0.00007 0.00440 0.00103 0.00582

145 4PX 0.00756 -0.00019 -0.00214 -0.00129 0.00330

146 4PY 0.02015 0.00039 -0.00248 -0.00355 0.00008

147 4PZ -0.01128 0.00038 0.00717 0.00593 -0.00120

148 5D 0 0.00034 -0.00005 0.00005 -0.00005 0.00008

149 5D+1 -0.00162 -0.00011 -0.00042 0.00008 0.00003

150 5D-1 0.00121 0.00003 0.00026 -0.00005 0.00000

151 5D+2 0.00004 0.00010 -0.00015 0.00002 -0.00021

152 5D-2 -0.00007 -0.00006 0.00007 0.00014 -0.00016

153 9 C 1S 0.00039 0.00005 0.00071 0.00008 0.00125

154 2S 0.00073 0.00007 0.00116 0.00013 0.00201

155 2PX 0.00509 -0.00046 0.00047 0.00022 -0.00215

156 2PY -0.00458 -0.00003 -0.00197 0.00039 -0.00125

157 2PZ -0.01286 -0.00141 -0.00472 -0.00034 0.00081

158 3S -0.00335 -0.00025 -0.00363 -0.00039 -0.00598

159 3PX 0.00711 -0.00074 0.00099 0.00050 -0.00272

160 3PY -0.00711 -0.00016 -0.00287 0.00056 -0.00090

161 3PZ -0.01849 -0.00222 -0.00740 -0.00041 0.00120

162 4S -0.00518 -0.00023 -0.00307 -0.00465 -0.00923

163 4PX 0.01310 0.00069 0.00155 -0.00087 -0.00241

164 4PY -0.00887 0.00003 -0.00138 -0.00023 -0.00176

165 4PZ -0.03006 -0.00115 -0.00395 -0.00002 0.00055

166 5D 0 0.00076 -0.00003 -0.00015 -0.00013 -0.00019

167 5D+1 0.00139 -0.00035 -0.00145 -0.00060 0.00054

168 5D-1 -0.00065 -0.00029 -0.00072 -0.00016 0.00044

169 5D+2 -0.00222 -0.00060 0.00095 0.00045 0.00136

170 5D-2 -0.00138 -0.00009 0.00003 0.00048 0.00034

171 10 H 1S 0.00193 -0.00293 -0.00063 0.00165 0.00512

172 2S 0.00239 -0.00451 -0.00064 0.00226 0.00766

173 3S 0.00254 -0.00044 -0.00008 0.00213 0.00905

174 4PX -0.00020 0.00048 0.00009 -0.00014 0.00058

175 4PY 0.00042 0.00051 0.00025 0.00047 0.00122

176 4PZ 0.00062 -0.00028 0.00034 0.00106 -0.00040

177 11 H 1S 0.00570 0.00043 0.00057 -0.00045 0.00021

178 2S 0.00886 0.00117 0.00073 -0.00112 0.00035

179 3S 0.00494 0.00131 0.00032 -0.00118 -0.00002

180 4PX 0.00019 0.00003 0.00010 -0.00006 0.00001

181 4PY 0.00011 0.00007 0.00009 0.00000 -0.00006

182 4PZ 0.00086 0.00010 0.00016 -0.00011 0.00001

183 12 H 1S -0.00162 0.00000 -0.00116 -0.00022 -0.00012

184 2S -0.00493 -0.00013 -0.00232 -0.00055 -0.00012

185 3S -0.00674 -0.00012 -0.00233 -0.00097 0.00005

186 4PX 0.00122 0.00011 0.00044 0.00016 -0.00012

187 4PY -0.00104 -0.00008 -0.00028 0.00000 0.00007

188 4PZ 0.00109 0.00010 0.00034 0.00005 -0.00011

189 13 H 1S -0.00081 -0.00030 -0.00059 0.00018 -0.00017

190 2S -0.00086 -0.00037 -0.00138 0.00042 -0.00077

191 3S -0.00246 -0.00024 -0.00081 0.00081 -0.00111

192 4PX 0.00046 -0.00001 -0.00024 -0.00015 0.00007

193 4PY 0.00097 0.00002 -0.00015 -0.00025 0.00001

194 4PZ -0.00083 0.00000 0.00028 0.00027 -0.00005

195 14 H 1S 0.00455 0.00022 -0.00020 -0.00087 -0.00124

196 2S 0.00669 0.00037 -0.00064 -0.00151 -0.00151

197 3S 0.00846 0.00147 -0.00002 -0.00079 -0.00103

198 4PX 0.00054 0.00006 -0.00003 0.00002 -0.00013

199 4PY -0.00036 -0.00015 -0.00003 0.00004 0.00018

200 4PZ -0.00079 -0.00009 -0.00044 0.00002 0.00001

26 27 28 29 30

26 12D-2 0.00361

27 2 C 1S -0.00132 0.65899

28 2S -0.00190 0.56135 0.49150

29 2PX 0.01345 0.00147 0.00204 0.09369

30 2PY 0.00040 -0.00268 -0.00276 -0.00166 0.08339

31 2PZ 0.00072 0.00055 0.00057 0.00343 -0.00233

32 3S 0.00426 -0.08673 -0.15108 -0.00942 0.00432

33 3PX 0.01928 0.00177 0.00323 0.13397 0.00274

34 3PY 0.00132 0.00728 0.00952 0.00373 0.12367

35 3PZ 0.00065 -0.00131 -0.00163 0.00238 -0.00161

36 4S 0.00481 -0.06351 -0.09754 0.01032 -0.02428

37 4PX 0.01274 -0.00088 -0.00286 0.08489 0.01378

38 4PY -0.00166 0.01614 0.02272 0.00277 0.04394

39 4PZ 0.00127 -0.00355 -0.00535 -0.00373 0.01262

40 5D 0 0.00009 0.00123 0.00341 0.00039 0.00043

41 5D+1 0.00040 -0.00011 -0.00082 0.00027 0.00083

42 5D-1 0.00023 0.00079 0.00176 0.00161 0.00126

43 5D+2 0.00035 0.00218 0.00282 0.00315 0.01049

44 5D-2 -0.00064 -0.00024 0.00043 0.00498 -0.00366

45 3 N 1S 0.00088 0.00446 0.00678 0.01218 -0.01109

46 2S 0.00121 0.00704 0.01027 0.01956 -0.01766

47 2PX -0.00592 -0.01685 -0.02627 -0.03478 0.03782

48 2PY 0.00324 0.01872 0.02986 0.04413 -0.02199

49 2PZ -0.00268 -0.00558 -0.00878 -0.02209 0.02063

50 3S -0.00265 -0.01645 -0.02332 -0.05604 0.04924

51 3PX -0.00889 -0.02785 -0.04085 -0.05071 0.05420

52 3PY 0.00483 0.02991 0.04521 0.06379 -0.02831

53 3PZ -0.00411 -0.00926 -0.01371 -0.03292 0.03009

54 4S 0.00112 -0.01302 -0.01006 -0.01438 0.01504

55 4PX -0.00429 -0.01301 -0.01874 -0.02296 0.02844

56 4PY 0.00087 0.02470 0.03637 0.03647 -0.02789

57 4PZ -0.00171 -0.00530 -0.00769 -0.01936 0.02140

58 5D 0 0.00024 0.00292 0.00368 0.00220 -0.00145

59 5D+1 -0.00002 -0.00163 -0.00205 -0.00169 0.00277

60 5D-1 0.00071 0.00201 0.00267 0.00473 -0.00245

61 5D+2 0.00113 0.00127 0.00211 0.00771 0.00287

62 5D-2 0.00096 0.00537 0.00715 0.00757 -0.00507

63 4 C 1S 0.00026 -0.00137 -0.00214 -0.00386 0.00016

64 2S 0.00027 -0.00216 -0.00338 -0.00624 0.00021

65 2PX 0.00054 0.00364 0.00574 0.00915 -0.00503

66 2PY 0.00573 -0.00101 -0.00184 0.00307 -0.00784

67 2PZ -0.00301 0.00043 0.00075 0.00111 -0.00060

68 3S -0.00018 0.00600 0.00968 0.01898 -0.00086

69 3PX 0.00045 0.00442 0.00704 0.01135 -0.00611

70 3PY 0.00841 -0.00144 -0.00260 0.00373 -0.00984

71 3PZ -0.00462 0.00066 0.00112 0.00200 -0.00109

72 4S 0.01159 0.01265 0.01511 0.05293 0.02067

73 4PX 0.00581 0.01684 0.02150 0.03223 -0.00717

74 4PY 0.00164 -0.00191 -0.00116 0.00509 -0.01004

75 4PZ -0.00241 0.00183 0.00225 0.00589 -0.00070

76 5D 0 0.00009 0.00006 0.00023 0.00131 0.00044

77 5D+1 -0.00068 -0.00017 -0.00034 -0.00217 0.00126

78 5D-1 0.00054 -0.00035 -0.00053 -0.00133 0.00128

79 5D+2 -0.00025 -0.00240 -0.00391 -0.00727 0.00235

80 5D-2 0.00126 0.00101 0.00171 0.00127 -0.00145

81 5 C 1S 0.00179 -0.00134 -0.00216 -0.00349 -0.00179

82 2S 0.00276 -0.00217 -0.00347 -0.00568 -0.00284

83 2PX -0.00573 0.00136 0.00216 0.00449 0.00570

84 2PY -0.00666 0.00341 0.00555 0.00538 -0.00131

85 2PZ -0.00230 0.00040 0.00061 0.00439 -0.00363

86 3S -0.00702 0.00681 0.01072 0.01766 0.00783

87 3PX -0.00876 0.00247 0.00394 0.00964 0.00767

88 3PY -0.00908 0.00434 0.00727 0.00778 -0.00128

89 3PZ -0.00370 0.00075 0.00116 0.00721 -0.00578

90 4S -0.00634 -0.00052 0.00423 0.03281 0.01505

91 4PX -0.00673 0.00059 0.00018 0.00099 0.01450

92 4PY -0.01305 0.00266 0.00542 -0.02027 -0.01260

93 4PZ -0.00275 0.00173 0.00227 0.01346 -0.00517

94 5D 0 0.00053 0.00049 0.00075 0.00113 0.00106

95 5D+1 -0.00048 0.00008 0.00014 -0.00018 0.00073

96 5D-1 -0.00006 -0.00009 -0.00019 -0.00014 -0.00109

97 5D+2 -0.00048 -0.00047 -0.00085 -0.00092 -0.00248

98 5D-2 0.00013 -0.00139 -0.00229 -0.00272 -0.00542

99 6 C 1S 0.00004 0.00099 0.00160 0.00207 -0.00093

100 2S 0.00008 0.00160 0.00258 0.00337 -0.00148

101 2PX 0.00036 -0.00204 -0.00333 -0.00232 0.00089

102 2PY -0.00091 0.00088 0.00140 0.00225 0.00004

103 2PZ 0.00097 0.00175 0.00281 0.00536 -0.00251

104 3S -0.00036 -0.00507 -0.00804 -0.01062 0.00435

105 3PX 0.00020 -0.00329 -0.00522 -0.00447 0.00115

106 3PY -0.00120 0.00110 0.00174 0.00311 0.00020

107 3PZ 0.00133 0.00239 0.00378 0.00757 -0.00373

108 4S 0.00027 0.00277 0.00120 -0.01101 0.00305

109 4PX 0.00090 -0.00082 -0.00170 -0.00067 -0.00293

110 4PY -0.00123 -0.00347 -0.00439 0.00022 0.00262

111 4PZ 0.00120 0.00098 0.00241 0.00585 -0.00179

112 5D 0 0.00022 -0.00018 -0.00032 -0.00058 0.00026

113 5D+1 -0.00049 -0.00006 -0.00001 0.00001 0.00000

114 5D-1 -0.00004 0.00005 0.00007 -0.00002 0.00011

115 5D+2 0.00037 -0.00013 -0.00015 0.00076 -0.00063

116 5D-2 0.00042 0.00015 0.00016 0.00043 -0.00059

117 7 C 1S -0.00030 -0.00024 -0.00039 -0.00062 0.00043

118 2S -0.00047 -0.00040 -0.00064 -0.00102 0.00070

119 2PX 0.00144 -0.00001 -0.00001 -0.00106 -0.00027

120 2PY 0.00023 0.00051 0.00079 0.00165 -0.00074

121 2PZ 0.00025 -0.00090 -0.00143 -0.00291 0.00151

122 3S 0.00136 0.00141 0.00211 0.00302 -0.00203

123 3PX 0.00192 -0.00009 -0.00011 -0.00154 -0.00046

124 3PY 0.00028 0.00054 0.00098 0.00252 -0.00098

125 3PZ 0.00050 -0.00122 -0.00196 -0.00409 0.00209

126 4S 0.00158 -0.00162 -0.00170 0.00634 -0.00361

127 4PX 0.00209 -0.00176 -0.00227 -0.00202 0.00044

128 4PY 0.00033 0.00112 0.00135 0.00324 -0.00172

129 4PZ 0.00084 0.00020 -0.00044 -0.00567 0.00316

130 5D 0 -0.00007 -0.00010 -0.00016 -0.00032 0.00005

131 5D+1 0.00005 0.00022 0.00032 0.00053 -0.00014

132 5D-1 0.00005 -0.00002 -0.00007 0.00006 -0.00011

133 5D+2 0.00007 -0.00017 -0.00028 -0.00032 -0.00007

134 5D-2 0.00006 -0.00001 0.00002 0.00007 0.00000

135 8 C 1S 0.00039 -0.00014 -0.00020 -0.00056 -0.00057

136 2S 0.00062 -0.00019 -0.00030 -0.00090 -0.00091

137 2PX -0.00179 0.00041 0.00066 0.00178 0.00014

138 2PY 0.00018 0.00027 0.00042 0.00176 -0.00314

139 2PZ 0.00003 -0.00018 -0.00029 -0.00196 0.00365

140 3S -0.00181 0.00016 0.00046 0.00261 0.00253

141 3PX -0.00242 0.00044 0.00082 0.00267 0.00047

142 3PY 0.00027 0.00017 0.00029 0.00221 -0.00482

143 3PZ 0.00007 -0.00021 -0.00032 -0.00266 0.00533

144 4S -0.00284 0.00314 0.00470 0.00048 0.00889

145 4PX -0.00246 0.00206 0.00317 0.00389 0.00199

146 4PY -0.00012 0.00138 0.00194 0.00695 -0.00516

147 4PZ 0.00082 0.00024 0.00013 -0.00421 0.00670

148 5D 0 0.00004 -0.00014 -0.00017 -0.00008 -0.00002

149 5D+1 0.00006 0.00002 0.00001 -0.00019 -0.00001

150 5D-1 0.00012 0.00001 0.00000 0.00001 -0.00016

151 5D+2 0.00021 -0.00013 -0.00019 -0.00015 -0.00010

152 5D-2 0.00036 -0.00022 -0.00035 -0.00060 -0.00019

153 9 C 1S -0.00123 0.00066 0.00110 0.00116 0.00187

154 2S -0.00194 0.00107 0.00174 0.00181 0.00293

155 2PX 0.00450 -0.00168 -0.00271 -0.00282 -0.00391

156 2PY 0.00261 -0.00113 -0.00185 -0.00248 -0.00180

157 2PZ 0.00018 -0.00017 -0.00029 -0.00011 -0.00084

158 3S 0.00555 -0.00356 -0.00552 -0.00483 -0.00843

159 3PX 0.00642 -0.00206 -0.00343 -0.00358 -0.00542

160 3PY 0.00374 -0.00147 -0.00231 -0.00267 -0.00155

161 3PZ 0.00017 -0.00018 -0.00033 -0.00010 -0.00133

162 4S -0.00186 -0.00255 -0.00550 -0.02713 -0.01743

163 4PX -0.00056 0.00039 0.00028 -0.01697 -0.00284

164 4PY 0.00240 0.00041 -0.00068 -0.00549 -0.00342

165 4PZ 0.00018 -0.00073 -0.00094 -0.00384 0.00029

166 5D 0 -0.00011 -0.00021 -0.00028 0.00006 -0.00045

167 5D+1 -0.00030 0.00008 0.00011 0.00057 -0.00057

168 5D-1 -0.00004 -0.00002 -0.00003 0.00032 -0.00010

169 5D+2 0.00007 -0.00028 -0.00046 -0.00012 0.00115

170 5D-2 0.00061 0.00018 0.00027 0.00031 0.00075

171 10 H 1S 0.01086 -0.01937 -0.03060 0.06969 0.02801

172 2S 0.01424 -0.03204 -0.04640 0.09223 0.03868

173 3S 0.00512 -0.00198 -0.00362 0.03991 0.02457

174 4PX -0.00117 0.00647 0.00822 -0.00558 -0.00242

175 4PY -0.00079 0.00266 0.00353 -0.00434 0.00319

176 4PZ -0.00009 0.00046 0.00055 -0.00077 -0.00004

177 11 H 1S -0.00138 -0.00013 -0.00015 -0.00129 0.00128

178 2S -0.00196 0.00070 0.00101 -0.00159 0.00184

179 3S -0.00115 0.00303 0.00488 0.00333 -0.00316

180 4PX 0.00000 0.00004 0.00003 0.00004 -0.00007

181 4PY 0.00007 0.00014 0.00020 0.00026 -0.00014

182 4PZ 0.00006 0.00014 0.00018 0.00027 -0.00015

183 12 H 1S -0.00033 -0.00040 -0.00067 -0.00112 0.00059

184 2S -0.00079 -0.00114 -0.00158 -0.00229 0.00082

185 3S -0.00059 -0.00112 -0.00158 -0.00226 0.00057

186 4PX 0.00007 -0.00001 -0.00001 -0.00015 0.00007

187 4PY 0.00003 0.00003 0.00005 0.00014 -0.00007

188 4PZ 0.00010 -0.00005 -0.00008 -0.00022 0.00003

189 13 H 1S 0.00084 -0.00028 -0.00044 -0.00085 -0.00063

190 2S 0.00159 -0.00028 -0.00050 -0.00068 -0.00152

191 3S 0.00099 -0.00003 -0.00015 -0.00169 -0.00120

192 4PX -0.00006 0.00002 0.00004 0.00026 -0.00012

193 4PY 0.00001 0.00000 0.00003 0.00017 -0.00030

194 4PZ 0.00008 -0.00004 -0.00004 -0.00012 0.00031

195 14 H 1S -0.00060 0.00029 0.00049 0.00034 -0.00185

196 2S -0.00046 0.00074 0.00112 0.00363 -0.00309

197 3S -0.00073 0.00302 0.00428 0.00197 -0.00089

198 4PX 0.00036 -0.00011 -0.00014 0.00017 -0.00004

199 4PY 0.00021 -0.00006 -0.00009 0.00034 0.00006

200 4PZ 0.00001 -0.00001 -0.00001 -0.00004 -0.00003

31 32 33 34 35

31 2PZ 0.07345

32 3S -0.00167 0.45920

33 3PX 0.00272 -0.01692 0.19224

34 3PY -0.00162 -0.01860 0.01296 0.18549

35 3PZ 0.11390 0.00173 0.00012 0.00001 0.17697

36 4S 0.00855 0.26150 0.01092 -0.04816 0.01448

37 4PX -0.00757 0.00764 0.12129 0.02496 -0.01401

38 4PY 0.00972 -0.05449 0.00811 0.06778 0.01591

39 4PZ 0.10056 0.01332 -0.00777 0.02005 0.15638

40 5D 0 -0.00177 -0.01352 0.00073 0.00147 -0.00267

41 5D+1 -0.00713 0.00430 0.00053 0.00098 -0.01092

42 5D-1 -0.00075 -0.00646 0.00232 0.00220 -0.00177

43 5D+2 -0.00031 -0.00582 0.00433 0.01640 -0.00049

44 5D-2 -0.00014 -0.00384 0.00668 -0.00510 -0.00036

45 3 N 1S 0.00372 -0.01462 0.01794 -0.01600 0.00547

46 2S 0.00595 -0.02170 0.02672 -0.02398 0.00816

47 2PX -0.02226 0.07505 -0.04566 0.04989 -0.03153

48 2PY 0.02133 -0.08753 0.06617 -0.02663 0.03103

49 2PZ 0.05558 0.02481 -0.03249 0.02898 0.08580

50 3S -0.01676 0.04866 -0.06994 0.06241 -0.02118

51 3PX -0.03298 0.10848 -0.06655 0.07129 -0.04679

52 3PY 0.03124 -0.12446 0.09567 -0.03327 0.04554

53 3PZ 0.08529 0.03595 -0.04847 0.04228 0.13173

54 4S -0.00373 -0.01338 -0.01255 0.01756 -0.00365

55 4PX -0.01948 0.04893 -0.02949 0.03825 -0.02795

56 4PY 0.02174 -0.09774 0.05701 -0.03591 0.03186

57 4PZ 0.06441 0.01955 -0.02901 0.03053 0.09831

58 5D 0 0.00467 -0.00741 0.00323 -0.00157 0.00708

59 5D+1 0.00422 0.00416 -0.00236 0.00386 0.00674

60 5D-1 -0.00705 -0.00599 0.00695 -0.00317 -0.01106

61 5D+2 0.00143 -0.00661 0.01151 0.00494 0.00205

62 5D-2 0.00424 -0.01640 0.01025 -0.00606 0.00603

63 4 C 1S -0.00139 0.00666 -0.00526 -0.00063 -0.00214

64 2S -0.00221 0.01070 -0.00820 -0.00114 -0.00323

65 2PX 0.00159 -0.01931 0.01133 -0.00603 0.00161

66 2PY 0.00093 0.00735 0.00318 -0.01250 0.00136

67 2PZ -0.01166 -0.00322 0.00153 -0.00082 -0.01859

68 3S 0.00541 -0.03184 0.02408 0.00326 0.00727

69 3PX 0.00306 -0.02471 0.01346 -0.00731 0.00355

70 3PY 0.00048 0.01038 0.00348 -0.01568 0.00064

71 3PZ -0.01830 -0.00476 0.00277 -0.00153 -0.02909

72 4S 0.02881 -0.02927 0.07220 0.03807 0.04236

73 4PX -0.00702 -0.04678 0.04568 -0.00571 -0.01225

74 4PY 0.00514 -0.00233 0.00722 -0.01573 0.00750

75 4PZ -0.01660 -0.00564 0.00804 -0.00071 -0.02716

76 5D 0 -0.00027 -0.00093 0.00198 0.00082 -0.00043

77 5D+1 0.00618 0.00105 -0.00335 0.00173 0.00958

78 5D-1 0.00408 0.00162 -0.00192 0.00185 0.00621

79 5D+2 -0.00322 0.01194 -0.01069 0.00249 -0.00463

80 5D-2 0.00124 -0.00555 0.00186 -0.00184 0.00183

81 5 C 1S -0.00079 0.00704 -0.00563 -0.00214 -0.00139

82 2S -0.00124 0.01127 -0.00890 -0.00353 -0.00210

83 2PX 0.00123 -0.00690 0.00645 0.00755 0.00194

84 2PY -0.00495 -0.01891 0.00762 0.00058 -0.00815

85 2PZ -0.02059 -0.00173 0.00594 -0.00591 -0.03103

86 3S 0.00384 -0.03391 0.02681 0.01032 0.00610

87 3PX 0.00283 -0.01277 0.01396 0.00985 0.00441

88 3PY -0.00773 -0.02571 0.01111 0.00173 -0.01265

89 3PZ -0.03116 -0.00339 0.00978 -0.00935 -0.04691

90 4S 0.01004 -0.03160 0.05061 0.02157 0.01551

91 4PX 0.00162 0.00180 0.00030 0.02005 0.00237

92 4PY -0.01480 -0.02110 -0.02897 -0.01817 -0.02271

93 4PZ -0.03980 -0.00535 0.01865 -0.00776 -0.06037

94 5D 0 0.00118 -0.00184 0.00170 0.00196 0.00186

95 5D+1 0.00196 -0.00043 -0.00026 0.00108 0.00336

96 5D-1 -0.00035 0.00072 -0.00022 -0.00170 -0.00060

97 5D+2 0.00014 0.00286 -0.00125 -0.00439 0.00032

98 5D-2 0.00068 0.00666 -0.00444 -0.00847 0.00109

99 6 C 1S 0.00110 -0.00522 0.00302 -0.00108 0.00160

100 2S 0.00168 -0.00831 0.00489 -0.00170 0.00241

101 2PX -0.00845 0.01121 -0.00301 0.00103 -0.01281

102 2PY -0.00201 -0.00459 0.00299 0.00075 -0.00328

103 2PZ -0.00466 -0.00927 0.00805 -0.00285 -0.00762

104 3S -0.00434 0.02540 -0.01535 0.00476 -0.00603

105 3PX -0.01303 0.01682 -0.00601 0.00116 -0.01974

106 3PY -0.00399 -0.00578 0.00414 0.00125 -0.00646

107 3PZ -0.00770 -0.01241 0.01134 -0.00429 -0.01249

108 4S -0.01249 0.00952 -0.01511 0.00392 -0.01844

109 4PX -0.01658 0.00713 0.00010 -0.00456 -0.02519

110 4PY 0.00286 0.00810 -0.00055 0.00407 0.00442

111 4PZ -0.00648 -0.01067 0.00926 -0.00148 -0.01048

112 5D 0 0.00074 0.00109 -0.00081 0.00033 0.00121

113 5D+1 -0.00149 -0.00040 0.00001 0.00002 -0.00235

114 5D-1 0.00077 -0.00009 -0.00001 0.00015 0.00120

115 5D+2 0.00019 0.00028 0.00087 -0.00102 0.00023

116 5D-2 0.00064 -0.00023 0.00051 -0.00093 0.00096

117 7 C 1S -0.00106 0.00137 -0.00119 0.00078 -0.00163

118 2S -0.00178 0.00214 -0.00175 0.00115 -0.00272

119 2PX 0.00363 -0.00009 -0.00115 -0.00022 0.00572

120 2PY 0.00176 -0.00246 0.00202 -0.00130 0.00262

121 2PZ -0.00059 0.00461 -0.00374 0.00231 -0.00057

122 3S 0.00702 -0.00641 0.00449 -0.00296 0.01068

123 3PX 0.00533 0.00004 -0.00167 -0.00043 0.00844

124 3PY 0.00291 -0.00364 0.00312 -0.00174 0.00433

125 3PZ -0.00131 0.00649 -0.00522 0.00319 -0.00154

126 4S 0.00019 0.00091 0.00879 -0.00530 0.00007

127 4PX 0.00581 0.00470 -0.00252 0.00079 0.00922

128 4PY 0.00077 -0.00267 0.00410 -0.00289 0.00113

129 4PZ 0.00578 0.00461 -0.00758 0.00497 0.00957

130 5D 0 0.00023 0.00053 -0.00050 -0.00001 0.00040

131 5D+1 0.00005 -0.00089 0.00076 -0.00008 0.00004

132 5D-1 -0.00098 0.00034 0.00012 -0.00015 -0.00151

133 5D+2 -0.00090 0.00096 -0.00042 -0.00018 -0.00134

134 5D-2 0.00042 -0.00025 0.00011 0.00004 0.00062

135 8 C 1S 0.00046 0.00043 -0.00078 -0.00084 0.00070

136 2S 0.00078 0.00084 -0.00131 -0.00136 0.00119

137 2PX -0.00581 -0.00187 0.00270 0.00004 -0.00882

138 2PY -0.00950 -0.00139 0.00263 -0.00453 -0.01470

139 2PZ 0.01301 0.00097 -0.00280 0.00549 0.01988

140 3S -0.00331 -0.00211 0.00391 0.00373 -0.00514

141 3PX -0.00861 -0.00277 0.00408 0.00048 -0.01307

142 3PY -0.01415 -0.00112 0.00329 -0.00706 -0.02187

143 3PZ 0.01885 0.00106 -0.00380 0.00807 0.02879

144 4S -0.00045 -0.01234 0.00145 0.01373 -0.00038

145 4PX -0.00839 -0.00871 0.00612 0.00309 -0.01259

146 4PY -0.01558 -0.00538 0.01011 -0.00692 -0.02407

147 4PZ 0.02605 0.00078 -0.00586 0.01019 0.04007

148 5D 0 -0.00029 0.00035 -0.00012 -0.00004 -0.00044

149 5D+1 0.00035 0.00007 -0.00029 -0.00004 0.00053

150 5D-1 -0.00033 0.00005 0.00004 -0.00024 -0.00051

151 5D+2 -0.00013 0.00054 -0.00018 -0.00021 -0.00017

152 5D-2 0.00043 0.00112 -0.00085 -0.00041 0.00071

153 9 C 1S 0.00004 -0.00359 0.00177 0.00297 0.00003

154 2S 0.00007 -0.00549 0.00279 0.00474 0.00005

155 2PX 0.00049 0.00843 -0.00422 -0.00651 0.00087

156 2PY 0.00058 0.00633 -0.00327 -0.00381 0.00120

157 2PZ -0.00071 0.00087 -0.00049 -0.00138 -0.00121

158 3S 0.00009 0.01619 -0.00759 -0.01402 0.00034

159 3PX 0.00142 0.01113 -0.00534 -0.00904 0.00236

160 3PY 0.00038 0.00778 -0.00342 -0.00390 0.00102

161 3PZ -0.00079 0.00114 -0.00064 -0.00214 -0.00134

162 4S -0.00994 0.02130 -0.04182 -0.02805 -0.01543

163 4PX -0.00509 0.00129 -0.02460 -0.00496 -0.00740

164 4PY -0.00034 0.00734 -0.00840 -0.00626 -0.00050

165 4PZ 0.00313 0.00228 -0.00585 -0.00012 0.00440

166 5D 0 -0.00038 0.00063 0.00003 -0.00066 -0.00058

167 5D+1 -0.00301 -0.00027 0.00078 -0.00091 -0.00456

168 5D-1 -0.00085 0.00011 0.00044 -0.00021 -0.00128

169 5D+2 0.00175 0.00158 -0.00017 0.00135 0.00276

170 5D-2 0.00144 -0.00067 0.00055 0.00107 0.00224

171 10 H 1S 0.00512 0.08115 0.09992 0.04163 0.00710

172 2S 0.00714 0.11123 0.13162 0.05729 0.00994

173 3S 0.00077 0.00981 0.05735 0.03848 0.00050

174 4PX -0.00121 -0.01632 -0.00800 -0.00306 -0.00192

175 4PY -0.00039 -0.00742 -0.00599 0.00486 -0.00047

176 4PZ 0.00477 -0.00100 -0.00123 0.00005 0.00742

177 11 H 1S -0.00221 0.00016 -0.00192 0.00215 -0.00337

178 2S -0.00580 -0.00268 -0.00216 0.00327 -0.00895

179 3S -0.00454 -0.01499 0.00573 -0.00365 -0.00724

180 4PX -0.00018 0.00005 0.00009 -0.00008 -0.00028

181 4PY 0.00000 -0.00056 0.00043 -0.00016 -0.00002

182 4PZ -0.00054 -0.00044 0.00040 -0.00016 -0.00085

183 12 H 1S -0.00123 0.00230 -0.00152 0.00064 -0.00174

184 2S -0.00220 0.00425 -0.00312 0.00071 -0.00316

185 3S -0.00356 0.00426 -0.00319 0.00035 -0.00541

186 4PX 0.00040 0.00001 -0.00017 0.00012 0.00065

187 4PY 0.00007 -0.00020 0.00017 -0.00013 0.00010

188 4PZ -0.00003 0.00031 -0.00029 0.00004 -0.00003

189 13 H 1S 0.00027 0.00132 -0.00133 -0.00100 0.00046

190 2S 0.00091 0.00166 -0.00106 -0.00229 0.00148

191 3S 0.00334 0.00096 -0.00247 -0.00188 0.00523

192 4PX -0.00073 -0.00014 0.00037 -0.00017 -0.00111

193 4PY -0.00109 -0.00017 0.00025 -0.00043 -0.00168

194 4PZ 0.00124 0.00006 -0.00018 0.00048 0.00189

195 14 H 1S -0.00242 -0.00212 0.00015 -0.00201 -0.00396

196 2S -0.00456 -0.00405 0.00481 -0.00338 -0.00743

197 3S -0.00405 -0.01118 0.00305 0.00002 -0.00648

198 4PX -0.00014 0.00034 0.00025 -0.00008 -0.00022

199 4PY 0.00016 0.00022 0.00044 0.00008 0.00023

200 4PZ 0.00005 0.00005 -0.00008 -0.00006 0.00008

36 37 38 39 40

36 4S 0.18842

37 4PX 0.00164 0.09622

38 4PY -0.04884 -0.00062 0.04552

39 4PZ 0.01419 -0.01192 0.01721 0.14699

40 5D 0 -0.00623 -0.00055 0.00149 -0.00260 0.00105

41 5D+1 0.00089 0.00245 -0.00150 -0.00827 0.00046

42 5D-1 -0.00373 0.00250 0.00004 -0.00030 -0.00065

43 5D+2 -0.00764 0.01106 0.00070 0.00260 0.00014

44 5D-2 0.00007 0.00468 -0.00075 -0.00142 0.00024

45 3 N 1S 0.01417 0.00307 -0.00632 0.00149 0.00177

46 2S 0.01938 0.00728 -0.01171 0.00307 0.00240

47 2PX 0.04005 -0.04488 0.02335 -0.02446 0.00017

48 2PY -0.02943 0.00459 0.02900 0.00969 0.00133

49 2PZ 0.01116 -0.02256 0.01089 0.08020 -0.00590

50 3S -0.04820 -0.02963 0.03888 -0.01115 -0.00513

51 3PX 0.05894 -0.06647 0.03420 -0.03681 0.00035

52 3PY -0.04153 0.00736 0.04280 0.01546 0.00196

53 3PZ 0.01657 -0.03437 0.01675 0.12242 -0.00876

54 4S -0.06010 -0.00719 0.03344 -0.00674 -0.00363

55 4PX 0.02735 -0.03336 0.02020 -0.02327 0.00086

56 4PY -0.03346 -0.01511 0.03835 0.00533 0.00097

57 4PZ 0.00803 -0.01908 0.01018 0.09363 -0.00788

58 5D 0 -0.00176 -0.00138 0.00223 0.00518 0.00012

59 5D+1 0.00182 -0.00172 0.00200 0.00751 -0.00002

60 5D-1 -0.00273 0.00435 -0.00078 -0.01005 0.00074

61 5D+2 -0.00369 0.00613 0.00430 0.00108 0.00013

62 5D-2 -0.00880 0.00851 -0.00204 0.00464 0.00011

63 4 C 1S 0.01112 -0.01431 -0.00056 -0.00392 0.00025

64 2S 0.01590 -0.02043 0.00012 -0.00614 0.00047

65 2PX -0.02955 0.03117 -0.01421 0.00587 -0.00149

66 2PY 0.00361 0.01296 -0.01462 0.00203 0.00054

67 2PZ -0.00489 0.00455 -0.00544 -0.03081 -0.00290

68 3S -0.03924 0.05094 -0.00372 0.01604 -0.00131

69 3PX -0.04331 0.04627 -0.02181 0.01016 -0.00257

70 3PY 0.00386 0.02037 -0.02224 0.00269 0.00086

71 3PZ -0.00754 0.00742 -0.00868 -0.04824 -0.00436

72 4S -0.02696 0.08066 -0.00537 0.04960 -0.00100

73 4PX -0.02975 0.03943 -0.00826 -0.01023 0.00197

74 4PY 0.00269 0.00120 -0.00167 0.00333 -0.00063

75 4PZ -0.00806 0.01370 -0.01159 -0.04220 -0.00481

76 5D 0 0.00088 -0.00040 0.00061 -0.00155 0.00010

77 5D+1 -0.00044 -0.00152 0.00051 0.00840 -0.00071

78 5D-1 0.00117 -0.00138 0.00072 0.00712 -0.00015

79 5D+2 0.00454 -0.00290 -0.00370 -0.00187 -0.00003

80 5D-2 -0.00502 0.00306 -0.00233 0.00171 -0.00004

81 5 C 1S 0.00750 -0.00686 -0.00574 -0.00082 0.00057

82 2S 0.01147 -0.00989 -0.00907 -0.00116 0.00091

83 2PX -0.01653 0.00801 0.01627 0.00193 -0.00084

84 2PY -0.01625 0.00216 0.00761 -0.01383 -0.00132

85 2PZ -0.00409 0.00944 -0.00896 -0.04207 -0.00009

86 3S -0.03065 0.02491 0.02513 0.00304 -0.00251

87 3PX -0.02748 0.01436 0.02678 0.00340 -0.00143

88 3PY -0.02225 0.00320 0.01048 -0.02034 -0.00180

89 3PZ -0.00688 0.01474 -0.01310 -0.06420 -0.00010

90 4S -0.01864 0.02072 0.04277 0.01013 -0.00165

91 4PX -0.01340 0.01804 0.01031 0.00844 -0.00161

92 4PY -0.01797 -0.02624 0.00487 -0.03177 -0.00144

93 4PZ -0.00776 0.02398 -0.01708 -0.07704 0.00039

94 5D 0 0.00009 0.00005 0.00074 0.00201 0.00040

95 5D+1 -0.00096 -0.00037 0.00180 0.00172 0.00030

96 5D-1 0.00106 -0.00047 -0.00101 -0.00218 -0.00024

97 5D+2 0.00133 -0.00260 0.00125 -0.00045 -0.00006

98 5D-2 0.00613 -0.00162 -0.00658 0.00073 -0.00017

99 6 C 1S -0.00435 0.00186 -0.00084 0.00108 -0.00017

100 2S -0.00719 0.00368 -0.00130 0.00182 -0.00028

101 2PX 0.01174 -0.00412 0.00189 -0.01534 0.00073

102 2PY -0.00586 0.00542 -0.00052 -0.00406 -0.00012

103 2PZ -0.00867 0.00748 -0.00263 -0.00772 0.00019

104 3S 0.02213 -0.01316 0.00312 -0.00490 0.00074

105 3PX 0.01632 -0.00590 0.00190 -0.02347 0.00094

106 3PY -0.00787 0.00783 -0.00129 -0.00730 -0.00018

107 3PZ -0.01219 0.01131 -0.00447 -0.01258 0.00031

108 4S 0.01444 -0.01743 0.00488 -0.01098 0.00351

109 4PX 0.00980 -0.00456 0.00175 -0.02576 0.00226

110 4PY -0.00096 0.00871 -0.00374 0.00311 -0.00093

111 4PZ -0.00931 0.00607 0.00099 -0.01070 0.00060

112 5D 0 0.00089 -0.00086 0.00031 0.00224 0.00025

113 5D+1 -0.00090 0.00049 -0.00047 -0.00341 -0.00028

114 5D-1 0.00052 -0.00073 0.00067 0.00092 -0.00003

115 5D+2 -0.00036 0.00229 -0.00183 -0.00001 -0.00023

116 5D-2 -0.00078 0.00154 -0.00136 0.00178 0.00005

117 7 C 1S 0.00144 -0.00136 0.00026 -0.00210 -0.00002

118 2S 0.00211 -0.00201 0.00047 -0.00341 -0.00003

119 2PX -0.00016 -0.00223 0.00175 0.01031 0.00122

120 2PY -0.00214 0.00171 -0.00276 0.00284 -0.00062

121 2PZ 0.00488 -0.00560 0.00377 0.00104 0.00115

122 3S -0.00556 0.00498 -0.00120 0.01253 0.00009

123 3PX 0.00007 -0.00345 0.00283 0.01451 0.00177

124 3PY -0.00298 0.00220 -0.00368 0.00440 -0.00098

125 3PZ 0.00703 -0.00808 0.00526 0.00107 0.00172

126 4S -0.00319 0.01414 -0.00619 0.00145 0.00006

127 4PX 0.00193 -0.00194 0.00211 0.01655 0.00177

128 4PY -0.00164 0.00331 -0.00458 0.00027 -0.00078

129 4PZ 0.00632 -0.01057 0.00735 0.01457 0.00227

130 5D 0 0.00047 -0.00032 -0.00007 0.00020 -0.00002

131 5D+1 -0.00078 0.00080 -0.00004 0.00007 0.00004

132 5D-1 0.00015 0.00037 -0.00019 -0.00167 0.00008

133 5D+2 0.00115 -0.00065 -0.00016 -0.00166 0.00006

134 5D-2 -0.00026 0.00009 0.00011 0.00087 0.00002

135 8 C 1S 0.00348 -0.00247 -0.00056 0.00060 -0.00003

136 2S 0.00454 -0.00308 -0.00106 0.00117 0.00000

137 2PX -0.00492 0.00212 0.00197 -0.01236 -0.00009

138 2PY -0.00131 0.00542 -0.00499 -0.01886 0.00014

139 2PZ -0.00037 -0.00406 0.00669 0.02603 -0.00023

140 3S -0.00982 0.00654 0.00309 -0.00539 -0.00019

141 3PX -0.00696 0.00321 0.00277 -0.01817 -0.00011

142 3PY -0.00163 0.00804 -0.00775 -0.02769 0.00020

143 3PZ -0.00079 -0.00554 0.00963 0.03777 -0.00033

144 4S -0.01546 0.00420 0.01094 -0.00070 0.00067

145 4PX -0.00872 0.00334 0.00502 -0.01897 0.00027

146 4PY -0.00356 0.01102 -0.00644 -0.03329 0.00030

147 4PZ 0.00127 -0.00942 0.01226 0.05012 0.00010

148 5D 0 0.00019 0.00012 -0.00018 -0.00024 0.00003

149 5D+1 0.00009 -0.00029 -0.00020 0.00103 -0.00002

150 5D-1 0.00005 0.00028 -0.00027 -0.00057 0.00007

151 5D+2 0.00051 -0.00026 -0.00031 -0.00020 0.00006

152 5D-2 0.00112 -0.00057 -0.00060 0.00116 0.00008

153 9 C 1S -0.00451 -0.00064 0.00419 -0.00036 -0.00008

154 2S -0.00747 0.00001 0.00669 -0.00042 -0.00010

155 2PX 0.01325 0.00005 -0.01141 0.00221 0.00051

156 2PY 0.00433 -0.00070 -0.00551 0.00237 0.00032

157 2PZ 0.00145 0.00009 -0.00311 0.00133 -0.00040

158 3S 0.02282 -0.00300 -0.01953 0.00109 0.00010

159 3PX 0.01814 0.00022 -0.01528 0.00425 0.00074

160 3PY 0.00444 0.00064 -0.00722 0.00281 0.00041

161 3PZ 0.00209 0.00004 -0.00459 0.00196 -0.00057

162 4S 0.01551 -0.01332 -0.03176 -0.01231 -0.00136

163 4PX 0.00297 -0.01096 -0.00941 -0.00650 0.00058

164 4PY 0.00313 0.00009 -0.00984 0.00380 0.00010

165 4PZ 0.00201 -0.00467 -0.00175 0.01223 -0.00098

166 5D 0 0.00024 0.00013 -0.00042 -0.00092 0.00000

167 5D+1 -0.00052 0.00120 -0.00120 -0.00604 0.00001

168 5D-1 -0.00015 0.00061 -0.00043 -0.00155 -0.00002

169 5D+2 -0.00006 0.00042 0.00131 0.00382 0.00000

170 5D-2 0.00029 0.00001 0.00041 0.00290 0.00006

171 10 H 1S 0.04741 0.07657 0.00352 0.01019 -0.00242

172 2S 0.06396 0.10552 0.00161 0.01547 -0.00337

173 3S -0.00006 0.04998 0.00736 0.00382 -0.00052

174 4PX -0.00986 -0.00569 0.00040 -0.00218 0.00044

175 4PY -0.00626 -0.00332 0.00246 0.00024 0.00022

176 4PZ -0.00012 -0.00145 0.00081 0.00669 -0.00007

177 11 H 1S -0.00103 -0.00047 0.00151 -0.00396 0.00002

178 2S -0.00342 -0.00088 0.00268 -0.00956 0.00021

179 3S -0.00825 -0.00258 0.00572 -0.01097 0.00027

180 4PX 0.00033 -0.00021 0.00036 -0.00059 0.00003

181 4PY -0.00021 -0.00006 0.00009 -0.00012 0.00001

182 4PZ -0.00054 0.00059 -0.00029 -0.00079 0.00003

183 12 H 1S 0.00262 -0.00219 0.00066 -0.00271 0.00002

184 2S 0.00448 -0.00448 0.00126 -0.00540 -0.00019

185 3S 0.00336 -0.00303 -0.00044 -0.00650 -0.00033

186 4PX 0.00014 -0.00046 0.00044 0.00092 0.00012

187 4PY -0.00020 0.00019 -0.00028 0.00011 -0.00006

188 4PZ 0.00024 -0.00029 0.00011 0.00030 0.00011

189 13 H 1S 0.00226 -0.00074 -0.00259 0.00140 0.00012

190 2S 0.00421 -0.00162 -0.00388 0.00254 0.00028

191 3S 0.00352 -0.00354 -0.00162 0.00608 0.00014

192 4PX -0.00018 0.00035 -0.00024 -0.00148 0.00000

193 4PY -0.00009 0.00051 -0.00058 -0.00209 0.00001

194 4PZ 0.00014 -0.00035 0.00042 0.00242 -0.00002

195 14 H 1S 0.00245 -0.00083 -0.00186 -0.00529 -0.00017

196 2S 0.00271 0.00141 -0.00219 -0.01014 -0.00023

197 3S -0.00555 -0.00039 0.00207 -0.00896 0.00026

198 4PX 0.00048 0.00045 -0.00063 -0.00017 0.00005

199 4PY 0.00060 0.00041 -0.00030 0.00041 -0.00001

200 4PZ 0.00000 -0.00005 -0.00019 0.00022 -0.00001

41 42 43 44 45

41 5D+1 0.00147

42 5D-1 -0.00062 0.00233

43 5D+2 0.00051 0.00088 0.00542

44 5D-2 0.00004 0.00000 0.00067 0.00232

45 3 N 1S -0.00123 0.00190 0.00218 0.00628 0.65106

46 2S -0.00157 0.00262 0.00336 0.00850 0.56642

47 2PX 0.00161 -0.00374 -0.00839 -0.00377 -0.00248

48 2PY -0.00550 -0.00081 -0.01685 0.00050 -0.01277

49 2PZ -0.00944 0.00699 0.00231 -0.00407 0.00127

50 3S 0.00307 -0.00612 -0.00955 -0.01945 -0.11410

51 3PX 0.00229 -0.00548 -0.01265 -0.00516 -0.00085

52 3PY -0.00795 -0.00100 -0.02357 0.00068 -0.01609

53 3PZ -0.01434 0.01017 0.00319 -0.00598 0.00205

54 4S 0.00039 -0.00475 -0.01320 -0.01406 -0.12403

55 4PX 0.00164 -0.00361 -0.00728 -0.00272 0.00124

56 4PY -0.00682 -0.00206 -0.02517 -0.00170 -0.02147

57 4PZ -0.01171 0.01148 0.00360 -0.00485 0.00272

58 5D 0 -0.00074 0.00000 -0.00117 -0.00009 0.00021

59 5D+1 -0.00002 -0.00028 0.00008 -0.00018 -0.00039

60 5D-1 0.00117 -0.00052 -0.00031 0.00051 -0.00008

61 5D+2 -0.00032 0.00013 -0.00027 0.00002 -0.00160

62 5D-2 -0.00029 0.00056 0.00147 0.00075 0.00041

63 4 C 1S -0.00076 -0.00063 -0.00353 -0.00067 0.00224

64 2S -0.00123 -0.00092 -0.00568 -0.00108 0.00325

65 2PX 0.00191 0.00161 0.01104 0.00167 -0.00815

66 2PY -0.00008 0.00095 0.00290 -0.00057 -0.00630

67 2PZ -0.00451 0.00330 0.00137 -0.00056 0.00071

68 3S 0.00371 0.00237 0.01623 0.00327 -0.00684

69 3PX 0.00263 0.00276 0.01728 0.00247 -0.01343

70 3PY 0.00024 0.00142 0.00544 -0.00101 -0.01053

71 3PZ -0.00688 0.00481 0.00219 -0.00076 0.00126

72 4S 0.00015 0.00577 0.02169 0.00051 0.02024

73 4PX 0.00337 -0.00093 0.00624 0.00093 0.00428

74 4PY -0.00225 0.00179 -0.00318 0.00071 -0.00423

75 4PZ -0.00659 0.00696 0.00370 -0.00125 0.00287

76 5D 0 -0.00033 -0.00006 -0.00045 -0.00001 0.00138

77 5D+1 -0.00107 0.00067 0.00060 -0.00015 -0.00030

78 5D-1 -0.00025 0.00049 0.00009 -0.00040 -0.00006

79 5D+2 0.00069 -0.00004 0.00149 -0.00036 -0.00049

80 5D-2 0.00007 -0.00006 0.00049 -0.00121 -0.00549

81 5 C 1S 0.00034 -0.00052 -0.00040 -0.00228 -0.00087

82 2S 0.00057 -0.00082 -0.00063 -0.00364 -0.00129

83 2PX -0.00063 0.00157 0.00225 0.00820 0.00234

84 2PY -0.00044 -0.00026 0.00072 0.00172 0.00176

85 2PZ -0.00159 -0.00140 0.00142 0.00118 -0.00103

86 3S -0.00181 0.00239 0.00155 0.00992 0.00306

87 3PX -0.00122 0.00239 0.00280 0.01317 0.00376

88 3PY -0.00045 -0.00050 0.00094 0.00191 0.00109

89 3PZ -0.00244 -0.00234 0.00208 0.00206 -0.00120

90 4S -0.00315 0.00197 -0.00548 0.01063 -0.00603

91 4PX -0.00036 0.00460 0.01034 0.01089 0.00815

92 4PY -0.00082 -0.00143 -0.00360 0.00220 -0.00507

93 4PZ -0.00122 -0.00282 0.00409 0.00215 -0.00035

94 5D 0 0.00018 -0.00042 -0.00007 -0.00033 -0.00018

95 5D+1 -0.00009 -0.00101 0.00003 0.00053 0.00022

96 5D-1 -0.00056 0.00027 -0.00025 -0.00008 0.00008

97 5D+2 -0.00028 -0.00004 -0.00144 0.00085 0.00074

98 5D-2 0.00010 -0.00028 0.00012 -0.00027 -0.00028

99 6 C 1S 0.00009 0.00008 0.00064 0.00008 -0.00147

100 2S 0.00012 0.00010 0.00102 0.00008 -0.00227

101 2PX -0.00036 -0.00036 -0.00210 -0.00003 0.00376

102 2PY 0.00042 -0.00021 0.00126 0.00045 0.00052

103 2PZ 0.00095 -0.00051 0.00136 0.00015 -0.00157

104 3S -0.00026 -0.00018 -0.00270 -0.00003 0.00647

105 3PX -0.00050 -0.00053 -0.00266 -0.00004 0.00437

106 3PY 0.00068 -0.00019 0.00189 0.00055 0.00108

107 3PZ 0.00151 -0.00082 0.00227 0.00036 -0.00181

108 4S 0.00374 -0.00346 -0.00406 0.00027 0.00852

109 4PX 0.00213 -0.00297 -0.00377 0.00052 0.00072

110 4PY -0.00016 0.00016 0.00420 0.00086 0.00172

111 4PZ 0.00135 -0.00106 0.00003 -0.00002 -0.00217

112 5D 0 0.00037 -0.00030 -0.00009 0.00004 0.00019

113 5D+1 -0.00031 0.00023 0.00021 0.00006 0.00021

114 5D-1 -0.00019 0.00008 -0.00032 -0.00005 0.00008

115 5D+2 -0.00028 0.00048 0.00068 -0.00005 -0.00106

116 5D-2 0.00026 0.00001 0.00058 0.00013 -0.00043

117 7 C 1S -0.00012 0.00005 -0.00033 -0.00015 0.00056

118 2S -0.00020 0.00007 -0.00053 -0.00024 0.00088

119 2PX 0.00199 -0.00172 -0.00030 0.00012 -0.00053

120 2PY -0.00060 0.00098 0.00079 -0.00041 -0.00131

121 2PZ 0.00115 -0.00161 -0.00159 -0.00018 0.00178

122 3S 0.00058 -0.00020 0.00150 0.00069 -0.00248

123 3PX 0.00275 -0.00259 -0.00055 0.00025 -0.00063

124 3PY -0.00100 0.00148 0.00098 -0.00067 -0.00204

125 3PZ 0.00177 -0.00233 -0.00234 -0.00031 0.00260

126 4S 0.00136 -0.00050 0.00358 0.00170 -0.00333

127 4PX 0.00316 -0.00259 0.00015 0.00031 -0.00043

128 4PY -0.00109 0.00134 0.00091 -0.00048 -0.00137

129 4PZ 0.00239 -0.00311 -0.00221 -0.00018 0.00338

130 5D 0 -0.00009 0.00001 -0.00002 0.00001 -0.00005

131 5D+1 0.00010 -0.00007 0.00015 0.00006 0.00008

132 5D-1 0.00009 -0.00010 0.00004 0.00006 0.00016

133 5D+2 -0.00005 -0.00009 -0.00022 -0.00007 0.00014

134 5D-2 0.00009 -0.00006 0.00005 0.00003 -0.00002

135 8 C 1S 0.00001 -0.00012 -0.00034 -0.00045 -0.00013

136 2S 0.00002 -0.00020 -0.00056 -0.00075 -0.00016

137 2PX -0.00047 0.00004 0.00089 0.00178 -0.00002

138 2PY 0.00008 -0.00076 0.00073 0.00017 -0.00041

139 2PZ 0.00026 0.00124 -0.00028 0.00000 0.00064

140 3S -0.00004 0.00071 0.00185 0.00230 0.00022

141 3PX -0.00065 0.00000 0.00124 0.00239 -0.00040

142 3PY 0.00020 -0.00108 0.00118 0.00032 -0.00065

143 3PZ 0.00040 0.00180 -0.00035 -0.00003 0.00086

144 4S 0.00023 -0.00048 0.00207 0.00327 0.00076

145 4PX -0.00081 -0.00073 0.00055 0.00243 -0.00040

146 4PY -0.00022 -0.00171 0.00097 0.00081 0.00017

147 4PZ 0.00050 0.00113 -0.00129 -0.00059 0.00117

148 5D 0 0.00009 -0.00005 0.00002 0.00000 -0.00002

149 5D+1 0.00001 0.00010 -0.00007 -0.00017 -0.00004

150 5D-1 0.00007 -0.00011 0.00003 0.00002 0.00001

151 5D+2 0.00001 -0.00006 -0.00012 -0.00018 -0.00008

152 5D-2 0.00009 -0.00007 -0.00017 -0.00027 -0.00001

153 9 C 1S -0.00002 0.00009 0.00026 0.00100 0.00043

154 2S -0.00003 0.00012 0.00036 0.00151 0.00074

155 2PX 0.00055 -0.00080 -0.00062 -0.00297 -0.00085

156 2PY 0.00000 0.00016 -0.00045 -0.00194 -0.00112

157 2PZ -0.00006 0.00091 -0.00026 -0.00114 -0.00035

158 3S 0.00000 -0.00018 -0.00086 -0.00412 -0.00245

159 3PX 0.00075 -0.00113 -0.00103 -0.00409 -0.00117

160 3PY -0.00002 0.00040 -0.00026 -0.00259 -0.00194

161 3PZ -0.00019 0.00122 -0.00042 -0.00169 -0.00050

162 4S 0.00069 0.00168 0.00465 -0.00140 0.00221

163 4PX 0.00084 -0.00118 0.00100 -0.00148 -0.00140

164 4PY 0.00050 0.00128 0.00164 -0.00141 0.00235

165 4PZ 0.00002 0.00281 -0.00087 -0.00193 -0.00098

166 5D 0 0.00000 -0.00009 0.00006 0.00009 0.00001

167 5D+1 -0.00015 -0.00021 0.00020 0.00020 -0.00006

168 5D-1 -0.00008 0.00003 0.00006 0.00003 -0.00004

169 5D+2 -0.00004 0.00034 0.00023 0.00060 0.00030

170 5D-2 0.00000 0.00005 -0.00025 -0.00043 -0.00024

171 10 H 1S 0.00118 0.00117 0.00814 0.00320 0.00548

172 2S 0.00166 0.00228 0.01348 0.00528 0.01100

173 3S 0.00065 0.00206 0.00951 0.00321 0.00609

174 4PX -0.00009 0.00014 -0.00013 0.00005 0.00076

175 4PY -0.00003 0.00012 0.00052 -0.00016 -0.00046

176 4PZ -0.00044 -0.00007 -0.00004 -0.00006 0.00021

177 11 H 1S 0.00029 -0.00044 -0.00010 0.00019 0.00144

178 2S 0.00070 -0.00077 -0.00046 0.00008 0.00173

179 3S 0.00004 -0.00117 -0.00363 -0.00054 -0.00384

180 4PX -0.00007 -0.00002 -0.00021 0.00001 0.00000

181 4PY -0.00001 -0.00003 -0.00007 -0.00002 0.00006

182 4PZ 0.00011 -0.00005 0.00014 0.00001 -0.00005

183 12 H 1S -0.00034 0.00002 -0.00065 -0.00018 0.00069

184 2S -0.00089 0.00025 -0.00137 -0.00038 0.00074

185 3S -0.00066 0.00060 -0.00075 -0.00044 0.00049

186 4PX 0.00014 -0.00018 -0.00014 0.00000 0.00006

187 4PY -0.00006 0.00011 0.00008 -0.00004 -0.00016

188 4PZ 0.00016 -0.00015 -0.00005 0.00000 0.00001

189 13 H 1S 0.00016 -0.00021 -0.00046 -0.00094 -0.00019

190 2S 0.00022 -0.00056 -0.00119 -0.00176 -0.00044

191 3S -0.00006 -0.00025 -0.00119 -0.00133 -0.00044

192 4PX -0.00003 -0.00006 0.00003 0.00004 -0.00004

193 4PY 0.00001 -0.00008 0.00005 -0.00001 -0.00008

194 4PZ 0.00002 0.00008 -0.00008 -0.00009 0.00002

195 14 H 1S 0.00012 -0.00052 -0.00030 -0.00015 0.00006

196 2S 0.00013 -0.00080 -0.00070 -0.00014 0.00074

197 3S 0.00025 -0.00100 -0.00086 -0.00038 0.00014

198 4PX 0.00005 -0.00006 0.00003 -0.00021 -0.00006

199 4PY 0.00000 0.00004 0.00001 -0.00003 0.00002

200 4PZ -0.00001 0.00003 -0.00002 -0.00011 -0.00005

46 47 48 49 50

46 2S 0.50865

47 2PX -0.00372 0.09616

48 2PY -0.01974 -0.00074 0.13042

49 2PZ 0.00200 -0.00075 -0.00619 0.09390

50 3S -0.19407 0.00947 0.05653 -0.00601 0.58670

51 3PX -0.00247 0.14163 0.00051 -0.00195 0.01007

52 3PY -0.02539 0.00058 0.18469 -0.00707 0.07479

53 3PZ 0.00302 -0.00163 -0.00723 0.14104 -0.00848

54 4S -0.18409 -0.01677 0.09910 -0.01397 0.47832

55 4PX 0.00127 0.07735 0.00511 -0.00691 -0.00152

56 4PY -0.03801 0.01557 0.17140 -0.00718 0.12273

57 4PZ 0.00488 -0.01143 -0.00656 0.11711 -0.01593

58 5D 0 0.00056 0.00004 0.00882 0.00270 -0.00199

59 5D+1 -0.00083 0.00164 -0.00100 0.00248 0.00290

60 5D-1 0.00002 -0.00078 0.00252 -0.01173 -0.00041

61 5D+2 -0.00268 -0.00187 0.00947 -0.00052 0.00814

62 5D-2 0.00122 -0.01494 0.00226 -0.00023 -0.00523

63 4 C 1S 0.00330 0.01604 0.00944 0.00183 -0.00653

64 2S 0.00483 0.02532 0.01518 0.00295 -0.00996

65 2PX -0.01290 -0.05062 -0.02864 -0.00364 0.03528

66 2PY -0.01010 -0.03772 -0.01935 -0.00020 0.02967

67 2PZ 0.00115 -0.00132 0.00337 0.02419 -0.00377

68 3S -0.01007 -0.07038 -0.04373 -0.01008 0.02015

69 3PX -0.02055 -0.07695 -0.04768 -0.00189 0.05387

70 3PY -0.01609 -0.05717 -0.03350 -0.00070 0.04454

71 3PZ 0.00193 -0.00220 0.00491 0.03614 -0.00585

72 4S 0.02677 -0.08268 -0.05292 0.02147 -0.06079

73 4PX 0.00221 -0.03831 0.00196 -0.02935 0.00893

74 4PY -0.00731 -0.01448 0.01735 0.00680 0.02320

75 4PZ 0.00391 -0.01158 -0.00105 0.04028 -0.00939

76 5D 0 0.00222 0.00274 0.00263 0.00078 -0.00614

77 5D+1 -0.00044 -0.00224 -0.00088 0.00946 0.00107

78 5D-1 -0.00007 -0.00121 -0.00130 0.00521 0.00013

79 5D+2 -0.00056 0.00027 -0.01538 -0.00011 0.00079

80 5D-2 -0.00831 -0.00879 0.00111 -0.00049 0.02242

81 5 C 1S -0.00137 -0.00118 -0.00665 -0.00011 0.00487

82 2S -0.00207 -0.00179 -0.01074 -0.00016 0.00734

83 2PX 0.00377 -0.00429 0.00407 -0.00073 -0.01190

84 2PY 0.00285 0.00967 0.01470 -0.00229 -0.00992

85 2PZ -0.00166 -0.00325 -0.00230 -0.01022 0.00525

86 3S 0.00494 0.00440 0.03311 0.00064 -0.01753

87 3PX 0.00581 -0.00713 0.01157 -0.00174 -0.01752

88 3PY 0.00209 0.01406 0.02084 -0.00411 -0.00944

89 3PZ -0.00205 -0.00477 -0.00252 -0.01631 0.00696

90 4S -0.00853 0.02232 0.08058 -0.00318 0.01887

91 4PX 0.01464 -0.01772 -0.03213 0.01077 -0.04893

92 4PY -0.00495 0.02610 0.01839 -0.00506 0.00241

93 4PZ -0.00086 -0.00807 -0.00831 -0.02622 0.00394

94 5D 0 -0.00028 0.00037 0.00125 -0.00145 0.00097

95 5D+1 0.00030 0.00097 0.00089 -0.00130 -0.00074

96 5D-1 0.00004 -0.00051 0.00128 0.00296 0.00022

97 5D+2 0.00083 0.00180 0.00381 -0.00076 -0.00116

98 5D-2 -0.00052 -0.00403 -0.00457 -0.00001 0.00171

99 6 C 1S -0.00233 -0.00430 0.00151 -0.00055 0.00696

100 2S -0.00365 -0.00679 0.00245 -0.00095 0.01090

101 2PX 0.00598 0.01001 -0.00133 -0.00015 -0.01761

102 2PY 0.00085 -0.00160 -0.00078 -0.00137 -0.00306

103 2PZ -0.00251 -0.00660 0.00061 -0.00556 0.00713

104 3S 0.01047 0.01906 -0.00873 0.00337 -0.03104

105 3PX 0.00747 0.01374 -0.00399 -0.00007 -0.02390

106 3PY 0.00175 -0.00183 -0.00224 -0.00207 -0.00588

107 3PZ -0.00294 -0.00980 -0.00103 -0.00868 0.00847

108 4S 0.01214 0.02915 -0.00515 -0.02588 -0.02969

109 4PX 0.00112 0.01528 0.00564 -0.02111 -0.00341

110 4PY 0.00356 -0.00699 -0.01526 0.00853 -0.01392

111 4PZ -0.00297 -0.00099 0.00590 -0.00771 0.00662

112 5D 0 0.00030 0.00067 -0.00106 -0.00173 -0.00080

113 5D+1 0.00031 0.00055 0.00024 0.00170 -0.00090

114 5D-1 0.00010 0.00070 0.00143 0.00081 -0.00015

115 5D+2 -0.00144 -0.00521 -0.00250 0.00168 0.00334

116 5D-2 -0.00077 -0.00356 -0.00221 -0.00101 0.00255

117 7 C 1S 0.00089 0.00234 0.00014 0.00052 -0.00245

118 2S 0.00140 0.00376 0.00019 0.00078 -0.00401

119 2PX -0.00085 0.00083 -0.00159 -0.00886 0.00258

120 2PY -0.00210 -0.00635 -0.00104 0.00265 0.00649

121 2PZ 0.00286 0.00933 -0.00028 -0.00595 -0.00866

122 3S -0.00404 -0.01154 -0.00009 -0.00147 0.01219

123 3PX -0.00104 0.00167 -0.00163 -0.01253 0.00318

124 3PY -0.00324 -0.00871 -0.00043 0.00460 0.00984

125 3PZ 0.00409 0.01346 -0.00048 -0.00933 -0.01201

126 4S -0.00452 -0.01811 -0.00929 -0.00649 0.01009

127 4PX -0.00038 0.00058 -0.00679 -0.01241 -0.00003

128 4PY -0.00250 -0.00951 -0.00104 0.00253 0.00883

129 4PZ 0.00497 0.01341 -0.00118 -0.01081 -0.01310

130 5D 0 -0.00007 0.00001 -0.00017 0.00034 0.00014

131 5D+1 0.00008 -0.00060 0.00008 -0.00041 -0.00009

132 5D-1 0.00024 0.00004 -0.00057 -0.00069 -0.00068

133 5D+2 0.00021 0.00091 -0.00002 -0.00026 -0.00053

134 5D-2 -0.00001 -0.00008 0.00003 -0.00010 -0.00002

135 8 C 1S -0.00017 0.00090 0.00056 0.00010 0.00035

136 2S -0.00026 0.00149 0.00094 0.00018 0.00075

137 2PX -0.00006 -0.00263 -0.00082 -0.00217 0.00019

138 2PY -0.00066 -0.00177 -0.00124 -0.00409 0.00171

139 2PZ 0.00102 -0.00014 0.00029 0.00643 -0.00304

140 3S 0.00063 -0.00479 -0.00381 -0.00068 -0.00291

141 3PX -0.00052 -0.00331 -0.00091 -0.00321 0.00103

142 3PY -0.00094 -0.00301 -0.00297 -0.00619 0.00212

143 3PZ 0.00141 -0.00032 0.00044 0.00923 -0.00436

144 4S 0.00103 0.00220 0.00121 -0.00220 -0.00283

145 4PX -0.00109 0.00072 0.00638 -0.00519 0.00455

146 4PY -0.00029 -0.00265 0.00344 -0.00826 0.00236

147 4PZ 0.00157 0.00252 0.00384 0.01097 -0.00356

148 5D 0 0.00002 0.00012 -0.00043 -0.00046 -0.00028

149 5D+1 -0.00008 -0.00004 -0.00018 0.00001 0.00034

150 5D-1 0.00001 -0.00011 -0.00022 -0.00044 0.00003

151 5D+2 -0.00012 0.00003 -0.00023 -0.00011 0.00040

152 5D-2 -0.00003 0.00023 -0.00058 -0.00010 0.00021

153 9 C 1S 0.00072 0.00167 0.00244 -0.00051 -0.00251

154 2S 0.00117 0.00271 0.00389 -0.00082 -0.00382

155 2PX -0.00135 -0.00215 -0.00597 0.00035 0.00427

156 2PY -0.00183 -0.00424 -0.00607 0.00112 0.00578

157 2PZ -0.00056 -0.00039 -0.00123 -0.00077 0.00190

158 3S -0.00371 -0.00866 -0.01138 0.00289 0.01119

159 3PX -0.00183 -0.00299 -0.00738 0.00059 0.00566

160 3PY -0.00289 -0.00692 -0.00921 0.00169 0.00815

161 3PZ -0.00084 -0.00055 -0.00142 -0.00108 0.00297

162 4S 0.00577 -0.01664 -0.05110 0.00812 -0.02376

163 4PX -0.00106 0.00316 -0.01671 0.00024 -0.00084

164 4PY 0.00334 -0.01130 -0.02023 0.00240 -0.00783

165 4PZ -0.00124 0.00059 -0.00259 0.00421 0.00291

166 5D 0 0.00002 -0.00031 -0.00034 -0.00035 -0.00007

167 5D+1 -0.00011 -0.00037 -0.00042 -0.00158 0.00043

168 5D-1 -0.00006 -0.00034 -0.00022 -0.00039 0.00021

169 5D+2 0.00048 -0.00150 -0.00156 0.00095 -0.00133

170 5D-2 -0.00036 0.00035 0.00101 0.00054 0.00105

171 10 H 1S 0.00932 -0.01016 -0.00297 -0.00114 -0.03044

172 2S 0.01851 -0.01876 -0.01731 0.00120 -0.05868

173 3S 0.00974 -0.01665 -0.01128 0.00023 -0.02870

174 4PX 0.00066 -0.00155 0.00042 -0.00085 0.00018

175 4PY -0.00081 0.00144 -0.00210 0.00127 0.00260

176 4PZ 0.00025 -0.00114 0.00110 0.00365 -0.00046

177 11 H 1S 0.00233 0.00640 0.00077 -0.00083 -0.00729

178 2S 0.00285 0.01101 0.00266 -0.00383 -0.00917

179 3S -0.00627 0.01005 0.02424 -0.00693 0.01821

180 4PX 0.00004 0.00046 0.00078 0.00017 -0.00028

181 4PY 0.00000 0.00006 0.00063 -0.00007 0.00033

182 4PZ -0.00009 -0.00045 -0.00036 -0.00057 0.00028

183 12 H 1S 0.00110 0.00312 0.00030 0.00081 -0.00321

184 2S 0.00127 0.00607 0.00200 0.00306 -0.00403

185 3S 0.00088 0.00425 -0.00051 0.00271 -0.00279

186 4PX 0.00008 0.00056 0.00022 -0.00066 -0.00025

187 4PY -0.00023 -0.00060 -0.00012 0.00028 0.00063

188 4PZ 0.00001 0.00035 -0.00028 -0.00083 -0.00004

189 13 H 1S -0.00028 0.00119 -0.00046 -0.00055 0.00090

190 2S -0.00084 0.00241 0.00154 -0.00153 0.00318

191 3S -0.00093 0.00171 0.00232 0.00078 0.00372

192 4PX -0.00005 0.00000 0.00009 -0.00039 0.00010

193 4PY -0.00010 0.00003 -0.00003 -0.00048 0.00019

194 4PZ 0.00004 0.00018 0.00026 0.00060 -0.00012

195 14 H 1S 0.00015 0.00342 0.00347 -0.00163 -0.00061

196 2S 0.00085 0.00463 0.00785 -0.00371 -0.00136

197 3S -0.00021 0.00575 0.00913 -0.00431 0.00192

198 4PX -0.00009 -0.00031 -0.00047 -0.00009 0.00029

199 4PY 0.00003 -0.00029 -0.00005 0.00002 -0.00004

200 4PZ -0.00008 -0.00004 -0.00013 -0.00010 0.00026

51 52 53 54 55

51 3PX 0.20876

52 3PY 0.00309 0.26180

53 3PZ -0.00363 -0.00772 0.21196

54 4S -0.02691 0.13533 -0.01960 0.42400

55 4PX 0.11395 0.00859 -0.01066 -0.01831 0.06413

56 4PY 0.02480 0.24202 -0.00813 0.16383 0.01900

57 4PZ -0.01775 -0.00726 0.17538 -0.02021 -0.01720

58 5D 0 0.00018 0.01263 0.00424 0.00194 0.00033

59 5D+1 0.00235 -0.00130 0.00377 0.00103 0.00104

60 5D-1 -0.00103 0.00341 -0.01766 0.00176 0.00027

61 5D+2 -0.00276 0.01360 -0.00066 0.01026 -0.00108

62 5D-2 -0.02201 0.00302 -0.00023 0.00065 -0.01152

63 4 C 1S 0.02488 0.01439 0.00247 -0.00854 0.01229

64 2S 0.03883 0.02252 0.00419 -0.00959 0.01834

65 2PX -0.07497 -0.04269 -0.00589 0.03289 -0.04329

66 2PY -0.05608 -0.02740 -0.00040 0.01785 -0.03432

67 2PZ -0.00222 0.00471 0.03633 -0.00154 -0.00149

68 3S -0.10657 -0.06290 -0.01507 0.00935 -0.04745

69 3PX -0.11406 -0.07068 -0.00352 0.04784 -0.06623

70 3PY -0.08514 -0.04745 -0.00128 0.02504 -0.05176

71 3PZ -0.00364 0.00684 0.05431 -0.00233 -0.00247

72 4S -0.12435 -0.07283 0.03136 -0.05575 -0.06210

73 4PX -0.05659 0.00163 -0.04410 0.02167 -0.02859

74 4PY -0.02091 0.02451 0.01043 0.02575 -0.01404

75 4PZ -0.01739 -0.00149 0.05992 -0.00473 -0.01158

76 5D 0 0.00411 0.00387 0.00121 -0.00447 0.00235

77 5D+1 -0.00330 -0.00118 0.01419 0.00083 -0.00264

78 5D-1 -0.00193 -0.00170 0.00783 -0.00138 -0.00141

79 5D+2 0.00022 -0.02172 -0.00040 -0.00642 -0.00100

80 5D-2 -0.01313 0.00126 -0.00072 0.02118 -0.00773

81 5 C 1S -0.00154 -0.00889 -0.00036 -0.00377 -0.00166

82 2S -0.00276 -0.01450 -0.00057 -0.00505 -0.00243

83 2PX -0.00515 0.00588 -0.00085 -0.01103 -0.00508

84 2PY 0.01421 0.02033 -0.00328 -0.00172 0.01308

85 2PZ -0.00442 -0.00344 -0.01533 0.00603 -0.00262

86 3S 0.00808 0.04533 0.00197 0.01367 0.00592

87 3PX -0.00855 0.01646 -0.00210 -0.01292 -0.00811

88 3PY 0.02054 0.02882 -0.00597 0.00098 0.01899

89 3PZ -0.00643 -0.00387 -0.02441 0.00884 -0.00362

90 4S 0.03533 0.11364 -0.00346 0.04976 0.01997

91 4PX -0.02511 -0.04517 0.01591 -0.05364 -0.01861

92 4PY 0.03895 0.02445 -0.00722 0.00700 0.02563

93 4PZ -0.01127 -0.01216 -0.03953 0.00736 -0.00606

94 5D 0 0.00051 0.00189 -0.00214 0.00066 0.00053

95 5D+1 0.00151 0.00129 -0.00172 -0.00017 0.00084

96 5D-1 -0.00073 0.00181 0.00450 0.00059 -0.00082

97 5D+2 0.00297 0.00536 -0.00105 0.00052 0.00072

98 5D-2 -0.00595 -0.00683 0.00000 0.00101 -0.00388

99 6 C 1S -0.00566 0.00214 -0.00090 0.00770 -0.00346

100 2S -0.00933 0.00326 -0.00151 0.01286 -0.00557

101 2PX 0.01387 -0.00104 0.00014 -0.02583 0.01202

102 2PY -0.00268 -0.00182 -0.00234 -0.00452 0.00098

103 2PZ -0.01002 0.00003 -0.00782 0.00891 -0.00494

104 3S 0.02747 -0.01110 0.00518 -0.03958 0.01576

105 3PX 0.01894 -0.00456 0.00041 -0.03654 0.01671

106 3PY -0.00318 -0.00418 -0.00355 -0.00817 0.00173

107 3PZ -0.01493 -0.00270 -0.01225 0.01040 -0.00721

108 4S 0.04279 -0.00662 -0.03884 -0.03733 0.02605

109 4PX 0.02197 0.00807 -0.03099 -0.00998 0.01638

110 4PY -0.01094 -0.02224 0.01268 -0.01797 -0.00439

111 4PZ -0.00212 0.00748 -0.01075 0.00710 0.00145

112 5D 0 0.00098 -0.00148 -0.00258 -0.00147 0.00062

113 5D+1 0.00082 0.00026 0.00254 -0.00048 0.00043

114 5D-1 0.00107 0.00213 0.00120 0.00024 0.00059

115 5D+2 -0.00769 -0.00351 0.00244 0.00276 -0.00477

116 5D-2 -0.00517 -0.00324 -0.00154 0.00265 -0.00370

117 7 C 1S 0.00346 0.00038 0.00077 -0.00611 0.00312

118 2S 0.00545 0.00048 0.00110 -0.00824 0.00468

119 2PX 0.00097 -0.00257 -0.01260 0.00146 0.00037

120 2PY -0.00898 -0.00146 0.00361 0.00935 -0.00709

121 2PZ 0.01309 -0.00026 -0.00863 -0.01504 0.01170

122 3S -0.01638 -0.00048 -0.00178 0.01941 -0.01309

123 3PX 0.00207 -0.00276 -0.01776 0.00148 0.00117

124 3PY -0.01228 -0.00058 0.00638 0.01413 -0.00977

125 3PZ 0.01889 -0.00048 -0.01356 -0.02122 0.01677

126 4S -0.02647 -0.01389 -0.00936 0.01251 -0.01702

127 4PX 0.00023 -0.01010 -0.01775 -0.00495 0.00149

128 4PY -0.01342 -0.00130 0.00322 0.01284 -0.01022

129 4PZ 0.01892 -0.00145 -0.01560 -0.02133 0.01583

130 5D 0 0.00007 -0.00019 0.00048 0.00018 -0.00016

131 5D+1 -0.00094 0.00003 -0.00059 -0.00015 -0.00024

132 5D-1 0.00001 -0.00081 -0.00101 -0.00114 0.00025

133 5D+2 0.00132 0.00004 -0.00035 -0.00086 0.00081

134 5D-2 -0.00014 0.00000 -0.00009 0.00009 -0.00008

135 8 C 1S 0.00165 0.00077 0.00021 0.00189 -0.00041

136 2S 0.00247 0.00130 0.00033 0.00218 0.00029

137 2PX -0.00325 -0.00119 -0.00316 0.00207 -0.00516

138 2PY -0.00249 -0.00223 -0.00595 0.00514 -0.00270

139 2PZ -0.00039 0.00065 0.00957 -0.00577 0.00023

140 3S -0.00707 -0.00537 -0.00108 -0.00415 -0.00414

141 3PX -0.00404 -0.00133 -0.00470 0.00372 -0.00687

142 3PY -0.00425 -0.00493 -0.00901 0.00699 -0.00445

143 3PZ -0.00076 0.00097 0.01373 -0.00840 0.00045

144 4S 0.00335 0.00179 -0.00296 -0.00414 0.00197

145 4PX 0.00193 0.00909 -0.00750 0.00879 -0.00234

146 4PY -0.00365 0.00422 -0.01199 0.00864 -0.00307

147 4PZ 0.00328 0.00594 0.01656 -0.00750 0.00333

148 5D 0 0.00013 -0.00062 -0.00070 -0.00066 0.00031

149 5D+1 -0.00010 -0.00024 -0.00004 0.00000 0.00020

150 5D-1 -0.00017 -0.00034 -0.00062 0.00014 -0.00012

151 5D+2 0.00003 -0.00033 -0.00017 0.00038 -0.00007

152 5D-2 0.00029 -0.00083 -0.00014 0.00018 0.00016

153 9 C 1S 0.00283 0.00364 -0.00070 -0.00012 0.00124

154 2S 0.00436 0.00574 -0.00116 -0.00092 0.00236

155 2PX -0.00364 -0.00880 0.00059 0.00405 -0.00169

156 2PY -0.00624 -0.00882 0.00159 0.00950 -0.00668

157 2PZ -0.00079 -0.00166 -0.00161 -0.00032 0.00155

158 3S -0.01306 -0.01649 0.00419 0.00573 -0.00875

159 3PX -0.00506 -0.01092 0.00100 0.00595 -0.00228

160 3PY -0.01019 -0.01334 0.00237 0.01344 -0.01020

161 3PZ -0.00114 -0.00190 -0.00227 -0.00020 0.00236

162 4S -0.02504 -0.07359 0.01138 -0.03788 -0.01554

163 4PX 0.00426 -0.02431 0.00047 -0.00664 0.00223

164 4PY -0.01680 -0.02887 0.00315 -0.00783 -0.01235

165 4PZ 0.00059 -0.00344 0.00538 -0.00121 0.00228

166 5D 0 -0.00044 -0.00049 -0.00052 -0.00009 -0.00031

167 5D+1 -0.00047 -0.00062 -0.00237 0.00077 -0.00044

168 5D-1 -0.00050 -0.00032 -0.00060 0.00021 -0.00024

169 5D+2 -0.00211 -0.00211 0.00140 -0.00188 -0.00190

170 5D-2 0.00046 0.00148 0.00080 0.00159 0.00047

171 10 H 1S -0.01546 -0.00239 -0.00212 -0.02207 -0.00806

172 2S -0.02824 -0.02200 0.00107 -0.04803 -0.01578

173 3S -0.02476 -0.01470 -0.00007 -0.02370 -0.01366

174 4PX -0.00220 0.00043 -0.00125 0.00056 -0.00108

175 4PY 0.00208 -0.00292 0.00188 0.00041 0.00102

176 4PZ -0.00169 0.00162 0.00560 0.00000 -0.00103

177 11 H 1S 0.00918 0.00071 -0.00148 -0.00879 0.00704

178 2S 0.01584 0.00318 -0.00608 -0.01091 0.01199

179 3S 0.01483 0.03366 -0.01016 0.02246 0.01087

180 4PX 0.00062 0.00113 0.00029 -0.00063 0.00066

181 4PY 0.00011 0.00090 -0.00006 0.00070 0.00005

182 4PZ -0.00070 -0.00057 -0.00084 0.00015 -0.00027

183 12 H 1S 0.00456 0.00069 0.00105 -0.00473 0.00391

184 2S 0.00894 0.00327 0.00435 -0.00585 0.00667

185 3S 0.00627 -0.00043 0.00379 -0.00441 0.00398

186 4PX 0.00078 0.00031 -0.00094 -0.00051 0.00072

187 4PY -0.00084 -0.00016 0.00038 0.00090 -0.00071

188 4PZ 0.00049 -0.00041 -0.00120 -0.00038 0.00030

189 13 H 1S 0.00141 -0.00058 -0.00094 -0.00045 0.00287

190 2S 0.00306 0.00229 -0.00240 0.00210 0.00478

191 3S 0.00235 0.00339 0.00112 0.00361 0.00234

192 4PX 0.00000 0.00012 -0.00058 0.00022 0.00006

193 4PY 0.00004 -0.00008 -0.00072 0.00040 0.00008

194 4PZ 0.00021 0.00040 0.00089 -0.00043 0.00042

195 14 H 1S 0.00484 0.00488 -0.00240 -0.00424 0.00571

196 2S 0.00659 0.01109 -0.00547 -0.00436 0.00803

197 3S 0.00828 0.01280 -0.00627 0.00242 0.00756

198 4PX -0.00049 -0.00068 -0.00013 0.00044 -0.00028

199 4PY -0.00045 -0.00004 0.00002 -0.00054 -0.00002

200 4PZ -0.00007 -0.00018 -0.00017 0.00025 0.00004

56 57 58 59 60

56 4PY 0.23568

57 4PZ -0.00997 0.15115

58 5D 0 0.01119 0.00327 0.00087

59 5D+1 -0.00115 0.00250 0.00012 0.00071

60 5D-1 0.00272 -0.01397 -0.00021 -0.00032 0.00160

61 5D+2 0.01109 -0.00028 0.00052 -0.00003 0.00028

62 5D-2 -0.00001 0.00133 0.00017 -0.00026 0.00023

63 4 C 1S 0.01601 0.00157 0.00192 -0.00019 -0.00030

64 2S 0.02451 0.00208 0.00264 -0.00026 -0.00047

65 2PX -0.03896 0.00045 -0.00269 -0.00068 0.00049

66 2PY -0.03520 0.00261 -0.00165 -0.00033 -0.00014

67 2PZ 0.00659 0.02945 -0.00042 -0.00540 -0.00385

68 3S -0.06723 -0.00633 -0.00615 0.00058 0.00159

69 3PX -0.06481 0.00512 -0.00437 -0.00099 0.00019

70 3PY -0.05827 0.00330 -0.00283 -0.00050 -0.00019

71 3PZ 0.00970 0.04365 -0.00067 -0.00839 -0.00585

72 4S -0.09688 0.03844 -0.00332 0.00155 -0.00134

73 4PX -0.00288 -0.03249 -0.00049 -0.00137 0.00434

74 4PY 0.01874 0.01101 0.00102 -0.00034 -0.00061

75 4PZ -0.00047 0.05341 -0.00082 -0.00758 -0.00571

76 5D 0 0.00322 0.00053 0.00031 -0.00029 -0.00013

77 5D+1 -0.00096 0.01209 0.00023 0.00028 -0.00121

78 5D-1 -0.00215 0.00661 0.00013 0.00038 -0.00055

79 5D+2 -0.01996 -0.00033 -0.00105 0.00024 -0.00024

80 5D-2 0.00176 0.00016 0.00004 -0.00007 0.00009

81 5 C 1S -0.00724 -0.00038 0.00004 0.00000 -0.00009

82 2S -0.01242 -0.00052 -0.00004 0.00000 -0.00011

83 2PX 0.00199 -0.00051 -0.00064 0.00060 0.00016

84 2PY 0.02713 -0.00410 0.00010 -0.00172 0.00016

85 2PZ -0.00421 -0.01457 -0.00119 -0.00395 -0.00001

86 3S 0.04085 0.00178 0.00065 -0.00007 0.00027

87 3PX 0.00936 -0.00137 -0.00070 0.00091 0.00037

88 3PY 0.03878 -0.00690 0.00011 -0.00243 0.00034

89 3PZ -0.00525 -0.02340 -0.00177 -0.00604 0.00005

90 4S 0.10486 -0.00366 0.00395 0.00140 0.00147

91 4PX -0.05076 0.01586 -0.00311 0.00077 -0.00138

92 4PY 0.04121 -0.01079 -0.00004 -0.00284 -0.00021

93 4PZ -0.01352 -0.03481 -0.00256 -0.00670 0.00129

94 5D 0 0.00142 -0.00212 0.00020 0.00024 0.00022

95 5D+1 0.00084 -0.00313 0.00008 0.00011 -0.00003

96 5D-1 0.00175 0.00350 0.00008 -0.00052 -0.00045

97 5D+2 0.00431 -0.00094 0.00024 0.00015 0.00009

98 5D-2 -0.00527 -0.00009 -0.00024 -0.00011 -0.00016

99 6 C 1S 0.00325 -0.00023 0.00033 0.00005 0.00020

100 2S 0.00497 -0.00033 0.00043 0.00006 0.00029

101 2PX -0.00427 -0.00397 -0.00094 -0.00158 -0.00035

102 2PY 0.00034 -0.00185 -0.00060 -0.00055 0.00035

103 2PZ 0.00198 -0.00945 -0.00021 -0.00202 0.00040

104 3S -0.01565 0.00135 -0.00101 -0.00002 -0.00082

105 3PX -0.00857 -0.00571 -0.00158 -0.00245 -0.00060

106 3PY -0.00096 -0.00272 -0.00093 -0.00092 0.00051

107 3PZ 0.00030 -0.01479 -0.00050 -0.00310 0.00059

108 4S -0.00689 -0.03627 -0.00061 0.00143 0.00321

109 4PX 0.00788 -0.03238 -0.00080 -0.00171 0.00220

110 4PY -0.02013 0.00908 -0.00140 -0.00085 -0.00131

111 4PZ 0.00964 -0.01418 -0.00016 -0.00251 0.00069

112 5D 0 -0.00153 -0.00242 0.00000 0.00035 0.00024

113 5D+1 0.00098 0.00201 -0.00006 -0.00050 -0.00028

114 5D-1 0.00176 0.00128 0.00013 0.00017 -0.00005

115 5D+2 -0.00438 0.00301 -0.00020 -0.00010 -0.00023

116 5D-2 -0.00329 -0.00083 -0.00007 0.00018 0.00015

117 7 C 1S 0.00085 0.00033 -0.00005 -0.00013 -0.00004

118 2S 0.00092 0.00058 -0.00013 -0.00019 -0.00007

119 2PX -0.00192 -0.01451 0.00019 0.00058 0.00095

120 2PY -0.00235 0.00641 0.00030 0.00036 -0.00024

121 2PZ -0.00035 -0.01109 -0.00029 0.00022 0.00066

122 3S -0.00091 -0.00104 0.00058 0.00068 0.00018

123 3PX -0.00195 -0.02098 0.00027 0.00074 0.00130

124 3PY -0.00184 0.01027 0.00052 0.00051 -0.00043

125 3PZ -0.00062 -0.01691 -0.00041 0.00030 0.00104

126 4S -0.01357 -0.00818 -0.00085 -0.00041 0.00062

127 4PX -0.00908 -0.02072 -0.00022 0.00109 0.00138

128 4PY -0.00365 0.00790 0.00032 0.00043 -0.00021

129 4PZ -0.00172 -0.01928 0.00003 0.00166 0.00132

130 5D 0 -0.00030 0.00065 0.00001 0.00012 -0.00004

131 5D+1 0.00019 -0.00073 -0.00003 -0.00009 0.00006

132 5D-1 -0.00084 -0.00120 -0.00012 -0.00018 0.00005

133 5D+2 -0.00015 -0.00063 -0.00002 -0.00015 -0.00003

134 5D-2 0.00016 -0.00036 0.00002 -0.00002 0.00000

135 8 C 1S 0.00118 -0.00019 0.00013 0.00008 0.00000

136 2S 0.00201 -0.00008 0.00022 0.00013 -0.00001

137 2PX -0.00258 -0.00305 -0.00047 -0.00097 0.00003

138 2PY -0.00067 -0.00659 -0.00062 -0.00203 0.00015

139 2PZ -0.00053 0.00935 0.00059 0.00250 -0.00023

140 3S -0.00752 -0.00039 -0.00074 -0.00050 0.00004

141 3PX -0.00319 -0.00459 -0.00066 -0.00144 0.00005

142 3PY -0.00262 -0.00983 -0.00099 -0.00295 0.00024

143 3PZ -0.00064 0.01346 0.00085 0.00361 -0.00031

144 4S 0.00066 -0.00521 -0.00041 -0.00004 0.00016

145 4PX 0.00746 -0.00793 -0.00016 -0.00149 0.00024

146 4PY 0.00608 -0.01324 -0.00076 -0.00365 0.00040

147 4PZ 0.00383 0.01465 0.00149 0.00463 -0.00043

148 5D 0 -0.00054 -0.00067 -0.00005 -0.00002 0.00005

149 5D+1 -0.00031 0.00028 0.00003 0.00013 0.00002

150 5D-1 -0.00031 -0.00077 -0.00003 -0.00009 0.00003

151 5D+2 -0.00058 -0.00020 0.00000 -0.00001 0.00001

152 5D-2 -0.00098 -0.00015 0.00003 0.00012 0.00001

153 9 C 1S 0.00278 -0.00090 0.00003 0.00007 0.00009

154 2S 0.00472 -0.00138 0.00004 0.00009 0.00015

155 2PX -0.00775 -0.00008 0.00001 -0.00018 -0.00014

156 2PY -0.01225 0.00242 -0.00003 0.00030 -0.00016

157 2PZ -0.00136 0.00149 0.00005 0.00052 0.00023

158 3S -0.01490 0.00463 -0.00009 -0.00020 -0.00051

159 3PX -0.00984 0.00002 0.00010 -0.00015 -0.00017

160 3PY -0.01863 0.00381 -0.00013 0.00038 -0.00023

161 3PZ -0.00155 0.00207 0.00011 0.00072 0.00028

162 4S -0.06339 0.01185 -0.00337 -0.00167 -0.00186

163 4PX -0.01772 -0.00266 -0.00106 -0.00085 -0.00064

164 4PY -0.03207 0.00574 -0.00094 0.00045 -0.00026

165 4PZ -0.00300 0.01102 0.00031 0.00202 0.00012

166 5D 0 -0.00041 -0.00054 -0.00005 -0.00010 0.00000

167 5D+1 -0.00066 -0.00218 -0.00018 -0.00054 0.00004

168 5D-1 -0.00055 -0.00043 -0.00005 -0.00014 0.00000

169 5D+2 -0.00356 0.00160 -0.00005 0.00045 -0.00006

170 5D-2 0.00091 0.00094 0.00019 0.00028 0.00001

171 10 H 1S -0.01943 0.00193 -0.00099 0.00055 0.00104

172 2S -0.04585 0.00668 -0.00210 0.00078 0.00095

173 3S -0.02698 0.00353 -0.00121 -0.00009 0.00057

174 4PX 0.00141 -0.00097 0.00007 -0.00021 0.00008

175 4PY -0.00214 0.00127 -0.00009 0.00010 -0.00020

176 4PZ 0.00125 0.00416 0.00030 0.00031 -0.00047

177 11 H 1S 0.00275 -0.00174 -0.00038 -0.00023 0.00021

178 2S 0.00663 -0.00598 -0.00053 -0.00057 0.00064

179 3S 0.03714 -0.01013 0.00106 -0.00096 0.00108

180 4PX 0.00093 -0.00004 -0.00001 -0.00010 -0.00003

181 4PY 0.00096 -0.00028 0.00008 -0.00008 -0.00001

182 4PZ -0.00039 -0.00090 -0.00007 -0.00015 0.00005

183 12 H 1S -0.00005 0.00128 -0.00009 0.00005 -0.00009

184 2S 0.00222 0.00431 -0.00003 -0.00007 -0.00039

185 3S -0.00070 0.00424 -0.00015 -0.00027 -0.00036

186 4PX 0.00030 -0.00118 0.00002 0.00007 0.00008

187 4PY -0.00024 0.00063 0.00002 0.00002 -0.00003

188 4PZ -0.00034 -0.00133 -0.00002 0.00004 0.00009

189 13 H 1S -0.00027 -0.00036 0.00010 0.00012 0.00006

190 2S 0.00257 -0.00160 0.00037 0.00021 0.00018

191 3S 0.00348 0.00138 0.00051 0.00061 -0.00003

192 4PX 0.00012 -0.00052 -0.00003 -0.00014 0.00002

193 4PY 0.00014 -0.00073 -0.00006 -0.00022 0.00002

194 4PZ 0.00036 0.00088 0.00008 0.00022 -0.00002

195 14 H 1S 0.00931 -0.00297 0.00005 -0.00065 0.00013

196 2S 0.01652 -0.00575 0.00019 -0.00117 0.00041

197 3S 0.01637 -0.00703 0.00032 -0.00101 0.00051

198 4PX -0.00077 -0.00013 -0.00001 -0.00005 0.00001

199 4PY -0.00002 0.00015 0.00001 0.00004 0.00001

200 4PZ -0.00025 0.00003 0.00001 0.00005 0.00002

61 62 63 64 65

61 5D+2 0.00141

62 5D-2 0.00045 0.00260

63 4 C 1S -0.00047 -0.00373 0.65856

64 2S -0.00069 -0.00549 0.56042 0.48990

65 2PX -0.00234 0.00823 0.00224 0.00315 0.08210

66 2PY 0.00239 0.00249 0.00164 0.00219 -0.00525

67 2PZ 0.00051 0.00017 0.00037 0.00103 0.00047

68 3S 0.00170 0.01415 -0.08597 -0.14916 -0.00791

69 3PX -0.00363 0.01232 -0.00052 0.00004 0.12193

70 3PY 0.00325 0.00400 -0.00141 -0.00126 -0.00476

71 3PZ 0.00076 0.00025 0.00369 0.00503 0.00205

72 4S 0.00348 0.01477 -0.07699 -0.10972 0.01106

73 4PX 0.00057 0.00722 0.00981 0.01235 0.05602

74 4PY 0.00276 0.00054 0.00951 0.01159 -0.01217

75 4PZ 0.00066 0.00137 0.00004 0.00281 0.00963

76 5D 0 0.00013 -0.00039 0.00181 0.00379 -0.00131

77 5D+1 -0.00018 0.00033 0.00000 0.00031 0.00323

78 5D-1 0.00009 0.00005 0.00024 0.00038 -0.00079

79 5D+2 -0.00088 -0.00067 -0.00112 -0.00173 -0.00141

80 5D-2 0.00030 0.00114 -0.00066 -0.00024 0.00775

81 5 C 1S -0.00019 -0.00082 0.00549 0.00877 -0.00402

82 2S -0.00024 -0.00113 0.00875 0.01354 -0.00653

83 2PX 0.00138 0.00057 0.00198 0.00297 0.00675

84 2PY -0.00102 0.00182 -0.01674 -0.02677 0.01092

85 2PZ 0.00074 -0.00049 0.00508 0.00818 -0.00221

86 3S 0.00074 0.00272 -0.02289 -0.03465 0.01856

87 3PX 0.00250 0.00106 0.00332 0.00490 0.01028

88 3PY -0.00133 0.00252 -0.02913 -0.04313 0.01400

89 3PZ 0.00117 -0.00070 0.00828 0.01274 -0.00337

90 4S 0.00547 -0.00058 0.00669 0.00567 0.00007

91 4PX -0.00035 0.00223 -0.00982 -0.01208 0.01945

92 4PY -0.00338 -0.00108 -0.00886 -0.01500 0.01036

93 4PZ 0.00096 0.00003 0.00446 0.00726 0.00175

94 5D 0 0.00021 -0.00003 0.00164 0.00210 -0.00062

95 5D+1 0.00011 -0.00010 -0.00007 -0.00009 -0.00106

96 5D-1 0.00014 -0.00010 0.00234 0.00313 -0.00061

97 5D+2 0.00024 -0.00065 0.00466 0.00619 -0.00378

98 5D-2 -0.00079 0.00033 0.00244 0.00321 0.00582

99 6 C 1S -0.00054 0.00067 0.00559 0.00886 0.01593

100 2S -0.00076 0.00124 0.00886 0.01367 0.02542

101 2PX 0.00204 -0.00196 -0.01472 -0.02356 -0.04276

102 2PY -0.00069 0.00097 0.00886 0.01417 0.02859

103 2PZ -0.00054 0.00155 0.00898 0.01438 0.02717

104 3S 0.00172 -0.00412 -0.02363 -0.03575 -0.07162

105 3PX 0.00273 -0.00272 -0.02609 -0.03834 -0.05975

106 3PY -0.00109 0.00129 0.01508 0.02252 0.04101

107 3PZ -0.00091 0.00228 0.01442 0.02211 0.04063

108 4S -0.00010 -0.00485 0.00210 -0.00074 -0.04219

109 4PX 0.00149 -0.00259 -0.00221 -0.00478 -0.02779

110 4PY -0.00142 0.00168 0.00177 0.00541 0.03249

111 4PZ -0.00002 0.00107 0.00882 0.01365 0.02301

112 5D 0 -0.00012 -0.00012 0.00133 0.00167 0.00046

113 5D+1 -0.00016 0.00000 0.00215 0.00290 0.00341

114 5D-1 0.00017 -0.00012 -0.00205 -0.00269 -0.00363

115 5D+2 0.00024 0.00051 -0.00321 -0.00417 -0.00113

116 5D-2 -0.00020 0.00036 0.00390 0.00514 0.00583

117 7 C 1S 0.00009 -0.00032 -0.00132 -0.00197 -0.00372

118 2S 0.00017 -0.00049 -0.00196 -0.00309 -0.00585

119 2PX -0.00050 0.00007 0.00344 0.00544 0.00547

120 2PY -0.00020 0.00071 0.00083 0.00129 0.00384

121 2PZ 0.00025 -0.00098 -0.00214 -0.00336 -0.00716

122 3S -0.00056 0.00146 0.00537 0.00881 0.01672

123 3PX -0.00062 0.00007 0.00341 0.00612 0.00659

124 3PY -0.00023 0.00100 0.00051 0.00111 0.00498

125 3PZ 0.00035 -0.00144 -0.00209 -0.00383 -0.01044

126 4S -0.00060 0.00241 0.00292 0.00617 0.02747

127 4PX -0.00088 0.00030 0.00400 0.00694 0.01292

128 4PY 0.00014 0.00086 -0.00087 -0.00170 -0.00104

129 4PZ -0.00007 -0.00132 -0.00096 -0.00211 -0.00868

130 5D 0 0.00002 -0.00007 -0.00041 -0.00068 -0.00136

131 5D+1 -0.00005 0.00018 0.00102 0.00151 0.00305

132 5D-1 0.00004 -0.00003 0.00005 -0.00002 -0.00018

133 5D+2 0.00013 -0.00022 -0.00119 -0.00182 -0.00405

134 5D-2 -0.00007 0.00007 -0.00001 0.00033 0.00133

135 8 C 1S -0.00008 -0.00011 -0.00095 -0.00138 -0.00208

136 2S -0.00013 -0.00016 -0.00140 -0.00214 -0.00332

137 2PX 0.00038 -0.00004 0.00158 0.00246 0.00050

138 2PY -0.00024 0.00005 0.00327 0.00524 0.00675

139 2PZ -0.00008 0.00036 -0.00055 -0.00100 0.00209

140 3S 0.00035 0.00044 0.00340 0.00566 0.00978

141 3PX 0.00056 -0.00009 0.00153 0.00268 0.00032

142 3PY -0.00043 0.00007 0.00475 0.00777 0.01036

143 3PZ -0.00013 0.00058 -0.00100 -0.00167 0.00359

144 4S 0.00053 -0.00011 0.00819 0.01071 0.01084

145 4PX 0.00103 -0.00047 0.00417 0.00524 -0.00310

146 4PY 0.00021 0.00030 0.00634 0.00958 0.01063

147 4PZ -0.00004 0.00033 -0.00039 -0.00143 -0.00004

148 5D 0 -0.00002 -0.00001 0.00005 0.00010 0.00032

149 5D+1 -0.00003 0.00001 -0.00004 -0.00008 -0.00017

150 5D-1 -0.00002 -0.00001 0.00044 0.00066 0.00053

151 5D+2 0.00002 -0.00007 0.00019 0.00034 -0.00049

152 5D-2 -0.00006 -0.00011 0.00031 0.00042 -0.00078

153 9 C 1S 0.00022 -0.00002 -0.00130 -0.00198 -0.00001

154 2S 0.00035 0.00004 -0.00196 -0.00312 0.00002

155 2PX -0.00069 -0.00030 0.00284 0.00451 -0.00011

156 2PY -0.00031 -0.00040 0.00371 0.00584 -0.00095

157 2PZ -0.00020 0.00009 -0.00102 -0.00164 -0.00126

158 3S -0.00098 -0.00029 0.00566 0.00931 -0.00025

159 3PX -0.00086 -0.00039 0.00218 0.00441 -0.00128

160 3PY -0.00033 -0.00043 0.00351 0.00648 -0.00093

161 3PZ -0.00026 0.00013 -0.00124 -0.00212 -0.00208

162 4S -0.00550 0.00117 -0.00784 -0.00848 0.02070

163 4PX -0.00243 -0.00082 0.00158 0.00213 0.00612

164 4PY -0.00122 0.00047 0.00049 0.00205 0.00460

165 4PZ -0.00056 -0.00006 -0.00317 -0.00505 -0.00369

166 5D 0 -0.00002 0.00002 0.00022 0.00030 0.00035

167 5D+1 0.00007 -0.00007 0.00051 0.00075 -0.00037

168 5D-1 0.00006 -0.00001 0.00022 0.00042 -0.00016

169 5D+2 0.00020 -0.00006 0.00107 0.00171 -0.00066

170 5D-2 0.00010 -0.00003 -0.00116 -0.00135 -0.00151

171 10 H 1S 0.00498 0.00249 -0.00413 -0.00653 0.01095

172 2S 0.00587 0.00391 -0.00487 -0.00861 0.01974

173 3S 0.00288 0.00336 -0.00303 -0.00550 0.01726

174 4PX -0.00039 0.00028 -0.00031 -0.00042 0.00089

175 4PY -0.00018 -0.00021 -0.00022 -0.00030 0.00003

176 4PZ 0.00002 0.00022 -0.00009 -0.00014 0.00005

177 11 H 1S -0.00050 -0.00040 0.00391 0.00648 0.01018

178 2S -0.00074 -0.00071 0.00568 0.00951 0.01486

179 3S 0.00059 -0.00045 0.00741 0.01091 0.00869

180 4PX 0.00018 -0.00007 -0.00094 -0.00128 -0.00208

181 4PY 0.00000 0.00002 0.00073 0.00069 0.00015

182 4PZ -0.00006 0.00011 0.00055 0.00093 0.00213

183 12 H 1S 0.00030 -0.00052 -0.00245 -0.00391 -0.00708

184 2S 0.00056 -0.00106 -0.00473 -0.00714 -0.01398

185 3S 0.00024 -0.00089 -0.00276 -0.00435 -0.01061

186 4PX 0.00000 -0.00003 -0.00001 0.00005 0.00008

187 4PY -0.00001 0.00006 -0.00002 -0.00002 0.00010

188 4PZ -0.00002 -0.00005 0.00020 0.00018 -0.00036

189 13 H 1S -0.00011 -0.00013 -0.00085 -0.00130 -0.00221

190 2S -0.00005 -0.00028 -0.00112 -0.00187 -0.00516

191 3S -0.00006 -0.00027 -0.00013 -0.00059 -0.00482

192 4PX 0.00003 -0.00001 -0.00020 -0.00025 -0.00023

193 4PY -0.00002 -0.00001 0.00011 0.00019 0.00038

194 4PZ 0.00000 0.00003 -0.00027 -0.00042 -0.00012

195 14 H 1S -0.00022 0.00013 -0.00192 -0.00297 0.00086

196 2S 0.00002 0.00029 -0.00299 -0.00444 0.00032

197 3S 0.00005 0.00013 -0.00129 -0.00208 0.00198

198 4PX -0.00001 0.00001 0.00018 0.00030 0.00017

199 4PY 0.00005 0.00003 -0.00014 -0.00007 -0.00026

200 4PZ -0.00002 0.00000 0.00000 -0.00005 -0.00011

66 67 68 69 70

66 2PY 0.08293

67 2PZ -0.00124 0.07508

68 3S -0.00507 -0.00600 0.45587

69 3PX -0.00456 0.00282 -0.00340 0.18179

70 3PY 0.12177 -0.00154 0.00023 -0.00217 0.17943

71 3PZ -0.00130 0.11632 -0.01428 0.00621 -0.00151

72 4S 0.03305 -0.01113 0.27054 0.02352 0.05411

73 4PX -0.00656 -0.00671 -0.02435 0.08012 -0.00761

74 4PY 0.03787 0.00375 -0.02212 -0.01689 0.05357

75 4PZ 0.00487 0.10178 -0.01852 0.01770 0.00755

76 5D 0 -0.00057 0.00392 -0.01338 -0.00221 -0.00104

77 5D+1 -0.00118 0.00157 -0.00195 0.00524 -0.00184

78 5D-1 0.00425 -0.00198 -0.00115 -0.00105 0.00638

79 5D+2 0.00905 -0.00205 0.00483 -0.00129 0.01383

80 5D-2 0.00439 -0.00048 -0.00198 0.01150 0.00661

81 5 C 1S 0.01846 -0.00238 -0.02298 -0.00724 0.03346

82 2S 0.02960 -0.00383 -0.03475 -0.01067 0.04957

83 2PX 0.01008 -0.00371 -0.00705 0.01095 0.01443

84 2PY -0.06258 0.02234 0.07606 0.01562 -0.08947

85 2PZ 0.01701 0.04391 -0.02344 -0.00164 0.02397

86 3S -0.08327 0.01065 0.08812 0.02687 -0.12720

87 3PX 0.01339 -0.00567 -0.01163 0.01654 0.01850

88 3PY -0.08800 0.03145 0.11193 0.01994 -0.12560

89 3PZ 0.02491 0.06730 -0.03474 -0.00253 0.03498

90 4S -0.06558 -0.00497 -0.00289 -0.00290 -0.10296

91 4PX 0.02005 -0.00075 0.02351 0.03221 0.03036

92 4PY -0.07566 0.03378 0.04467 0.01428 -0.10935

93 4PZ 0.02054 0.06755 -0.02104 0.00518 0.02909

94 5D 0 0.00143 -0.00329 -0.00416 -0.00116 0.00222

95 5D+1 -0.00023 0.00000 0.00012 -0.00152 -0.00046

96 5D-1 0.00297 0.00704 -0.00722 -0.00072 0.00426

97 5D+2 0.00479 -0.00232 -0.01367 -0.00557 0.00612

98 5D-2 0.00176 0.00020 -0.00714 0.00864 0.00262

99 6 C 1S -0.00832 -0.00215 -0.02355 0.02709 -0.01445

100 2S -0.01335 -0.00347 -0.03560 0.04030 -0.02153

101 2PX 0.02679 0.02256 0.06635 -0.06085 0.03981

102 2PY -0.00770 0.00290 -0.04054 0.04135 -0.01073

103 2PZ -0.00832 0.02376 -0.04157 0.03846 -0.01099

104 3S 0.03883 0.00956 0.09230 -0.10448 0.05748

105 3PX 0.03809 0.03499 0.09747 -0.08473 0.05674

106 3PY -0.01102 0.00535 -0.05942 0.05932 -0.01530

107 3PZ -0.01135 0.03653 -0.06088 0.05763 -0.01475

108 4S 0.01518 -0.02528 0.01411 -0.06451 0.02262

109 4PX 0.01735 0.01425 0.01689 -0.04147 0.02608

110 4PY -0.00483 0.01465 -0.02404 0.04825 -0.00559

111 4PZ -0.00937 0.03036 -0.03854 0.03180 -0.01209

112 5D 0 0.00047 -0.00449 -0.00315 0.00051 0.00068

113 5D+1 -0.00291 0.00640 -0.00668 0.00504 -0.00420

114 5D-1 0.00014 -0.00194 0.00594 -0.00517 0.00002

115 5D+2 0.00671 0.00121 0.00890 -0.00097 0.00979

116 5D-2 0.00237 -0.00330 -0.01114 0.00838 0.00354

117 7 C 1S 0.00082 0.00145 0.00524 -0.00376 0.00025

118 2S 0.00123 0.00231 0.00875 -0.00678 0.00093

119 2PX -0.00172 -0.00476 -0.01590 0.00600 -0.00145

120 2PY -0.00089 -0.00460 -0.00312 0.00636 -0.00239

121 2PZ 0.00250 -0.00071 0.00981 -0.01051 0.00386

122 3S -0.00317 -0.00850 -0.02604 0.02226 -0.00419

123 3PX -0.00227 -0.00516 -0.02037 0.00685 -0.00181

124 3PY -0.00221 -0.00610 -0.00346 0.00840 -0.00490

125 3PZ 0.00389 -0.00114 0.01300 -0.01538 0.00596

126 4S 0.00534 0.00058 -0.02241 0.03904 0.00945

127 4PX -0.00035 -0.00996 -0.02188 0.01653 0.00125

128 4PY 0.00353 -0.00709 0.00663 0.00018 0.00327

129 4PZ 0.00064 -0.01617 0.00828 -0.01381 0.00147

130 5D 0 0.00070 -0.00163 0.00205 -0.00184 0.00091

131 5D+1 -0.00076 0.00089 -0.00392 0.00435 -0.00096

132 5D-1 0.00129 0.00180 0.00035 -0.00021 0.00200

133 5D+2 0.00180 0.00212 0.00487 -0.00578 0.00260

134 5D-2 -0.00086 0.00061 -0.00207 0.00174 -0.00113

135 8 C 1S -0.00159 -0.00090 0.00334 -0.00279 -0.00160

136 2S -0.00245 -0.00148 0.00565 -0.00471 -0.00298

137 2PX 0.00586 0.00922 -0.00704 0.00248 0.00761

138 2PY 0.00005 0.01853 -0.01608 0.00946 0.00089

139 2PZ -0.00201 -0.02339 0.00380 0.00241 -0.00282

140 3S 0.00750 0.00557 -0.01650 0.01485 0.01104

141 3PX 0.00759 0.01387 -0.00870 0.00293 0.00973

142 3PY 0.00094 0.02666 -0.02439 0.01453 0.00261

143 3PZ -0.00336 -0.03374 0.00579 0.00423 -0.00466

144 4S -0.00013 0.00249 -0.02333 0.01518 0.00069

145 4PX 0.00349 0.01466 -0.01087 -0.00320 0.00408

146 4PY -0.00066 0.03400 -0.02703 0.01509 0.00005

147 4PZ -0.00599 -0.04300 0.00733 -0.00173 -0.00861

148 5D 0 0.00032 0.00018 -0.00031 0.00040 0.00057

149 5D+1 0.00002 -0.00126 0.00038 -0.00029 -0.00004

150 5D-1 0.00038 0.00080 -0.00185 0.00067 0.00064

151 5D+2 0.00091 0.00009 -0.00114 -0.00059 0.00114

152 5D-2 0.00079 -0.00127 -0.00103 -0.00127 0.00112

153 9 C 1S -0.00398 0.00035 0.00564 -0.00023 -0.00353

154 2S -0.00631 0.00057 0.00931 -0.00027 -0.00673

155 2PX 0.00651 -0.00027 -0.01389 -0.00131 0.00933

156 2PY 0.00944 -0.00428 -0.01734 -0.00067 0.01220

157 2PZ -0.00037 -0.00521 0.00553 -0.00225 -0.00076

158 3S 0.01943 -0.00184 -0.02848 0.00039 0.02487

159 3PX 0.00902 -0.00145 -0.01708 -0.00349 0.01280

160 3PY 0.01420 -0.00552 -0.02256 -0.00020 0.01843

161 3PZ -0.00044 -0.00673 0.00765 -0.00363 -0.00101

162 4S 0.01286 0.01493 0.01110 0.03354 0.01949

163 4PX -0.00316 0.00913 -0.00603 0.00846 -0.00339

164 4PY 0.01776 -0.00756 -0.01016 0.00818 0.02521

165 4PZ -0.00310 -0.02162 0.01575 -0.00582 -0.00511

166 5D 0 0.00059 0.00093 -0.00066 0.00057 0.00098

167 5D+1 0.00171 0.00545 -0.00194 -0.00028 0.00236

168 5D-1 0.00131 0.00176 -0.00140 -0.00016 0.00184

169 5D+2 0.00617 -0.00443 -0.00473 -0.00070 0.00869

170 5D-2 -0.00079 -0.00277 0.00219 -0.00233 -0.00147

171 10 H 1S 0.00601 0.00043 0.01869 0.01663 0.00931

172 2S 0.01275 0.00091 0.02767 0.03051 0.01975

173 3S 0.00737 0.00391 0.01801 0.02652 0.01197

174 4PX -0.00090 0.00058 0.00096 0.00130 -0.00126

175 4PY -0.00046 0.00001 0.00065 0.00018 -0.00054

176 4PZ -0.00011 -0.00111 0.00032 0.00012 -0.00020

177 11 H 1S -0.00689 0.00113 -0.01985 0.01402 -0.00965

178 2S -0.01333 0.00319 -0.02936 0.02019 -0.01881

179 3S -0.02097 0.00671 -0.02868 0.01053 -0.03088

180 4PX 0.00151 0.00148 0.00288 -0.00299 0.00223

181 4PY -0.00120 0.00111 -0.00042 0.00020 -0.00177

182 4PZ -0.00027 0.00158 -0.00286 0.00303 -0.00027

183 12 H 1S 0.00274 -0.00072 0.01178 -0.00956 0.00334

184 2S 0.00371 0.00162 0.01982 -0.01912 0.00422

185 3S 0.00343 0.00339 0.01252 -0.01452 0.00432

186 4PX -0.00030 -0.00051 -0.00033 -0.00001 -0.00041

187 4PY 0.00006 -0.00027 0.00003 0.00022 0.00001

188 4PZ 0.00032 -0.00044 -0.00016 -0.00061 0.00056

189 13 H 1S -0.00045 -0.00113 0.00387 -0.00371 -0.00056

190 2S -0.00160 -0.00214 0.00596 -0.00843 -0.00238

191 3S -0.00133 -0.00624 0.00310 -0.00756 -0.00226

192 4PX 0.00005 0.00134 0.00047 -0.00027 0.00004

193 4PY -0.00024 0.00203 -0.00070 0.00050 -0.00026

194 4PZ -0.00053 -0.00203 0.00123 -0.00029 -0.00074

195 14 H 1S -0.00810 0.00638 0.00851 0.00035 -0.01111

196 2S -0.01194 0.01070 0.01203 -0.00096 -0.01651

197 3S -0.01496 0.00944 0.00593 0.00155 -0.02081

198 4PX 0.00053 0.00030 -0.00095 0.00019 0.00078

199 4PY 0.00104 -0.00049 -0.00022 -0.00044 0.00143

200 4PZ -0.00011 -0.00050 0.00037 -0.00017 -0.00018

71 72 73 74 75

71 3PZ 0.18036

72 4S -0.02006 0.26718

73 4PX -0.00887 0.00084 0.06195

74 4PY 0.00599 -0.00661 -0.01287 0.02910

75 4PZ 0.15742 -0.00460 -0.00542 0.00680 0.14713

76 5D 0 0.00625 -0.00714 0.00039 0.00050 0.00479

77 5D+1 0.00231 0.00362 -0.00172 0.00020 0.00430

78 5D-1 -0.00316 0.00095 -0.00115 0.00160 -0.00207

79 5D+2 -0.00312 0.00721 -0.00375 0.00136 -0.00237

80 5D-2 -0.00058 0.00438 0.00679 0.00123 0.00081

81 5 C 1S -0.00375 0.00282 0.00084 0.00111 -0.00117

82 2S -0.00575 0.00204 0.00048 0.00430 -0.00194

83 2PX -0.00531 -0.01619 -0.00689 0.01143 -0.00576

84 2PY 0.03361 0.01325 0.00470 -0.03095 0.02369

85 2PZ 0.06919 -0.00286 -0.00345 0.01290 0.05707

86 3S 0.01520 0.00122 0.00213 -0.01931 0.00589

87 3PX -0.00809 -0.02688 -0.01056 0.01863 -0.00918

88 3PY 0.04725 0.02210 0.00637 -0.04400 0.03275

89 3PZ 0.10607 -0.00500 -0.00513 0.01944 0.08692

90 4S -0.00799 -0.05723 -0.00402 0.00443 -0.01987

91 4PX -0.00103 0.01184 -0.00821 0.01107 0.00273

92 4PY 0.05151 -0.04112 -0.00171 -0.03365 0.03734

93 4PZ 0.10638 0.00881 0.00017 0.01311 0.09129

94 5D 0 -0.00500 -0.00013 0.00132 0.00007 -0.00460

95 5D+1 0.00011 -0.00126 -0.00119 -0.00039 -0.00041

96 5D-1 0.01096 -0.00509 -0.00115 0.00204 0.01036

97 5D+2 -0.00345 -0.01154 -0.00451 0.00599 -0.00349

98 5D-2 0.00047 -0.00352 0.00380 -0.00024 0.00139

99 6 C 1S -0.00397 -0.00072 0.00894 -0.00525 0.00216

100 2S -0.00568 -0.00329 0.01473 -0.00800 0.00249

101 2PX 0.03379 0.03033 -0.03392 0.01212 0.02437

102 2PY 0.00491 -0.03202 0.02144 -0.00463 0.00962

103 2PZ 0.03839 -0.04435 0.03033 -0.00528 0.02244

104 3S 0.01356 0.01631 -0.04376 0.02198 -0.00449

105 3PX 0.05256 0.04383 -0.04824 0.01699 0.03816

106 3PY 0.00891 -0.04655 0.03096 -0.00690 0.01537

107 3PZ 0.05898 -0.06572 0.04495 -0.00774 0.03477

108 4S -0.03923 -0.02706 -0.01258 0.00544 -0.04790

109 4PX 0.02208 -0.01928 -0.01120 0.00519 0.00955

110 4PY 0.02314 -0.01986 0.02025 -0.00687 0.02440

111 4PZ 0.04855 -0.05789 0.03121 -0.00622 0.02740

112 5D 0 -0.00684 -0.00115 0.00076 0.00006 -0.00684

113 5D+1 0.00996 -0.00570 0.00206 -0.00150 0.00956

114 5D-1 -0.00321 0.00581 -0.00385 0.00052 -0.00169

115 5D+2 0.00180 0.01258 -0.00324 0.00357 0.00285

116 5D-2 -0.00486 -0.00460 0.00520 0.00030 -0.00347

117 7 C 1S 0.00132 0.00527 -0.00152 0.00233 0.00337

118 2S 0.00254 0.00789 -0.00395 0.00279 0.00479

119 2PX -0.00640 -0.03548 0.01944 -0.00314 -0.02096

120 2PY -0.00673 0.01679 -0.00213 0.00590 -0.00608

121 2PZ -0.00082 0.00067 -0.00722 0.00262 -0.00893

122 3S -0.01156 -0.01943 0.01596 -0.00491 -0.01437

123 3PX -0.00661 -0.05020 0.02646 -0.00407 -0.02837

124 3PY -0.00890 0.02539 -0.00395 0.00867 -0.00808

125 3PZ -0.00131 -0.00009 -0.00972 0.00401 -0.01364

126 4S 0.00223 -0.01588 0.02332 -0.00729 0.00337

127 4PX -0.01410 -0.04408 0.02679 -0.00474 -0.03160

128 4PY -0.01079 0.03619 -0.01039 0.01091 -0.00667

129 4PZ -0.02454 -0.00868 -0.00199 0.00049 -0.03632

130 5D 0 -0.00262 0.00389 -0.00227 0.00026 -0.00082

131 5D+1 0.00145 -0.00396 0.00328 -0.00081 0.00102

132 5D-1 0.00279 -0.00094 -0.00012 0.00003 0.00250

133 5D+2 0.00325 0.00328 -0.00322 0.00101 0.00211

134 5D-2 0.00105 -0.00375 0.00233 -0.00062 -0.00029

135 8 C 1S -0.00134 0.00832 -0.00506 -0.00293 -0.00082

136 2S -0.00230 0.01187 -0.00560 -0.00348 -0.00169

137 2PX 0.01448 -0.00954 -0.00856 0.00623 0.01615

138 2PY 0.02865 -0.02351 0.00886 -0.00764 0.03284

139 2PZ -0.03635 0.00027 0.00231 0.00153 -0.03820

140 3S 0.00890 -0.02866 0.00782 0.00692 0.00748

141 3PX 0.02175 -0.01265 -0.01207 0.00828 0.02403

142 3PY 0.04125 -0.03566 0.01339 -0.01104 0.04761

143 3PZ -0.05244 0.00062 0.00431 0.00162 -0.05526

144 4S 0.00406 -0.04311 0.01160 -0.00133 0.00407

145 4PX 0.02295 -0.01480 -0.00986 0.00497 0.02491

146 4PY 0.05273 -0.03449 0.01395 -0.01140 0.05852

147 4PZ -0.06685 0.00042 0.00413 0.00087 -0.06989

148 5D 0 0.00032 -0.00036 0.00062 -0.00002 -0.00024

149 5D+1 -0.00192 0.00146 0.00032 0.00084 -0.00295

150 5D-1 0.00128 -0.00257 0.00105 -0.00034 0.00122

151 5D+2 0.00017 -0.00053 -0.00075 0.00122 0.00000

152 5D-2 -0.00195 -0.00069 0.00013 0.00067 -0.00235

153 9 C 1S 0.00051 -0.00360 -0.00467 -0.00036 -0.00106

154 2S 0.00082 -0.00587 -0.00529 -0.00059 -0.00179

155 2PX -0.00045 0.00306 0.00932 -0.00420 0.00402

156 2PY -0.00647 -0.01331 -0.00431 0.01290 -0.00599

157 2PZ -0.00794 0.01692 0.00237 0.00313 -0.01374

158 3S -0.00254 0.01760 0.00759 0.00308 0.00540

159 3PX -0.00233 0.00571 0.01225 -0.00568 0.00410

160 3PY -0.00832 -0.01604 -0.00612 0.01871 -0.00755

161 3PZ -0.01020 0.02469 0.00335 0.00500 -0.01949

162 4S 0.02316 0.02494 0.00515 -0.00690 0.02844

163 4PX 0.01409 -0.01436 0.00776 -0.01217 0.01589

164 4PY -0.01146 0.00078 -0.00236 0.01392 -0.00995

165 4PZ -0.03375 0.02846 -0.00205 0.00628 -0.03821

166 5D 0 0.00148 -0.00056 -0.00014 0.00021 0.00143

167 5D+1 0.00855 -0.00013 -0.00092 0.00118 0.00788

168 5D-1 0.00280 -0.00004 -0.00014 0.00140 0.00164

169 5D+2 -0.00675 -0.00090 -0.00224 0.00504 -0.00639

170 5D-2 -0.00434 0.00380 -0.00051 0.00066 -0.00451

171 10 H 1S 0.00096 0.05550 0.01623 0.00034 0.00528

172 2S 0.00190 0.08303 0.02223 0.00090 0.00895

173 3S 0.00638 0.04443 0.01569 -0.00059 0.01014

174 4PX 0.00088 -0.00306 0.00023 -0.00038 0.00057

175 4PY -0.00001 -0.00059 -0.00102 -0.00059 -0.00015

176 4PZ -0.00174 0.00137 -0.00061 0.00020 -0.00167

177 11 H 1S 0.00176 -0.02910 0.01048 -0.00362 0.00364

178 2S 0.00497 -0.04530 0.01677 -0.00723 0.00644

179 3S 0.01045 -0.04337 0.01421 -0.00841 0.00907

180 4PX 0.00224 0.00044 -0.00154 0.00087 0.00165

181 4PY 0.00175 0.00012 0.00021 -0.00075 0.00109

182 4PZ 0.00255 -0.00340 0.00233 -0.00033 0.00156

183 12 H 1S -0.00110 0.02039 -0.01497 0.00435 0.00067

184 2S 0.00237 0.03041 -0.02550 0.00727 0.00491

185 3S 0.00495 0.01769 -0.01559 0.00489 0.00659

186 4PX -0.00070 -0.00146 0.00036 0.00000 -0.00189

187 4PY -0.00041 0.00140 -0.00013 0.00043 -0.00026

188 4PZ -0.00068 -0.00212 0.00100 -0.00024 -0.00136

189 13 H 1S -0.00172 0.00811 0.00215 0.00069 -0.00405

190 2S -0.00325 0.01202 0.00277 0.00109 -0.00755

191 3S -0.00971 0.00875 -0.00107 0.00107 -0.01077

192 4PX 0.00208 0.00051 -0.00028 0.00015 0.00201

193 4PY 0.00314 -0.00133 0.00085 -0.00081 0.00334

194 4PZ -0.00316 0.00128 0.00040 -0.00009 -0.00339

195 14 H 1S 0.00974 0.01303 0.00819 -0.01386 0.01045

196 2S 0.01634 0.01781 0.01197 -0.01966 0.01757

197 3S 0.01444 -0.00321 0.00945 -0.01492 0.01354

198 4PX 0.00047 -0.00002 0.00068 -0.00016 0.00077

199 4PY -0.00075 0.00283 0.00069 -0.00020 -0.00052

200 4PZ -0.00077 0.00086 -0.00002 0.00046 -0.00128

76 77 78 79 80

76 5D 0 0.00084

77 5D+1 -0.00016 0.00186

78 5D-1 -0.00008 -0.00012 0.00155

79 5D+2 -0.00036 -0.00021 0.00030 0.00345

80 5D-2 -0.00020 0.00029 -0.00016 0.00033 0.00301

81 5 C 1S 0.00166 -0.00088 0.00262 0.00604 0.00157

82 2S 0.00207 -0.00112 0.00350 0.00813 0.00206

83 2PX 0.00012 0.00062 0.00133 0.00146 -0.00610

84 2PY -0.00029 0.00174 -0.00499 -0.00614 -0.00402

85 2PZ 0.00310 0.00165 -0.00772 0.00236 0.00210

86 3S -0.00392 0.00244 -0.00779 -0.01851 -0.00411

87 3PX 0.00015 0.00100 0.00164 0.00116 -0.00935

88 3PY -0.00044 0.00222 -0.00716 -0.00834 -0.00536

89 3PZ 0.00475 0.00247 -0.01195 0.00331 0.00298

90 4S -0.00042 0.00157 -0.00638 -0.01871 -0.00635

91 4PX -0.00146 0.00172 0.00316 0.00437 -0.00761

92 4PY 0.00026 0.00209 -0.00600 -0.00788 -0.00631

93 4PZ 0.00402 0.00266 -0.01321 0.00362 0.00420

94 5D 0 0.00013 -0.00017 0.00020 0.00019 0.00021

95 5D+1 -0.00001 0.00009 -0.00013 -0.00013 -0.00028

96 5D-1 0.00047 0.00009 0.00039 -0.00013 0.00002

97 5D+2 0.00016 -0.00003 0.00004 0.00000 -0.00057

98 5D-2 -0.00011 0.00034 -0.00006 0.00018 0.00109

99 6 C 1S 0.00116 0.00240 -0.00137 -0.00301 0.00368

100 2S 0.00143 0.00315 -0.00185 -0.00402 0.00492

101 2PX 0.00047 -0.00410 0.00333 0.00062 -0.00618

102 2PY 0.00005 0.00251 0.00079 -0.00783 -0.00375

103 2PZ 0.00364 -0.00520 0.00245 -0.00232 0.00229

104 3S -0.00263 -0.00672 0.00410 0.00937 -0.01081

105 3PX 0.00070 -0.00589 0.00463 0.00093 -0.00880

106 3PY 0.00022 0.00340 0.00119 -0.01106 -0.00531

107 3PZ 0.00549 -0.00787 0.00375 -0.00339 0.00309

108 4S -0.00143 -0.00742 0.00346 0.00440 -0.00880

109 4PX 0.00156 -0.00757 0.00400 -0.00083 -0.00487

110 4PY 0.00030 0.00238 0.00174 -0.00601 -0.00349

111 4PZ 0.00430 -0.00767 0.00476 -0.00473 -0.00015

112 5D 0 0.00006 -0.00044 0.00035 0.00014 0.00006

113 5D+1 0.00038 0.00045 -0.00028 -0.00079 -0.00016

114 5D-1 -0.00032 0.00050 -0.00026 0.00009 -0.00010

115 5D+2 -0.00023 0.00046 -0.00026 0.00129 0.00096

116 5D-2 -0.00002 0.00001 0.00022 0.00069 0.00101

117 7 C 1S -0.00009 0.00085 -0.00046 -0.00159 -0.00086

118 2S -0.00012 0.00118 -0.00055 -0.00218 -0.00164

119 2PX 0.00144 -0.00673 0.00350 0.00186 0.00076

120 2PY 0.00016 0.00162 -0.00244 0.00228 0.00349

121 2PZ 0.00148 -0.00344 0.00288 -0.00369 -0.00347

122 3S 0.00020 -0.00268 0.00109 0.00511 0.00563

123 3PX 0.00216 -0.00976 0.00505 0.00239 0.00080

124 3PY 0.00028 0.00247 -0.00359 0.00303 0.00497

125 3PZ 0.00224 -0.00528 0.00418 -0.00507 -0.00478

126 4S 0.00010 -0.00215 0.00202 0.00297 0.00401

127 4PX 0.00193 -0.00871 0.00569 0.00036 -0.00020

128 4PY -0.00034 0.00347 -0.00460 0.00335 0.00501

129 4PZ 0.00141 -0.00595 0.00482 -0.00352 -0.00445

130 5D 0 -0.00027 0.00054 -0.00023 0.00025 0.00008

131 5D+1 0.00016 -0.00015 0.00026 -0.00064 -0.00017

132 5D-1 0.00010 -0.00028 0.00030 -0.00020 -0.00023

133 5D+2 0.00016 -0.00044 0.00006 0.00052 0.00001

134 5D-2 0.00016 -0.00051 0.00035 0.00003 -0.00002

135 8 C 1S 0.00008 -0.00024 -0.00004 0.00034 0.00030

136 2S 0.00014 -0.00032 -0.00013 0.00038 0.00051

137 2PX -0.00018 0.00086 -0.00062 0.00039 0.00031

138 2PY -0.00031 0.00018 0.00030 -0.00091 0.00032

139 2PZ -0.00075 -0.00025 0.00064 -0.00026 -0.00127

140 3S -0.00033 0.00069 0.00047 -0.00049 -0.00156

141 3PX -0.00024 0.00114 -0.00088 0.00045 0.00053

142 3PY -0.00054 0.00020 0.00055 -0.00109 0.00049

143 3PZ -0.00106 -0.00038 0.00090 -0.00043 -0.00187

144 4S -0.00040 0.00015 0.00192 -0.00189 -0.00376

145 4PX 0.00009 0.00112 -0.00130 0.00038 0.00015

146 4PY 0.00015 0.00068 0.00007 -0.00238 0.00000

147 4PZ -0.00123 -0.00106 0.00226 -0.00097 -0.00197

148 5D 0 0.00011 -0.00018 0.00004 -0.00004 -0.00014

149 5D+1 0.00008 0.00000 -0.00041 0.00011 0.00017

150 5D-1 0.00003 -0.00022 0.00026 -0.00004 0.00002

151 5D+2 0.00002 -0.00008 0.00005 -0.00027 0.00021

152 5D-2 0.00000 -0.00024 0.00007 0.00009 0.00024

153 9 C 1S 0.00021 -0.00014 0.00020 0.00070 -0.00144

154 2S 0.00032 -0.00016 0.00019 0.00063 -0.00235

155 2PX -0.00026 -0.00114 0.00156 -0.00045 0.00306

156 2PY -0.00078 0.00010 -0.00087 -0.00114 0.00392

157 2PZ 0.00052 0.00082 -0.00434 0.00124 0.00102

158 3S -0.00082 0.00043 -0.00022 -0.00004 0.00703

159 3PX -0.00048 -0.00167 0.00229 -0.00080 0.00420

160 3PY -0.00115 0.00023 -0.00136 -0.00155 0.00558

161 3PZ 0.00088 0.00128 -0.00668 0.00180 0.00157

162 4S -0.00112 0.00180 0.00031 0.00406 0.00219

163 4PX -0.00040 -0.00095 0.00197 0.00039 0.00038

164 4PY -0.00087 0.00007 -0.00011 0.00129 0.00331

165 4PZ -0.00030 0.00163 -0.00506 0.00193 0.00110

166 5D 0 0.00009 0.00002 0.00002 0.00018 -0.00004

167 5D+1 0.00026 0.00024 -0.00077 0.00027 0.00028

168 5D-1 0.00021 0.00001 -0.00048 0.00014 0.00015

169 5D+2 -0.00008 -0.00003 0.00043 0.00060 -0.00016

170 5D-2 -0.00010 -0.00008 -0.00016 -0.00037 0.00045

171 10 H 1S 0.00051 -0.00019 0.00005 -0.00123 -0.00043

172 2S 0.00056 0.00027 0.00026 -0.00002 -0.00084

173 3S 0.00021 0.00052 0.00005 0.00033 0.00006

174 4PX -0.00009 0.00003 -0.00013 -0.00005 0.00009

175 4PY -0.00007 0.00014 0.00007 0.00029 -0.00011

176 4PZ -0.00004 0.00040 0.00028 -0.00016 0.00008

177 11 H 1S -0.00078 0.00146 0.00064 -0.00517 -0.00524

178 2S -0.00085 0.00150 0.00073 -0.00782 -0.00731

179 3S 0.00049 0.00011 -0.00035 -0.00739 -0.00237

180 4PX 0.00003 -0.00021 0.00028 -0.00022 -0.00047

181 4PY 0.00027 -0.00027 0.00000 0.00005 0.00046

182 4PZ 0.00020 -0.00034 0.00023 -0.00025 -0.00002

183 12 H 1S 0.00019 0.00098 -0.00048 -0.00106 -0.00065

184 2S 0.00023 0.00181 -0.00108 -0.00147 -0.00105

185 3S 0.00002 0.00138 -0.00123 -0.00001 -0.00060

186 4PX 0.00017 -0.00050 0.00033 -0.00022 -0.00013

187 4PY -0.00002 0.00015 -0.00022 0.00030 0.00032

188 4PZ 0.00003 -0.00042 0.00025 0.00013 -0.00009

189 13 H 1S 0.00050 -0.00039 -0.00017 0.00021 0.00040

190 2S 0.00079 -0.00081 -0.00058 0.00023 0.00110

191 3S 0.00012 -0.00005 -0.00061 0.00030 0.00111

192 4PX 0.00006 0.00004 -0.00008 0.00002 0.00004

193 4PY 0.00002 0.00000 0.00001 -0.00005 0.00001

194 4PZ -0.00002 -0.00003 0.00008 -0.00002 -0.00010

195 14 H 1S 0.00052 -0.00010 0.00005 0.00028 -0.00130

196 2S 0.00078 -0.00022 -0.00014 0.00009 -0.00180

197 3S 0.00054 -0.00062 0.00014 -0.00172 -0.00151

198 4PX -0.00001 -0.00009 0.00011 -0.00007 0.00024

199 4PY 0.00004 -0.00001 -0.00002 0.00019 0.00006

200 4PZ 0.00002 0.00008 -0.00038 0.00006 0.00014

81 82 83 84 85

81 5 C 1S 0.65858

82 2S 0.56054 0.49017

83 2PX 0.00463 0.00613 0.06928

84 2PY -0.00259 -0.00367 0.00705 0.09058

85 2PZ -0.00005 -0.00024 -0.00361 0.00240 0.07931

86 3S -0.08547 -0.14829 -0.01510 0.00887 0.00166

87 3PX -0.00293 -0.00265 0.10686 0.00859 -0.00433

88 3PY -0.00031 -0.00150 0.00769 0.12904 0.00426

89 3PZ -0.00162 -0.00230 -0.00478 0.00467 0.12173

90 4S -0.08923 -0.12923 0.02491 0.00854 0.00471

91 4PX -0.00110 -0.00334 0.07603 -0.00250 -0.01019

92 4PY -0.00757 -0.00994 0.01125 0.10107 0.00605

93 4PZ -0.00044 -0.00141 -0.01113 0.01129 0.12236

94 5D 0 0.00183 0.00385 -0.00028 -0.00039 -0.00133

95 5D+1 -0.00057 -0.00096 0.00207 0.00038 0.00040

96 5D-1 0.00062 0.00127 0.00056 -0.00136 0.00191

97 5D+2 -0.00204 -0.00238 0.00530 -0.00692 0.00185

98 5D-2 0.00043 -0.00007 -0.00526 -0.00408 0.00100

99 6 C 1S -0.00154 -0.00234 -0.00097 0.00389 -0.00075

100 2S -0.00231 -0.00366 -0.00156 0.00614 -0.00124

101 2PX 0.00408 0.00644 0.00171 -0.00690 0.00148

102 2PY -0.00363 -0.00573 0.00013 0.00669 -0.00495

103 2PZ -0.00151 -0.00238 0.00128 0.00247 -0.00795

104 3S 0.00636 0.01054 0.00511 -0.01844 0.00463

105 3PX 0.00381 0.00706 0.00191 -0.00873 0.00385

106 3PY -0.00412 -0.00710 -0.00033 0.00937 -0.00706

107 3PZ -0.00174 -0.00294 0.00248 0.00362 -0.01134

108 4S 0.00707 0.01122 0.00428 -0.01993 -0.01896

109 4PX 0.00590 0.00824 0.00497 -0.01018 -0.01208

110 4PY -0.00403 -0.00645 0.00183 0.00773 -0.00442

111 4PZ -0.00215 -0.00305 0.00259 0.00422 -0.01997

112 5D 0 0.00040 0.00059 0.00051 -0.00264 -0.00338

113 5D+1 -0.00052 -0.00084 0.00024 0.00373 0.00285

114 5D-1 -0.00043 -0.00043 -0.00050 0.00041 0.00034

115 5D+2 0.00053 0.00112 -0.00004 -0.00329 0.00501

116 5D-2 0.00159 0.00217 0.00181 -0.00429 -0.00146

117 7 C 1S -0.00086 -0.00123 -0.00230 0.00130 0.00256

118 2S -0.00125 -0.00191 -0.00365 0.00198 0.00414

119 2PX 0.00111 0.00175 0.00356 -0.00931 -0.02119

120 2PY -0.00156 -0.00249 -0.00581 -0.00189 0.01361

121 2PZ -0.00122 -0.00181 -0.00206 -0.00323 -0.01420

122 3S 0.00297 0.00501 0.01058 -0.00622 -0.01322

123 3PX 0.00068 0.00147 0.00502 -0.01274 -0.02970

124 3PY -0.00277 -0.00442 -0.00901 -0.00198 0.01989

125 3PZ -0.00128 -0.00211 -0.00327 -0.00551 -0.02044

126 4S 0.00655 0.00860 0.01602 -0.00668 -0.01327

127 4PX 0.00202 0.00242 0.00537 -0.01422 -0.03445

128 4PY -0.00230 -0.00355 -0.01081 -0.00343 0.02713

129 4PZ -0.00178 -0.00195 -0.00106 -0.00916 -0.03093

130 5D 0 0.00021 0.00035 0.00013 -0.00033 0.00056

131 5D+1 -0.00033 -0.00049 0.00040 0.00028 -0.00136

132 5D-1 0.00043 0.00063 0.00043 -0.00035 -0.00084

133 5D+2 0.00028 0.00046 -0.00046 -0.00092 0.00114

134 5D-2 -0.00026 -0.00031 0.00069 -0.00013 -0.00199

135 8 C 1S -0.00134 -0.00201 -0.00325 -0.00194 -0.00004

136 2S -0.00201 -0.00317 -0.00512 -0.00298 -0.00007

137 2PX 0.00463 0.00732 0.00558 0.00730 0.00455

138 2PY -0.00033 -0.00052 -0.00180 0.00102 -0.00020

139 2PZ 0.00084 0.00133 0.00406 -0.00061 -0.00746

140 3S 0.00577 0.00940 0.01481 0.00837 0.00169

141 3PX 0.00481 0.00842 0.00675 0.01027 0.00619

142 3PY -0.00020 -0.00044 -0.00204 0.00045 -0.00079

143 3PZ 0.00094 0.00172 0.00608 -0.00034 -0.01058

144 4S 0.00269 0.00478 0.02732 0.00873 -0.01206

145 4PX 0.00408 0.00670 0.01181 0.01065 0.00830

146 4PY 0.00125 0.00246 0.00137 0.00839 0.00333

147 4PZ -0.00157 -0.00211 0.00325 -0.00633 -0.02317

148 5D 0 0.00011 0.00008 0.00038 -0.00037 0.00047

149 5D+1 -0.00055 -0.00081 -0.00164 -0.00048 0.00330

150 5D-1 -0.00013 -0.00021 0.00015 -0.00109 -0.00136

151 5D+2 -0.00125 -0.00172 -0.00304 -0.00215 0.00002

152 5D-2 -0.00082 -0.00153 -0.00258 -0.00345 -0.00001

153 9 C 1S 0.00540 0.00854 0.01315 0.01293 -0.00161

154 2S 0.00856 0.01314 0.02099 0.02075 -0.00261

155 2PX -0.01423 -0.02279 -0.02924 -0.03851 -0.00627

156 2PY -0.01242 -0.01988 -0.03454 -0.02787 0.00761

157 2PZ -0.00282 -0.00447 -0.00976 0.00004 0.03408

158 3S -0.02226 -0.03357 -0.05943 -0.05986 0.00879

159 3PX -0.02524 -0.03718 -0.04117 -0.05518 -0.01010

160 3PY -0.02209 -0.03250 -0.04821 -0.04025 0.01231

161 3PZ -0.00412 -0.00654 -0.01498 0.00062 0.05288

162 4S -0.00103 -0.00310 -0.02571 -0.01543 0.00798

163 4PX -0.00615 -0.01031 -0.01260 -0.01272 -0.01253

164 4PY -0.00510 -0.00953 -0.02630 -0.02934 0.00764

165 4PZ -0.00452 -0.00697 -0.01276 -0.00104 0.03405

166 5D 0 0.00211 0.00275 0.00157 0.00131 0.00038

167 5D+1 0.00017 0.00019 -0.00036 0.00071 0.00742

168 5D-1 -0.00062 -0.00081 -0.00061 -0.00075 0.00519

169 5D+2 -0.00069 -0.00082 0.00424 -0.00745 -0.00027

170 5D-2 -0.00580 -0.00756 -0.00542 -0.00472 0.00016

171 10 H 1S -0.00269 -0.00425 0.00967 -0.00153 0.00232

172 2S -0.00256 -0.00448 0.01702 -0.00514 0.00494

173 3S -0.00039 -0.00102 0.01485 0.00103 0.00439

174 4PX -0.00035 -0.00050 0.00026 0.00133 0.00022

175 4PY -0.00030 -0.00041 0.00086 0.00013 -0.00021

176 4PZ -0.00004 -0.00006 0.00001 -0.00034 -0.00162

177 11 H 1S -0.00268 -0.00418 -0.00038 0.00732 -0.00393

178 2S -0.00431 -0.00666 -0.00212 0.01318 -0.00605

179 3S -0.00328 -0.00549 -0.00282 0.01818 -0.00616

180 4PX -0.00001 0.00008 0.00030 -0.00034 -0.00029

181 4PY 0.00017 0.00014 -0.00003 0.00065 -0.00055

182 4PZ -0.00010 -0.00015 0.00007 0.00006 -0.00071

183 12 H 1S -0.00048 -0.00073 -0.00258 0.00050 0.00368

184 2S -0.00118 -0.00180 -0.00492 0.00199 0.00835

185 3S 0.00021 0.00001 -0.00413 0.00115 0.01053

186 4PX -0.00025 -0.00033 0.00001 -0.00044 -0.00205

187 4PY -0.00001 -0.00003 -0.00037 -0.00017 0.00129

188 4PZ 0.00030 0.00041 0.00024 -0.00080 -0.00149

189 13 H 1S -0.00241 -0.00384 -0.00500 -0.00477 0.00285

190 2S -0.00485 -0.00730 -0.01141 -0.00770 0.00576

191 3S -0.00291 -0.00457 -0.00943 -0.00623 0.00311

192 4PX -0.00005 0.00004 0.00010 0.00061 0.00065

193 4PY -0.00004 -0.00007 -0.00005 0.00039 0.00017

194 4PZ -0.00019 -0.00021 0.00020 -0.00009 -0.00087

195 14 H 1S 0.00352 0.00581 0.00792 0.01127 -0.00143

196 2S 0.00451 0.00795 0.01124 0.01743 -0.00112

197 3S 0.00479 0.00689 0.00530 0.02026 -0.00619

198 4PX -0.00078 -0.00123 -0.00212 -0.00222 -0.00047

199 4PY -0.00110 -0.00119 0.00005 -0.00218 0.00091

200 4PZ -0.00013 -0.00030 -0.00128 0.00015 0.00277

86 87 88 89 90

86 3S 0.44786

87 3PX -0.00147 0.16638

88 3PY 0.00731 0.00834 0.18425

89 3PZ 0.00628 -0.00527 0.00786 0.18696

90 4S 0.32109 0.05630 0.00794 0.01061 0.30727

91 4PX 0.01105 0.11681 -0.00706 -0.01499 0.02510

92 4PY 0.02088 0.01513 0.14276 0.01059 0.01775

93 4PZ 0.00699 -0.01550 0.01732 0.18760 -0.00023

94 5D 0 -0.01339 -0.00104 -0.00033 -0.00211 -0.00730

95 5D+1 0.00266 0.00342 0.00030 0.00072 0.00134

96 5D-1 -0.00441 0.00071 -0.00212 0.00283 -0.00444

97 5D+2 0.00383 0.00890 -0.01026 0.00292 0.00916

98 5D-2 0.00256 -0.00812 -0.00592 0.00142 -0.00334

99 6 C 1S 0.00630 -0.00082 0.00345 -0.00099 0.00709

100 2S 0.01041 -0.00150 0.00637 -0.00174 0.00935

101 2PX -0.01907 0.00117 -0.00931 0.00268 -0.02496

102 2PY 0.01669 -0.00025 0.00753 -0.00687 0.01735

103 2PZ 0.00582 0.00221 0.00326 -0.01188 0.00549

104 3S -0.03153 0.00587 -0.02252 0.00688 -0.02075

105 3PX -0.02454 0.00088 -0.01168 0.00650 -0.03517

106 3PY 0.02265 -0.00124 0.01048 -0.00982 0.02297

107 3PZ 0.00755 0.00404 0.00472 -0.01690 0.00673

108 4S -0.03154 0.00480 -0.02631 -0.02897 -0.00956

109 4PX -0.02090 0.00629 -0.01456 -0.01786 -0.02103

110 4PY 0.01810 0.00193 0.00883 -0.00599 0.00625

111 4PZ 0.00627 0.00391 0.00548 -0.02992 0.00606

112 5D 0 -0.00155 0.00077 -0.00375 -0.00518 0.00048

113 5D+1 0.00243 0.00036 0.00500 0.00442 0.00064

114 5D-1 0.00053 -0.00069 0.00062 0.00047 0.00021

115 5D+2 -0.00394 -0.00005 -0.00442 0.00746 -0.00456

116 5D-2 -0.00492 0.00271 -0.00622 -0.00234 -0.00334

117 7 C 1S 0.00293 -0.00290 0.00131 0.00392 0.00762

118 2S 0.00505 -0.00496 0.00239 0.00638 0.00972

119 2PX -0.00606 0.00551 -0.01232 -0.03220 0.00212

120 2PY 0.00793 -0.00761 -0.00141 0.02000 0.01595

121 2PZ 0.00432 -0.00361 -0.00480 -0.02115 0.00813

122 3S -0.01515 0.01560 -0.00891 -0.02047 -0.01904

123 3PX -0.00706 0.00783 -0.01681 -0.04506 0.00412

124 3PY 0.01387 -0.01176 -0.00092 0.02924 0.02571

125 3PZ 0.00555 -0.00563 -0.00803 -0.03049 0.01175

126 4S -0.01936 0.02327 -0.01083 -0.02013 -0.03207

127 4PX -0.00599 0.00786 -0.01974 -0.05207 0.00306

128 4PY 0.01094 -0.01480 -0.00322 0.04038 0.01887

129 4PZ 0.00210 -0.00215 -0.01285 -0.04655 0.01353

130 5D 0 -0.00095 0.00020 -0.00051 0.00079 -0.00149

131 5D+1 0.00119 0.00061 0.00025 -0.00200 0.00134

132 5D-1 -0.00169 0.00047 -0.00066 -0.00124 -0.00296

133 5D+2 -0.00145 -0.00068 -0.00112 0.00169 -0.00205

134 5D-2 0.00048 0.00110 -0.00012 -0.00302 0.00115

135 8 C 1S 0.00564 -0.00314 -0.00112 0.00018 0.00285

136 2S 0.00926 -0.00573 -0.00263 0.00013 0.00481

137 2PX -0.02115 0.00688 0.00922 0.00663 -0.02955

138 2PY 0.00113 -0.00309 -0.00010 -0.00007 -0.02304

139 2PZ -0.00405 0.00557 -0.00049 -0.01066 0.00943

140 3S -0.02802 0.01916 0.01048 0.00238 -0.01461

141 3PX -0.02709 0.00813 0.01297 0.00896 -0.04045

142 3PY 0.00106 -0.00367 -0.00168 -0.00086 -0.03457

143 3PZ -0.00605 0.00835 0.00007 -0.01505 0.01384

144 4S -0.01596 0.03952 0.01006 -0.01796 -0.00055

145 4PX -0.01990 0.01700 0.01379 0.01235 -0.02342

146 4PY -0.00875 0.00105 0.00942 0.00531 -0.04060

147 4PZ 0.00441 0.00475 -0.00820 -0.03439 0.02781

148 5D 0 0.00007 0.00063 -0.00048 0.00078 0.00125

149 5D+1 0.00214 -0.00238 -0.00043 0.00509 0.00429

150 5D-1 0.00056 0.00025 -0.00165 -0.00208 -0.00045

151 5D+2 0.00397 -0.00451 -0.00307 -0.00001 0.00201

152 5D-2 0.00505 -0.00358 -0.00476 0.00000 0.00495

153 9 C 1S -0.02250 0.02256 0.02201 -0.00219 0.00637

154 2S -0.03397 0.03364 0.03262 -0.00359 0.00682

155 2PX 0.06452 -0.04078 -0.05475 -0.00969 0.02544

156 2PY 0.05641 -0.05016 -0.03965 0.01160 0.03657

157 2PZ 0.01317 -0.01390 0.00196 0.05274 0.02506

158 3S 0.08648 -0.08809 -0.08570 0.01232 -0.01009

159 3PX 0.09532 -0.05705 -0.07849 -0.01555 0.03928

160 3PY 0.08346 -0.06968 -0.05730 0.01879 0.05414

161 3PZ 0.01936 -0.02139 0.00376 0.08184 0.03751

162 4S 0.01118 -0.04172 -0.02281 0.01124 -0.05996

163 4PX 0.02996 -0.01853 -0.01893 -0.01921 -0.00498

164 4PY 0.03119 -0.03895 -0.04152 0.01147 0.01623

165 4PZ 0.01994 -0.01817 0.00134 0.05267 0.04300

166 5D 0 -0.00578 0.00216 0.00188 0.00054 -0.00298

167 5D+1 -0.00028 -0.00049 0.00101 0.01130 -0.00151

168 5D-1 0.00181 -0.00073 -0.00093 0.00801 0.00315

169 5D+2 0.00134 0.00684 -0.01073 -0.00042 0.00622

170 5D-2 0.01618 -0.00731 -0.00650 0.00028 0.01301

171 10 H 1S 0.01204 0.01601 -0.00205 0.00370 0.02142

172 2S 0.01375 0.02736 -0.00741 0.00770 0.02314

173 3S 0.00392 0.02293 0.00106 0.00677 0.00672

174 4PX 0.00119 0.00034 0.00180 0.00036 -0.00062

175 4PY 0.00087 0.00122 0.00014 -0.00033 0.00004

176 4PZ 0.00018 0.00004 -0.00053 -0.00245 0.00053

177 11 H 1S 0.01209 -0.00124 0.00941 -0.00541 0.01375

178 2S 0.01891 -0.00419 0.01733 -0.00834 0.01977

179 3S 0.01680 -0.00409 0.02486 -0.00871 0.01947

180 4PX -0.00052 0.00040 -0.00050 -0.00040 -0.00047

181 4PY -0.00007 0.00003 0.00090 -0.00084 -0.00074

182 4PZ 0.00032 0.00007 0.00005 -0.00105 0.00027

183 12 H 1S 0.00246 -0.00415 0.00054 0.00558 0.00162

184 2S 0.00553 -0.00768 0.00264 0.01265 0.00336

185 3S 0.00131 -0.00637 0.00181 0.01595 0.00064

186 4PX 0.00063 0.00001 -0.00061 -0.00309 0.00121

187 4PY 0.00019 -0.00046 -0.00012 0.00191 0.00082

188 4PZ -0.00104 0.00034 -0.00108 -0.00226 -0.00062

189 13 H 1S 0.01142 -0.00681 -0.00586 0.00407 0.02058

190 2S 0.02017 -0.01597 -0.00937 0.00843 0.03205

191 3S 0.01325 -0.01333 -0.00785 0.00459 0.01805

192 4PX -0.00042 0.00012 0.00087 0.00092 -0.00026

193 4PY 0.00021 -0.00009 0.00047 0.00024 -0.00127

194 4PZ 0.00034 0.00032 -0.00002 -0.00131 0.00255

195 14 H 1S -0.01751 0.01090 0.01607 -0.00225 -0.02925

196 2S -0.02536 0.01558 0.02479 -0.00176 -0.04178

197 3S -0.01721 0.00679 0.02866 -0.00926 -0.02284

198 4PX 0.00341 -0.00303 -0.00316 -0.00073 0.00110

199 4PY 0.00150 0.00021 -0.00307 0.00137 -0.00051

200 4PZ 0.00122 -0.00187 0.00037 0.00429 0.00242

91 92 93 94 95

91 4PX 0.10876

92 4PY 0.00032 0.13212

93 4PZ -0.01975 0.01638 0.19760

94 5D 0 -0.00219 -0.00159 -0.00211 0.00073

95 5D+1 0.00208 0.00137 0.00198 0.00000 0.00117

96 5D-1 0.00119 0.00034 0.00316 -0.00019 0.00031

97 5D+2 0.00541 -0.00535 0.00111 -0.00021 0.00034

98 5D-2 -0.00360 -0.00267 0.00215 -0.00019 -0.00012

99 6 C 1S -0.00707 0.00266 0.00062 0.00005 -0.00024

100 2S -0.00849 0.00508 0.00092 0.00009 -0.00024

101 2PX 0.00722 -0.00718 -0.00332 -0.00073 0.00062

102 2PY 0.00530 0.02114 -0.00837 0.00040 -0.00012

103 2PZ 0.00194 0.00604 -0.01769 -0.00051 -0.00095

104 3S 0.01609 -0.01872 -0.00105 -0.00027 0.00025

105 3PX 0.01056 -0.00845 -0.00199 -0.00124 0.00090

106 3PY 0.00725 0.03002 -0.01194 0.00054 -0.00027

107 3PZ 0.00427 0.00930 -0.02581 -0.00078 -0.00140

108 4S 0.00380 -0.01728 -0.03463 0.00210 0.00025

109 4PX 0.00673 -0.00425 -0.02453 -0.00003 0.00097

110 4PY 0.01458 0.02081 -0.00868 -0.00052 0.00011

111 4PZ 0.00398 0.01154 -0.03956 -0.00056 -0.00090

112 5D 0 0.00024 -0.00362 -0.00584 0.00027 -0.00006

113 5D+1 0.00046 0.00617 0.00469 -0.00029 0.00005

114 5D-1 -0.00109 -0.00032 0.00148 0.00002 0.00021

115 5D+2 0.00075 -0.00606 0.00871 -0.00009 0.00006

116 5D-2 0.00249 -0.00489 -0.00233 0.00019 -0.00009

117 7 C 1S -0.00761 -0.00007 0.00403 -0.00009 0.00004

118 2S -0.00932 0.00058 0.00665 -0.00007 0.00013

119 2PX 0.00295 -0.01300 -0.04241 0.00043 -0.00136

120 2PY -0.01105 -0.00981 0.02718 0.00034 0.00029

121 2PZ -0.00089 -0.00187 -0.02760 0.00073 -0.00022

122 3S 0.01767 -0.00547 -0.02143 0.00010 -0.00050

123 3PX 0.00440 -0.01760 -0.05996 0.00054 -0.00183

124 3PY -0.01726 -0.01360 0.03998 0.00048 0.00049

125 3PZ -0.00204 -0.00417 -0.03997 0.00108 -0.00042

126 4S 0.02807 -0.00247 -0.02174 -0.00035 -0.00015

127 4PX 0.00799 -0.01731 -0.06826 0.00084 -0.00214

128 4PY -0.01787 -0.01544 0.05188 0.00031 0.00071

129 4PZ -0.00140 -0.01074 -0.05801 0.00171 -0.00063

130 5D 0 0.00004 -0.00054 0.00212 0.00004 0.00029

131 5D+1 0.00053 0.00099 -0.00284 0.00001 -0.00012

132 5D-1 0.00136 0.00045 -0.00153 -0.00006 0.00010

133 5D+2 -0.00085 -0.00173 0.00203 -0.00007 0.00018

134 5D-2 0.00063 -0.00009 -0.00371 0.00000 -0.00009

135 8 C 1S -0.00630 -0.00460 -0.00059 0.00017 -0.00021

136 2S -0.00971 -0.00625 -0.00053 0.00020 -0.00037

137 2PX 0.01868 0.01564 0.01510 -0.00075 0.00314

138 2PY 0.00437 0.01306 0.00465 -0.00127 0.00186

139 2PZ 0.00773 -0.00479 -0.02744 0.00073 -0.00459

140 3S 0.02704 0.01460 0.00246 -0.00037 0.00110

141 3PX 0.02492 0.02189 0.02163 -0.00113 0.00458

142 3PY 0.00766 0.01828 0.00584 -0.00189 0.00269

143 3PZ 0.01097 -0.00652 -0.03980 0.00109 -0.00682

144 4S 0.03623 0.01954 -0.02098 0.00002 0.00194

145 4PX 0.01970 0.02048 0.02463 -0.00064 0.00480

146 4PY 0.00867 0.02757 0.01649 -0.00166 0.00414

147 4PZ 0.00442 -0.01594 -0.05959 0.00161 -0.00646

148 5D 0 -0.00018 -0.00054 -0.00016 0.00006 -0.00023

149 5D+1 -0.00269 -0.00185 0.00349 0.00006 -0.00063

150 5D-1 0.00046 -0.00056 -0.00204 -0.00005 0.00013

151 5D+2 -0.00227 -0.00216 0.00050 -0.00009 0.00016

152 5D-2 -0.00345 -0.00443 -0.00088 0.00001 -0.00024

153 9 C 1S 0.00183 0.01295 -0.00530 0.00198 0.00006

154 2S 0.00667 0.02078 -0.00769 0.00251 0.00012

155 2PX -0.02964 -0.04189 -0.00882 -0.00155 -0.00054

156 2PY -0.02784 -0.02869 0.01115 -0.00111 -0.00016

157 2PZ -0.01857 -0.00835 0.03978 0.00021 -0.00545

158 3S -0.03107 -0.06054 0.02132 -0.00492 -0.00044

159 3PX -0.04161 -0.06032 -0.01436 -0.00228 -0.00066

160 3PY -0.03799 -0.04195 0.01814 -0.00174 -0.00024

161 3PZ -0.02837 -0.01172 0.06221 0.00032 -0.00812

162 4S 0.00438 -0.00613 0.01911 -0.00366 -0.00105

163 4PX -0.00759 -0.00439 -0.01600 -0.00145 0.00101

164 4PY -0.01585 -0.03307 0.00867 -0.00071 -0.00145

165 4PZ -0.02402 -0.01557 0.03355 0.00063 -0.00852

166 5D 0 0.00074 0.00150 0.00100 0.00019 0.00019

167 5D+1 -0.00054 0.00162 0.01319 -0.00020 0.00054

168 5D-1 -0.00123 -0.00117 0.00697 -0.00006 -0.00030

169 5D+2 0.00619 -0.00900 -0.00227 0.00004 -0.00007

170 5D-2 -0.00638 -0.00693 -0.00031 -0.00018 -0.00033

171 10 H 1S 0.01870 -0.02729 0.00863 0.00052 0.00020

172 2S 0.03487 -0.04110 0.01458 0.00048 0.00027

173 3S 0.02686 -0.01488 0.01112 0.00022 0.00052

174 4PX 0.00023 0.00370 0.00024 -0.00011 0.00000

175 4PY 0.00166 0.00097 -0.00055 -0.00003 0.00007

176 4PZ 0.00001 -0.00077 -0.00312 0.00009 0.00011

177 11 H 1S 0.00410 0.02107 -0.00716 0.00030 0.00000

178 2S 0.00323 0.03345 -0.01111 0.00034 -0.00020

179 3S -0.00913 0.03239 -0.00989 0.00010 0.00005

180 4PX 0.00065 0.00008 -0.00094 -0.00003 0.00004

181 4PY -0.00072 -0.00004 -0.00092 -0.00007 -0.00001

182 4PZ 0.00040 0.00064 -0.00148 -0.00002 -0.00006

183 12 H 1S -0.00161 0.00110 0.00858 0.00030 0.00064

184 2S -0.00390 0.00316 0.01771 0.00018 0.00110

185 3S -0.00467 0.00090 0.01864 -0.00012 0.00025

186 4PX 0.00014 -0.00043 -0.00389 0.00008 -0.00007

187 4PY -0.00091 -0.00094 0.00250 0.00001 0.00002

188 4PZ 0.00010 -0.00106 -0.00293 0.00003 -0.00005

189 13 H 1S -0.01431 -0.01144 0.00464 0.00046 -0.00044

190 2S -0.02620 -0.01807 0.00887 0.00067 -0.00084

191 3S -0.01753 -0.01242 0.00370 0.00044 -0.00089

192 4PX -0.00026 0.00061 0.00212 -0.00002 0.00032

193 4PY -0.00018 0.00112 0.00103 -0.00009 0.00022

194 4PZ -0.00064 -0.00115 -0.00235 0.00013 -0.00035

195 14 H 1S 0.00619 0.01287 -0.00029 -0.00042 -0.00048

196 2S 0.00777 0.01979 0.00175 -0.00060 -0.00046

197 3S 0.00021 0.02551 -0.00715 0.00010 0.00025

198 4PX -0.00212 -0.00243 -0.00048 -0.00007 0.00004

199 4PY 0.00039 -0.00296 0.00109 -0.00013 -0.00021

200 4PZ -0.00203 -0.00046 0.00336 0.00005 -0.00037

96 97 98 99 100

96 5D-1 0.00123

97 5D+2 0.00018 0.00278

98 5D-2 0.00022 -0.00025 0.00187

99 6 C 1S -0.00015 0.00001 0.00153 0.65850

100 2S -0.00030 -0.00030 0.00219 0.56008 0.48912

101 2PX 0.00332 0.00042 -0.00350 -0.00015 -0.00035

102 2PY 0.00088 -0.00030 0.00318 -0.00066 -0.00111

103 2PZ 0.00298 -0.00177 0.00149 -0.00065 -0.00085

104 3S 0.00102 0.00205 -0.00537 -0.08315 -0.14438

105 3PX 0.00491 0.00041 -0.00482 -0.00102 -0.00154

106 3PY 0.00141 -0.00053 0.00459 -0.00049 -0.00083

107 3PZ 0.00458 -0.00265 0.00225 -0.00042 -0.00072

108 4S -0.00177 0.00166 -0.00284 -0.06998 -0.10158

109 4PX 0.00347 0.00056 -0.00209 0.00101 0.00186

110 4PY 0.00204 -0.00145 0.00341 0.00521 0.00459

111 4PZ 0.00438 -0.00210 0.00069 -0.00350 -0.00442

112 5D 0 -0.00035 0.00026 0.00002 0.00014 0.00123

113 5D+1 0.00064 -0.00035 0.00029 0.00110 0.00253

114 5D-1 -0.00021 0.00013 -0.00028 0.00022 0.00094

115 5D+2 0.00011 0.00019 0.00000 -0.00033 -0.00028

116 5D-2 -0.00009 0.00023 0.00056 -0.00005 0.00028

117 7 C 1S 0.00015 0.00044 0.00002 0.00532 0.00838

118 2S 0.00019 0.00059 -0.00009 0.00838 0.01284

119 2PX -0.00032 0.00000 -0.00046 -0.01371 -0.02183

120 2PY -0.00115 0.00082 0.00054 -0.01067 -0.01700

121 2PZ 0.00047 0.00042 -0.00059 0.00767 0.01226

122 3S -0.00053 -0.00133 0.00064 -0.02169 -0.03287

123 3PX -0.00029 0.00006 -0.00074 -0.02237 -0.03337

124 3PY -0.00162 0.00108 0.00073 -0.01736 -0.02634

125 3PZ 0.00064 0.00065 -0.00088 0.01283 0.01862

126 4S 0.00116 -0.00247 0.00179 0.00300 0.00357

127 4PX -0.00057 0.00006 0.00003 -0.00587 -0.01048

128 4PY -0.00183 0.00223 0.00062 -0.00535 -0.00924

129 4PZ -0.00105 0.00063 -0.00107 0.00225 0.00360

130 5D 0 -0.00007 0.00004 -0.00002 0.00160 0.00203

131 5D+1 0.00014 -0.00017 0.00021 0.00198 0.00258

132 5D-1 0.00039 0.00002 0.00000 0.00286 0.00375

133 5D+2 0.00029 -0.00008 -0.00030 -0.00038 -0.00062

134 5D-2 0.00009 -0.00025 0.00000 -0.00359 -0.00474

135 8 C 1S -0.00054 -0.00206 -0.00043 -0.00089 -0.00137

136 2S -0.00077 -0.00305 -0.00025 -0.00139 -0.00219

137 2PX 0.00295 0.00543 0.00053 -0.00014 -0.00018

138 2PY 0.00354 -0.00200 0.00167 0.00342 0.00550

139 2PZ -0.00493 0.00252 -0.00093 0.00105 0.00164

140 3S 0.00196 0.00803 -0.00066 0.00359 0.00596

141 3PX 0.00430 0.00737 0.00079 -0.00010 -0.00027

142 3PY 0.00520 -0.00276 0.00250 0.00432 0.00729

143 3PZ -0.00725 0.00334 -0.00137 0.00160 0.00262

144 4S 0.00204 0.00437 -0.00190 0.00831 0.01113

145 4PX 0.00401 0.00436 -0.00056 0.00107 0.00082

146 4PY 0.00652 -0.00321 0.00234 0.00798 0.01163

147 4PZ -0.00777 0.00288 -0.00171 0.00082 0.00086

148 5D 0 -0.00011 -0.00022 -0.00006 0.00004 0.00016

149 5D+1 -0.00066 0.00025 -0.00002 -0.00007 -0.00019

150 5D-1 0.00028 -0.00011 0.00008 0.00010 0.00017

151 5D+2 0.00018 0.00063 0.00031 0.00026 0.00036

152 5D-2 -0.00023 0.00008 0.00010 -0.00021 -0.00043

153 9 C 1S -0.00032 -0.00080 -0.00443 -0.00027 -0.00037

154 2S -0.00041 -0.00090 -0.00583 -0.00036 -0.00053

155 2PX 0.00056 -0.00503 0.00457 0.00034 0.00054

156 2PY 0.00000 0.00705 0.00297 0.00112 0.00165

157 2PZ -0.00538 -0.00042 -0.00003 -0.00015 -0.00029

158 3S 0.00088 0.00165 0.01268 0.00104 0.00143

159 3PX 0.00070 -0.00695 0.00631 0.00000 0.00015

160 3PY -0.00007 0.01006 0.00412 0.00096 0.00176

161 3PZ -0.00802 -0.00047 -0.00005 -0.00014 -0.00032

162 4S 0.00184 -0.00279 0.00982 -0.00577 -0.00540

163 4PX 0.00218 -0.00524 0.00338 0.00009 0.00041

164 4PY -0.00075 0.00635 0.00299 -0.00348 -0.00362

165 4PZ -0.00895 0.00098 -0.00083 -0.00060 -0.00115

166 5D 0 0.00023 -0.00010 -0.00018 0.00009 0.00007

167 5D+1 0.00061 0.00024 0.00018 -0.00021 -0.00031

168 5D-1 -0.00016 0.00030 -0.00001 -0.00014 -0.00022

169 5D+2 -0.00026 0.00192 -0.00023 -0.00069 -0.00105

170 5D-2 -0.00061 0.00002 0.00023 -0.00048 -0.00066

171 10 H 1S -0.00033 -0.00109 -0.00209 0.00070 0.00118

172 2S -0.00042 -0.00124 -0.00244 0.00135 0.00199

173 3S 0.00009 -0.00102 -0.00193 0.00164 0.00245

174 4PX 0.00002 -0.00003 0.00008 0.00016 0.00024

175 4PY -0.00006 -0.00005 -0.00022 -0.00010 -0.00014

176 4PZ -0.00007 0.00001 0.00005 0.00007 0.00009

177 11 H 1S 0.00029 -0.00021 0.00160 -0.02027 -0.03183

178 2S 0.00043 -0.00058 0.00215 -0.03129 -0.04516

179 3S 0.00081 -0.00118 0.00068 -0.00061 -0.00170

180 4PX 0.00025 0.00005 -0.00017 -0.00108 -0.00136

181 4PY 0.00012 -0.00017 -0.00012 0.00640 0.00811

182 4PZ 0.00022 -0.00013 0.00017 -0.00122 -0.00155

183 12 H 1S 0.00009 0.00041 -0.00019 0.00468 0.00757

184 2S 0.00037 0.00099 -0.00049 0.00574 0.01004

185 3S 0.00011 0.00080 -0.00044 0.00407 0.00592

186 4PX -0.00002 0.00003 -0.00004 -0.00093 -0.00100

187 4PY -0.00009 0.00005 0.00001 -0.00086 -0.00133

188 4PZ 0.00000 0.00002 -0.00007 0.00017 -0.00012

189 13 H 1S -0.00062 -0.00160 -0.00035 -0.00097 -0.00159

190 2S -0.00119 -0.00235 -0.00037 -0.00208 -0.00336

191 3S -0.00149 -0.00099 0.00004 -0.00064 -0.00137

192 4PX 0.00029 -0.00001 -0.00003 -0.00019 -0.00026

193 4PY 0.00035 -0.00037 0.00008 0.00000 0.00010

194 4PZ -0.00042 -0.00011 -0.00016 -0.00006 -0.00008

195 14 H 1S -0.00001 -0.00710 0.00091 0.00003 0.00009

196 2S 0.00024 -0.01001 0.00097 -0.00052 -0.00039

197 3S 0.00076 -0.00589 -0.00048 0.00090 0.00170

198 4PX 0.00012 -0.00022 0.00024 0.00014 0.00020

199 4PY -0.00019 -0.00042 0.00028 -0.00027 -0.00038

200 4PZ -0.00041 0.00015 -0.00003 0.00008 0.00009

101 102 103 104 105

101 2PX 0.08447

102 2PY 0.00061 0.08757

103 2PZ -0.00549 -0.00175 0.07152

104 3S 0.00184 0.00112 0.00173 0.43582

105 3PX 0.12137 0.00188 -0.00592 0.00479 0.17469

106 3PY 0.00163 0.12421 -0.00079 -0.00055 0.00383

107 3PZ -0.00542 -0.00063 0.10718 0.00203 -0.00479

108 4S -0.00406 0.02132 0.00485 0.25177 -0.00535

109 4PX 0.06802 0.00299 0.02230 -0.00615 0.09762

110 4PY 0.00864 0.07576 0.00927 -0.00360 0.01389

111 4PZ 0.01608 0.01219 0.08667 0.00821 0.02537

112 5D 0 0.00020 -0.00098 0.00224 -0.00625 0.00019

113 5D+1 0.00094 0.00473 0.00155 -0.00954 0.00152

114 5D-1 0.00149 -0.00207 -0.00697 -0.00433 0.00194

115 5D+2 0.00033 -0.00708 -0.00388 0.00041 0.00052

116 5D-2 -0.00751 -0.00209 0.00281 -0.00189 -0.01093

117 7 C 1S 0.01300 0.01151 -0.00923 -0.02146 0.02273

118 2S 0.02079 0.01841 -0.01471 -0.03237 0.03325

119 2PX -0.01004 -0.02199 0.05478 0.06110 -0.01372

120 2PY -0.03976 -0.01750 0.00311 0.04881 -0.05600

121 2PZ 0.04152 0.02372 0.02064 -0.03393 0.06034

122 3S -0.06023 -0.05337 0.04126 0.08342 -0.08670

123 3PX -0.01098 -0.03049 0.07916 0.08660 -0.01468

124 3PY -0.05764 -0.02445 0.00439 0.07166 -0.08107

125 3PZ 0.05861 0.03173 0.03216 -0.04575 0.08526

126 4S -0.02057 -0.02514 0.03047 -0.00910 -0.03027

127 4PX 0.00549 -0.00865 0.07049 0.03271 0.00861

128 4PY -0.04267 -0.02678 -0.01495 0.03026 -0.06036

129 4PZ 0.03973 0.02408 0.03433 -0.00949 0.05771

130 5D 0 -0.00039 -0.00105 -0.00569 -0.00410 -0.00073

131 5D+1 0.00179 0.00506 0.00285 -0.00548 0.00262

132 5D-1 0.00578 0.00144 0.00098 -0.00813 0.00825

133 5D+2 0.00372 -0.00567 0.00124 0.00201 0.00542

134 5D-2 -0.00240 -0.00046 0.00579 0.01018 -0.00329

135 8 C 1S -0.00180 -0.00305 0.00043 0.00373 -0.00259

136 2S -0.00296 -0.00494 0.00064 0.00627 -0.00406

137 2PX -0.00097 -0.00178 -0.00169 0.00045 -0.00087

138 2PY 0.00861 0.00829 -0.00380 -0.01762 0.01201

139 2PZ -0.00132 0.00505 -0.00262 -0.00481 -0.00279

140 3S 0.00929 0.01479 -0.00110 -0.01836 0.01230

141 3PX -0.00122 -0.00308 -0.00179 0.00124 -0.00096

142 3PY 0.01231 0.01174 -0.00553 -0.02476 0.01709

143 3PZ -0.00171 0.00781 -0.00372 -0.00807 -0.00377

144 4S 0.01433 0.02208 -0.00063 -0.02654 0.01907

145 4PX -0.00277 -0.00826 -0.00535 0.00036 -0.00371

146 4PY 0.01550 0.01809 -0.00544 -0.03211 0.02192

147 4PZ -0.00274 0.00571 0.00058 -0.00132 -0.00548

148 5D 0 0.00132 0.00112 0.00102 -0.00077 0.00193

149 5D+1 -0.00126 -0.00050 -0.00023 0.00091 -0.00179

150 5D-1 0.00102 0.00012 0.00164 -0.00064 0.00143

151 5D+2 0.00110 0.00137 0.00084 -0.00072 0.00170

152 5D-2 -0.00003 -0.00091 0.00083 0.00155 -0.00005

153 9 C 1S 0.00112 -0.00076 0.00045 0.00098 0.00124

154 2S 0.00177 -0.00107 0.00077 0.00135 0.00211

155 2PX 0.00297 0.00145 0.00352 -0.00192 0.00434

156 2PY -0.00323 0.00303 -0.00130 -0.00412 -0.00411

157 2PZ -0.00925 -0.00313 -0.01214 0.00157 -0.01326

158 3S -0.00571 0.00185 -0.00314 -0.00281 -0.00751

159 3PX 0.00474 0.00149 0.00443 -0.00144 0.00689

160 3PY -0.00457 0.00399 -0.00243 -0.00545 -0.00575

161 3PZ -0.01354 -0.00461 -0.01814 0.00212 -0.01936

162 4S 0.00059 0.01693 0.00324 0.00406 0.00302

163 4PX 0.00718 0.00647 0.00897 -0.00298 0.01070

164 4PY -0.00328 0.00500 0.00006 0.00480 -0.00400

165 4PZ -0.01848 -0.00642 -0.02155 0.00496 -0.02690

166 5D 0 0.00009 -0.00003 0.00031 0.00006 0.00016

167 5D+1 -0.00015 -0.00099 -0.00109 0.00088 0.00000

168 5D-1 0.00004 -0.00038 0.00009 0.00072 0.00017

169 5D+2 -0.00007 -0.00070 -0.00030 0.00310 -0.00020

170 5D-2 -0.00147 -0.00104 -0.00011 0.00173 -0.00203

171 10 H 1S -0.00132 0.00140 0.00227 -0.00352 -0.00240

172 2S -0.00304 0.00138 0.00285 -0.00482 -0.00488

173 3S -0.00368 0.00148 0.00358 -0.00632 -0.00532

174 4PX -0.00042 0.00026 0.00030 -0.00071 -0.00050

175 4PY 0.00003 -0.00003 -0.00026 0.00036 0.00010

176 4PZ -0.00064 -0.00014 -0.00031 -0.00021 -0.00098

177 11 H 1S -0.00741 0.07303 -0.00664 0.08475 -0.00965

178 2S -0.00806 0.10182 -0.00615 0.10796 -0.01020

179 3S -0.00134 0.04467 0.00391 0.00474 -0.00169

180 4PX 0.00479 0.00228 0.00002 0.00262 0.00692

181 4PY 0.00226 -0.00631 0.00284 -0.01606 0.00316

182 4PZ -0.00015 0.00234 0.00460 0.00305 -0.00004

183 12 H 1S 0.01111 0.00605 -0.00825 -0.02151 0.01614

184 2S 0.01760 0.00625 -0.01586 -0.03067 0.02552

185 3S 0.01037 0.00094 -0.01475 -0.01438 0.01499

186 4PX 0.00124 0.00044 0.00425 0.00130 0.00187

187 4PY -0.00320 -0.00221 -0.00029 0.00372 -0.00453

188 4PZ 0.00123 -0.00084 0.00198 0.00158 0.00176

189 13 H 1S -0.00111 -0.00232 0.00347 0.00529 -0.00152

190 2S -0.00323 -0.00672 0.00576 0.01062 -0.00449

191 3S -0.00559 -0.00668 -0.00147 0.00554 -0.00808

192 4PX -0.00006 -0.00049 0.00026 0.00067 -0.00003

193 4PY 0.00065 0.00039 -0.00003 -0.00069 0.00092

194 4PZ -0.00032 0.00006 0.00000 0.00021 -0.00053

195 14 H 1S 0.00189 -0.00045 -0.00039 -0.00071 0.00247

196 2S 0.00294 -0.00142 -0.00153 -0.00094 0.00379

197 3S 0.00468 0.00530 0.00361 -0.00671 0.00649

198 4PX 0.00041 0.00030 0.00041 -0.00054 0.00060

199 4PY -0.00023 -0.00065 -0.00047 0.00093 -0.00037

200 4PZ -0.00089 -0.00023 -0.00103 -0.00009 -0.00126

106 107 108 109 110

106 3PY 0.17629

107 3PZ 0.00168 0.16083

108 4S 0.02904 0.00712 0.18382

109 4PX 0.00554 0.03575 0.00382 0.07704

110 4PY 0.10789 0.01616 0.00663 0.01272 0.07479

111 4PZ 0.01949 0.13075 0.01625 0.04880 0.02501

112 5D 0 -0.00132 0.00339 -0.00090 0.00162 -0.00120

113 5D+1 0.00687 0.00246 -0.00634 0.00130 0.00533

114 5D-1 -0.00308 -0.01049 -0.00341 -0.00177 -0.00308

115 5D+2 -0.01014 -0.00590 -0.00463 -0.00382 -0.00668

116 5D-2 -0.00295 0.00397 -0.00042 -0.00368 -0.00146

117 7 C 1S 0.01912 -0.01584 0.00506 0.00103 0.00287

118 2S 0.02835 -0.02329 0.00474 0.00309 0.00624

119 2PX -0.03054 0.08110 0.05350 0.01523 -0.01516

120 2PY -0.02547 0.00309 0.02571 -0.04786 -0.02745

121 2PZ 0.03464 0.03326 -0.01256 0.04236 0.02526

122 3S -0.07530 0.05931 -0.00343 -0.01484 -0.02451

123 3PX -0.04227 0.11735 0.07543 0.02463 -0.02033

124 3PY -0.03563 0.00429 0.03863 -0.07073 -0.03994

125 3PZ 0.04648 0.05144 -0.01524 0.06042 0.03379

126 4S -0.03475 0.04523 -0.04386 0.01822 0.00404

127 4PX -0.01102 0.10569 0.04378 0.03885 0.00221

128 4PY -0.03903 -0.02420 0.00982 -0.06249 -0.04107

129 4PZ 0.03512 0.05361 0.01802 0.04787 0.02382

130 5D 0 -0.00159 -0.00856 -0.00377 -0.00230 -0.00158

131 5D+1 0.00726 0.00444 -0.00025 0.00304 0.00491

132 5D-1 0.00219 0.00173 -0.00567 0.00661 0.00301

133 5D+2 -0.00796 0.00187 -0.00047 0.00268 -0.00477

134 5D-2 -0.00061 0.00857 0.00734 0.00017 0.00003

135 8 C 1S -0.00399 0.00059 0.00439 -0.00587 -0.00455

136 2S -0.00663 0.00073 0.00744 -0.00841 -0.00766

137 2PX -0.00226 -0.00251 -0.00884 -0.00302 -0.00257

138 2PY 0.01234 -0.00550 -0.02050 0.02009 0.01872

139 2PZ 0.00697 -0.00380 0.00154 0.00086 0.00582

140 3S 0.02046 -0.00051 -0.02213 0.02059 0.02313

141 3PX -0.00398 -0.00269 -0.01201 -0.00423 -0.00427

142 3PY 0.01749 -0.00801 -0.03002 0.03013 0.02779

143 3PZ 0.01080 -0.00536 0.00143 0.00193 0.00934

144 4S 0.03100 0.00022 -0.01853 0.03297 0.03320

145 4PX -0.01164 -0.00828 -0.01201 -0.00331 -0.00799

146 4PY 0.02648 -0.00759 -0.03229 0.03045 0.03082

147 4PZ 0.00761 0.00086 0.01260 0.00277 0.00577

148 5D 0 0.00163 0.00161 0.00019 0.00159 0.00136

149 5D+1 -0.00079 -0.00036 0.00085 -0.00228 -0.00117

150 5D-1 0.00023 0.00249 -0.00080 0.00283 0.00132

151 5D+2 0.00198 0.00132 0.00014 0.00035 0.00062

152 5D-2 -0.00130 0.00120 0.00169 0.00021 -0.00081

153 9 C 1S -0.00074 0.00069 -0.00275 0.00047 0.00223

154 2S -0.00131 0.00121 -0.00169 0.00128 0.00203

155 2PX 0.00244 0.00499 0.00414 0.00804 0.00447

156 2PY 0.00436 -0.00223 -0.00821 -0.00592 0.00228

157 2PZ -0.00495 -0.01830 -0.00132 -0.01906 -0.00688

158 3S 0.00305 -0.00494 -0.00382 -0.00695 -0.00214

159 3PX 0.00261 0.00620 0.00686 0.01150 0.00554

160 3PY 0.00572 -0.00403 -0.01225 -0.00911 0.00302

161 3PZ -0.00730 -0.02730 -0.00276 -0.02846 -0.01008

162 4S 0.02491 0.00602 -0.00088 -0.00578 0.01854

163 4PX 0.00979 0.01372 0.00080 0.01798 0.01460

164 4PY 0.00723 0.00019 -0.00016 -0.00841 0.00399

165 4PZ -0.01008 -0.03276 0.00345 -0.03499 -0.01514

166 5D 0 -0.00004 0.00050 -0.00001 0.00001 -0.00002

167 5D+1 -0.00140 -0.00161 -0.00206 -0.00168 -0.00116

168 5D-1 -0.00054 0.00016 -0.00063 -0.00091 -0.00040

169 5D+2 -0.00110 -0.00039 0.00262 -0.00074 -0.00128

170 5D-2 -0.00155 -0.00030 0.00219 -0.00294 -0.00229

171 10 H 1S 0.00210 0.00354 -0.00948 -0.00364 0.00588

172 2S 0.00219 0.00466 -0.01438 -0.00779 0.00932

173 3S 0.00224 0.00560 -0.01042 -0.00554 0.00601

174 4PX 0.00036 0.00044 0.00004 -0.00012 -0.00009

175 4PY -0.00006 -0.00037 0.00044 -0.00022 0.00007

176 4PZ -0.00027 -0.00051 -0.00051 -0.00110 0.00012

177 11 H 1S 0.10296 -0.00880 0.06677 -0.00570 0.06108

178 2S 0.14373 -0.00756 0.08814 -0.00355 0.08591

179 3S 0.06336 0.00634 0.01461 0.00806 0.03812

180 4PX 0.00327 0.00022 0.00166 0.00408 0.00243

181 4PY -0.00877 0.00422 -0.01069 0.00321 -0.00451

182 4PZ 0.00341 0.00696 0.00273 0.00166 0.00269

183 12 H 1S 0.00875 -0.01135 -0.02962 0.00486 0.00738

184 2S 0.00900 -0.02236 -0.04353 0.00540 0.00735

185 3S 0.00129 -0.02152 -0.01850 -0.00127 -0.00096

186 4PX 0.00070 0.00647 0.00087 0.00294 0.00114

187 4PY -0.00320 -0.00060 0.00235 -0.00409 -0.00308

188 4PZ -0.00115 0.00293 0.00371 0.00200 -0.00090

189 13 H 1S -0.00368 0.00523 0.00767 -0.00321 -0.00464

190 2S -0.01008 0.00849 0.01229 -0.00631 -0.01031

191 3S -0.00983 -0.00257 0.00644 -0.00915 -0.00997

192 4PX -0.00073 0.00038 0.00006 -0.00040 -0.00072

193 4PY 0.00057 -0.00002 -0.00113 0.00152 0.00115

194 4PZ 0.00001 0.00000 0.00100 -0.00034 -0.00015

195 14 H 1S -0.00052 -0.00046 0.00332 0.00496 0.00032

196 2S -0.00185 -0.00219 0.00338 0.00766 0.00000

197 3S 0.00780 0.00554 -0.00107 0.01163 0.00771

198 4PX 0.00046 0.00060 -0.00020 0.00079 0.00060

199 4PY -0.00094 -0.00071 0.00104 -0.00045 -0.00090

200 4PZ -0.00037 -0.00157 -0.00042 -0.00173 -0.00054

111 112 113 114 115

111 4PZ 0.11596

112 5D 0 0.00274 0.00079

113 5D+1 0.00277 -0.00048 0.00128

114 5D-1 -0.00853 -0.00012 -0.00019 0.00089

115 5D+2 -0.00724 -0.00006 -0.00065 0.00065 0.00206

116 5D-2 0.00116 0.00016 -0.00015 -0.00047 -0.00006

117 7 C 1S -0.00443 -0.00005 0.00273 0.00238 -0.00091

118 2S -0.00710 -0.00009 0.00347 0.00303 -0.00118

119 2PX 0.06774 0.00217 -0.00186 -0.00587 -0.00468

120 2PY -0.01392 0.00097 -0.00442 0.00065 0.00666

121 2PZ 0.03766 0.00452 -0.00108 -0.00133 -0.00129

122 3S 0.01943 0.00054 -0.00729 -0.00608 0.00273

123 3PX 0.09869 0.00319 -0.00261 -0.00841 -0.00668

124 3PY -0.02023 0.00146 -0.00644 0.00109 0.00986

125 3PZ 0.05673 0.00664 -0.00174 -0.00211 -0.00180

126 4S 0.02326 0.00007 -0.00131 -0.00485 -0.00080

127 4PX 0.09384 0.00437 -0.00148 -0.00772 -0.00759

128 4PY -0.04093 0.00091 -0.00532 0.00320 0.01046

129 4PZ 0.05771 0.00634 -0.00276 -0.00295 -0.00383

130 5D 0 -0.00763 -0.00013 -0.00013 0.00066 0.00063

131 5D+1 0.00533 0.00006 0.00065 -0.00038 -0.00093

132 5D-1 0.00292 0.00006 0.00042 -0.00009 -0.00035

133 5D+2 0.00130 0.00014 -0.00035 0.00016 0.00074

134 5D-2 0.00692 0.00006 -0.00012 -0.00067 -0.00047

135 8 C 1S -0.00038 0.00026 -0.00068 0.00021 0.00076

136 2S -0.00110 0.00047 -0.00104 0.00035 0.00127

137 2PX -0.00379 -0.00073 0.00034 0.00086 0.00201

138 2PY 0.00111 -0.00382 0.00496 -0.00057 -0.00376

139 2PZ -0.00101 0.00161 -0.00161 -0.00139 -0.00365

140 3S 0.00526 -0.00144 0.00283 -0.00108 -0.00356

141 3PX -0.00465 -0.00106 0.00050 0.00127 0.00297

142 3PY 0.00162 -0.00567 0.00728 -0.00095 -0.00566

143 3PZ -0.00124 0.00228 -0.00221 -0.00211 -0.00549

144 4S 0.01160 -0.00199 0.00463 -0.00165 -0.00719

145 4PX -0.00960 -0.00176 0.00105 0.00152 0.00259

146 4PY 0.00278 -0.00580 0.00813 -0.00036 -0.00493

147 4PZ 0.00527 0.00345 -0.00347 -0.00217 -0.00585

148 5D 0 0.00171 0.00010 0.00007 -0.00018 -0.00021

149 5D+1 -0.00106 0.00020 -0.00031 -0.00008 0.00011

150 5D-1 0.00254 -0.00008 0.00021 -0.00022 -0.00034

151 5D+2 0.00139 0.00023 -0.00014 0.00005 0.00021

152 5D-2 0.00101 0.00017 -0.00022 -0.00012 -0.00006

153 9 C 1S 0.00215 -0.00008 0.00047 -0.00014 -0.00070

154 2S 0.00334 -0.00017 0.00066 -0.00021 -0.00108

155 2PX 0.00554 -0.00022 0.00048 -0.00052 -0.00067

156 2PY -0.00272 0.00054 -0.00115 0.00012 0.00088

157 2PZ -0.02182 0.00009 -0.00123 -0.00016 0.00076

158 3S -0.01072 0.00064 -0.00177 0.00063 0.00335

159 3PX 0.00745 -0.00025 0.00050 -0.00061 -0.00088

160 3PY -0.00491 0.00070 -0.00166 0.00022 0.00151

161 3PZ -0.03268 0.00006 -0.00175 -0.00026 0.00114

162 4S 0.00030 -0.00039 0.00106 -0.00071 0.00245

163 4PX 0.01558 -0.00118 0.00254 -0.00131 -0.00286

164 4PY -0.00204 0.00118 -0.00185 -0.00041 0.00163

165 4PZ -0.03660 0.00125 -0.00351 0.00046 0.00151

166 5D 0 0.00033 -0.00004 0.00009 0.00001 0.00015

167 5D+1 -0.00257 -0.00044 0.00032 0.00023 0.00084

168 5D-1 -0.00057 -0.00005 0.00001 -0.00010 0.00024

169 5D+2 -0.00075 0.00060 -0.00077 -0.00003 0.00051

170 5D-2 -0.00086 0.00040 -0.00065 0.00013 0.00040

171 10 H 1S 0.00124 -0.00014 0.00000 -0.00024 0.00116

172 2S -0.00010 -0.00010 -0.00002 -0.00047 0.00227

173 3S 0.00172 -0.00035 0.00052 -0.00048 0.00135

174 4PX 0.00030 -0.00006 0.00010 -0.00003 -0.00007

175 4PY -0.00027 0.00001 0.00001 0.00000 -0.00003

176 4PZ -0.00041 0.00008 -0.00013 0.00004 -0.00003

177 11 H 1S 0.00367 -0.00265 0.00239 -0.00220 -0.00657

178 2S 0.01000 -0.00360 0.00388 -0.00331 -0.00995

179 3S 0.01494 -0.00170 0.00376 -0.00157 -0.00666

180 4PX 0.00176 -0.00004 0.00012 -0.00002 -0.00019

181 4PY 0.00304 0.00037 0.00016 0.00006 0.00020

182 4PZ 0.00607 0.00009 0.00016 -0.00054 -0.00044

183 12 H 1S -0.01349 0.00194 -0.00251 0.00143 0.00418

184 2S -0.02406 0.00235 -0.00337 0.00264 0.00646

185 3S -0.01955 0.00042 -0.00100 0.00209 0.00348

186 4PX 0.00545 0.00047 -0.00034 -0.00041 -0.00016

187 4PY -0.00167 -0.00003 -0.00025 0.00012 0.00049

188 4PZ 0.00352 0.00004 0.00015 -0.00020 -0.00051

189 13 H 1S 0.00282 0.00093 -0.00097 0.00010 0.00133

190 2S 0.00394 0.00142 -0.00181 0.00012 0.00214

191 3S -0.00505 0.00090 -0.00166 0.00048 0.00150

192 4PX 0.00004 -0.00003 0.00001 0.00012 0.00035

193 4PY 0.00032 -0.00031 0.00039 -0.00002 -0.00018

194 4PZ 0.00004 0.00021 -0.00022 -0.00006 -0.00014

195 14 H 1S -0.00045 -0.00096 0.00139 -0.00012 -0.00093

196 2S -0.00160 -0.00175 0.00224 -0.00005 -0.00141

197 3S 0.00845 -0.00146 0.00255 -0.00055 -0.00299

198 4PX 0.00073 -0.00004 0.00008 -0.00004 -0.00006

199 4PY -0.00107 0.00005 -0.00014 0.00001 0.00018

200 4PZ -0.00182 0.00001 -0.00011 0.00001 0.00007

116 117 118 119 120

116 5D-2 0.00152

117 7 C 1S -0.00458 0.65914

118 2S -0.00573 0.56099 0.49056

119 2PX 0.00414 -0.00083 -0.00097 0.08294

120 2PY 0.00048 -0.00285 -0.00378 0.00178 0.07292

121 2PZ -0.00500 -0.00209 -0.00323 -0.01009 -0.00659

122 3S 0.01147 -0.08700 -0.15017 0.00263 0.01057

123 3PX 0.00547 0.00003 -0.00045 0.11745 0.00187

124 3PY 0.00030 0.00070 -0.00023 0.00203 0.10918

125 3PZ -0.00704 -0.00146 -0.00270 -0.01014 -0.00710

126 4S 0.00914 -0.08518 -0.12548 -0.00778 -0.03838

127 4PX 0.00397 -0.00584 -0.00651 0.09027 -0.01776

128 4PY -0.00062 0.00387 0.00606 -0.01614 0.08904

129 4PZ -0.00430 0.00667 0.00738 0.01898 -0.00497

130 5D 0 -0.00010 0.00019 -0.00020 -0.00649 0.00082

131 5D+1 -0.00002 0.00048 0.00255 0.00308 -0.00428

132 5D-1 -0.00019 -0.00098 -0.00129 -0.00179 -0.00665

133 5D+2 -0.00037 0.00063 0.00028 0.00066 0.00242

134 5D-2 0.00040 -0.00025 -0.00086 0.00727 0.00167

135 8 C 1S -0.00038 0.00142 0.00216 -0.00003 0.01024

136 2S -0.00073 0.00216 0.00332 -0.00009 0.01619

137 2PX -0.00032 0.00219 0.00346 -0.00683 0.01270

138 2PY 0.00236 -0.00945 -0.01493 -0.00993 -0.04858

139 2PZ 0.00117 -0.00396 -0.00617 0.01529 -0.02358

140 3S 0.00225 -0.00497 -0.00748 0.00037 -0.04347

141 3PX -0.00058 0.00323 0.00530 -0.00958 0.01936

142 3PY 0.00379 -0.01473 -0.02363 -0.01402 -0.07351

143 3PZ 0.00178 -0.00606 -0.00976 0.02193 -0.03635

144 4S 0.00312 -0.00623 -0.01077 0.00190 -0.06876

145 4PX 0.00007 -0.00136 -0.00195 -0.01353 0.01173

146 4PY 0.00234 -0.00659 -0.01199 -0.02101 -0.06469

147 4PZ 0.00169 -0.00414 -0.00675 0.03034 -0.03009

148 5D 0 -0.00005 -0.00008 -0.00013 0.00057 -0.00162

149 5D+1 -0.00008 0.00062 0.00100 0.00153 0.00179

150 5D-1 0.00027 -0.00125 -0.00214 0.00080 -0.00358

151 5D+2 -0.00035 0.00146 0.00248 0.00002 0.00297

152 5D-2 0.00010 0.00009 0.00016 0.00223 0.00004

153 9 C 1S 0.00005 -0.00098 -0.00153 0.00077 -0.00387

154 2S 0.00002 -0.00150 -0.00241 0.00132 -0.00622

155 2PX 0.00114 -0.00256 -0.00416 0.00105 -0.01033

156 2PY 0.00012 0.00233 0.00376 0.00091 0.00677

157 2PZ -0.00037 0.00039 0.00068 -0.00068 0.00199

158 3S 0.00016 0.00410 0.00691 -0.00487 0.02009

159 3PX 0.00149 -0.00362 -0.00563 0.00171 -0.01405

160 3PY 0.00014 0.00292 0.00497 0.00048 0.01020

161 3PZ -0.00062 0.00055 0.00105 -0.00114 0.00271

162 4S -0.00064 0.00350 0.00698 -0.00920 0.01425

163 4PX 0.00133 -0.00768 -0.01087 0.00230 -0.02512

164 4PY 0.00048 0.00224 0.00431 0.00403 0.01121

165 4PZ -0.00061 0.00247 0.00390 0.00066 0.01241

166 5D 0 0.00001 -0.00003 0.00003 -0.00059 0.00090

167 5D+1 -0.00023 0.00062 0.00097 -0.00365 0.00386

168 5D-1 -0.00008 0.00024 0.00033 0.00004 0.00086

169 5D+2 0.00032 0.00040 0.00065 0.00203 0.00291

170 5D-2 -0.00032 0.00078 0.00139 0.00084 0.00545

171 10 H 1S 0.00081 -0.00059 -0.00093 -0.00077 0.00127

172 2S 0.00166 -0.00086 -0.00145 -0.00134 0.00325

173 3S 0.00131 -0.00096 -0.00157 -0.00085 0.00143

174 4PX -0.00002 -0.00006 -0.00007 -0.00005 -0.00008

175 4PY -0.00003 0.00000 0.00001 -0.00002 -0.00010

176 4PZ 0.00005 -0.00007 -0.00012 0.00043 0.00010

177 11 H 1S -0.00158 0.00447 0.00733 -0.00859 -0.00706

178 2S -0.00230 0.00596 0.01026 -0.00993 -0.01510

179 3S -0.00117 0.00408 0.00586 -0.00440 -0.02135

180 4PX -0.00048 0.00086 0.00127 -0.00059 -0.00250

181 4PY 0.00009 0.00062 0.00044 0.00150 -0.00228

182 4PZ 0.00011 -0.00043 -0.00059 0.00343 -0.00001

183 12 H 1S -0.00290 -0.02295 -0.03587 -0.04776 0.01462

184 2S -0.00468 -0.03473 -0.05022 -0.06992 0.02235

185 3S -0.00285 -0.00309 -0.00551 -0.03186 0.01148

186 4PX -0.00007 -0.00402 -0.00507 0.00163 0.00066

187 4PY 0.00009 0.00186 0.00225 0.00124 0.00519

188 4PZ 0.00011 0.00541 0.00670 0.00624 -0.00203

189 13 H 1S -0.00075 0.00312 0.00498 0.00169 0.01420

190 2S -0.00113 0.00377 0.00604 0.00440 0.02335

191 3S -0.00050 0.00181 0.00281 0.00127 0.01556

192 4PX -0.00014 0.00048 0.00072 -0.00095 0.00265

193 4PY 0.00013 -0.00083 -0.00120 -0.00104 -0.00282

194 4PZ 0.00001 -0.00005 -0.00009 0.00107 -0.00025

195 14 H 1S -0.00010 -0.00202 -0.00325 -0.00307 -0.00901

196 2S -0.00007 -0.00370 -0.00580 -0.00549 -0.01503

197 3S -0.00018 -0.00272 -0.00440 -0.00093 -0.02024

198 4PX 0.00007 -0.00001 -0.00008 0.00003 -0.00092

199 4PY 0.00005 -0.00011 -0.00014 -0.00002 0.00061

200 4PZ -0.00004 0.00010 0.00016 -0.00010 0.00039

121 122 123 124 125

121 2PZ 0.07468

122 3S 0.00821 0.45392

123 3PX -0.01053 0.00408 0.16660

124 3PY -0.00843 0.00816 0.00199 0.16374

125 3PZ 0.10615 0.00814 -0.00900 -0.00854 0.15125

126 4S 0.01181 0.31803 -0.01054 -0.06305 0.01253

127 4PX 0.01712 0.01028 0.12907 -0.02713 0.02844

128 4PY -0.01103 -0.01388 -0.02385 0.13343 -0.01347

129 4PZ 0.07806 -0.01061 0.03070 -0.00574 0.11256

130 5D 0 -0.00108 0.00210 -0.00932 0.00121 -0.00185

131 5D+1 0.00086 -0.01262 0.00439 -0.00620 0.00132

132 5D-1 0.00400 0.00252 -0.00230 -0.00999 0.00542

133 5D+2 0.00209 0.00152 0.00115 0.00370 0.00319

134 5D-2 -0.00103 0.00387 0.01029 0.00246 -0.00114

135 8 C 1S 0.00293 -0.00499 -0.00017 0.01589 0.00455

136 2S 0.00451 -0.00752 -0.00017 0.02552 0.00730

137 2PX -0.00187 -0.00959 -0.00891 0.01877 -0.00222

138 2PY -0.02269 0.03959 -0.01476 -0.07337 -0.03479

139 2PZ 0.00628 0.01644 0.02182 -0.03626 0.00894

140 3S -0.01116 0.01530 0.00019 -0.06975 -0.01907

141 3PX -0.00263 -0.01520 -0.01249 0.02872 -0.00304

142 3PY -0.03449 0.06381 -0.02095 -0.11120 -0.05288

143 3PZ 0.00902 0.02700 0.03127 -0.05589 0.01270

144 4S -0.01441 0.02888 0.00204 -0.10623 -0.02430

145 4PX -0.01120 0.00475 -0.01887 0.01724 -0.01611

146 4PY -0.03011 0.03535 -0.03052 -0.09737 -0.04652

147 4PZ 0.01325 0.01932 0.04311 -0.04626 0.01922

148 5D 0 0.00172 0.00033 0.00086 -0.00236 0.00244

149 5D+1 0.00038 -0.00286 0.00215 0.00255 0.00069

150 5D-1 -0.00012 0.00627 0.00113 -0.00549 -0.00032

151 5D+2 0.00293 -0.00738 0.00020 0.00454 0.00434

152 5D-2 0.00010 -0.00046 0.00311 0.00005 0.00024

153 9 C 1S -0.00090 0.00403 0.00102 -0.00505 -0.00140

154 2S -0.00140 0.00670 0.00168 -0.00852 -0.00226

155 2PX -0.00244 0.01234 0.00094 -0.01459 -0.00368

156 2PY 0.00366 -0.01103 0.00167 0.00954 0.00558

157 2PZ -0.00450 -0.00249 -0.00142 0.00235 -0.00619

158 3S 0.00364 -0.02055 -0.00617 0.02899 0.00625

159 3PX -0.00313 0.01598 0.00167 -0.01974 -0.00471

160 3PY 0.00490 -0.01537 0.00123 0.01441 0.00749

161 3PZ -0.00672 -0.00411 -0.00226 0.00309 -0.00926

162 4S 0.00831 -0.02411 -0.01226 0.02134 0.01229

163 4PX -0.00437 0.02570 0.00294 -0.03734 -0.00724

164 4PY 0.00714 -0.01446 0.00604 0.01615 0.01092

165 4PZ -0.00368 -0.01082 0.00004 0.01768 -0.00459

166 5D 0 -0.00002 -0.00026 -0.00085 0.00140 -0.00004

167 5D+1 -0.00199 -0.00276 -0.00514 0.00576 -0.00286

168 5D-1 0.00005 -0.00082 0.00011 0.00124 0.00014

169 5D+2 0.00217 -0.00167 0.00293 0.00418 0.00329

170 5D-2 0.00225 -0.00406 0.00122 0.00834 0.00347

171 10 H 1S -0.00254 0.00266 -0.00121 0.00178 -0.00369

172 2S -0.00478 0.00443 -0.00217 0.00449 -0.00690

173 3S -0.00487 0.00456 -0.00144 0.00178 -0.00704

174 4PX -0.00018 0.00012 -0.00007 -0.00012 -0.00026

175 4PY 0.00007 -0.00007 -0.00003 -0.00015 0.00009

176 4PZ 0.00008 0.00047 0.00062 0.00017 0.00009

177 11 H 1S 0.00381 -0.02290 -0.01215 -0.00960 0.00385

178 2S 0.00678 -0.03364 -0.01391 -0.02122 0.00745

179 3S 0.00435 -0.01587 -0.00602 -0.03107 0.00493

180 4PX 0.00297 -0.00341 -0.00062 -0.00360 0.00416

181 4PY 0.00139 0.00055 0.00222 -0.00343 0.00213

182 4PZ 0.00183 0.00131 0.00498 0.00001 0.00274

183 12 H 1S 0.04966 0.09777 -0.06543 0.02147 0.06831

184 2S 0.06689 0.12428 -0.09597 0.03301 0.09191

185 3S 0.02095 0.01695 -0.04413 0.01743 0.02872

186 4PX 0.00616 0.01011 0.00263 0.00093 0.00883

187 4PY -0.00277 -0.00392 0.00160 0.00780 -0.00369

188 4PZ -0.00275 -0.01289 0.00874 -0.00290 -0.00355

189 13 H 1S 0.00451 -0.01396 0.00207 0.02107 0.00657

190 2S 0.00625 -0.01685 0.00581 0.03471 0.00937

191 3S 0.00174 -0.00708 0.00148 0.02338 0.00297

192 4PX 0.00002 -0.00189 -0.00135 0.00395 0.00003

193 4PY -0.00182 0.00283 -0.00156 -0.00429 -0.00281

194 4PZ 0.00082 0.00027 0.00147 -0.00046 0.00116

195 14 H 1S -0.00552 0.00917 -0.00466 -0.01316 -0.00827

196 2S -0.01055 0.01581 -0.00831 -0.02206 -0.01577

197 3S -0.00527 0.01196 -0.00152 -0.02992 -0.00833

198 4PX -0.00015 0.00045 0.00002 -0.00132 -0.00024

199 4PY -0.00022 0.00034 -0.00006 0.00090 -0.00027

200 4PZ -0.00034 -0.00049 -0.00017 0.00052 -0.00047

126 127 128 129 130

126 4S 0.26222

127 4PX 0.01152 0.11542

128 4PY -0.06460 -0.04385 0.11872

129 4PZ -0.00801 0.05147 -0.01537 0.09692

130 5D 0 0.00169 -0.00803 0.00267 -0.00334 0.00073

131 5D+1 -0.00590 0.00513 -0.00588 0.00249 -0.00049

132 5D-1 0.00670 0.00100 -0.00783 0.00294 0.00001

133 5D+2 -0.00034 0.00010 0.00319 0.00184 0.00007

134 5D-2 0.00097 0.00748 -0.00050 0.00142 -0.00059

135 8 C 1S -0.00581 -0.00284 0.00709 0.00358 0.00027

136 2S -0.01009 -0.00464 0.01280 0.00584 0.00042

137 2PX -0.01412 -0.01419 0.02155 -0.00933 0.00060

138 2PY 0.06431 -0.00908 -0.06232 -0.03518 0.00107

139 2PZ 0.01818 0.02954 -0.03042 0.01593 -0.00234

140 3S 0.02689 0.01325 -0.03832 -0.01576 -0.00126

141 3PX -0.02184 -0.02054 0.03208 -0.01315 0.00089

142 3PY 0.09966 -0.01231 -0.09433 -0.05296 0.00157

143 3PZ 0.03001 0.04326 -0.04700 0.02287 -0.00342

144 4S 0.05757 0.01847 -0.07944 -0.01734 -0.00084

145 4PX -0.00100 -0.02616 0.02022 -0.02314 0.00186

146 4PY 0.07584 -0.02215 -0.08109 -0.05061 0.00194

147 4PZ 0.01838 0.05324 -0.04123 0.03373 -0.00371

148 5D 0 0.00145 0.00180 -0.00257 0.00219 -0.00014

149 5D+1 -0.00436 0.00202 0.00296 0.00153 -0.00029

150 5D-1 0.00697 0.00184 -0.00507 -0.00034 -0.00003

151 5D+2 -0.00756 0.00033 0.00459 0.00338 -0.00010

152 5D-2 -0.00094 0.00285 -0.00019 0.00136 -0.00016

153 9 C 1S 0.00801 0.00264 -0.00836 -0.00091 0.00001

154 2S 0.01085 0.00337 -0.01249 -0.00065 0.00006

155 2PX 0.02138 0.00298 -0.01903 -0.00167 0.00054

156 2PY -0.01838 0.00324 0.01623 0.00422 -0.00055

157 2PZ -0.00794 0.00040 0.00663 -0.00249 -0.00113

158 3S -0.02660 -0.00849 0.03599 -0.00103 -0.00019

159 3PX 0.02852 0.00431 -0.02617 -0.00163 0.00080

160 3PY -0.02593 0.00346 0.02401 0.00524 -0.00073

161 3PZ -0.01218 0.00048 0.00990 -0.00395 -0.00174

162 4S -0.01834 -0.01055 0.01995 0.00205 -0.00030

163 4PX 0.03972 0.00654 -0.03893 -0.00606 0.00028

164 4PY -0.02187 0.00794 0.01991 0.01021 -0.00098

165 4PZ -0.02583 0.00213 0.02239 0.00310 -0.00147

166 5D 0 -0.00017 -0.00103 0.00088 -0.00033 0.00010

167 5D+1 -0.00345 -0.00633 0.00628 -0.00454 0.00030

168 5D-1 -0.00134 0.00001 0.00158 -0.00016 -0.00015

169 5D+2 -0.00372 0.00315 0.00442 0.00386 -0.00014

170 5D-2 -0.00710 0.00070 0.00692 0.00380 -0.00007

171 10 H 1S 0.00769 0.00013 0.00221 -0.00448 -0.00010

172 2S 0.01186 -0.00013 0.00518 -0.00782 -0.00006

173 3S 0.00904 -0.00104 0.00201 -0.00748 -0.00004

174 4PX -0.00009 -0.00025 -0.00019 -0.00024 -0.00001

175 4PY -0.00028 -0.00003 -0.00020 0.00022 0.00002

176 4PZ -0.00010 0.00066 -0.00001 0.00066 0.00000

177 11 H 1S -0.01894 -0.00597 -0.01850 0.00782 -0.00127

178 2S -0.02439 -0.00426 -0.03234 0.01260 -0.00212

179 3S -0.00107 0.00160 -0.03220 0.00484 -0.00118

180 4PX -0.00121 0.00058 -0.00317 0.00305 -0.00009

181 4PY 0.00297 0.00276 -0.00203 0.00107 -0.00003

182 4PZ 0.00098 0.00467 -0.00156 0.00295 -0.00043

183 12 H 1S 0.06575 -0.03903 0.02092 0.03303 0.00345

184 2S 0.08141 -0.06032 0.03391 0.04221 0.00512

185 3S 0.00684 -0.03222 0.02082 0.01015 0.00235

186 4PX 0.00669 0.00411 -0.00057 0.00697 -0.00024

187 4PY -0.00621 -0.00095 0.00658 -0.00230 0.00002

188 4PZ -0.00868 0.00634 -0.00292 -0.00016 -0.00049

189 13 H 1S -0.02052 0.00177 0.01924 0.00858 -0.00005

190 2S -0.02982 0.00371 0.03100 0.01269 -0.00020

191 3S -0.01681 -0.00078 0.02036 0.00532 0.00014

192 4PX -0.00285 -0.00187 0.00382 -0.00045 0.00013

193 4PY 0.00429 -0.00127 -0.00361 -0.00285 0.00013

194 4PZ -0.00037 0.00202 -0.00034 0.00192 -0.00013

195 14 H 1S 0.01762 -0.00539 -0.01603 -0.00821 0.00039

196 2S 0.02842 -0.00973 -0.02533 -0.01560 0.00071

197 3S 0.02419 -0.00023 -0.03071 -0.00783 0.00003

198 4PX 0.00124 0.00022 -0.00141 -0.00021 0.00003

199 4PY 0.00030 -0.00025 0.00063 -0.00015 0.00001

200 4PZ -0.00119 -0.00003 0.00103 -0.00019 -0.00009

131 132 133 134 135

131 5D+1 0.00098

132 5D-1 0.00027 0.00095

133 5D+2 -0.00041 0.00001 0.00083

134 5D-2 0.00021 -0.00031 0.00002 0.00087

135 8 C 1S -0.00062 -0.00149 0.00118 0.00020 0.65900

136 2S -0.00100 -0.00251 0.00203 0.00035 0.56074

137 2PX -0.00188 -0.00012 0.00067 -0.00200 -0.00062

138 2PY 0.00125 0.00413 -0.00163 -0.00087 0.00289

139 2PZ 0.00109 0.00039 -0.00307 -0.00052 0.00229

140 3S 0.00291 0.00735 -0.00609 -0.00108 -0.08612

141 3PX -0.00266 -0.00024 0.00113 -0.00272 0.00099

142 3PY 0.00182 0.00628 -0.00257 -0.00123 -0.00028

143 3PZ 0.00169 0.00075 -0.00460 -0.00071 0.00116

144 4S 0.00431 0.00798 -0.00605 -0.00027 -0.08384

145 4PX -0.00247 0.00012 0.00130 -0.00184 -0.00531

146 4PY 0.00218 0.00613 -0.00210 -0.00155 -0.00487

147 4PZ 0.00213 0.00028 -0.00422 0.00052 -0.00488

148 5D 0 0.00020 0.00012 -0.00002 0.00009 0.00046

149 5D+1 0.00007 -0.00026 -0.00020 0.00003 -0.00017

150 5D-1 0.00014 0.00045 -0.00007 0.00015 -0.00117

151 5D+2 0.00005 -0.00003 0.00011 -0.00011 0.00040

152 5D-2 0.00013 -0.00006 0.00003 0.00022 0.00015

153 9 C 1S 0.00008 0.00008 0.00011 0.00037 0.00536

154 2S 0.00027 0.00015 0.00012 0.00067 0.00843

155 2PX 0.00099 0.00033 0.00099 0.00132 0.01772

156 2PY -0.00038 0.00027 -0.00070 -0.00131 -0.00679

157 2PZ -0.00015 -0.00206 -0.00156 -0.00082 0.00435

158 3S -0.00137 -0.00070 -0.00005 -0.00219 -0.02150

159 3PX 0.00137 0.00039 0.00149 0.00188 0.03052

160 3PY -0.00062 0.00032 -0.00096 -0.00192 -0.01095

161 3PZ -0.00022 -0.00304 -0.00235 -0.00130 0.00730

162 4S -0.00020 -0.00047 0.00003 -0.00113 0.00164

163 4PX 0.00155 0.00229 0.00013 0.00144 0.00327

164 4PY -0.00026 -0.00036 -0.00074 -0.00094 -0.00359

165 4PZ -0.00071 -0.00385 -0.00247 -0.00122 0.00369

166 5D 0 -0.00003 -0.00002 0.00014 0.00005 0.00136

167 5D+1 -0.00032 -0.00011 0.00032 -0.00025 -0.00191

168 5D-1 -0.00002 -0.00011 0.00001 -0.00006 0.00027

169 5D+2 -0.00009 -0.00013 -0.00007 -0.00002 -0.00420

170 5D-2 -0.00013 -0.00057 0.00025 0.00014 0.00336

171 10 H 1S 0.00028 0.00009 -0.00030 0.00007 -0.00107

172 2S 0.00031 0.00007 -0.00046 0.00006 -0.00156

173 3S 0.00028 0.00016 -0.00040 0.00008 -0.00150

174 4PX 0.00002 0.00000 -0.00003 0.00001 -0.00001

175 4PY -0.00002 0.00000 -0.00001 0.00000 -0.00008

176 4PZ 0.00001 -0.00007 -0.00007 0.00004 0.00004

177 11 H 1S 0.00295 -0.00079 -0.00511 0.00145 -0.00221

178 2S 0.00457 -0.00057 -0.00722 0.00200 -0.00420

179 3S 0.00318 0.00123 -0.00347 0.00052 -0.00245

180 4PX 0.00020 0.00033 0.00008 -0.00006 -0.00007

181 4PY 0.00005 0.00044 0.00046 -0.00016 0.00010

182 4PZ 0.00030 0.00006 -0.00006 0.00043 -0.00009

183 12 H 1S -0.00494 0.00231 0.00220 -0.00334 0.00311

184 2S -0.00707 0.00303 0.00332 -0.00512 0.00373

185 3S -0.00263 0.00062 0.00149 -0.00275 0.00176

186 4PX -0.00013 0.00024 0.00021 0.00023 0.00016

187 4PY -0.00020 -0.00061 0.00016 0.00017 0.00097

188 4PZ 0.00063 -0.00012 -0.00006 0.00039 -0.00011

189 13 H 1S 0.00062 -0.00031 0.00039 0.00147 -0.02311

190 2S 0.00055 -0.00089 0.00087 0.00219 -0.03480

191 3S -0.00040 -0.00139 0.00063 0.00069 -0.00323

192 4PX -0.00008 -0.00001 0.00017 0.00005 -0.00473

193 4PY 0.00011 0.00033 -0.00006 0.00004 -0.00305

194 4PZ 0.00013 -0.00001 -0.00018 0.00013 -0.00410

195 14 H 1S 0.00040 -0.00005 0.00028 0.00062 0.00443

196 2S 0.00049 0.00011 0.00039 0.00079 0.00577

197 3S 0.00117 0.00121 -0.00036 0.00068 0.00421

198 4PX 0.00009 0.00009 0.00005 0.00006 0.00114

199 4PY -0.00004 -0.00016 0.00005 0.00004 -0.00033

200 4PZ -0.00003 -0.00015 -0.00014 -0.00010 0.00016

136 137 138 139 140

136 2S 0.49015

137 2PX -0.00053 0.08861

138 2PY 0.00395 0.00197 0.07084

139 2PZ 0.00339 0.00511 -0.00538 0.07205

140 3S -0.14882 0.00177 -0.01067 -0.00882 0.44980

141 3PX 0.00083 0.12356 0.00228 0.00370 0.00231

142 3PY 0.00071 0.00153 0.10592 -0.00734 -0.00796

143 3PZ 0.00245 0.00343 -0.00672 0.10489 -0.00895

144 4S -0.12344 -0.02437 0.03094 -0.00549 0.31213

145 4PX -0.00503 0.07230 0.01364 -0.01687 0.00477

146 4PY -0.00681 0.01050 0.09557 -0.02412 0.01406

147 4PZ -0.00568 -0.01702 -0.01803 0.09254 0.00886

148 5D 0 0.00111 -0.00418 0.00044 0.00118 -0.00422

149 5D+1 -0.00171 -0.00392 -0.00597 0.00422 0.00920

150 5D-1 -0.00215 -0.00300 0.00389 -0.00187 0.00665

151 5D+2 -0.00074 0.00445 -0.00390 -0.00149 0.00644

152 5D-2 0.00009 -0.00683 -0.00061 -0.00051 0.00019

153 9 C 1S 0.00842 -0.01776 0.00654 -0.00325 -0.02173

154 2S 0.01290 -0.02830 0.01040 -0.00521 -0.03302

155 2PX 0.02832 -0.05274 0.02693 -0.02139 -0.08134

156 2PY -0.01087 0.01856 -0.00096 0.00756 0.03165

157 2PZ 0.00689 -0.03123 -0.02107 0.04516 -0.01903

158 3S -0.03251 0.07969 -0.03032 0.01401 0.08418

159 3PX 0.04472 -0.07469 0.03728 -0.03062 -0.11595

160 3PY -0.01637 0.02647 -0.00184 0.01104 0.04407

161 3PZ 0.01081 -0.04563 -0.03141 0.06853 -0.02749

162 4S 0.00054 0.05933 -0.01414 0.00650 0.00514

163 4PX 0.00712 -0.03465 0.03942 -0.02417 -0.02752

164 4PY -0.00610 0.01814 -0.01109 0.01611 0.01970

165 4PZ 0.00552 -0.04138 -0.04509 0.06823 -0.01388

166 5D 0 0.00169 -0.00094 0.00068 -0.00288 -0.00320

167 5D+1 -0.00244 0.00380 -0.00033 -0.00630 0.00515

168 5D-1 0.00039 -0.00171 -0.00156 0.00230 -0.00082

169 5D+2 -0.00532 0.00199 -0.00650 0.00336 0.01095

170 5D-2 0.00417 -0.00635 -0.00592 -0.00067 -0.00805

171 10 H 1S -0.00169 0.00256 0.00020 0.00050 0.00498

172 2S -0.00266 0.00512 -0.00048 0.00101 0.00866

173 3S -0.00260 0.00695 0.00219 -0.00111 0.00835

174 4PX -0.00001 0.00018 0.00036 -0.00030 0.00005

175 4PY -0.00011 0.00011 -0.00017 0.00023 0.00024

176 4PZ 0.00007 -0.00052 -0.00081 0.00110 -0.00027

177 11 H 1S -0.00357 -0.00238 0.00888 0.00528 0.01041

178 2S -0.00655 -0.00363 0.01528 0.00946 0.01816

179 3S -0.00399 -0.00357 0.01896 0.00411 0.01104

180 4PX -0.00016 -0.00013 0.00070 0.00007 0.00065

181 4PY 0.00011 -0.00024 0.00071 -0.00014 -0.00022

182 4PZ -0.00011 -0.00017 -0.00015 -0.00016 0.00028

183 12 H 1S 0.00496 0.00464 -0.01227 -0.00679 -0.01383

184 2S 0.00596 0.01025 -0.01853 -0.01199 -0.01650

185 3S 0.00274 0.00607 -0.01119 -0.00650 -0.00700

186 4PX 0.00025 -0.00044 -0.00215 0.00115 -0.00065

187 4PY 0.00142 0.00090 -0.00324 -0.00189 -0.00344

188 4PZ -0.00015 -0.00044 0.00017 0.00101 0.00041

189 13 H 1S -0.03611 -0.05397 -0.02897 -0.03368 0.09828

190 2S -0.05041 -0.07617 -0.04239 -0.04538 0.12490

191 3S -0.00575 -0.03156 -0.02070 -0.01220 0.01763

192 4PX -0.00597 -0.00080 -0.00248 -0.00585 0.01192

193 4PY -0.00374 -0.00330 0.00408 -0.00401 0.00689

194 4PZ -0.00507 -0.00586 -0.00336 0.00116 0.00963

195 14 H 1S 0.00726 -0.00971 0.00463 -0.00212 -0.02253

196 2S 0.01011 -0.01360 0.01164 -0.00461 -0.03345

197 3S 0.00622 -0.01151 0.02112 -0.00463 -0.01726

198 4PX 0.00153 -0.00274 0.00229 -0.00187 -0.00348

199 4PY -0.00003 -0.00111 -0.00200 0.00118 -0.00159

200 4PZ 0.00019 -0.00184 -0.00158 0.00343 -0.00030

141 142 143 144 145

141 3PX 0.17253

142 3PY 0.00136 0.15864

143 3PZ -0.00024 -0.00889 0.15298

144 4S -0.03487 0.05254 -0.00343 0.25729

145 4PX 0.10153 0.01920 -0.02764 -0.00930 0.07009

146 4PY 0.01479 0.14269 -0.03361 0.05936 0.02949

147 4PZ -0.02800 -0.02522 0.13559 0.01090 -0.04284

148 5D 0 -0.00593 0.00066 0.00189 -0.00108 -0.00380

149 5D+1 -0.00562 -0.00868 0.00631 0.00355 -0.00537

150 5D-1 -0.00415 0.00604 -0.00247 0.00797 -0.00137

151 5D+2 0.00639 -0.00587 -0.00248 0.00033 0.00280

152 5D-2 -0.00951 -0.00073 -0.00055 0.00159 -0.00574

153 9 C 1S -0.02888 0.01056 -0.00545 0.00501 -0.00668

154 2S -0.04316 0.01628 -0.00770 0.00613 -0.01217

155 2PX -0.07225 0.03967 -0.02943 -0.03579 -0.03978

156 2PY 0.02576 -0.00049 0.00930 0.01060 0.01027

157 2PZ -0.04575 -0.03100 0.06717 -0.02215 -0.04116

158 3S 0.11275 -0.04584 0.01744 -0.01498 0.03925

159 3PX -0.10223 0.05486 -0.04222 -0.05171 -0.05638

160 3PY 0.03674 -0.00141 0.01365 0.01392 0.01455

161 3PZ -0.06699 -0.04623 0.10188 -0.03285 -0.06085

162 4S 0.08357 -0.02256 0.00727 -0.04250 0.03034

163 4PX -0.04730 0.05901 -0.03334 0.01040 -0.02003

164 4PY 0.02473 -0.01572 0.02174 -0.00297 0.00492

165 4PZ -0.06088 -0.06630 0.10082 -0.02921 -0.05898

166 5D 0 -0.00124 0.00095 -0.00418 -0.00101 0.00054

167 5D+1 0.00556 -0.00058 -0.00929 0.00083 0.00513

168 5D-1 -0.00251 -0.00229 0.00340 -0.00153 -0.00217

169 5D+2 0.00259 -0.00935 0.00469 0.00501 0.00016

170 5D-2 -0.00866 -0.00891 -0.00099 -0.00843 -0.00656

171 10 H 1S 0.00365 0.00030 0.00079 0.00329 0.00286

172 2S 0.00714 -0.00052 0.00149 0.00516 0.00479

173 3S 0.00967 0.00334 -0.00163 0.00792 0.00784

174 4PX 0.00024 0.00053 -0.00042 0.00048 0.00035

175 4PY 0.00015 -0.00024 0.00033 0.00080 0.00021

176 4PZ -0.00077 -0.00119 0.00160 -0.00003 -0.00076

177 11 H 1S -0.00384 0.01312 0.00804 0.01753 -0.00637

178 2S -0.00585 0.02262 0.01441 0.02883 -0.00961

179 3S -0.00529 0.02791 0.00673 0.02344 -0.00334

180 4PX -0.00018 0.00100 0.00012 0.00127 -0.00035

181 4PY -0.00030 0.00104 -0.00017 0.00030 0.00013

182 4PZ -0.00021 -0.00023 -0.00022 0.00042 -0.00061

183 12 H 1S 0.00655 -0.01837 -0.00973 -0.02071 0.00540

184 2S 0.01458 -0.02788 -0.01748 -0.02977 0.01135

185 3S 0.00869 -0.01709 -0.00975 -0.01635 0.00591

186 4PX -0.00063 -0.00315 0.00169 -0.00110 -0.00120

187 4PY 0.00139 -0.00493 -0.00293 -0.00507 0.00095

188 4PZ -0.00061 0.00022 0.00140 0.00118 -0.00099

189 13 H 1S -0.07361 -0.04113 -0.04642 0.06660 -0.03932

190 2S -0.10391 -0.06046 -0.06253 0.08236 -0.05637

191 3S -0.04326 -0.03025 -0.01672 0.00711 -0.02562

192 4PX -0.00083 -0.00355 -0.00843 0.00695 0.00073

193 4PY -0.00447 0.00626 -0.00554 0.00792 -0.00092

194 4PZ -0.00823 -0.00473 0.00200 0.00697 -0.00557

195 14 H 1S -0.01316 0.00590 -0.00169 -0.01522 -0.00913

196 2S -0.01838 0.01595 -0.00467 -0.01990 -0.01108

197 3S -0.01589 0.03085 -0.00535 0.00318 -0.00560

198 4PX -0.00373 0.00341 -0.00265 -0.00083 -0.00175

199 4PY -0.00151 -0.00305 0.00183 -0.00304 -0.00205

200 4PZ -0.00274 -0.00230 0.00506 -0.00092 -0.00258

146 147 148 149 150

146 4PY 0.13883

147 4PZ -0.04817 0.13082

148 5D 0 -0.00027 0.00226 0.00036

149 5D+1 -0.00905 0.00723 0.00015 0.00135

150 5D-1 0.00547 -0.00155 0.00011 -0.00021 0.00057

151 5D+2 -0.00447 -0.00183 -0.00030 0.00023 -0.00020

152 5D-2 -0.00242 0.00177 0.00033 0.00048 0.00029

153 9 C 1S 0.00424 -0.00106 0.00257 -0.00098 0.00133

154 2S 0.00709 -0.00135 0.00329 -0.00129 0.00178

155 2PX 0.02884 -0.01867 0.00261 -0.00201 0.00280

156 2PY -0.01158 0.01158 -0.00084 0.00237 0.00032

157 2PZ -0.04257 0.06179 0.00240 0.00767 -0.00227

158 3S -0.02304 0.00177 -0.00672 0.00282 -0.00408

159 3PX 0.03959 -0.02648 0.00366 -0.00286 0.00393

160 3PY -0.01707 0.01646 -0.00120 0.00344 0.00036

161 3PZ -0.06363 0.09327 0.00358 0.01160 -0.00346

162 4S -0.00685 -0.01159 -0.00360 -0.00059 -0.00365

163 4PX 0.05266 -0.02665 0.00163 -0.00380 0.00389

164 4PY -0.02615 0.02166 -0.00050 0.00311 -0.00070

165 4PZ -0.08296 0.09742 0.00286 0.01188 -0.00390

166 5D 0 0.00120 -0.00374 0.00010 -0.00029 0.00009

167 5D+1 0.00185 -0.00998 -0.00030 -0.00009 -0.00009

168 5D-1 -0.00313 0.00291 0.00021 0.00062 -0.00014

169 5D+2 -0.00975 0.00580 -0.00010 0.00077 -0.00017

170 5D-2 -0.00990 0.00186 0.00024 0.00077 -0.00020

171 10 H 1S 0.00254 -0.00077 -0.00001 -0.00023 -0.00001

172 2S 0.00199 -0.00146 -0.00003 -0.00027 -0.00007

173 3S 0.00549 -0.00471 -0.00018 -0.00060 0.00003

174 4PX 0.00047 -0.00056 -0.00001 -0.00003 0.00001

175 4PY -0.00040 0.00037 0.00000 -0.00001 -0.00001

176 4PZ -0.00139 0.00215 -0.00001 0.00005 -0.00003

177 11 H 1S 0.01556 0.00667 0.00059 -0.00033 0.00007

178 2S 0.02552 0.01191 0.00094 -0.00043 0.00033

179 3S 0.02985 0.00458 0.00059 -0.00110 0.00096

180 4PX 0.00128 0.00009 0.00010 -0.00011 0.00008

181 4PY 0.00087 -0.00011 0.00004 -0.00009 0.00015

182 4PZ -0.00007 0.00008 0.00009 -0.00003 0.00011

183 12 H 1S -0.01596 -0.01169 0.00033 -0.00073 -0.00010

184 2S -0.02314 -0.02063 0.00012 -0.00096 -0.00045

185 3S -0.01343 -0.01165 -0.00017 0.00021 -0.00092

186 4PX -0.00343 0.00228 0.00014 0.00007 0.00009

187 4PY -0.00429 -0.00245 -0.00015 0.00015 -0.00030

188 4PZ 0.00011 0.00199 0.00002 0.00008 -0.00001

189 13 H 1S -0.03134 -0.02099 0.00087 0.00491 0.00237

190 2S -0.04822 -0.02702 0.00132 0.00712 0.00302

191 3S -0.02861 -0.00352 0.00078 0.00314 0.00034

192 4PX -0.00127 -0.00653 -0.00020 0.00011 0.00017

193 4PY 0.00657 -0.00467 0.00005 -0.00027 0.00052

194 4PZ -0.00474 0.00400 0.00018 0.00075 0.00014

195 14 H 1S 0.01619 -0.00731 -0.00003 -0.00143 -0.00054

196 2S 0.02975 -0.01324 -0.00003 -0.00248 -0.00050

197 3S 0.03468 -0.00898 0.00055 -0.00256 0.00100

198 4PX 0.00266 -0.00188 0.00014 -0.00018 0.00025

199 4PY -0.00205 0.00136 -0.00003 0.00023 -0.00021

200 4PZ -0.00346 0.00477 0.00017 0.00062 -0.00016

151 152 153 154 155

151 5D+2 0.00104

152 5D-2 0.00003 0.00102

153 9 C 1S -0.00301 0.00314 0.65842

154 2S -0.00409 0.00407 0.55990 0.48883

155 2PX -0.00250 0.00514 0.00049 0.00058 0.08542

156 2PY 0.00608 0.00450 0.00003 0.00009 -0.00069

157 2PZ -0.00220 0.00241 -0.00014 -0.00030 0.00244

158 3S 0.00943 -0.00839 -0.08249 -0.14348 -0.00132

159 3PX -0.00341 0.00733 -0.00019 -0.00008 0.12147

160 3PY 0.00859 0.00631 -0.00061 -0.00095 -0.00068

161 3PZ -0.00322 0.00358 -0.00030 -0.00050 0.00234

162 4S 0.00431 -0.00777 -0.07149 -0.10139 -0.00870

163 4PX -0.00293 0.00253 0.00533 0.00728 0.05651

164 4PY 0.00540 0.00356 -0.00823 -0.00800 -0.00641

165 4PZ -0.00178 0.00386 0.00204 0.00203 -0.00712

166 5D 0 -0.00015 0.00010 0.00083 0.00262 -0.00007

167 5D+1 0.00039 -0.00028 -0.00066 -0.00140 -0.00199

168 5D-1 0.00001 0.00033 -0.00003 0.00042 -0.00015

169 5D+2 0.00072 0.00035 -0.00067 -0.00085 -0.00484

170 5D-2 0.00047 0.00103 -0.00035 -0.00058 0.00698

171 10 H 1S -0.00017 -0.00047 0.00099 0.00160 -0.00324

172 2S -0.00023 -0.00071 0.00191 0.00267 -0.00589

173 3S -0.00036 -0.00100 0.00219 0.00313 -0.00792

174 4PX -0.00004 -0.00005 0.00007 0.00013 -0.00029

175 4PY -0.00002 -0.00002 0.00010 0.00018 -0.00041

176 4PZ -0.00001 0.00005 0.00001 0.00002 0.00002

177 11 H 1S 0.00030 -0.00058 0.00007 0.00016 0.00112

178 2S 0.00036 -0.00081 -0.00036 -0.00010 0.00135

179 3S -0.00060 -0.00077 0.00119 0.00218 0.00276

180 4PX 0.00008 -0.00002 0.00007 0.00013 0.00026

181 4PY -0.00007 0.00004 0.00028 0.00040 0.00035

182 4PZ 0.00010 0.00004 -0.00008 -0.00008 0.00034

183 12 H 1S 0.00102 -0.00117 -0.00107 -0.00175 -0.00278

184 2S 0.00188 -0.00180 -0.00232 -0.00377 -0.00667

185 3S 0.00125 -0.00067 -0.00110 -0.00212 -0.00523

186 4PX 0.00015 0.00007 -0.00011 -0.00011 -0.00020

187 4PY 0.00018 0.00004 -0.00008 -0.00021 -0.00066

188 4PZ 0.00004 0.00017 0.00013 0.00018 0.00027

189 13 H 1S 0.00121 0.00483 0.00461 0.00747 0.01424

190 2S 0.00174 0.00711 0.00550 0.00962 0.02264

191 3S 0.00049 0.00325 0.00383 0.00554 0.01414

192 4PX 0.00037 0.00006 -0.00113 -0.00122 -0.00118

193 4PY -0.00027 0.00018 0.00061 0.00108 0.00300

194 4PZ -0.00007 0.00044 0.00014 0.00043 0.00017

195 14 H 1S -0.00412 -0.00461 -0.01976 -0.03103 0.01447

196 2S -0.00610 -0.00645 -0.03166 -0.04540 0.02146

197 3S -0.00388 -0.00248 -0.00184 -0.00330 0.01261

198 4PX -0.00005 0.00040 0.00112 0.00139 0.00480

199 4PY -0.00021 -0.00039 -0.00643 -0.00813 0.00213

200 4PZ -0.00003 0.00031 0.00116 0.00144 -0.00057

156 157 158 159 160

156 2PY 0.08725

157 2PZ 0.00057 0.07112

158 3S 0.00224 0.00145 0.43389

159 3PX -0.00056 0.00282 -0.00091 0.17292

160 3PY 0.12312 0.00199 0.00631 -0.00030 0.17393

161 3PZ 0.00215 0.10740 0.00212 0.00234 0.00481

162 4S -0.02097 -0.00385 0.24335 -0.01264 -0.02794

163 4PX -0.01094 -0.01719 -0.01798 0.07984 -0.01592

164 4PY 0.07297 0.00922 0.00945 -0.00885 0.10326

165 4PZ 0.01042 0.09921 -0.00093 -0.01054 0.01619

166 5D 0 -0.00065 -0.00194 -0.01105 -0.00015 -0.00103

167 5D+1 0.00085 -0.00199 0.00510 -0.00290 0.00132

168 5D-1 0.00244 0.00521 -0.00245 -0.00027 0.00357

169 5D+2 0.00643 0.00150 0.00202 -0.00671 0.00924

170 5D-2 0.00542 0.00441 0.00197 0.01017 0.00781

171 10 H 1S -0.00160 -0.00098 -0.00425 -0.00443 -0.00125

172 2S -0.00153 -0.00114 -0.00578 -0.00823 -0.00060

173 3S -0.00390 -0.00387 -0.00756 -0.01127 -0.00451

174 4PX -0.00033 -0.00015 -0.00049 -0.00042 -0.00050

175 4PY -0.00025 -0.00011 -0.00063 -0.00057 -0.00031

176 4PZ 0.00008 0.00010 -0.00004 0.00008 0.00007

177 11 H 1S 0.00059 -0.00036 -0.00131 0.00129 0.00056

178 2S 0.00129 -0.00010 -0.00242 0.00142 0.00135

179 3S -0.00469 -0.00403 -0.00826 0.00352 -0.00731

180 4PX -0.00020 -0.00080 -0.00051 0.00041 -0.00030

181 4PY -0.00060 -0.00073 -0.00101 0.00047 -0.00090

182 4PZ -0.00004 -0.00096 -0.00002 0.00043 -0.00010

183 12 H 1S 0.00167 -0.00266 0.00582 -0.00396 0.00282

184 2S 0.00480 -0.00451 0.01202 -0.00939 0.00748

185 3S 0.00448 0.00207 0.00789 -0.00732 0.00674

186 4PX 0.00032 -0.00035 0.00011 -0.00026 0.00042

187 4PY 0.00041 0.00029 0.00106 -0.00088 0.00062

188 4PZ 0.00000 -0.00020 -0.00049 0.00042 -0.00009

189 13 H 1S -0.00220 0.00434 -0.02129 0.02046 -0.00333

190 2S -0.00004 0.00972 -0.02932 0.03248 -0.00034

191 3S 0.00286 0.01186 -0.01323 0.02035 0.00402

192 4PX -0.00023 -0.00314 0.00170 -0.00165 -0.00034

193 4PY -0.00137 -0.00247 -0.00347 0.00419 -0.00199

194 4PZ -0.00084 0.00329 -0.00192 0.00024 -0.00120

195 14 H 1S -0.07203 0.00602 0.08217 0.02019 -0.10102

196 2S -0.10066 0.00666 0.10718 0.02994 -0.14120

197 3S -0.04559 -0.00449 0.00809 0.01741 -0.06461

198 4PX 0.00166 -0.00082 -0.00264 0.00682 0.00232

199 4PY -0.00633 0.00294 0.01604 0.00305 -0.00872

200 4PZ 0.00248 0.00514 -0.00266 -0.00085 0.00356

161 162 163 164 165

161 3PZ 0.16229

162 4S -0.00597 0.18357

163 4PX -0.02681 -0.00508 0.05498

164 4PY 0.01491 -0.00400 -0.01669 0.06764

165 4PZ 0.14958 -0.01040 -0.03519 0.02330 0.14752

166 5D 0 -0.00292 -0.00514 0.00065 -0.00075 -0.00288

167 5D+1 -0.00285 0.00344 -0.00120 0.00015 -0.00393

168 5D-1 0.00796 -0.00204 -0.00174 0.00268 0.00699

169 5D+2 0.00231 -0.00185 -0.00586 0.00713 0.00479

170 5D-2 0.00653 -0.00269 0.00072 0.00477 0.00754

171 10 H 1S -0.00150 -0.01733 -0.01339 -0.00198 -0.00345

172 2S -0.00176 -0.01823 -0.01837 0.00022 -0.00409

173 3S -0.00579 -0.00707 -0.00849 -0.00177 -0.00730

174 4PX -0.00022 0.00175 0.00128 -0.00011 -0.00017

175 4PY -0.00017 0.00046 0.00064 -0.00004 0.00006

176 4PZ 0.00016 -0.00054 -0.00022 0.00007 0.00052

177 11 H 1S -0.00052 0.01154 0.00634 0.00240 -0.00188

178 2S -0.00004 0.01503 0.01002 0.00301 -0.00280

179 3S -0.00592 -0.00382 0.01005 -0.00897 -0.01017

180 4PX -0.00118 0.00011 0.00061 -0.00020 -0.00148

181 4PY -0.00108 -0.00168 0.00061 -0.00114 -0.00140

182 4PZ -0.00144 0.00090 0.00083 0.00021 -0.00168

183 12 H 1S -0.00402 0.00759 -0.00530 0.00221 -0.00205

184 2S -0.00672 0.01214 -0.01015 0.00467 -0.00344

185 3S 0.00324 0.00701 -0.00856 0.00459 0.00479

186 4PX -0.00054 0.00008 -0.00020 0.00065 -0.00018

187 4PY 0.00042 0.00064 -0.00185 0.00069 0.00109

188 4PZ -0.00031 -0.00072 0.00036 0.00011 -0.00027

189 13 H 1S 0.00568 -0.03097 0.00952 -0.00495 0.01144

190 2S 0.01348 -0.04468 0.01259 -0.00429 0.02112

191 3S 0.01737 -0.01906 0.00305 0.00192 0.02056

192 4PX -0.00476 -0.00007 -0.00010 -0.00081 -0.00395

193 4PY -0.00376 -0.00272 0.00423 -0.00241 -0.00454

194 4PZ 0.00486 -0.00312 -0.00036 -0.00046 0.00551

195 14 H 1S 0.00793 0.06363 0.01590 -0.06045 -0.00367

196 2S 0.00849 0.08361 0.02396 -0.08565 -0.00872

197 3S -0.00734 0.01493 0.02026 -0.04269 -0.01685

198 4PX -0.00128 -0.00237 0.00342 0.00084 -0.00167

199 4PY 0.00429 0.00989 0.00041 -0.00464 0.00320

200 4PZ 0.00781 -0.00243 -0.00190 0.00265 0.00752

166 167 168 169 170

166 5D 0 0.00044

167 5D+1 0.00016 0.00136

168 5D-1 -0.00003 0.00016 0.00060

169 5D+2 -0.00015 -0.00008 0.00035 0.00228

170 5D-2 -0.00019 -0.00021 0.00042 0.00052 0.00215

171 10 H 1S 0.00010 0.00028 0.00026 0.00137 -0.00003

172 2S 0.00023 0.00060 0.00050 0.00269 -0.00033

173 3S 0.00029 0.00078 0.00010 0.00131 -0.00124

174 4PX 0.00000 0.00005 -0.00002 -0.00016 -0.00010

175 4PY -0.00003 -0.00003 -0.00002 0.00008 -0.00001

176 4PZ -0.00004 -0.00024 -0.00006 0.00013 0.00012

177 11 H 1S -0.00017 -0.00086 -0.00035 -0.00096 -0.00094

178 2S -0.00037 -0.00145 -0.00047 -0.00183 -0.00161

179 3S -0.00020 -0.00118 -0.00075 -0.00345 -0.00197

180 4PX 0.00001 -0.00006 -0.00002 -0.00001 -0.00010

181 4PY 0.00001 -0.00008 -0.00003 -0.00018 -0.00010

182 4PZ 0.00002 -0.00009 -0.00001 -0.00001 -0.00002

183 12 H 1S 0.00044 0.00120 0.00007 0.00071 0.00136

184 2S 0.00060 0.00236 0.00016 0.00109 0.00197

185 3S 0.00024 0.00183 0.00053 0.00068 0.00120

186 4PX -0.00003 -0.00030 0.00000 0.00025 0.00022

187 4PY 0.00007 0.00032 0.00007 0.00017 0.00037

188 4PZ -0.00005 -0.00026 -0.00003 0.00007 -0.00004

189 13 H 1S 0.00079 0.00185 0.00069 0.00237 0.00518

190 2S 0.00102 0.00247 0.00131 0.00320 0.00813

191 3S 0.00030 0.00030 0.00102 0.00120 0.00480

192 4PX 0.00013 0.00064 -0.00015 0.00018 0.00007

193 4PY 0.00014 0.00023 -0.00017 -0.00050 -0.00027

194 4PZ -0.00010 -0.00029 0.00016 0.00040 0.00044

195 14 H 1S -0.00187 -0.00016 -0.00217 -0.00691 -0.00339

196 2S -0.00239 -0.00010 -0.00310 -0.01024 -0.00514

197 3S -0.00014 -0.00085 -0.00190 -0.00687 -0.00380

198 4PX 0.00008 -0.00007 -0.00001 -0.00024 0.00039

199 4PY -0.00044 0.00000 -0.00005 -0.00023 0.00011

200 4PZ -0.00008 -0.00012 0.00046 0.00028 0.00040

171 172 173 174 175

171 10 H 1S 0.08184

172 2S 0.11218 0.15610

173 3S 0.04601 0.06567 0.03377

174 4PX -0.00814 -0.01093 -0.00337 0.00108

175 4PY -0.00348 -0.00447 -0.00082 0.00048 0.00048

176 4PZ -0.00058 -0.00077 -0.00042 0.00001 0.00004

177 11 H 1S -0.00113 -0.00259 -0.00146 0.00021 0.00009

178 2S -0.00256 -0.00547 -0.00277 0.00044 0.00014

179 3S -0.00527 -0.01145 -0.00563 0.00065 -0.00022

180 4PX -0.00015 -0.00037 -0.00030 -0.00001 0.00000

181 4PY -0.00003 -0.00010 0.00004 0.00001 -0.00001

182 4PZ 0.00015 0.00019 0.00024 0.00002 -0.00001

183 12 H 1S -0.00096 -0.00148 -0.00190 -0.00008 0.00004

184 2S -0.00206 -0.00317 -0.00335 -0.00011 0.00007

185 3S -0.00156 -0.00205 -0.00201 -0.00006 0.00006

186 4PX -0.00020 -0.00040 -0.00038 -0.00001 0.00000

187 4PY 0.00012 0.00030 0.00018 -0.00001 -0.00001

188 4PZ -0.00013 -0.00022 -0.00013 -0.00001 0.00000

189 13 H 1S -0.00149 -0.00249 -0.00357 -0.00010 -0.00009

190 2S -0.00256 -0.00459 -0.00631 -0.00020 -0.00023

191 3S -0.00282 -0.00458 -0.00499 -0.00011 -0.00012

192 4PX 0.00011 0.00017 0.00020 0.00001 -0.00001

193 4PY -0.00005 -0.00017 0.00006 0.00004 -0.00002

194 4PZ -0.00004 -0.00012 -0.00040 -0.00004 0.00001

195 14 H 1S -0.00165 -0.00368 -0.00111 0.00029 -0.00003

196 2S -0.00062 -0.00322 -0.00064 0.00028 -0.00020

197 3S -0.00280 -0.00625 -0.00149 0.00055 0.00001

198 4PX 0.00010 0.00005 -0.00022 -0.00004 -0.00003

199 4PY 0.00033 0.00045 0.00004 -0.00005 -0.00001

200 4PZ -0.00010 -0.00011 -0.00032 -0.00001 -0.00001

176 177 178 179 180

176 4PZ 0.00033

177 11 H 1S -0.00011 0.07964

178 2S -0.00032 0.10929 0.15094

179 3S -0.00029 0.04137 0.05966 0.03576

180 4PX -0.00002 0.00192 0.00277 0.00128 0.00036

181 4PY 0.00000 -0.00870 -0.01152 -0.00288 -0.00011

182 4PZ -0.00003 0.00214 0.00312 0.00146 0.00009

183 12 H 1S -0.00011 -0.00314 -0.00613 -0.00575 0.00083

184 2S -0.00020 -0.00623 -0.01125 -0.00940 0.00119

185 3S -0.00027 -0.00411 -0.00698 -0.00602 0.00049

186 4PX 0.00004 -0.00011 -0.00006 -0.00008 0.00013

187 4PY 0.00000 -0.00110 -0.00189 -0.00186 -0.00024

188 4PZ 0.00001 -0.00043 -0.00032 0.00022 0.00004

189 13 H 1S 0.00006 -0.00297 -0.00523 -0.00562 -0.00018

190 2S 0.00012 -0.00637 -0.01042 -0.00939 -0.00040

191 3S 0.00029 -0.00522 -0.00837 -0.00678 -0.00050

192 4PX -0.00006 -0.00056 -0.00096 -0.00074 -0.00002

193 4PY -0.00009 0.00051 0.00085 0.00124 0.00005

194 4PZ 0.00010 0.00007 0.00011 -0.00020 -0.00002

195 14 H 1S -0.00020 0.00131 0.00209 0.00644 0.00010

196 2S -0.00040 0.00175 0.00299 0.01023 0.00016

197 3S -0.00030 0.00595 0.01000 0.01413 0.00045

198 4PX -0.00002 0.00014 0.00022 0.00030 0.00003

199 4PY 0.00001 -0.00041 -0.00071 -0.00061 -0.00003

200 4PZ 0.00002 -0.00005 -0.00002 -0.00039 -0.00007

181 182 183 184 185

181 4PY 0.00127

182 4PZ -0.00010 0.00039

183 12 H 1S -0.00010 -0.00062 0.07776

184 2S -0.00010 -0.00121 0.10714 0.14871

185 3S -0.00012 -0.00107 0.03575 0.05121 0.02099

186 4PX 0.00011 0.00029 0.00501 0.00648 0.00131

187 4PY -0.00012 -0.00005 -0.00181 -0.00223 -0.00022

188 4PZ 0.00017 0.00014 -0.00791 -0.01095 -0.00364

189 13 H 1S -0.00011 0.00027 0.00211 0.00312 0.00251

190 2S -0.00006 0.00035 0.00338 0.00498 0.00386

191 3S -0.00010 -0.00023 0.00268 0.00387 0.00292

192 4PX -0.00002 0.00002 0.00080 0.00142 0.00080

193 4PY 0.00005 0.00001 -0.00068 -0.00102 -0.00067

194 4PZ -0.00002 0.00000 -0.00013 -0.00039 -0.00023

195 14 H 1S 0.00040 -0.00001 -0.00257 -0.00533 -0.00387

196 2S 0.00062 -0.00011 -0.00458 -0.00876 -0.00601

197 3S 0.00061 0.00033 -0.00570 -0.00974 -0.00661

198 4PX 0.00003 0.00004 -0.00027 -0.00050 -0.00035

199 4PY -0.00003 -0.00004 0.00006 -0.00007 -0.00001

200 4PZ -0.00006 -0.00008 -0.00016 -0.00020 0.00029

186 187 188 189 190

186 4PX 0.00080

187 4PY -0.00022 0.00050

188 4PZ -0.00036 0.00014 0.00097

189 13 H 1S 0.00045 0.00097 -0.00016 0.07761

190 2S 0.00078 0.00161 -0.00023 0.10680 0.14796

191 3S 0.00029 0.00115 -0.00022 0.03609 0.05147

192 4PX -0.00002 0.00018 -0.00010 0.00638 0.00858

193 4PY -0.00017 -0.00019 -0.00004 0.00339 0.00437

194 4PZ 0.00013 -0.00004 0.00003 0.00616 0.00851

195 14 H 1S -0.00057 -0.00060 -0.00005 -0.00321 -0.00620

196 2S -0.00105 -0.00097 -0.00010 -0.00620 -0.01104

197 3S -0.00052 -0.00141 0.00028 -0.00549 -0.00924

198 4PX -0.00002 -0.00006 0.00003 0.00095 0.00146

199 4PY -0.00001 0.00004 -0.00002 0.00038 0.00053

200 4PZ -0.00003 0.00004 -0.00002 0.00036 0.00084

191 192 193 194 195

191 3S 0.02136

192 4PX 0.00229 0.00087

193 4PY 0.00073 0.00036 0.00067

194 4PZ 0.00305 0.00030 0.00013 0.00077

195 14 H 1S -0.00410 -0.00021 0.00111 0.00033 0.08008

196 2S -0.00689 -0.00038 0.00190 0.00018 0.11108

197 3S -0.00625 -0.00058 0.00202 -0.00029 0.04449

198 4PX 0.00075 -0.00002 0.00023 -0.00004 -0.00111

199 4PY 0.00038 -0.00003 -0.00008 0.00018 0.00877

200 4PZ 0.00097 -0.00022 -0.00021 0.00023 -0.00223

196 197 198 199 200

196 2S 0.15482

197 3S 0.06357 0.03437

198 4PX -0.00138 -0.00001 0.00034

199 4PY 0.01192 0.00334 -0.00015 0.00129

200 4PZ -0.00320 -0.00172 -0.00003 -0.00010 0.00047

Full Mulliken population analysis:

1 2 3 4 5

1 1 S 1S 0.36887

2 2S 0.45156 1.08247

3 3S -0.02826 -0.20623 0.63370

4 4S -0.01946 -0.15258 0.66198 0.99054

5 5S 0.00133 0.01191 -0.09355 -0.29712 0.97706

6 6S 0.00057 0.00519 -0.04061 -0.13010 0.55321

7 7PX 0.00000 0.00000 0.00000 0.00000 0.00000

8 7PY 0.00000 0.00000 0.00000 0.00000 0.00000

9 7PZ 0.00000 0.00000 0.00000 0.00000 0.00000

10 8PX 0.00000 0.00000 0.00000 0.00000 0.00000

11 8PY 0.00000 0.00000 0.00000 0.00000 0.00000

12 8PZ 0.00000 0.00000 0.00000 0.00000 0.00000

13 9PX 0.00000 0.00000 0.00000 0.00000 0.00000

14 9PY 0.00000 0.00000 0.00000 0.00000 0.00000

15 9PZ 0.00000 0.00000 0.00000 0.00000 0.00000

16 10PX 0.00000 0.00000 0.00000 0.00000 0.00000

17 10PY 0.00000 0.00000 0.00000 0.00000 0.00000

18 10PZ 0.00000 0.00000 0.00000 0.00000 0.00000

19 11PX 0.00000 0.00000 0.00000 0.00000 0.00000

20 11PY 0.00000 0.00000 0.00000 0.00000 0.00000

21 11PZ 0.00000 0.00000 0.00000 0.00000 0.00000

22 12D 0 0.00000 0.00000 0.00000 0.00000 0.00000

23 12D+1 0.00000 0.00000 0.00000 0.00000 0.00000

24 12D-1 0.00000 0.00000 0.00000 0.00000 0.00000

25 12D+2 0.00000 0.00000 0.00000 0.00000 0.00000

26 12D-2 0.00000 0.00000 0.00000 0.00000 0.00000

27 2 C 1S 0.00000 0.00000 0.00000 0.00000 -0.00010

28 2S 0.00000 0.00000 0.00000 0.00000 -0.00016

29 2PX 0.00000 0.00000 0.00000 0.00000 0.00003

30 2PY 0.00000 0.00000 0.00000 -0.00003 0.00312

31 2PZ 0.00000 0.00000 0.00000 0.00000 0.00007

32 3S 0.00000 0.00001 -0.00007 -0.00034 0.00410

33 3PX 0.00000 0.00000 -0.00001 -0.00003 0.00023

34 3PY 0.00001 0.00009 -0.00081 -0.00327 0.02341

35 3PZ 0.00000 0.00000 -0.00002 -0.00008 0.00054

36 4S -0.00002 -0.00019 0.00153 0.00493 -0.02361

37 4PX 0.00000 0.00000 -0.00002 -0.00007 0.00044

38 4PY -0.00001 -0.00008 0.00069 0.00234 -0.01075

39 4PZ 0.00000 0.00000 0.00004 0.00013 -0.00066

40 5D 0 0.00000 0.00000 -0.00003 -0.00012 0.00056

41 5D+1 0.00000 0.00000 0.00000 0.00000 0.00000

42 5D-1 0.00000 0.00000 -0.00002 -0.00008 0.00042

43 5D+2 0.00000 0.00002 -0.00022 -0.00088 0.00434

44 5D-2 0.00000 0.00000 0.00000 0.00001 -0.00006

45 3 N 1S 0.00000 0.00000 0.00000 0.00000 0.00000

46 2S 0.00000 0.00000 0.00000 0.00000 0.00000

47 2PX 0.00000 0.00000 0.00000 0.00000 0.00000

48 2PY 0.00000 0.00000 0.00000 0.00000 -0.00001

49 2PZ 0.00000 0.00000 0.00000 0.00000 0.00000

50 3S 0.00000 0.00000 0.00000 0.00000 0.00002

51 3PX 0.00000 0.00000 0.00000 0.00000 0.00001

52 3PY 0.00000 0.00000 0.00000 0.00000 -0.00017

53 3PZ 0.00000 0.00000 0.00000 0.00000 -0.00001

54 4S 0.00000 0.00000 -0.00004 -0.00015 0.00148

55 4PX 0.00000 0.00000 -0.00001 -0.00004 0.00029

56 4PY 0.00000 -0.00001 0.00005 0.00021 -0.00297

57 4PZ 0.00000 0.00000 0.00000 0.00001 -0.00011

58 5D 0 0.00000 0.00000 0.00000 0.00000 0.00000

59 5D+1 0.00000 0.00000 0.00000 0.00000 0.00000

60 5D-1 0.00000 0.00000 0.00000 0.00000 0.00000

61 5D+2 0.00000 0.00000 0.00000 0.00000 0.00000

62 5D-2 0.00000 0.00000 0.00000 0.00000 0.00000

63 4 C 1S 0.00000 0.00000 0.00000 0.00000 0.00000

64 2S 0.00000 0.00000 0.00000 0.00000 0.00000

65 2PX 0.00000 0.00000 0.00000 0.00000 0.00000

66 2PY 0.00000 0.00000 0.00000 0.00000 0.00000

67 2PZ 0.00000 0.00000 0.00000 0.00000 0.00000

68 3S 0.00000 0.00000 0.00000 0.00000 -0.00007

69 3PX 0.00000 0.00000 0.00000 0.00000 -0.00021

70 3PY 0.00000 0.00000 0.00000 0.00000 -0.00002

71 3PZ 0.00000 0.00000 0.00000 0.00000 -0.00001

72 4S 0.00000 0.00000 0.00004 0.00012 -0.00083

73 4PX 0.00000 -0.00002 0.00018 0.00055 -0.00226

74 4PY 0.00000 -0.00001 0.00006 0.00020 -0.00090

75 4PZ 0.00000 0.00000 0.00001 0.00003 -0.00023

76 5D 0 0.00000 0.00000 0.00000 0.00000 0.00000

77 5D+1 0.00000 0.00000 0.00000 0.00000 0.00000

78 5D-1 0.00000 0.00000 0.00000 0.00000 0.00000

79 5D+2 0.00000 0.00000 0.00000 0.00000 0.00000

80 5D-2 0.00000 0.00000 0.00000 0.00000 0.00001

81 5 C 1S 0.00000 0.00000 0.00000 0.00000 -0.00001

82 2S 0.00000 0.00000 0.00000 0.00000 -0.00002

83 2PX 0.00000 0.00000 0.00000 0.00000 0.00065

84 2PY 0.00000 0.00000 0.00000 0.00000 0.00003

85 2PZ 0.00000 0.00000 0.00000 0.00000 0.00003

86 3S 0.00000 0.00000 0.00000 -0.00003 0.00039

87 3PX 0.00000 0.00001 -0.00013 -0.00067 0.00801

88 3PY 0.00000 0.00000 0.00000 -0.00002 0.00032

89 3PZ 0.00000 0.00000 0.00000 -0.00002 0.00030

90 4S -0.00001 -0.00005 0.00047 0.00133 -0.00715

91 4PX 0.00000 -0.00002 0.00027 0.00102 -0.00994

92 4PY 0.00000 -0.00001 0.00008 0.00015 0.00027

93 4PZ 0.00000 0.00000 0.00004 0.00008 -0.00021

94 5D 0 0.00000 0.00000 -0.00001 -0.00003 0.00027

95 5D+1 0.00000 0.00000 0.00000 -0.00002 0.00021

96 5D-1 0.00000 0.00000 0.00000 0.00000 0.00000

97 5D+2 0.00000 0.00000 -0.00003 -0.00016 0.00162

98 5D-2 0.00000 0.00000 -0.00001 -0.00003 0.00033

99 6 C 1S 0.00000 0.00000 0.00000 0.00000 0.00000

100 2S 0.00000 0.00000 0.00000 0.00000 0.00000

101 2PX 0.00000 0.00000 0.00000 0.00000 0.00000

102 2PY 0.00000 0.00000 0.00000 0.00000 0.00000

103 2PZ 0.00000 0.00000 0.00000 0.00000 0.00000

104 3S 0.00000 0.00000 0.00000 0.00000 0.00000

105 3PX 0.00000 0.00000 0.00000 0.00000 0.00000

106 3PY 0.00000 0.00000 0.00000 0.00000 0.00000

107 3PZ 0.00000 0.00000 0.00000 0.00000 0.00000

108 4S 0.00000 0.00000 0.00000 0.00000 0.00000

109 4PX 0.00000 0.00000 0.00000 0.00000 0.00001

110 4PY 0.00000 0.00000 0.00000 0.00000 0.00005

111 4PZ 0.00000 0.00000 0.00000 0.00000 0.00000

112 5D 0 0.00000 0.00000 0.00000 0.00000 0.00000

113 5D+1 0.00000 0.00000 0.00000 0.00000 0.00000

114 5D-1 0.00000 0.00000 0.00000 0.00000 0.00000

115 5D+2 0.00000 0.00000 0.00000 0.00000 0.00000

116 5D-2 0.00000 0.00000 0.00000 0.00000 0.00000

117 7 C 1S 0.00000 0.00000 0.00000 0.00000 0.00000

118 2S 0.00000 0.00000 0.00000 0.00000 0.00000

119 2PX 0.00000 0.00000 0.00000 0.00000 0.00000

120 2PY 0.00000 0.00000 0.00000 0.00000 0.00000

121 2PZ 0.00000 0.00000 0.00000 0.00000 0.00000

122 3S 0.00000 0.00000 0.00000 0.00000 0.00000

123 3PX 0.00000 0.00000 0.00000 0.00000 0.00000

124 3PY 0.00000 0.00000 0.00000 0.00000 0.00000

125 3PZ 0.00000 0.00000 0.00000 0.00000 0.00000

126 4S 0.00000 0.00000 0.00000 0.00000 0.00000

127 4PX 0.00000 0.00000 0.00000 0.00000 0.00000

128 4PY 0.00000 0.00000 0.00000 0.00000 0.00000

129 4PZ 0.00000 0.00000 0.00000 0.00000 0.00000

130 5D 0 0.00000 0.00000 0.00000 0.00000 0.00000

131 5D+1 0.00000 0.00000 0.00000 0.00000 0.00000

132 5D-1 0.00000 0.00000 0.00000 0.00000 0.00000

133 5D+2 0.00000 0.00000 0.00000 0.00000 0.00000

134 5D-2 0.00000 0.00000 0.00000 0.00000 0.00000

135 8 C 1S 0.00000 0.00000 0.00000 0.00000 0.00000

136 2S 0.00000 0.00000 0.00000 0.00000 0.00000

137 2PX 0.00000 0.00000 0.00000 0.00000 0.00000

138 2PY 0.00000 0.00000 0.00000 0.00000 0.00000

139 2PZ 0.00000 0.00000 0.00000 0.00000 0.00000

140 3S 0.00000 0.00000 0.00000 0.00000 0.00000

141 3PX 0.00000 0.00000 0.00000 0.00000 0.00000

142 3PY 0.00000 0.00000 0.00000 0.00000 0.00000

143 3PZ 0.00000 0.00000 0.00000 0.00000 0.00000

144 4S 0.00000 0.00000 0.00000 0.00000 0.00000

145 4PX 0.00000 0.00000 0.00000 0.00000 0.00002

146 4PY 0.00000 0.00000 0.00000 0.00000 0.00000

147 4PZ 0.00000 0.00000 0.00000 0.00000 0.00000

148 5D 0 0.00000 0.00000 0.00000 0.00000 0.00000

149 5D+1 0.00000 0.00000 0.00000 0.00000 0.00000

150 5D-1 0.00000 0.00000 0.00000 0.00000 0.00000

151 5D+2 0.00000 0.00000 0.00000 0.00000 0.00000

152 5D-2 0.00000 0.00000 0.00000 0.00000 0.00000

153 9 C 1S 0.00000 0.00000 0.00000 0.00000 0.00000

154 2S 0.00000 0.00000 0.00000 0.00000 0.00000

155 2PX 0.00000 0.00000 0.00000 0.00000 0.00000

156 2PY 0.00000 0.00000 0.00000 0.00000 0.00000

157 2PZ 0.00000 0.00000 0.00000 0.00000 0.00000

158 3S 0.00000 0.00000 0.00000 0.00000 -0.00002

159 3PX 0.00000 0.00000 0.00000 0.00000 -0.00007

160 3PY 0.00000 0.00000 0.00000 0.00000 0.00000

161 3PZ 0.00000 0.00000 0.00000 0.00000 0.00000

162 4S 0.00000 0.00000 0.00000 0.00001 -0.00068

163 4PX 0.00000 -0.00001 0.00006 0.00022 -0.00196

164 4PY 0.00000 0.00000 -0.00001 -0.00002 0.00004

165 4PZ 0.00000 0.00000 0.00000 0.00001 -0.00005

166 5D 0 0.00000 0.00000 0.00000 0.00000 0.00000

167 5D+1 0.00000 0.00000 0.00000 0.00000 0.00000

168 5D-1 0.00000 0.00000 0.00000 0.00000 0.00000

169 5D+2 0.00000 0.00000 0.00000 0.00000 0.00001

170 5D-2 0.00000 0.00000 0.00000 0.00000 0.00000

171 10 H 1S 0.00000 0.00000 0.00000 0.00000 -0.00008

172 2S 0.00000 0.00000 0.00001 0.00007 -0.00164

173 3S 0.00000 -0.00003 0.00022 0.00073 -0.00480

174 4PX 0.00000 0.00000 0.00000 0.00000 0.00000

175 4PY 0.00000 0.00000 0.00000 0.00000 0.00003

176 4PZ 0.00000 0.00000 0.00000 0.00000 0.00000

177 11 H 1S 0.00000 0.00000 0.00000 0.00000 0.00000

178 2S 0.00000 0.00000 0.00000 0.00000 0.00000

179 3S 0.00000 0.00000 0.00000 0.00000 0.00000

180 4PX 0.00000 0.00000 0.00000 0.00000 0.00000

181 4PY 0.00000 0.00000 0.00000 0.00000 0.00000

182 4PZ 0.00000 0.00000 0.00000 0.00000 0.00000

183 12 H 1S 0.00000 0.00000 0.00000 0.00000 0.00000

184 2S 0.00000 0.00000 0.00000 0.00000 0.00000

185 3S 0.00000 0.00000 0.00000 0.00000 0.00000

186 4PX 0.00000 0.00000 0.00000 0.00000 0.00000

187 4PY 0.00000 0.00000 0.00000 0.00000 0.00000

188 4PZ 0.00000 0.00000 0.00000 0.00000 0.00000

189 13 H 1S 0.00000 0.00000 0.00000 0.00000 0.00000

190 2S 0.00000 0.00000 0.00000 0.00000 0.00000

191 3S 0.00000 0.00000 0.00000 0.00000 0.00000

192 4PX 0.00000 0.00000 0.00000 0.00000 0.00000

193 4PY 0.00000 0.00000 0.00000 0.00000 0.00000

194 4PZ 0.00000 0.00000 0.00000 0.00000 0.00000

195 14 H 1S 0.00000 0.00000 0.00000 0.00000 0.00000

196 2S 0.00000 0.00000 0.00000 0.00000 0.00000

197 3S 0.00000 0.00000 -0.00001 -0.00002 0.00009

198 4PX 0.00000 0.00000 0.00000 0.00000 0.00000

199 4PY 0.00000 0.00000 0.00000 0.00000 0.00000

200 4PZ 0.00000 0.00000 0.00000 0.00000 0.00000

6 7 8 9 10

6 6S 0.64407

7 7PX 0.00000 0.29970

8 7PY 0.00000 0.00000 0.30190

9 7PZ 0.00000 0.00000 0.00000 0.30497

10 8PX 0.00000 0.32017 0.00000 0.00000 1.05261

11 8PY 0.00000 0.00000 0.32247 0.00000 0.00000

12 8PZ 0.00000 0.00000 0.00000 0.32553 0.00000

13 9PX 0.00000 0.00073 0.00000 0.00000 0.00548

14 9PY 0.00000 0.00000 0.00001 0.00000 0.00000

15 9PZ 0.00000 0.00000 0.00000 -0.00080 0.00000

16 10PX 0.00000 -0.00263 0.00000 0.00000 -0.03165

17 10PY 0.00000 0.00000 -0.00273 0.00000 0.00000

18 10PZ 0.00000 0.00000 0.00000 -0.00378 0.00000

19 11PX 0.00000 -0.00010 0.00000 0.00000 -0.00142

20 11PY 0.00000 0.00000 -0.00011 0.00000 0.00000

21 11PZ 0.00000 0.00000 0.00000 -0.00028 0.00000

22 12D 0 0.00000 0.00000 0.00000 0.00000 0.00000

23 12D+1 0.00000 0.00000 0.00000 0.00000 0.00000

24 12D-1 0.00000 0.00000 0.00000 0.00000 0.00000

25 12D+2 0.00000 0.00000 0.00000 0.00000 0.00000

26 12D-2 0.00000 0.00000 0.00000 0.00000 0.00000

27 2 C 1S 0.00121 0.00000 0.00000 0.00000 0.00000

28 2S 0.00221 0.00000 0.00000 0.00000 0.00000

29 2PX 0.00005 0.00000 0.00000 0.00000 0.00000

30 2PY -0.00102 0.00000 0.00000 0.00000 0.00000

31 2PZ -0.00007 0.00000 0.00000 0.00000 0.00000

32 3S -0.03266 0.00000 -0.00006 0.00000 0.00000

33 3PX 0.00023 0.00000 0.00000 0.00000 -0.00004

34 3PY -0.00611 0.00000 -0.00026 -0.00001 -0.00002

35 3PZ -0.00037 0.00000 -0.00001 -0.00001 0.00000

36 4S -0.08283 0.00000 -0.00016 0.00000 -0.00002

37 4PX 0.00058 0.00001 0.00000 0.00000 0.00008

38 4PY -0.03660 0.00000 -0.00009 -0.00001 -0.00004

39 4PZ -0.00159 0.00000 -0.00001 -0.00005 0.00000

40 5D 0 -0.00006 0.00000 0.00000 0.00000 0.00000

41 5D+1 0.00000 0.00000 0.00000 0.00000 0.00000

42 5D-1 0.00016 0.00000 -0.00001 -0.00001 0.00000

43 5D+2 0.00133 0.00000 -0.00004 0.00000 -0.00001

44 5D-2 -0.00009 -0.00001 0.00000 0.00000 -0.00011

45 3 N 1S -0.00008 0.00000 0.00000 0.00000 0.00000

46 2S -0.00014 0.00000 0.00000 0.00000 0.00000

47 2PX 0.00010 0.00000 0.00000 0.00000 0.00000

48 2PY -0.00044 0.00000 0.00000 0.00000 0.00000

49 2PZ -0.00002 0.00000 0.00000 0.00000 0.00000

50 3S 0.00315 0.00000 0.00000 0.00000 0.00000

51 3PX 0.00067 0.00000 0.00000 0.00000 0.00000

52 3PY -0.00314 0.00000 0.00000 0.00000 0.00000

53 3PZ -0.00013 0.00000 0.00000 0.00000 0.00000

54 4S 0.01204 0.00000 0.00000 0.00000 0.00001

55 4PX 0.00247 0.00000 0.00000 0.00000 0.00000

56 4PY -0.01326 0.00000 0.00002 0.00000 0.00000

57 4PZ -0.00053 0.00000 0.00000 0.00000 0.00001

58 5D 0 -0.00004 0.00000 0.00000 0.00000 0.00000

59 5D+1 0.00000 0.00000 0.00000 0.00000 0.00000

60 5D-1 -0.00002 0.00000 0.00000 0.00000 0.00000

61 5D+2 -0.00005 0.00000 0.00000 0.00000 0.00000

62 5D-2 0.00003 0.00000 0.00000 0.00000 0.00000

63 4 C 1S 0.00001 0.00000 0.00000 0.00000 0.00000

64 2S 0.00002 0.00000 0.00000 0.00000 0.00000

65 2PX -0.00028 0.00000 0.00000 0.00000 0.00000

66 2PY 0.00018 0.00000 0.00000 0.00000 0.00000

67 2PZ -0.00001 0.00000 0.00000 0.00000 0.00000

68 3S -0.00043 0.00000 0.00000 0.00000 0.00000

69 3PX -0.00222 0.00000 0.00000 0.00000 0.00000

70 3PY 0.00146 0.00000 0.00000 0.00000 0.00000

71 3PZ -0.00009 0.00000 0.00000 0.00000 0.00000

72 4S 0.00045 0.00001 0.00001 0.00000 0.00013

73 4PX -0.00056 0.00001 0.00000 0.00000 0.00009

74 4PY 0.00254 0.00001 0.00000 0.00000 0.00018

75 4PZ -0.00059 0.00000 0.00000 0.00000 0.00003

76 5D 0 -0.00003 0.00000 0.00000 0.00000 0.00000

77 5D+1 -0.00003 0.00000 0.00000 0.00000 0.00000

78 5D-1 0.00000 0.00000 0.00000 0.00000 0.00000

79 5D+2 -0.00004 0.00000 0.00000 0.00000 0.00000

80 5D-2 -0.00010 0.00000 0.00000 0.00000 0.00000

81 5 C 1S 0.00030 0.00000 0.00000 0.00000 0.00000

82 2S 0.00053 0.00000 0.00000 0.00000 0.00000

83 2PX -0.00077 0.00000 0.00000 0.00000 -0.00001

84 2PY -0.00017 0.00000 0.00000 0.00000 0.00000

85 2PZ -0.00007 0.00000 0.00000 0.00000 0.00000

86 3S -0.00839 -0.00001 0.00000 0.00000 -0.00026

87 3PX -0.00339 -0.00008 0.00000 0.00000 -0.00140

88 3PY -0.00095 0.00000 0.00000 0.00000 -0.00008

89 3PZ -0.00034 0.00000 0.00000 0.00000 -0.00006

90 4S -0.02765 -0.00012 0.00000 -0.00001 -0.00165

91 4PX -0.03797 -0.00015 0.00001 -0.00001 -0.00206

92 4PY -0.00139 0.00000 -0.00001 0.00000 0.00002

93 4PZ -0.00137 -0.00001 0.00000 -0.00002 -0.00009

94 5D 0 -0.00002 0.00000 0.00000 0.00000 -0.00002

95 5D+1 0.00010 0.00000 0.00000 0.00000 -0.00002

96 5D-1 0.00001 0.00000 0.00000 0.00000 0.00000

97 5D+2 0.00028 -0.00001 0.00000 0.00000 -0.00012

98 5D-2 0.00034 0.00000 0.00000 0.00000 -0.00001

99 6 C 1S 0.00000 0.00000 0.00000 0.00000 0.00000

100 2S 0.00000 0.00000 0.00000 0.00000 0.00000

101 2PX 0.00000 0.00000 0.00000 0.00000 0.00000

102 2PY 0.00000 0.00000 0.00000 0.00000 0.00000

103 2PZ 0.00000 0.00000 0.00000 0.00000 0.00000

104 3S -0.00001 0.00000 0.00000 0.00000 0.00000

105 3PX 0.00002 0.00000 0.00000 0.00000 0.00000

106 3PY 0.00001 0.00000 0.00000 0.00000 0.00000

107 3PZ 0.00000 0.00000 0.00000 0.00000 0.00000

108 4S 0.00006 0.00000 0.00000 0.00000 0.00000

109 4PX 0.00016 0.00000 0.00000 0.00000 0.00000

110 4PY 0.00041 0.00000 0.00000 0.00000 0.00000

111 4PZ 0.00003 0.00000 0.00000 0.00000 0.00000

112 5D 0 0.00000 0.00000 0.00000 0.00000 0.00000

113 5D+1 0.00000 0.00000 0.00000 0.00000 0.00000

114 5D-1 0.00000 0.00000 0.00000 0.00000 0.00000

115 5D+2 0.00000 0.00000 0.00000 0.00000 0.00000

116 5D-2 0.00001 0.00000 0.00000 0.00000 0.00000

117 7 C 1S 0.00000 0.00000 0.00000 0.00000 0.00000

118 2S 0.00000 0.00000 0.00000 0.00000 0.00000

119 2PX 0.00000 0.00000 0.00000 0.00000 0.00000

120 2PY 0.00000 0.00000 0.00000 0.00000 0.00000

121 2PZ 0.00000 0.00000 0.00000 0.00000 0.00000

122 3S 0.00000 0.00000 0.00000 0.00000 0.00000

123 3PX 0.00000 0.00000 0.00000 0.00000 0.00000

124 3PY 0.00000 0.00000 0.00000 0.00000 0.00000

125 3PZ 0.00000 0.00000 0.00000 0.00000 0.00000

126 4S -0.00012 0.00000 0.00000 0.00000 0.00000

127 4PX -0.00001 0.00000 0.00000 0.00000 0.00000

128 4PY 0.00002 0.00000 0.00000 0.00000 0.00000

129 4PZ -0.00002 0.00000 0.00000 0.00000 0.00000

130 5D 0 0.00000 0.00000 0.00000 0.00000 0.00000

131 5D+1 0.00000 0.00000 0.00000 0.00000 0.00000

132 5D-1 0.00000 0.00000 0.00000 0.00000 0.00000

133 5D+2 0.00000 0.00000 0.00000 0.00000 0.00000

134 5D-2 0.00000 0.00000 0.00000 0.00000 0.00000

135 8 C 1S 0.00000 0.00000 0.00000 0.00000 0.00000

136 2S 0.00000 0.00000 0.00000 0.00000 0.00000

137 2PX 0.00000 0.00000 0.00000 0.00000 0.00000

138 2PY 0.00000 0.00000 0.00000 0.00000 0.00000

139 2PZ 0.00000 0.00000 0.00000 0.00000 0.00000

140 3S 0.00000 0.00000 0.00000 0.00000 0.00000

141 3PX 0.00002 0.00000 0.00000 0.00000 0.00000

142 3PY 0.00000 0.00000 0.00000 0.00000 0.00000

143 3PZ 0.00000 0.00000 0.00000 0.00000 0.00000

144 4S -0.00006 0.00000 0.00000 0.00000 0.00000

145 4PX 0.00014 0.00000 0.00000 0.00000 0.00000

146 4PY 0.00000 0.00000 0.00000 0.00000 0.00000

147 4PZ 0.00001 0.00000 0.00000 0.00000 0.00000

148 5D 0 0.00000 0.00000 0.00000 0.00000 0.00000

149 5D+1 0.00000 0.00000 0.00000 0.00000 0.00000

150 5D-1 0.00000 0.00000 0.00000 0.00000 0.00000

151 5D+2 0.00000 0.00000 0.00000 0.00000 0.00000

152 5D-2 0.00000 0.00000 0.00000 0.00000 0.00000

153 9 C 1S 0.00002 0.00000 0.00000 0.00000 0.00000

154 2S 0.00003 0.00000 0.00000 0.00000 0.00000

155 2PX -0.00015 0.00000 0.00000 0.00000 0.00000

156 2PY 0.00000 0.00000 0.00000 0.00000 0.00000

157 2PZ 0.00000 0.00000 0.00000 0.00000 0.00000

158 3S -0.00074 0.00000 0.00000 0.00000 0.00000

159 3PX -0.00104 0.00000 0.00000 0.00000 0.00000

160 3PY 0.00000 0.00000 0.00000 0.00000 0.00000

161 3PZ -0.00003 0.00000 0.00000 0.00000 0.00000

162 4S -0.00446 0.00000 0.00000 0.00000 0.00004

163 4PX -0.00378 0.00000 0.00000 0.00000 -0.00001

164 4PY -0.00021 0.00000 0.00000 0.00000 0.00002

165 4PZ -0.00013 0.00000 0.00000 0.00000 0.00001

166 5D 0 0.00000 0.00000 0.00000 0.00000 0.00000

167 5D+1 0.00000 0.00000 0.00000 0.00000 0.00000

168 5D-1 0.00000 0.00000 0.00000 0.00000 0.00000

169 5D+2 -0.00007 0.00000 0.00000 0.00000 0.00000

170 5D-2 0.00001 0.00000 0.00000 0.00000 0.00000

171 10 H 1S -0.00212 0.00000 0.00000 0.00000 0.00000

172 2S -0.01069 0.00000 0.00000 0.00000 0.00001

173 3S -0.00881 0.00000 0.00002 0.00000 0.00005

174 4PX -0.00015 0.00000 0.00000 0.00000 0.00000

175 4PY -0.00021 0.00000 0.00000 0.00000 0.00000

176 4PZ 0.00000 0.00000 0.00000 0.00000 0.00000

177 11 H 1S 0.00000 0.00000 0.00000 0.00000 0.00000

178 2S 0.00000 0.00000 0.00000 0.00000 0.00000

179 3S 0.00012 0.00000 0.00000 0.00000 0.00000

180 4PX 0.00000 0.00000 0.00000 0.00000 0.00000

181 4PY 0.00000 0.00000 0.00000 0.00000 0.00000

182 4PZ 0.00000 0.00000 0.00000 0.00000 0.00000

183 12 H 1S 0.00000 0.00000 0.00000 0.00000 0.00000

184 2S 0.00000 0.00000 0.00000 0.00000 0.00000

185 3S 0.00001 0.00000 0.00000 0.00000 0.00000

186 4PX 0.00000 0.00000 0.00000 0.00000 0.00000

187 4PY 0.00000 0.00000 0.00000 0.00000 0.00000

188 4PZ 0.00000 0.00000 0.00000 0.00000 0.00000

189 13 H 1S 0.00000 0.00000 0.00000 0.00000 0.00000

190 2S 0.00000 0.00000 0.00000 0.00000 0.00000

191 3S 0.00000 0.00000 0.00000 0.00000 0.00000

192 4PX 0.00000 0.00000 0.00000 0.00000 0.00000

193 4PY 0.00000 0.00000 0.00000 0.00000 0.00000

194 4PZ 0.00000 0.00000 0.00000 0.00000 0.00000

195 14 H 1S -0.00002 0.00000 0.00000 0.00000 0.00000

196 2S 0.00002 0.00000 0.00000 0.00000 0.00000

197 3S 0.00170 0.00000 0.00000 0.00000 0.00003

198 4PX 0.00000 0.00000 0.00000 0.00000 0.00000

199 4PY -0.00001 0.00000 0.00000 0.00000 0.00000

200 4PZ 0.00000 0.00000 0.00000 0.00000 0.00000

11 12 13 14 15

11 8PY 1.06002

12 8PZ 0.00000 1.06928

13 9PX 0.00000 0.00000 0.11652

14 9PY -0.00098 0.00000 0.00000 0.13460

15 9PZ 0.00000 -0.00762 0.00000 0.00000 0.14598

16 10PX 0.00000 0.00000 0.14090 0.00000 0.00000

17 10PY -0.03276 0.00000 0.00000 0.14880 0.00000

18 10PZ 0.00000 -0.04495 0.00000 0.00000 0.20790

19 11PX 0.00000 0.00000 0.01054 0.00000 0.00000

20 11PY -0.00150 0.00000 0.00000 0.01102 0.00000

21 11PZ 0.00000 -0.00383 0.00000 0.00000 0.02720

22 12D 0 0.00000 0.00000 0.00000 0.00000 0.00000

23 12D+1 0.00000 0.00000 0.00000 0.00000 0.00000

24 12D-1 0.00000 0.00000 0.00000 0.00000 0.00000

25 12D+2 0.00000 0.00000 0.00000 0.00000 0.00000

26 12D-2 0.00000 0.00000 0.00000 0.00000 0.00000

27 2 C 1S 0.00000 0.00000 0.00000 -0.00012 0.00000

28 2S 0.00000 0.00000 0.00000 -0.00015 0.00000

29 2PX 0.00000 0.00000 0.00002 0.00002 0.00000

30 2PY -0.00008 0.00000 0.00002 0.00390 0.00017

31 2PZ 0.00000 0.00000 0.00000 0.00018 0.00012

32 3S -0.00113 -0.00003 0.00003 0.01190 0.00030

33 3PX -0.00003 0.00000 0.00031 0.00021 -0.00001

34 3PY -0.00406 -0.00020 0.00016 0.02963 0.00152

35 3PZ -0.00019 -0.00020 -0.00001 0.00147 0.00223

36 4S -0.00218 -0.00004 0.00005 0.01595 0.00038

37 4PX -0.00006 0.00000 0.00005 0.00036 0.00000

38 4PY -0.00120 -0.00011 0.00024 0.00825 0.00077

39 4PZ -0.00008 -0.00066 0.00000 0.00062 0.00467

40 5D 0 -0.00005 -0.00004 0.00000 0.00028 0.00031

41 5D+1 0.00000 0.00000 0.00002 0.00001 -0.00001

42 5D-1 -0.00009 -0.00011 0.00000 0.00053 0.00123

43 5D+2 -0.00057 -0.00004 0.00004 0.00308 0.00025

44 5D-2 -0.00001 0.00000 0.00113 0.00008 0.00000

45 3 N 1S 0.00000 0.00000 0.00000 0.00000 0.00000

46 2S 0.00000 0.00000 0.00000 0.00000 0.00000

47 2PX 0.00000 0.00000 0.00000 0.00000 0.00000

48 2PY 0.00000 0.00000 0.00000 0.00000 0.00000

49 2PZ 0.00000 0.00000 0.00000 0.00000 0.00000

50 3S 0.00000 0.00000 0.00000 0.00000 0.00000

51 3PX 0.00000 0.00000 0.00000 0.00000 0.00000

52 3PY 0.00000 0.00000 0.00000 -0.00005 -0.00001

53 3PZ 0.00000 0.00000 0.00000 -0.00001 0.00000

54 4S -0.00001 0.00000 -0.00011 0.00017 0.00002

55 4PX 0.00002 0.00001 0.00000 -0.00014 -0.00005

56 4PY 0.00028 0.00003 -0.00003 -0.00254 -0.00029

57 4PZ 0.00004 0.00003 -0.00007 -0.00031 -0.00037

58 5D 0 0.00000 0.00000 0.00000 0.00000 0.00000

59 5D+1 0.00000 0.00000 0.00000 0.00000 0.00000

60 5D-1 0.00000 0.00000 0.00000 0.00000 0.00000

61 5D+2 0.00000 0.00000 0.00000 0.00000 0.00000

62 5D-2 0.00000 0.00000 0.00000 0.00000 0.00000

63 4 C 1S 0.00000 0.00000 0.00000 0.00000 0.00000

64 2S 0.00000 0.00000 0.00000 0.00000 0.00000

65 2PX 0.00000 0.00000 0.00000 0.00000 0.00000

66 2PY 0.00000 0.00000 0.00000 0.00000 0.00000

67 2PZ 0.00000 0.00000 0.00000 0.00000 0.00000

68 3S 0.00000 0.00000 -0.00002 -0.00002 0.00000

69 3PX 0.00000 0.00000 -0.00002 -0.00004 0.00000

70 3PY 0.00000 0.00000 -0.00006 0.00000 0.00000

71 3PZ 0.00000 0.00000 -0.00001 0.00000 0.00000

72 4S 0.00012 0.00001 -0.00089 -0.00123 -0.00009

73 4PX 0.00006 0.00003 -0.00098 -0.00063 -0.00034

74 4PY 0.00002 0.00003 -0.00111 -0.00028 -0.00031

75 4PZ 0.00002 0.00004 -0.00022 -0.00021 -0.00044

76 5D 0 0.00000 0.00000 0.00000 0.00000 0.00000

77 5D+1 0.00000 0.00000 0.00000 0.00000 0.00000

78 5D-1 0.00000 0.00000 0.00000 0.00000 0.00000

79 5D+2 0.00000 0.00000 0.00000 0.00000 0.00000

80 5D-2 0.00000 0.00000 0.00002 0.00000 0.00000

81 5 C 1S 0.00000 0.00000 -0.00002 0.00000 0.00000

82 2S 0.00000 0.00000 -0.00001 0.00000 0.00000

83 2PX 0.00000 0.00000 0.00092 -0.00001 0.00003

84 2PY 0.00000 0.00000 0.00005 0.00000 0.00000

85 2PZ 0.00000 0.00000 0.00004 0.00000 0.00001

86 3S 0.00000 -0.00001 0.00364 -0.00001 0.00017

87 3PX 0.00001 -0.00005 0.01440 -0.00013 0.00051

88 3PY 0.00000 0.00000 0.00080 0.00001 0.00004

89 3PZ 0.00000 -0.00002 0.00067 0.00001 0.00036

90 4S -0.00003 -0.00009 0.01150 0.00023 0.00062

91 4PX 0.00014 -0.00013 0.01508 -0.00060 0.00104

92 4PY -0.00015 0.00000 0.00050 0.00171 0.00003

93 4PZ 0.00000 -0.00033 0.00081 0.00000 0.00235

94 5D 0 0.00000 -0.00001 0.00016 0.00001 0.00009

95 5D+1 0.00000 -0.00001 0.00024 0.00001 0.00024

96 5D-1 0.00000 0.00000 -0.00001 0.00001 0.00001

97 5D+2 -0.00001 -0.00001 0.00120 0.00011 0.00009

98 5D-2 -0.00001 0.00000 0.00015 0.00023 0.00001

99 6 C 1S 0.00000 0.00000 0.00000 0.00000 0.00000

100 2S 0.00000 0.00000 0.00000 0.00000 0.00000

101 2PX 0.00000 0.00000 0.00000 0.00000 0.00000

102 2PY 0.00000 0.00000 0.00000 0.00000 0.00000

103 2PZ 0.00000 0.00000 0.00000 0.00000 0.00000

104 3S 0.00000 0.00000 0.00000 0.00000 0.00000

105 3PX 0.00000 0.00000 0.00000 0.00000 0.00000

106 3PY 0.00000 0.00000 0.00000 0.00000 0.00000

107 3PZ 0.00000 0.00000 0.00000 0.00000 0.00000

108 4S 0.00000 0.00000 0.00001 0.00000 0.00000

109 4PX 0.00000 0.00000 0.00003 0.00002 0.00000

110 4PY 0.00000 0.00000 0.00001 0.00000 0.00000

111 4PZ 0.00000 0.00000 0.00000 0.00000 0.00000

112 5D 0 0.00000 0.00000 0.00000 0.00000 0.00000

113 5D+1 0.00000 0.00000 0.00000 0.00000 0.00000

114 5D-1 0.00000 0.00000 0.00000 0.00000 0.00000

115 5D+2 0.00000 0.00000 0.00000 0.00000 0.00000

116 5D-2 0.00000 0.00000 0.00000 0.00000 0.00000

117 7 C 1S 0.00000 0.00000 0.00000 0.00000 0.00000

118 2S 0.00000 0.00000 0.00000 0.00000 0.00000

119 2PX 0.00000 0.00000 0.00000 0.00000 0.00000

120 2PY 0.00000 0.00000 0.00000 0.00000 0.00000

121 2PZ 0.00000 0.00000 0.00000 0.00000 0.00000

122 3S 0.00000 0.00000 0.00000 0.00000 0.00000

123 3PX 0.00000 0.00000 0.00000 0.00000 0.00000

124 3PY 0.00000 0.00000 0.00000 0.00000 0.00000

125 3PZ 0.00000 0.00000 0.00000 0.00000 0.00000

126 4S 0.00000 0.00000 0.00000 0.00000 0.00000

127 4PX 0.00000 0.00000 0.00000 0.00000 0.00000

128 4PY 0.00000 0.00000 0.00000 0.00000 0.00000

129 4PZ 0.00000 0.00000 0.00000 0.00000 0.00000

130 5D 0 0.00000 0.00000 0.00000 0.00000 0.00000

131 5D+1 0.00000 0.00000 0.00000 0.00000 0.00000

132 5D-1 0.00000 0.00000 0.00000 0.00000 0.00000

133 5D+2 0.00000 0.00000 0.00000 0.00000 0.00000

134 5D-2 0.00000 0.00000 0.00000 0.00000 0.00000

135 8 C 1S 0.00000 0.00000 0.00000 0.00000 0.00000

136 2S 0.00000 0.00000 0.00000 0.00000 0.00000

137 2PX 0.00000 0.00000 0.00000 0.00000 0.00000

138 2PY 0.00000 0.00000 0.00000 0.00000 0.00000

139 2PZ 0.00000 0.00000 0.00000 0.00000 0.00000

140 3S 0.00000 0.00000 0.00000 0.00000 0.00000

141 3PX 0.00000 0.00000 0.00000 0.00000 0.00000

142 3PY 0.00000 0.00000 0.00000 0.00000 0.00000

143 3PZ 0.00000 0.00000 0.00000 0.00000 0.00000

144 4S 0.00000 0.00000 0.00001 0.00000 0.00000

145 4PX 0.00000 0.00000 0.00004 0.00000 0.00000

146 4PY 0.00000 0.00000 0.00000 0.00000 0.00000

147 4PZ 0.00000 0.00000 0.00000 0.00000 0.00000

148 5D 0 0.00000 0.00000 0.00000 0.00000 0.00000

149 5D+1 0.00000 0.00000 0.00000 0.00000 0.00000

150 5D-1 0.00000 0.00000 0.00000 0.00000 0.00000

151 5D+2 0.00000 0.00000 0.00000 0.00000 0.00000

152 5D-2 0.00000 0.00000 0.00000 0.00000 0.00000

153 9 C 1S 0.00000 0.00000 0.00000 0.00000 0.00000

154 2S 0.00000 0.00000 0.00000 0.00000 0.00000

155 2PX 0.00000 0.00000 0.00000 0.00000 0.00000

156 2PY 0.00000 0.00000 0.00000 0.00000 0.00000

157 2PZ 0.00000 0.00000 0.00000 0.00000 0.00000

158 3S 0.00000 0.00000 -0.00001 0.00000 0.00000

159 3PX 0.00000 0.00000 -0.00002 0.00000 0.00000

160 3PY 0.00000 0.00000 0.00000 0.00000 0.00000

161 3PZ 0.00000 0.00000 0.00000 0.00000 0.00000

162 4S 0.00000 0.00000 -0.00035 -0.00005 0.00002

163 4PX 0.00000 0.00001 -0.00053 -0.00007 -0.00008

164 4PY 0.00000 0.00000 -0.00007 -0.00001 0.00002

165 4PZ 0.00000 0.00002 -0.00008 0.00001 -0.00021

166 5D 0 0.00000 0.00000 0.00000 0.00000 0.00000

167 5D+1 0.00000 0.00000 0.00000 0.00000 0.00000

168 5D-1 0.00000 0.00000 0.00000 0.00000 0.00000

169 5D+2 0.00000 0.00000 0.00001 0.00000 0.00000

170 5D-2 0.00000 0.00000 0.00000 0.00000 0.00000

171 10 H 1S 0.00000 0.00000 0.00000 -0.00001 0.00000

172 2S 0.00003 0.00000 -0.00015 -0.00053 0.00000

173 3S 0.00026 0.00000 -0.00047 -0.00205 -0.00003

174 4PX 0.00000 0.00000 0.00000 0.00000 0.00000

175 4PY 0.00000 0.00000 0.00001 0.00003 0.00000

176 4PZ 0.00000 0.00000 0.00000 0.00000 0.00000

177 11 H 1S 0.00000 0.00000 0.00000 0.00000 0.00000

178 2S 0.00000 0.00000 0.00000 0.00000 0.00000

179 3S 0.00000 0.00000 0.00000 0.00000 0.00000

180 4PX 0.00000 0.00000 0.00000 0.00000 0.00000

181 4PY 0.00000 0.00000 0.00000 0.00000 0.00000

182 4PZ 0.00000 0.00000 0.00000 0.00000 0.00000

183 12 H 1S 0.00000 0.00000 0.00000 0.00000 0.00000

184 2S 0.00000 0.00000 0.00000 0.00000 0.00000

185 3S 0.00000 0.00000 0.00000 0.00000 0.00000

186 4PX 0.00000 0.00000 0.00000 0.00000 0.00000

187 4PY 0.00000 0.00000 0.00000 0.00000 0.00000

188 4PZ 0.00000 0.00000 0.00000 0.00000 0.00000

189 13 H 1S 0.00000 0.00000 0.00000 0.00000 0.00000

190 2S 0.00000 0.00000 0.00000 0.00000 0.00000

191 3S 0.00000 0.00000 0.00000 0.00000 0.00000

192 4PX 0.00000 0.00000 0.00000 0.00000 0.00000

193 4PY 0.00000 0.00000 0.00000 0.00000 0.00000

194 4PZ 0.00000 0.00000 0.00000 0.00000 0.00000

195 14 H 1S 0.00000 0.00000 0.00000 0.00000 0.00000

196 2S 0.00000 0.00000 -0.00001 0.00000 0.00000

197 3S 0.00000 0.00000 -0.00022 0.00001 -0.00001

198 4PX 0.00000 0.00000 0.00000 0.00000 0.00000

199 4PY 0.00000 0.00000 0.00000 0.00000 0.00000

200 4PZ 0.00000 0.00000 0.00000 0.00000 0.00000

16 17 18 19 20

16 10PX 0.36591

17 10PY 0.00000 0.35687

18 10PZ 0.00000 0.00000 0.62164

19 11PX 0.05130 0.00000 0.00000 0.02367

20 11PY 0.00000 0.05269 0.00000 0.00000 0.02867

21 11PZ 0.00000 0.00000 0.15088 0.00000 0.00000

22 12D 0 0.00000 0.00000 0.00000 0.00000 0.00000

23 12D+1 0.00000 0.00000 0.00000 0.00000 0.00000

24 12D-1 0.00000 0.00000 0.00000 0.00000 0.00000

25 12D+2 0.00000 0.00000 0.00000 0.00000 0.00000

26 12D-2 0.00000 0.00000 0.00000 0.00000 0.00000

27 2 C 1S -0.00001 -0.00194 -0.00004 0.00001 0.00014

28 2S -0.00001 -0.00368 -0.00009 0.00001 0.00015

29 2PX 0.00025 0.00010 -0.00001 -0.00031 0.00001

30 2PY 0.00016 0.01851 0.00099 0.00000 0.00047

31 2PZ 0.00000 0.00100 0.00207 0.00000 0.00010

32 3S 0.00011 0.04887 0.00110 -0.00016 -0.00059

33 3PX 0.00120 0.00059 -0.00004 -0.00170 0.00005

34 3PY 0.00057 0.07411 0.00475 -0.00003 0.00167

35 3PZ -0.00003 0.00452 0.01421 -0.00001 0.00054

36 4S 0.00018 0.06076 0.00134 -0.00025 0.00967

37 4PX -0.00030 0.00130 -0.00001 -0.00169 0.00036

38 4PY 0.00087 0.01795 0.00253 0.00009 -0.00020

39 4PZ -0.00001 0.00172 0.02186 0.00000 0.00028

40 5D 0 0.00001 0.00017 0.00096 0.00000 0.00002

41 5D+1 0.00006 0.00001 -0.00004 0.00000 0.00000

42 5D-1 -0.00001 0.00094 0.00595 0.00000 0.00001

43 5D+2 0.00009 0.00373 0.00054 0.00000 -0.00029

44 5D-2 0.00414 0.00013 -0.00001 0.00042 0.00000

45 3 N 1S 0.00000 0.00000 0.00000 0.00000 -0.00017

46 2S 0.00000 -0.00001 0.00000 0.00001 -0.00031

47 2PX 0.00000 -0.00001 -0.00001 -0.00001 0.00004

48 2PY -0.00002 -0.00039 -0.00004 -0.00011 -0.00092

49 2PZ -0.00001 -0.00004 -0.00002 -0.00003 -0.00011

50 3S -0.00016 0.00023 0.00002 -0.00022 0.00529

51 3PX -0.00001 -0.00011 -0.00007 -0.00009 0.00030

52 3PY -0.00023 -0.00368 -0.00043 -0.00063 -0.00508

53 3PZ -0.00009 -0.00041 -0.00032 -0.00020 -0.00067

54 4S -0.00088 0.00420 0.00042 -0.00021 0.01414

55 4PX -0.00005 -0.00050 -0.00043 -0.00002 0.00056

56 4PY -0.00038 -0.02128 -0.00259 -0.00131 -0.01401

57 4PZ -0.00065 -0.00273 -0.00518 -0.00066 -0.00224

58 5D 0 0.00001 -0.00006 0.00000 0.00000 -0.00002

59 5D+1 0.00000 0.00000 0.00001 0.00000 0.00000

60 5D-1 0.00000 -0.00001 -0.00001 -0.00001 -0.00002

61 5D+2 -0.00004 0.00008 -0.00001 -0.00003 -0.00001

62 5D-2 0.00000 0.00002 0.00000 -0.00001 0.00001

63 4 C 1S 0.00001 0.00001 0.00000 0.00008 0.00012

64 2S 0.00001 0.00001 0.00000 0.00014 0.00022

65 2PX -0.00008 -0.00010 -0.00002 -0.00017 -0.00036

66 2PY -0.00017 0.00002 -0.00001 -0.00040 0.00001

67 2PZ -0.00002 -0.00001 -0.00001 -0.00008 -0.00007

68 3S -0.00084 -0.00076 -0.00013 -0.00193 -0.00342

69 3PX -0.00078 -0.00133 -0.00015 -0.00085 -0.00230

70 3PY -0.00178 0.00028 -0.00010 -0.00215 0.00008

71 3PZ -0.00020 -0.00014 -0.00024 -0.00043 -0.00039

72 4S -0.00691 -0.00959 -0.00052 -0.00603 -0.01245

73 4PX -0.00522 -0.00307 -0.00230 -0.00100 -0.00144

74 4PY -0.00679 -0.00117 -0.00208 -0.00343 0.00001

75 4PZ -0.00154 -0.00135 -0.00434 -0.00148 -0.00153

76 5D 0 0.00000 0.00000 0.00001 0.00000 0.00000

77 5D+1 0.00000 -0.00002 -0.00002 -0.00001 -0.00003

78 5D-1 0.00002 0.00002 0.00002 0.00000 0.00000

79 5D+2 0.00000 -0.00009 -0.00001 -0.00002 -0.00013

80 5D-2 0.00048 0.00004 0.00003 0.00008 0.00000

81 5 C 1S -0.00091 -0.00001 -0.00005 0.00002 -0.00003

82 2S -0.00168 -0.00002 -0.00009 -0.00003 -0.00005

83 2PX 0.01209 -0.00008 0.00045 0.00085 0.00003

84 2PY 0.00076 0.00014 0.00004 0.00013 -0.00015

85 2PZ 0.00062 0.00002 0.00053 0.00006 0.00000

86 3S 0.02818 0.00030 0.00158 0.00094 0.00082

87 3PX 0.06182 -0.00033 0.00256 0.00403 0.00021

88 3PY 0.00410 0.00089 0.00023 0.00070 -0.00084

89 3PZ 0.00348 0.00010 0.00423 0.00033 0.00001

90 4S 0.05702 0.00209 0.00374 0.01402 0.00299

91 4PX 0.04918 -0.00264 0.00429 0.00244 -0.00091

92 4PY 0.00213 0.01030 0.00016 0.00077 0.00282

93 4PZ 0.00352 0.00004 0.01522 0.00046 -0.00005

94 5D 0 0.00032 0.00000 0.00043 0.00000 0.00000

95 5D+1 0.00072 0.00005 0.00202 0.00002 0.00001

96 5D-1 -0.00002 0.00004 0.00007 0.00000 0.00000

97 5D+2 0.00281 0.00038 0.00032 -0.00008 0.00007

98 5D-2 0.00042 0.00127 0.00004 0.00001 0.00010

99 6 C 1S 0.00000 0.00000 0.00000 -0.00001 0.00000

100 2S 0.00000 0.00000 0.00000 -0.00001 0.00000

101 2PX 0.00000 0.00000 0.00000 0.00006 0.00001

102 2PY 0.00000 0.00000 0.00000 0.00004 0.00001

103 2PZ 0.00000 0.00000 0.00000 0.00000 0.00000

104 3S 0.00000 0.00000 0.00000 0.00030 0.00003

105 3PX 0.00001 0.00000 0.00000 0.00040 0.00006

106 3PY 0.00001 0.00000 0.00000 0.00028 0.00004

107 3PZ 0.00000 0.00000 0.00000 -0.00001 0.00000

108 4S 0.00015 0.00005 -0.00002 0.00092 0.00062

109 4PX 0.00043 0.00034 -0.00005 0.00163 0.00119

110 4PY 0.00021 0.00007 0.00002 0.00076 0.00029

111 4PZ -0.00003 0.00000 0.00005 -0.00002 0.00003

112 5D 0 0.00000 0.00000 0.00000 -0.00001 -0.00001

113 5D+1 0.00000 0.00000 0.00000 0.00000 0.00000

114 5D-1 0.00000 0.00000 0.00000 0.00000 0.00000

115 5D+2 0.00000 0.00000 0.00000 0.00000 -0.00001

116 5D-2 0.00000 0.00000 0.00000 -0.00001 0.00000

117 7 C 1S 0.00000 0.00000 0.00000 0.00000 0.00000

118 2S 0.00000 0.00000 0.00000 0.00000 0.00000

119 2PX 0.00000 0.00000 0.00000 0.00001 0.00001

120 2PY 0.00000 0.00000 0.00000 0.00000 0.00000

121 2PZ 0.00000 0.00000 0.00000 0.00000 0.00000

122 3S 0.00000 0.00000 0.00000 0.00004 0.00000

123 3PX 0.00000 0.00000 0.00000 0.00012 0.00007

124 3PY 0.00000 0.00000 0.00000 0.00003 0.00000

125 3PZ 0.00000 0.00000 0.00000 -0.00001 -0.00001

126 4S 0.00003 0.00000 0.00000 0.00029 -0.00005

127 4PX 0.00002 0.00002 0.00003 0.00055 0.00030

128 4PY 0.00000 0.00000 0.00001 0.00009 -0.00001

129 4PZ 0.00000 0.00000 0.00003 -0.00007 -0.00004

130 5D 0 0.00000 0.00000 0.00000 0.00000 0.00000

131 5D+1 0.00000 0.00000 0.00000 0.00000 0.00000

132 5D-1 0.00000 0.00000 0.00000 0.00000 0.00000

133 5D+2 0.00000 0.00000 0.00000 0.00001 0.00000

134 5D-2 0.00000 0.00000 0.00000 0.00000 0.00000

135 8 C 1S 0.00000 0.00000 0.00000 -0.00001 0.00000

136 2S 0.00000 0.00000 0.00000 -0.00001 0.00000

137 2PX 0.00000 0.00000 0.00000 0.00008 0.00000

138 2PY 0.00000 0.00000 0.00000 0.00000 0.00000

139 2PZ 0.00000 0.00000 0.00000 0.00000 0.00000

140 3S 0.00000 0.00000 0.00000 0.00026 0.00001

141 3PX 0.00001 0.00000 0.00000 0.00053 0.00001

142 3PY 0.00000 0.00000 0.00000 0.00000 -0.00002

143 3PZ 0.00000 0.00000 0.00000 -0.00001 0.00000

144 4S 0.00026 0.00000 -0.00002 0.00121 0.00000

145 4PX 0.00077 0.00000 -0.00007 0.00235 0.00000

146 4PY 0.00000 0.00001 -0.00001 0.00001 -0.00008

147 4PZ -0.00001 0.00000 -0.00003 -0.00006 -0.00001

148 5D 0 0.00000 0.00000 0.00000 0.00000 0.00000

149 5D+1 0.00000 0.00000 0.00000 -0.00001 0.00000

150 5D-1 0.00000 0.00000 0.00000 0.00000 0.00000

151 5D+2 0.00000 0.00000 0.00000 0.00000 0.00000

152 5D-2 0.00000 0.00000 0.00000 0.00000 0.00000

153 9 C 1S 0.00000 0.00000 0.00000 0.00008 0.00000

154 2S 0.00001 0.00000 0.00000 0.00014 0.00000

155 2PX -0.00009 -0.00001 0.00000 -0.00039 -0.00002

156 2PY -0.00001 0.00000 0.00000 -0.00003 0.00002

157 2PZ -0.00001 0.00000 0.00000 -0.00005 0.00000

158 3S -0.00053 -0.00003 0.00001 -0.00204 0.00003

159 3PX -0.00130 -0.00008 -0.00006 -0.00208 -0.00015

160 3PY -0.00010 -0.00001 0.00001 -0.00016 0.00010

161 3PZ -0.00009 0.00000 -0.00009 -0.00027 0.00000

162 4S -0.00337 -0.00014 0.00019 -0.00472 0.00100

163 4PX -0.00336 -0.00037 -0.00067 -0.00246 0.00006

164 4PY -0.00056 -0.00034 0.00012 -0.00040 -0.00044

165 4PZ -0.00062 0.00005 -0.00239 -0.00079 0.00001

166 5D 0 -0.00001 0.00000 0.00000 -0.00001 0.00000

167 5D+1 0.00002 0.00000 0.00002 0.00001 0.00000

168 5D-1 0.00000 0.00000 0.00000 0.00000 0.00000

169 5D+2 0.00033 0.00002 0.00002 0.00011 -0.00001

170 5D-2 -0.00004 0.00000 0.00000 -0.00004 0.00002

171 10 H 1S -0.00021 -0.00103 -0.00001 -0.00061 -0.00252

172 2S -0.00180 -0.00691 -0.00006 -0.00214 -0.00955

173 3S -0.00271 -0.01152 -0.00015 -0.00148 -0.00876

174 4PX 0.00001 0.00004 0.00000 0.00002 -0.00006

175 4PY 0.00015 0.00055 0.00002 -0.00002 0.00007

176 4PZ 0.00000 0.00002 0.00006 0.00000 0.00001

177 11 H 1S 0.00000 0.00000 0.00000 -0.00001 0.00000

178 2S 0.00000 0.00000 0.00000 -0.00009 -0.00001

179 3S -0.00006 0.00004 0.00000 -0.00027 0.00031

180 4PX 0.00000 0.00000 0.00000 0.00000 0.00000

181 4PY 0.00000 0.00000 0.00000 0.00000 0.00000

182 4PZ 0.00000 0.00000 0.00000 0.00000 0.00000

183 12 H 1S 0.00000 0.00000 0.00000 0.00000 0.00000

184 2S 0.00000 0.00000 0.00000 0.00000 0.00000

185 3S 0.00000 0.00000 0.00000 -0.00002 -0.00001

186 4PX 0.00000 0.00000 0.00000 0.00000 0.00000

187 4PY 0.00000 0.00000 0.00000 0.00000 0.00000

188 4PZ 0.00000 0.00000 0.00000 0.00000 0.00000

189 13 H 1S 0.00000 0.00000 0.00000 -0.00001 0.00000

190 2S 0.00000 0.00000 0.00000 -0.00005 0.00000

191 3S -0.00002 0.00000 0.00000 -0.00019 -0.00002

192 4PX 0.00000 0.00000 0.00000 0.00000 0.00000

193 4PY 0.00000 0.00000 0.00000 0.00000 0.00000

194 4PZ 0.00000 0.00000 0.00000 0.00000 0.00000

195 14 H 1S -0.00001 0.00000 0.00000 -0.00027 0.00007

196 2S -0.00028 -0.00006 -0.00001 -0.00105 0.00028

197 3S -0.00175 -0.00010 -0.00007 -0.00161 0.00018

198 4PX -0.00001 0.00000 0.00000 -0.00004 0.00000

199 4PY 0.00000 0.00000 0.00000 -0.00001 0.00000

200 4PZ 0.00000 0.00000 0.00000 0.00000 0.00000

21 22 23 24 25

21 11PZ 0.10084

22 12D 0 0.00000 0.00280

23 12D+1 0.00000 0.00000 0.00331

24 12D-1 0.00000 0.00000 0.00000 0.00493

25 12D+2 0.00000 0.00000 0.00000 0.00000 0.01168

26 12D-2 0.00000 0.00000 0.00000 0.00000 0.00000

27 2 C 1S 0.00001 -0.00004 0.00000 -0.00002 -0.00017

28 2S 0.00001 -0.00007 0.00000 -0.00003 -0.00028

29 2PX 0.00000 0.00000 0.00001 0.00000 0.00004

30 2PY 0.00009 0.00040 0.00000 0.00039 0.00203

31 2PZ 0.00040 0.00011 0.00000 0.00043 0.00016

32 3S -0.00009 0.00133 0.00000 0.00055 0.00551

33 3PX -0.00001 -0.00001 0.00008 0.00000 0.00021

34 3PY 0.00052 0.00130 0.00001 0.00146 0.00649

35 3PZ 0.00311 0.00057 0.00003 0.00320 0.00063

36 4S 0.00022 0.00051 0.00000 0.00021 0.00258

37 4PX 0.00000 0.00000 0.00006 0.00000 0.00012

38 4PY 0.00040 0.00000 0.00001 0.00025 0.00018

39 4PZ 0.00453 0.00026 0.00005 0.00328 0.00021

40 5D 0 0.00013 0.00005 0.00000 0.00004 0.00005

41 5D+1 -0.00001 0.00000 0.00000 0.00001 0.00000

42 5D-1 0.00113 0.00006 -0.00001 0.00015 0.00012

43 5D+2 0.00003 0.00004 0.00000 0.00011 0.00054

44 5D-2 0.00000 0.00001 0.00003 0.00000 -0.00006

45 3 N 1S -0.00001 0.00000 0.00000 0.00000 0.00000

46 2S -0.00002 0.00000 0.00000 0.00000 0.00000

47 2PX -0.00002 0.00000 0.00000 0.00000 0.00000

48 2PY -0.00014 0.00000 0.00000 0.00000 0.00000

49 2PZ -0.00041 0.00000 0.00000 0.00000 0.00000

50 3S 0.00037 -0.00001 0.00000 0.00000 -0.00001

51 3PX -0.00013 -0.00001 0.00000 0.00000 -0.00001

52 3PY -0.00083 0.00000 0.00000 0.00001 0.00004

53 3PZ -0.00270 0.00000 0.00000 0.00000 0.00001

54 4S 0.00125 -0.00004 -0.00001 -0.00002 -0.00010

55 4PX -0.00039 -0.00004 0.00000 0.00002 -0.00005

56 4PY -0.00250 -0.00002 -0.00001 0.00008 0.00016

57 4PZ -0.01184 0.00004 -0.00002 0.00019 0.00004

58 5D 0 -0.00001 0.00000 0.00000 0.00000 0.00000

59 5D+1 0.00001 0.00000 0.00000 0.00000 0.00000

60 5D-1 -0.00007 0.00000 0.00000 0.00000 0.00000

61 5D+2 -0.00001 0.00000 0.00000 0.00000 0.00000

62 5D-2 0.00000 0.00000 0.00000 0.00000 0.00000

63 4 C 1S 0.00002 0.00000 0.00000 0.00000 0.00000

64 2S 0.00003 0.00000 0.00000 0.00000 0.00000

65 2PX -0.00006 0.00000 0.00000 0.00000 0.00000

66 2PY -0.00003 0.00000 0.00000 0.00000 0.00000

67 2PZ -0.00024 0.00000 0.00000 0.00000 0.00000

68 3S -0.00044 0.00000 0.00000 0.00000 0.00000

69 3PX -0.00027 0.00001 0.00000 0.00000 0.00000

70 3PY -0.00014 -0.00001 0.00001 0.00000 0.00000

71 3PZ -0.00153 0.00001 0.00001 0.00000 0.00000

72 4S 0.00036 -0.00003 -0.00004 -0.00008 0.00001

73 4PX -0.00199 -0.00005 -0.00002 0.00001 -0.00002

74 4PY -0.00162 -0.00006 0.00000 -0.00001 -0.00006

75 4PZ -0.00795 0.00002 0.00020 -0.00009 0.00002

76 5D 0 0.00000 0.00000 0.00000 0.00000 0.00000

77 5D+1 -0.00011 0.00000 0.00000 0.00000 0.00000

78 5D-1 0.00005 0.00000 0.00000 0.00000 0.00000

79 5D+2 0.00000 0.00000 0.00000 0.00000 0.00000

80 5D-2 0.00000 0.00000 0.00000 0.00000 0.00000

81 5 C 1S -0.00003 -0.00001 0.00000 0.00000 -0.00003

82 2S -0.00005 -0.00001 -0.00001 0.00000 -0.00005

83 2PX 0.00005 0.00012 0.00013 0.00000 0.00057

84 2PY 0.00001 0.00001 0.00001 0.00000 0.00001

85 2PZ 0.00027 0.00003 0.00009 0.00000 0.00006

86 3S 0.00083 0.00053 0.00026 0.00000 0.00244

87 3PX 0.00032 0.00075 0.00085 0.00000 0.00357

88 3PY 0.00005 0.00004 0.00007 0.00003 0.00013

89 3PZ 0.00184 0.00026 0.00119 -0.00006 0.00039

90 4S 0.00246 0.00046 0.00027 -0.00001 0.00231

91 4PX 0.00092 0.00013 0.00039 0.00000 0.00070

92 4PY 0.00009 0.00000 0.00003 0.00011 0.00012

93 4PZ 0.00805 0.00023 0.00246 -0.00013 0.00026

94 5D 0 0.00006 0.00002 0.00001 0.00001 0.00004

95 5D+1 0.00055 0.00004 0.00000 0.00002 0.00004

96 5D-1 0.00002 0.00000 -0.00001 0.00000 0.00000

97 5D+2 0.00002 0.00007 0.00005 0.00001 0.00015

98 5D-2 0.00000 -0.00001 0.00001 0.00001 0.00021

99 6 C 1S 0.00000 0.00000 0.00000 0.00000 0.00000

100 2S 0.00000 0.00000 0.00000 0.00000 0.00000

101 2PX 0.00000 0.00000 0.00000 0.00000 0.00000

102 2PY 0.00000 0.00000 0.00000 0.00000 0.00000

103 2PZ 0.00001 0.00000 0.00000 0.00000 0.00000

104 3S 0.00001 0.00000 0.00000 0.00000 0.00000

105 3PX -0.00001 0.00000 0.00000 0.00000 0.00000

106 3PY 0.00002 0.00000 0.00000 0.00000 0.00000

107 3PZ 0.00012 0.00000 0.00000 0.00000 0.00000

108 4S -0.00016 0.00000 0.00000 0.00000 0.00000

109 4PX -0.00026 -0.00001 0.00000 0.00000 0.00000

110 4PY 0.00009 0.00000 0.00000 0.00000 0.00000

111 4PZ 0.00064 0.00000 0.00000 0.00000 0.00000

112 5D 0 0.00000 0.00000 0.00000 0.00000 0.00000

113 5D+1 -0.00001 0.00000 0.00000 0.00000 0.00000

114 5D-1 0.00001 0.00000 0.00000 0.00000 0.00000

115 5D+2 0.00000 0.00000 0.00000 0.00000 0.00000

116 5D-2 0.00000 0.00000 0.00000 0.00000 0.00000

117 7 C 1S 0.00000 0.00000 0.00000 0.00000 0.00000

118 2S 0.00000 0.00000 0.00000 0.00000 0.00000

119 2PX 0.00001 0.00000 0.00000 0.00000 0.00000

120 2PY 0.00000 0.00000 0.00000 0.00000 0.00000

121 2PZ 0.00000 0.00000 0.00000 0.00000 0.00000

122 3S 0.00001 0.00000 0.00000 0.00000 0.00000

123 3PX 0.00005 0.00000 0.00000 0.00000 0.00000

124 3PY 0.00001 0.00000 0.00000 0.00000 0.00000

125 3PZ 0.00004 0.00000 0.00000 0.00000 0.00000

126 4S 0.00009 0.00000 0.00000 0.00000 0.00000

127 4PX 0.00031 0.00000 0.00000 0.00000 0.00000

128 4PY 0.00008 0.00000 0.00000 0.00000 0.00000

129 4PZ 0.00042 0.00000 0.00000 0.00000 0.00000

130 5D 0 0.00000 0.00000 0.00000 0.00000 0.00000

131 5D+1 0.00000 0.00000 0.00000 0.00000 0.00000

132 5D-1 0.00000 0.00000 0.00000 0.00000 0.00000

133 5D+2 0.00000 0.00000 0.00000 0.00000 0.00000

134 5D-2 0.00000 0.00000 0.00000 0.00000 0.00000

135 8 C 1S 0.00000 0.00000 0.00000 0.00000 0.00000

136 2S 0.00000 0.00000 0.00000 0.00000 0.00000

137 2PX 0.00000 0.00000 0.00000 0.00000 0.00000

138 2PY 0.00000 0.00000 0.00000 0.00000 0.00000

139 2PZ -0.00001 0.00000 0.00000 0.00000 0.00000

140 3S 0.00000 0.00000 0.00000 0.00000 0.00000

141 3PX -0.00002 0.00000 0.00000 0.00000 0.00000

142 3PY -0.00001 0.00000 0.00000 0.00000 0.00000

143 3PZ -0.00006 0.00000 0.00000 0.00000 0.00000

144 4S -0.00020 0.00000 0.00000 0.00000 0.00001

145 4PX -0.00025 0.00000 0.00000 0.00000 0.00001

146 4PY -0.00003 0.00000 0.00000 0.00000 0.00000

147 4PZ -0.00033 0.00000 0.00000 0.00000 0.00000

148 5D 0 0.00000 0.00000 0.00000 0.00000 0.00000

149 5D+1 -0.00001 0.00000 0.00000 0.00000 0.00000

150 5D-1 0.00000 0.00000 0.00000 0.00000 0.00000

151 5D+2 0.00000 0.00000 0.00000 0.00000 0.00000

152 5D-2 0.00000 0.00000 0.00000 0.00000 0.00000

153 9 C 1S 0.00000 0.00000 0.00000 0.00000 0.00000

154 2S 0.00000 0.00000 0.00000 0.00000 0.00000

155 2PX -0.00003 0.00000 0.00000 0.00000 0.00000

156 2PY 0.00001 0.00000 0.00000 0.00000 0.00000

157 2PZ -0.00013 0.00000 0.00000 0.00000 0.00000

158 3S 0.00007 0.00000 0.00000 0.00000 -0.00001

159 3PX -0.00016 0.00000 0.00000 0.00000 -0.00001

160 3PY 0.00004 0.00000 0.00000 0.00000 0.00000

161 3PZ -0.00085 0.00000 0.00000 0.00000 0.00000

162 4S 0.00024 0.00000 -0.00002 -0.00001 -0.00018

163 4PX -0.00085 -0.00002 0.00002 0.00000 -0.00011

164 4PY 0.00013 0.00000 -0.00001 0.00000 -0.00003

165 4PZ -0.00523 0.00001 0.00007 0.00000 0.00000

166 5D 0 0.00001 0.00000 0.00000 0.00000 0.00000

167 5D+1 0.00004 0.00000 0.00000 0.00000 0.00000

168 5D-1 0.00000 0.00000 0.00000 0.00000 0.00000

169 5D+2 0.00002 0.00000 0.00000 0.00000 0.00000

170 5D-2 0.00001 0.00000 0.00000 0.00000 0.00000

171 10 H 1S -0.00003 0.00001 0.00000 0.00000 -0.00001

172 2S -0.00009 0.00011 0.00000 -0.00001 -0.00018

173 3S -0.00013 0.00001 0.00000 -0.00002 -0.00024

174 4PX 0.00000 0.00000 0.00000 0.00000 0.00001

175 4PY 0.00001 0.00001 0.00000 0.00000 0.00002

176 4PZ 0.00004 0.00000 0.00000 0.00000 0.00000

177 11 H 1S 0.00000 0.00000 0.00000 0.00000 0.00000

178 2S -0.00001 0.00000 0.00000 0.00000 0.00000

179 3S -0.00003 0.00000 0.00000 0.00000 0.00000

180 4PX 0.00000 0.00000 0.00000 0.00000 0.00000

181 4PY 0.00000 0.00000 0.00000 0.00000 0.00000

182 4PZ 0.00000 0.00000 0.00000 0.00000 0.00000

183 12 H 1S 0.00000 0.00000 0.00000 0.00000 0.00000

184 2S -0.00001 0.00000 0.00000 0.00000 0.00000

185 3S -0.00006 0.00000 0.00000 0.00000 0.00000

186 4PX 0.00000 0.00000 0.00000 0.00000 0.00000

187 4PY 0.00000 0.00000 0.00000 0.00000 0.00000

188 4PZ 0.00000 0.00000 0.00000 0.00000 0.00000

189 13 H 1S 0.00000 0.00000 0.00000 0.00000 0.00000

190 2S 0.00000 0.00000 0.00000 0.00000 0.00000

191 3S 0.00003 0.00000 0.00000 0.00000 0.00000

192 4PX 0.00000 0.00000 0.00000 0.00000 0.00000

193 4PY 0.00000 0.00000 0.00000 0.00000 0.00000

194 4PZ 0.00000 0.00000 0.00000 0.00000 0.00000

195 14 H 1S -0.00001 0.00000 0.00000 0.00000 0.00000

196 2S -0.00006 0.00000 0.00000 0.00000 0.00000

197 3S -0.00014 -0.00002 0.00000 0.00000 -0.00001

198 4PX 0.00000 0.00000 0.00000 0.00000 0.00000

199 4PY 0.00000 0.00000 0.00000 0.00000 0.00000

200 4PZ -0.00002 0.00000 0.00000 0.00000 0.00000

26 27 28 29 30

26 12D-2 0.00361

27 2 C 1S 0.00000 0.65899

28 2S 0.00000 0.49311 0.49150

29 2PX 0.00048 0.00000 0.00000 0.09369

30 2PY 0.00000 0.00000 0.00000 0.00000 0.08339

31 2PZ 0.00000 0.00000 0.00000 0.00000 0.00000

32 3S 0.00005 -0.02855 -0.06327 0.00000 0.00000

33 3PX 0.00292 0.00000 0.00000 0.08429 0.00000

34 3PY -0.00003 0.00000 0.00000 0.00000 0.07780

35 3PZ 0.00000 0.00000 0.00000 0.00000 0.00000

36 4S 0.00003 -0.00977 -0.01825 0.00000 0.00000

37 4PX 0.00196 0.00000 0.00000 0.01870 0.00000

38 4PY 0.00001 0.00000 0.00000 0.00000 0.00968

39 4PZ 0.00000 0.00000 0.00000 0.00000 0.00000

40 5D 0 0.00000 0.00000 0.00000 0.00000 0.00000

41 5D+1 0.00002 0.00000 0.00000 0.00000 0.00000

42 5D-1 0.00000 0.00000 0.00000 0.00000 0.00000

43 5D+2 -0.00001 0.00000 0.00000 0.00000 0.00000

44 5D-2 0.00016 0.00000 0.00000 0.00000 0.00000

45 3 N 1S 0.00000 0.00000 0.00000 -0.00003 -0.00003

46 2S 0.00000 0.00000 0.00000 -0.00003 -0.00003

47 2PX 0.00000 -0.00003 -0.00001 0.00067 0.00088

48 2PY 0.00000 -0.00003 -0.00001 0.00102 0.00038

49 2PZ 0.00000 0.00000 0.00000 0.00017 0.00016

50 3S 0.00000 -0.00027 -0.00039 0.00401 0.00338

51 3PX 0.00001 -0.00100 -0.00169 0.00432 0.00712

52 3PY 0.00002 -0.00103 -0.00179 0.00839 0.00211

53 3PZ 0.00000 -0.00011 -0.00018 0.00147 0.00129

54 4S -0.00002 -0.00085 -0.00079 0.00120 0.00120

55 4PX -0.00003 -0.00120 -0.00212 -0.00002 0.00301

56 4PY 0.00004 -0.00218 -0.00395 0.00386 -0.00026

57 4PZ 0.00002 -0.00016 -0.00028 0.00069 0.00074

58 5D 0 0.00000 -0.00007 -0.00010 0.00015 0.00009

59 5D+1 0.00000 -0.00002 -0.00004 0.00008 0.00018

60 5D-1 0.00000 -0.00003 -0.00005 0.00031 0.00010

61 5D+2 0.00000 0.00000 0.00000 0.00044 0.00020

62 5D-2 0.00000 -0.00024 -0.00038 0.00106 0.00065

63 4 C 1S 0.00000 0.00000 0.00000 0.00000 0.00000

64 2S 0.00000 0.00000 0.00000 0.00000 0.00000

65 2PX 0.00000 0.00000 0.00000 0.00000 0.00000

66 2PY 0.00000 0.00000 0.00000 0.00000 0.00000

67 2PZ 0.00000 0.00000 0.00000 0.00000 0.00000

68 3S 0.00000 0.00000 0.00000 -0.00008 0.00000

69 3PX 0.00000 0.00001 0.00001 -0.00018 0.00000

70 3PY 0.00007 0.00000 0.00000 0.00000 -0.00001

71 3PZ 0.00002 0.00000 0.00000 -0.00001 0.00000

72 4S -0.00037 0.00020 0.00028 -0.00200 0.00003

73 4PX -0.00032 0.00077 0.00117 -0.00260 -0.00003

74 4PY 0.00006 0.00000 0.00000 0.00002 -0.00025

75 4PZ 0.00006 0.00001 0.00002 -0.00010 0.00000

76 5D 0 0.00000 0.00000 0.00000 0.00001 0.00000

77 5D+1 0.00000 0.00000 0.00000 0.00001 0.00000

78 5D-1 0.00000 0.00000 0.00000 0.00000 0.00000

79 5D+2 0.00000 0.00000 0.00000 0.00010 0.00000

80 5D-2 0.00001 0.00000 0.00000 0.00000 0.00000

81 5 C 1S 0.00000 0.00000 0.00000 0.00000 0.00000

82 2S 0.00000 0.00000 0.00000 0.00000 0.00000

83 2PX 0.00009 0.00000 0.00000 0.00000 0.00000

84 2PY 0.00005 0.00000 0.00000 0.00000 0.00000

85 2PZ 0.00001 0.00000 0.00000 0.00000 0.00000

86 3S 0.00020 0.00000 0.00000 -0.00003 -0.00001

87 3PX 0.00058 0.00000 0.00000 -0.00006 -0.00003

88 3PY 0.00057 0.00000 0.00000 -0.00004 0.00000

89 3PZ 0.00005 0.00000 0.00000 0.00000 0.00000

90 4S 0.00016 -0.00001 0.00006 -0.00084 -0.00027

91 4PX 0.00024 0.00002 0.00001 -0.00004 -0.00063

92 4PY 0.00148 0.00006 0.00014 0.00088 0.00014

93 4PZ 0.00003 0.00000 0.00000 -0.00003 0.00001

94 5D 0 0.00001 0.00000 0.00000 0.00000 0.00000

95 5D+1 0.00001 0.00000 0.00000 0.00000 0.00000

96 5D-1 0.00000 0.00000 0.00000 0.00000 0.00000

97 5D+2 0.00004 0.00000 0.00000 0.00000 0.00001

98 5D-2 -0.00001 0.00000 0.00000 0.00002 0.00002

99 6 C 1S 0.00000 0.00000 0.00000 0.00000 0.00000

100 2S 0.00000 0.00000 0.00000 0.00000 0.00000

101 2PX 0.00000 0.00000 0.00000 0.00000 0.00000

102 2PY 0.00000 0.00000 0.00000 0.00000 0.00000

103 2PZ 0.00000 0.00000 0.00000 0.00000 0.00000

104 3S 0.00000 0.00000 0.00000 0.00000 0.00000

105 3PX 0.00000 0.00000 0.00000 0.00000 0.00000

106 3PY 0.00000 0.00000 0.00000 0.00000 0.00000

107 3PZ 0.00000 0.00000 0.00000 0.00000 0.00000

108 4S 0.00000 0.00000 0.00000 0.00002 0.00000

109 4PX -0.00001 0.00000 0.00000 0.00001 -0.00001

110 4PY 0.00000 0.00000 0.00000 0.00000 0.00000

111 4PZ 0.00000 0.00000 0.00000 0.00000 0.00000

112 5D 0 0.00000 0.00000 0.00000 0.00000 0.00000

113 5D+1 0.00000 0.00000 0.00000 0.00000 0.00000

114 5D-1 0.00000 0.00000 0.00000 0.00000 0.00000

115 5D+2 0.00000 0.00000 0.00000 0.00000 0.00000

116 5D-2 0.00000 0.00000 0.00000 0.00000 0.00000

117 7 C 1S 0.00000 0.00000 0.00000 0.00000 0.00000

118 2S 0.00000 0.00000 0.00000 0.00000 0.00000

119 2PX 0.00000 0.00000 0.00000 0.00000 0.00000

120 2PY 0.00000 0.00000 0.00000 0.00000 0.00000

121 2PZ 0.00000 0.00000 0.00000 0.00000 0.00000

122 3S 0.00000 0.00000 0.00000 0.00000 0.00000

123 3PX 0.00000 0.00000 0.00000 0.00000 0.00000

124 3PY 0.00000 0.00000 0.00000 0.00000 0.00000

125 3PZ 0.00000 0.00000 0.00000 0.00000 0.00000

126 4S 0.00000 0.00000 0.00000 0.00000 0.00000

127 4PX 0.00000 0.00000 0.00000 0.00000 0.00000

128 4PY 0.00000 0.00000 0.00000 0.00000 0.00000

129 4PZ 0.00000 0.00000 0.00000 0.00000 0.00000

130 5D 0 0.00000 0.00000 0.00000 0.00000 0.00000

131 5D+1 0.00000 0.00000 0.00000 0.00000 0.00000

132 5D-1 0.00000 0.00000 0.00000 0.00000 0.00000

133 5D+2 0.00000 0.00000 0.00000 0.00000 0.00000

134 5D-2 0.00000 0.00000 0.00000 0.00000 0.00000

135 8 C 1S 0.00000 0.00000 0.00000 0.00000 0.00000

136 2S 0.00000 0.00000 0.00000 0.00000 0.00000

137 2PX 0.00000 0.00000 0.00000 0.00000 0.00000

138 2PY 0.00000 0.00000 0.00000 0.00000 0.00000

139 2PZ 0.00000 0.00000 0.00000 0.00000 0.00000

140 3S 0.00000 0.00000 0.00000 0.00000 0.00000

141 3PX 0.00000 0.00000 0.00000 0.00000 0.00000

142 3PY 0.00000 0.00000 0.00000 0.00000 0.00000

143 3PZ 0.00000 0.00000 0.00000 0.00000 0.00000

144 4S 0.00000 0.00000 0.00000 0.00000 0.00000

145 4PX 0.00000 0.00000 0.00000 0.00000 0.00000

146 4PY 0.00000 0.00000 0.00000 0.00000 0.00000

147 4PZ 0.00000 0.00000 0.00000 0.00000 0.00000

148 5D 0 0.00000 0.00000 0.00000 0.00000 0.00000

149 5D+1 0.00000 0.00000 0.00000 0.00000 0.00000

150 5D-1 0.00000 0.00000 0.00000 0.00000 0.00000

151 5D+2 0.00000 0.00000 0.00000 0.00000 0.00000

152 5D-2 0.00000 0.00000 0.00000 0.00000 0.00000

153 9 C 1S 0.00000 0.00000 0.00000 0.00000 0.00000

154 2S 0.00000 0.00000 0.00000 0.00000 0.00000

155 2PX 0.00000 0.00000 0.00000 0.00000 0.00000

156 2PY 0.00000 0.00000 0.00000 0.00000 0.00000

157 2PZ 0.00000 0.00000 0.00000 0.00000 0.00000

158 3S 0.00000 0.00000 0.00000 0.00000 0.00000

159 3PX 0.00001 0.00000 0.00000 0.00000 0.00000

160 3PY 0.00000 0.00000 0.00000 0.00000 0.00000

161 3PZ 0.00000 0.00000 0.00000 0.00000 0.00000

162 4S -0.00002 0.00000 0.00000 0.00002 0.00001

163 4PX -0.00002 0.00000 0.00000 0.00004 0.00001

164 4PY -0.00003 0.00000 0.00000 0.00001 0.00000

165 4PZ 0.00000 0.00000 0.00000 0.00000 0.00000

166 5D 0 0.00000 0.00000 0.00000 0.00000 0.00000

167 5D+1 0.00000 0.00000 0.00000 0.00000 0.00000

168 5D-1 0.00000 0.00000 0.00000 0.00000 0.00000

169 5D+2 0.00000 0.00000 0.00000 0.00000 0.00000

170 5D-2 0.00000 0.00000 0.00000 0.00000 0.00000

171 10 H 1S 0.00003 -0.00021 -0.00030 0.00654 0.00110

172 2S 0.00050 -0.00232 -0.00402 0.01474 0.00258

173 3S 0.00021 -0.00016 -0.00034 0.00245 0.00063

174 4PX 0.00001 -0.00050 -0.00077 0.00135 0.00034

175 4PY 0.00002 -0.00009 -0.00014 0.00060 0.00010

176 4PZ 0.00000 0.00000 0.00000 0.00002 0.00000

177 11 H 1S 0.00000 0.00000 0.00000 0.00000 0.00000

178 2S 0.00000 0.00000 0.00000 0.00000 0.00000

179 3S 0.00000 0.00000 0.00000 -0.00001 0.00000

180 4PX 0.00000 0.00000 0.00000 0.00000 0.00000

181 4PY 0.00000 0.00000 0.00000 0.00000 0.00000

182 4PZ 0.00000 0.00000 0.00000 0.00000 0.00000

183 12 H 1S 0.00000 0.00000 0.00000 0.00000 0.00000

184 2S 0.00000 0.00000 0.00000 0.00000 0.00000

185 3S 0.00000 0.00000 0.00000 0.00000 0.00000

186 4PX 0.00000 0.00000 0.00000 0.00000 0.00000

187 4PY 0.00000 0.00000 0.00000 0.00000 0.00000

188 4PZ 0.00000 0.00000 0.00000 0.00000 0.00000

189 13 H 1S 0.00000 0.00000 0.00000 0.00000 0.00000

190 2S 0.00000 0.00000 0.00000 0.00000 0.00000

191 3S 0.00000 0.00000 0.00000 0.00000 0.00000

192 4PX 0.00000 0.00000 0.00000 0.00000 0.00000

193 4PY 0.00000 0.00000 0.00000 0.00000 0.00000

194 4PZ 0.00000 0.00000 0.00000 0.00000 0.00000

195 14 H 1S 0.00000 0.00000 0.00000 0.00000 0.00000

196 2S 0.00000 0.00000 0.00000 0.00000 0.00000

197 3S -0.00001 0.00000 0.00000 0.00000 0.00000

198 4PX 0.00000 0.00000 0.00000 0.00000 0.00000

199 4PY 0.00000 0.00000 0.00000 0.00000 0.00000

200 4PZ 0.00000 0.00000 0.00000 0.00000 0.00000

31 32 33 34 35

31 2PZ 0.07345

32 3S 0.00000 0.45920

33 3PX 0.00000 0.00000 0.19224

34 3PY 0.00000 0.00000 0.00000 0.18549

35 3PZ 0.07166 0.00000 0.00000 0.00000 0.17697

36 4S 0.00000 0.20259 0.00000 0.00000 0.00000

37 4PX 0.00000 0.00000 0.07926 0.00000 0.00000

38 4PY 0.00000 0.00000 0.00000 0.04429 0.00000

39 4PZ 0.02215 0.00000 0.00000 0.00000 0.10219

40 5D 0 0.00000 0.00000 0.00000 0.00000 0.00000

41 5D+1 0.00000 0.00000 0.00000 0.00000 0.00000

42 5D-1 0.00000 0.00000 0.00000 0.00000 0.00000

43 5D+2 0.00000 0.00000 0.00000 0.00000 0.00000

44 5D-2 0.00000 0.00000 0.00000 0.00000 0.00000

45 3 N 1S 0.00000 -0.00035 -0.00089 -0.00076 -0.00009

46 2S 0.00000 -0.00055 -0.00146 -0.00126 -0.00015

47 2PX 0.00018 0.00438 0.00280 0.00534 0.00114

48 2PY 0.00016 0.00490 0.00708 0.00140 0.00108

49 2PZ 0.00013 0.00047 0.00118 0.00101 0.00329

50 3S 0.00039 0.00955 0.01870 0.01602 0.00184

51 3PX 0.00147 0.02438 0.00751 0.02096 0.00467

52 3PY 0.00134 0.02686 0.02813 0.00296 0.00436

53 3PZ 0.00317 0.00263 0.00483 0.00405 0.02119

54 4S 0.00010 -0.00520 0.00333 0.00447 0.00032

55 4PX 0.00070 0.02014 -0.00216 0.01032 0.00256

56 4PY 0.00075 0.03863 0.01538 -0.00341 0.00280

57 4PZ 0.00639 0.00262 0.00265 0.00268 0.03188

58 5D 0 0.00027 0.00069 0.00016 0.00007 0.00076

59 5D+1 0.00018 0.00026 0.00010 0.00035 0.00089

60 5D-1 0.00029 0.00035 0.00063 0.00011 0.00140

61 5D+2 0.00000 -0.00005 0.00174 0.00083 -0.00001

62 5D-2 0.00028 0.00298 0.00123 0.00062 0.00054

63 4 C 1S 0.00000 0.00000 0.00001 0.00000 0.00000

64 2S 0.00000 0.00000 0.00001 0.00000 0.00000

65 2PX 0.00000 -0.00008 -0.00018 0.00000 0.00000

66 2PY 0.00000 0.00000 0.00000 -0.00002 0.00000

67 2PZ 0.00000 0.00000 0.00000 0.00000 -0.00002

68 3S 0.00000 -0.00060 -0.00127 0.00001 -0.00006

69 3PX -0.00001 -0.00130 -0.00171 -0.00004 -0.00008

70 3PY 0.00000 -0.00002 0.00002 -0.00029 0.00000

71 3PZ -0.00002 -0.00004 -0.00007 0.00000 -0.00044

72 4S -0.00018 -0.00362 -0.01147 0.00021 -0.00109

73 4PX 0.00012 -0.01355 -0.01225 -0.00007 0.00074

74 4PY 0.00000 0.00002 0.00009 -0.00163 0.00002

75 4PZ -0.00037 -0.00026 -0.00048 0.00000 -0.00257

76 5D 0 0.00000 0.00002 0.00011 0.00000 -0.00001

77 5D+1 0.00001 0.00001 0.00013 0.00000 0.00023

78 5D-1 0.00000 0.00000 0.00000 0.00001 -0.00001

79 5D+2 0.00001 0.00051 0.00110 0.00001 0.00010

80 5D-2 0.00000 0.00002 0.00002 -0.00006 0.00000

81 5 C 1S 0.00000 0.00000 0.00000 0.00000 0.00000

82 2S 0.00000 0.00000 0.00000 0.00000 0.00000

83 2PX 0.00000 -0.00001 -0.00004 -0.00003 0.00000

84 2PY 0.00000 -0.00002 -0.00003 0.00000 0.00000

85 2PZ 0.00000 0.00000 0.00000 0.00000 -0.00002

86 3S 0.00000 -0.00039 -0.00076 -0.00020 -0.00001

87 3PX 0.00000 -0.00036 -0.00081 -0.00048 -0.00001

88 3PY 0.00000 -0.00051 -0.00054 -0.00004 0.00002

89 3PZ -0.00002 0.00000 -0.00002 0.00001 -0.00054

90 4S -0.00001 -0.00311 -0.00565 -0.00168 -0.00006

91 4PX 0.00000 0.00037 -0.00005 -0.00322 -0.00002

92 4PY 0.00002 -0.00299 0.00465 0.00052 0.00012

93 4PZ -0.00075 -0.00004 -0.00014 0.00004 -0.00500

94 5D 0 0.00000 0.00003 0.00006 0.00005 0.00000

95 5D+1 0.00000 0.00000 0.00000 0.00000 0.00005

96 5D-1 0.00000 0.00000 0.00000 0.00000 -0.00001

97 5D+2 0.00000 0.00003 0.00001 0.00013 0.00000

98 5D-2 0.00000 0.00018 0.00028 0.00030 0.00000

99 6 C 1S 0.00000 0.00000 0.00000 0.00000 0.00000

100 2S 0.00000 0.00000 0.00000 0.00000 0.00000

101 2PX 0.00000 0.00000 0.00000 0.00000 0.00000

102 2PY 0.00000 0.00000 0.00000 0.00000 0.00000

103 2PZ 0.00000 0.00000 0.00000 0.00000 0.00000

104 3S 0.00000 0.00000 0.00000 0.00000 0.00000

105 3PX 0.00000 0.00000 0.00000 0.00000 0.00000

106 3PY 0.00000 0.00000 0.00000 0.00000 0.00000

107 3PZ 0.00000 0.00000 0.00000 0.00000 0.00000

108 4S 0.00000 0.00007 0.00023 0.00001 0.00000

109 4PX 0.00000 0.00020 -0.00001 -0.00005 -0.00002

110 4PY 0.00000 -0.00004 -0.00001 0.00002 0.00000

111 4PZ -0.00001 0.00000 0.00001 0.00000 -0.00007

112 5D 0 0.00000 0.00000 0.00000 0.00000 0.00000

113 5D+1 0.00000 0.00000 0.00000 0.00000 0.00000

114 5D-1 0.00000 0.00000 0.00000 0.00000 0.00000

115 5D+2 0.00000 0.00000 0.00000 0.00000 0.00000

116 5D-2 0.00000 0.00000 0.00000 0.00000 0.00000

117 7 C 1S 0.00000 0.00000 0.00000 0.00000 0.00000

118 2S 0.00000 0.00000 0.00000 0.00000 0.00000

119 2PX 0.00000 0.00000 0.00000 0.00000 0.00000

120 2PY 0.00000 0.00000 0.00000 0.00000 0.00000

121 2PZ 0.00000 0.00000 0.00000 0.00000 0.00000

122 3S 0.00000 0.00000 0.00000 0.00000 0.00000

123 3PX 0.00000 0.00000 0.00000 0.00000 0.00000

124 3PY 0.00000 0.00000 0.00000 0.00000 0.00000

125 3PZ 0.00000 0.00000 0.00000 0.00000 0.00000

126 4S 0.00000 0.00000 -0.00001 0.00000 0.00000

127 4PX 0.00000 0.00001 0.00001 0.00000 0.00001

128 4PY 0.00000 0.00000 0.00000 0.00000 0.00000

129 4PZ 0.00000 0.00000 -0.00001 0.00000 0.00000

130 5D 0 0.00000 0.00000 0.00000 0.00000 0.00000

131 5D+1 0.00000 0.00000 0.00000 0.00000 0.00000

132 5D-1 0.00000 0.00000 0.00000 0.00000 0.00000

133 5D+2 0.00000 0.00000 0.00000 0.00000 0.00000

134 5D-2 0.00000 0.00000 0.00000 0.00000 0.00000

135 8 C 1S 0.00000 0.00000 0.00000 0.00000 0.00000

136 2S 0.00000 0.00000 0.00000 0.00000 0.00000

137 2PX 0.00000 0.00000 0.00000 0.00000 0.00000

138 2PY 0.00000 0.00000 0.00000 0.00000 0.00000

139 2PZ 0.00000 0.00000 0.00000 0.00000 0.00000

140 3S 0.00000 0.00000 0.00000 0.00000 0.00000

141 3PX 0.00000 0.00000 0.00000 0.00000 0.00000

142 3PY 0.00000 0.00000 0.00000 0.00000 0.00000

143 3PZ 0.00000 0.00000 0.00000 0.00000 0.00000

144 4S 0.00000 0.00000 0.00000 0.00000 0.00000

145 4PX 0.00000 -0.00001 -0.00001 0.00000 0.00000

146 4PY 0.00000 0.00000 -0.00001 0.00000 0.00000

147 4PZ 0.00000 0.00000 0.00000 0.00000 0.00000

148 5D 0 0.00000 0.00000 0.00000 0.00000 0.00000

149 5D+1 0.00000 0.00000 0.00000 0.00000 0.00000

150 5D-1 0.00000 0.00000 0.00000 0.00000 0.00000

151 5D+2 0.00000 0.00000 0.00000 0.00000 0.00000

152 5D-2 0.00000 0.00000 0.00000 0.00000 0.00000

153 9 C 1S 0.00000 0.00000 0.00000 0.00000 0.00000

154 2S 0.00000 0.00000 0.00000 0.00000 0.00000

155 2PX 0.00000 0.00000 0.00000 0.00000 0.00000

156 2PY 0.00000 0.00000 0.00000 0.00000 0.00000

157 2PZ 0.00000 0.00000 0.00000 0.00000 0.00000

158 3S 0.00000 0.00000 0.00000 0.00000 0.00000

159 3PX 0.00000 0.00000 0.00000 0.00000 0.00000

160 3PY 0.00000 0.00000 0.00000 0.00000 0.00000

161 3PZ 0.00000 0.00000 0.00000 0.00000 0.00000

162 4S 0.00000 0.00007 0.00025 0.00013 0.00000

163 4PX 0.00000 0.00001 0.00041 0.00007 0.00000

164 4PY 0.00000 0.00006 0.00013 0.00005 0.00000

165 4PZ 0.00000 0.00000 0.00000 0.00000 0.00001

166 5D 0 0.00000 0.00000 0.00000 0.00000 0.00000

167 5D+1 0.00000 0.00000 0.00000 0.00000 0.00000

168 5D-1 0.00000 0.00000 0.00000 0.00000 0.00000

169 5D+2 0.00000 0.00000 0.00000 0.00000 0.00000

170 5D-2 0.00000 0.00000 0.00000 0.00000 0.00000

171 10 H 1S 0.00004 0.01478 0.03486 0.00606 0.00018

172 2S 0.00008 0.04660 0.05909 0.01074 0.00033

173 3S 0.00000 0.00451 0.01230 0.00345 0.00001

174 4PX 0.00003 0.00574 0.00244 0.00073 0.00008

175 4PY 0.00000 0.00109 0.00143 0.00080 0.00001

176 4PZ 0.00042 0.00003 0.00005 0.00000 0.00194

177 11 H 1S 0.00000 0.00000 0.00000 0.00000 0.00000

178 2S 0.00000 0.00000 0.00000 0.00000 0.00000

179 3S 0.00000 -0.00009 -0.00006 -0.00002 0.00000

180 4PX 0.00000 0.00000 0.00000 0.00000 0.00000

181 4PY 0.00000 0.00000 0.00000 0.00000 0.00000

182 4PZ 0.00000 0.00000 0.00000 0.00000 0.00000

183 12 H 1S 0.00000 0.00000 0.00000 0.00000 0.00000

184 2S 0.00000 0.00000 0.00000 0.00000 0.00000

185 3S 0.00000 0.00000 0.00000 0.00000 0.00000

186 4PX 0.00000 0.00000 0.00000 0.00000 0.00000

187 4PY 0.00000 0.00000 0.00000 0.00000 0.00000

188 4PZ 0.00000 0.00000 0.00000 0.00000 0.00000

189 13 H 1S 0.00000 0.00000 0.00000 0.00000 0.00000

190 2S 0.00000 0.00000 0.00000 0.00000 0.00000

191 3S 0.00000 0.00000 0.00000 0.00000 0.00000

192 4PX 0.00000 0.00000 0.00000 0.00000 0.00000

193 4PY 0.00000 0.00000 0.00000 0.00000 0.00000

194 4PZ 0.00000 0.00000 0.00000 0.00000 0.00000

195 14 H 1S 0.00000 0.00000 0.00000 0.00000 0.00000

196 2S 0.00000 0.00000 0.00000 0.00000 0.00000

197 3S 0.00000 -0.00003 -0.00001 0.00000 0.00000

198 4PX 0.00000 0.00000 0.00000 0.00000 0.00000

199 4PY 0.00000 0.00000 0.00000 0.00000 0.00000

200 4PZ 0.00000 0.00000 0.00000 0.00000 0.00000

36 37 38 39 40

36 4S 0.18842

37 4PX 0.00000 0.09622

38 4PY 0.00000 0.00000 0.04552

39 4PZ 0.00000 0.00000 0.00000 0.14699

40 5D 0 0.00000 0.00000 0.00000 0.00000 0.00105

41 5D+1 0.00000 0.00000 0.00000 0.00000 0.00000

42 5D-1 0.00000 0.00000 0.00000 0.00000 0.00000

43 5D+2 0.00000 0.00000 0.00000 0.00000 0.00000

44 5D-2 0.00000 0.00000 0.00000 0.00000 0.00000

45 3 N 1S 0.00081 -0.00022 -0.00043 -0.00003 -0.00007

46 2S 0.00123 -0.00058 -0.00089 -0.00008 -0.00010

47 2PX 0.00175 -0.00073 0.00117 0.00042 -0.00001

48 2PY 0.00123 0.00023 0.00059 0.00016 0.00007

49 2PZ 0.00016 0.00038 0.00018 0.00505 0.00036

50 3S -0.01619 0.01047 0.01320 0.00128 0.00076

51 3PX 0.00961 -0.00559 0.00563 0.00206 -0.00002

52 3PY 0.00650 0.00121 0.00417 0.00083 0.00013

53 3PZ 0.00088 0.00192 0.00090 0.02906 0.00127

54 4S -0.03653 0.00323 0.01444 0.00099 0.00025

55 4PX 0.01047 -0.01057 0.00549 0.00215 0.00003

56 4PY 0.01230 -0.00411 0.01300 0.00047 -0.00003

57 4PZ 0.00100 0.00176 0.00090 0.05337 0.00075

58 5D 0 0.00005 0.00003 0.00004 0.00021 0.00001

59 5D+1 0.00003 -0.00001 0.00004 0.00059 0.00000

60 5D-1 0.00005 0.00008 -0.00001 0.00076 0.00009

61 5D+2 -0.00001 0.00051 0.00036 0.00000 0.00000

62 5D-2 0.00046 -0.00021 -0.00006 0.00009 0.00000

63 4 C 1S 0.00017 0.00065 0.00000 0.00003 0.00000

64 2S 0.00029 0.00111 0.00000 0.00005 0.00000

65 2PX -0.00112 -0.00251 -0.00005 -0.00010 0.00001

66 2PY 0.00000 0.00005 -0.00036 0.00000 0.00000

67 2PZ -0.00003 -0.00008 0.00000 -0.00068 0.00001

68 3S -0.00485 -0.01475 -0.00004 -0.00075 0.00003

69 3PX -0.00688 -0.01241 -0.00028 -0.00061 0.00014

70 3PY -0.00002 0.00026 -0.00231 0.00001 0.00000

71 3PZ -0.00019 -0.00045 -0.00002 -0.00456 0.00008

72 4S -0.00816 -0.03725 -0.00009 -0.00370 0.00004

73 4PX -0.01374 -0.01583 -0.00020 0.00116 -0.00008

74 4PY -0.00004 0.00003 -0.00051 0.00001 0.00000

75 4PZ -0.00060 -0.00156 -0.00005 -0.01200 0.00016

76 5D 0 -0.00003 -0.00002 0.00000 -0.00005 0.00000

77 5D+1 -0.00001 0.00006 0.00000 0.00070 0.00002

78 5D-1 0.00000 0.00000 0.00001 -0.00002 0.00000

79 5D+2 0.00032 0.00024 -0.00003 0.00005 0.00000

80 5D-2 0.00002 0.00003 -0.00022 0.00000 0.00000

81 5 C 1S 0.00009 0.00021 0.00012 0.00000 0.00000

82 2S 0.00016 0.00036 0.00023 0.00000 0.00000

83 2PX -0.00042 -0.00035 -0.00071 0.00000 0.00000

84 2PY -0.00029 -0.00009 -0.00009 0.00002 0.00000

85 2PZ 0.00000 -0.00002 0.00001 -0.00080 0.00000

86 3S -0.00302 -0.00507 -0.00356 -0.00002 0.00004

87 3PX -0.00307 -0.00212 -0.00430 -0.00003 0.00005

88 3PY -0.00173 -0.00051 -0.00030 0.00011 0.00005

89 3PZ -0.00002 -0.00011 0.00007 -0.00532 0.00000

90 4S -0.00487 -0.00728 -0.01045 -0.00012 0.00006

91 4PX -0.00471 -0.00381 -0.00339 -0.00013 0.00007

92 4PY -0.00439 0.00862 0.00016 0.00034 0.00004

93 4PZ -0.00009 -0.00037 0.00018 -0.02010 0.00000

94 5D 0 0.00000 0.00000 0.00002 0.00001 0.00001

95 5D+1 0.00000 0.00000 -0.00001 0.00011 0.00000

96 5D-1 0.00000 0.00000 0.00000 -0.00010 0.00000

97 5D+2 0.00003 -0.00004 -0.00010 0.00000 0.00000

98 5D-2 0.00037 0.00014 0.00018 0.00000 0.00001

99 6 C 1S 0.00000 0.00000 0.00000 0.00000 0.00000

100 2S 0.00000 -0.00001 0.00000 0.00000 0.00000

101 2PX 0.00002 0.00003 0.00000 0.00000 0.00000

102 2PY 0.00000 0.00001 0.00000 0.00000 0.00000

103 2PZ 0.00000 0.00000 0.00000 -0.00001 0.00000

104 3S 0.00016 0.00036 0.00002 0.00000 0.00000

105 3PX 0.00025 0.00029 0.00002 -0.00002 0.00000

106 3PY 0.00002 0.00009 -0.00001 0.00000 0.00000

107 3PZ 0.00000 0.00001 0.00000 -0.00008 0.00000

108 4S 0.00070 0.00204 0.00011 -0.00002 -0.00002

109 4PX 0.00115 0.00107 0.00010 -0.00010 -0.00004

110 4PY 0.00002 0.00049 -0.00014 0.00000 0.00000

111 4PZ 0.00002 0.00002 0.00000 -0.00052 0.00000

112 5D 0 0.00000 -0.00001 0.00000 0.00000 0.00000

113 5D+1 0.00000 0.00000 0.00000 -0.00003 0.00000

114 5D-1 0.00000 0.00000 0.00000 0.00000 0.00000

115 5D+2 0.00000 -0.00006 -0.00002 0.00000 0.00000

116 5D-2 0.00000 0.00002 -0.00001 0.00000 0.00000

117 7 C 1S 0.00000 0.00000 0.00000 0.00000 0.00000

118 2S 0.00000 0.00000 0.00000 0.00000 0.00000

119 2PX 0.00000 0.00000 0.00000 0.00000 0.00000

120 2PY 0.00000 0.00000 0.00000 0.00000 0.00000

121 2PZ 0.00000 0.00000 0.00000 0.00000 0.00000

122 3S 0.00000 -0.00001 0.00000 0.00000 0.00000

123 3PX 0.00000 0.00001 0.00000 0.00001 0.00000

124 3PY 0.00000 0.00000 0.00000 0.00000 0.00000

125 3PZ 0.00000 -0.00001 0.00000 0.00000 0.00000

126 4S -0.00002 -0.00030 0.00000 0.00001 0.00000

127 4PX 0.00004 0.00011 0.00000 0.00018 0.00000

128 4PY 0.00000 -0.00001 -0.00003 0.00000 0.00000

129 4PZ -0.00002 -0.00011 0.00000 0.00007 0.00000

130 5D 0 0.00000 0.00000 0.00000 0.00000 0.00000

131 5D+1 0.00000 0.00000 0.00000 0.00000 0.00000

132 5D-1 0.00000 0.00000 0.00000 0.00000 0.00000

133 5D+2 0.00000 0.00000 0.00000 0.00000 0.00000

134 5D-2 0.00000 0.00000 0.00000 0.00000 0.00000

135 8 C 1S 0.00000 0.00000 0.00000 0.00000 0.00000

136 2S 0.00000 0.00000 0.00000 0.00000 0.00000

137 2PX 0.00000 0.00000 0.00000 0.00000 0.00000

138 2PY 0.00000 0.00000 0.00000 0.00000 0.00000

139 2PZ 0.00000 0.00000 0.00000 0.00000 0.00000

140 3S 0.00000 0.00000 0.00000 0.00000 0.00000

141 3PX 0.00000 -0.00001 0.00000 0.00000 0.00000

142 3PY 0.00000 -0.00001 0.00000 0.00000 0.00000

143 3PZ 0.00000 0.00000 0.00000 0.00000 0.00000

144 4S -0.00006 -0.00005 -0.00005 0.00000 0.00000

145 4PX -0.00010 -0.00011 -0.00008 0.00006 0.00000

146 4PY -0.00002 -0.00017 0.00002 0.00004 0.00000

147 4PZ 0.00000 0.00003 -0.00002 0.00018 0.00000

148 5D 0 0.00000 0.00000 0.00000 0.00000 0.00000

149 5D+1 0.00000 0.00000 0.00000 0.00000 0.00000

150 5D-1 0.00000 0.00000 0.00000 0.00000 0.00000

151 5D+2 0.00000 0.00000 0.00000 0.00000 0.00000

152 5D-2 0.00000 0.00000 0.00000 0.00000 0.00000

153 9 C 1S 0.00000 0.00000 0.00000 0.00000 0.00000

154 2S 0.00000 0.00000 0.00000 0.00000 0.00000

155 2PX 0.00001 0.00000 0.00002 0.00000 0.00000

156 2PY 0.00000 0.00000 0.00001 0.00000 0.00000

157 2PZ 0.00000 0.00000 0.00000 0.00000 0.00000

158 3S 0.00008 0.00003 0.00016 0.00000 0.00000

159 3PX 0.00011 0.00000 0.00023 0.00000 0.00000

160 3PY 0.00002 -0.00001 0.00006 0.00000 0.00000

161 3PZ 0.00000 0.00000 0.00000 0.00001 0.00000

162 4S 0.00045 0.00082 0.00149 0.00002 0.00000

163 4PX 0.00018 0.00111 0.00094 0.00002 0.00000

164 4PY 0.00015 -0.00001 0.00046 -0.00001 0.00000

165 4PZ 0.00000 0.00002 0.00000 0.00036 0.00000

166 5D 0 0.00000 0.00000 0.00000 0.00000 0.00000

167 5D+1 0.00000 0.00000 0.00000 -0.00002 0.00000

168 5D-1 0.00000 0.00000 0.00000 0.00000 0.00000

169 5D+2 0.00000 0.00000 -0.00001 0.00000 0.00000

170 5D-2 0.00000 0.00000 0.00000 0.00000 0.00000

171 10 H 1S 0.01250 0.02638 0.00051 0.00026 0.00048

172 2S 0.03678 0.06125 0.00039 0.00066 0.00045

173 3S -0.00004 0.02276 0.00140 0.00013 0.00001

174 4PX 0.00199 -0.00017 -0.00004 0.00004 0.00004

175 4PY 0.00053 0.00034 0.00057 0.00000 0.00001

176 4PZ 0.00000 0.00003 -0.00001 0.00184 0.00000

177 11 H 1S 0.00000 0.00000 0.00000 0.00000 0.00000

178 2S -0.00001 0.00001 0.00002 0.00001 0.00000

179 3S -0.00029 0.00019 0.00020 0.00004 0.00000

180 4PX 0.00000 0.00000 0.00000 0.00000 0.00000

181 4PY 0.00000 0.00000 0.00000 0.00000 0.00000

182 4PZ 0.00000 0.00000 0.00000 0.00000 0.00000

183 12 H 1S 0.00000 0.00000 0.00000 0.00000 0.00000

184 2S 0.00000 0.00000 0.00000 0.00000 0.00000

185 3S 0.00001 0.00003 0.00000 -0.00002 0.00000

186 4PX 0.00000 0.00000 0.00000 0.00000 0.00000

187 4PY 0.00000 0.00000 0.00000 0.00000 0.00000

188 4PZ 0.00000 0.00000 0.00000 0.00000 0.00000

189 13 H 1S 0.00000 0.00000 0.00000 0.00000 0.00000

190 2S 0.00000 0.00000 0.00000 0.00000 0.00000

191 3S 0.00000 0.00001 0.00000 0.00000 0.00000

192 4PX 0.00000 0.00000 0.00000 0.00000 0.00000

193 4PY 0.00000 0.00000 0.00000 0.00000 0.00000

194 4PZ 0.00000 0.00000 0.00000 0.00000 0.00000

195 14 H 1S 0.00000 0.00000 0.00000 0.00000 0.00000

196 2S 0.00000 0.00000 0.00001 0.00000 0.00000

197 3S -0.00010 0.00001 -0.00008 -0.00001 0.00000

198 4PX 0.00000 0.00000 0.00000 0.00000 0.00000

199 4PY 0.00000 0.00000 0.00000 0.00000 0.00000

200 4PZ 0.00000 0.00000 0.00000 0.00000 0.00000

41 42 43 44 45

41 5D+1 0.00147

42 5D-1 0.00000 0.00233

43 5D+2 0.00000 0.00000 0.00542

44 5D-2 0.00000 0.00000 0.00000 0.00232

45 3 N 1S -0.00003 -0.00005 0.00001 -0.00047 0.65106

46 2S -0.00004 -0.00007 0.00001 -0.00072 0.50540

47 2PX 0.00007 0.00024 0.00056 0.00047 0.00000

48 2PY 0.00035 -0.00003 0.00134 0.00006 0.00000

49 2PZ 0.00050 0.00036 0.00001 0.00026 0.00000

50 3S 0.00030 0.00057 -0.00011 0.00558 -0.03928

51 3PX 0.00013 0.00068 0.00254 0.00089 0.00000

52 3PY 0.00098 -0.00005 0.00524 0.00010 0.00000

53 3PZ 0.00250 0.00170 0.00002 0.00074 0.00000

54 4S 0.00002 0.00021 -0.00007 0.00187 -0.01965

55 4PX -0.00001 0.00018 0.00130 -0.00007 0.00000

56 4PY 0.00034 0.00003 0.00462 0.00007 0.00000

57 4PZ 0.00197 0.00185 0.00001 0.00024 0.00000

58 5D 0 0.00009 0.00000 0.00000 0.00000 0.00000

59 5D+1 0.00000 -0.00005 -0.00001 0.00001 0.00000

60 5D-1 0.00021 0.00004 0.00002 0.00004 0.00000

61 5D+2 0.00002 -0.00001 0.00009 0.00000 0.00000

62 5D-2 0.00002 0.00004 -0.00003 0.00008 0.00000

63 4 C 1S 0.00000 0.00000 0.00000 0.00000 0.00000

64 2S 0.00000 0.00000 0.00000 0.00000 0.00000

65 2PX 0.00001 0.00000 0.00015 0.00000 0.00000

66 2PY 0.00000 0.00000 0.00000 0.00000 0.00000

67 2PZ 0.00001 0.00000 0.00000 0.00000 0.00000

68 3S 0.00005 0.00000 0.00069 -0.00001 -0.00007

69 3PX 0.00010 0.00000 0.00178 -0.00002 -0.00040

70 3PY 0.00000 -0.00001 -0.00003 0.00003 -0.00020

71 3PZ 0.00016 0.00000 0.00005 0.00000 0.00000

72 4S 0.00000 0.00000 0.00154 0.00000 0.00088

73 4PX 0.00014 0.00000 0.00051 -0.00001 0.00032

74 4PY 0.00000 -0.00003 0.00003 -0.00007 -0.00020

75 4PZ 0.00055 0.00002 0.00010 0.00000 0.00001

76 5D 0 0.00001 0.00000 0.00002 0.00000 -0.00003

77 5D+1 0.00004 0.00000 0.00002 0.00000 0.00000

78 5D-1 0.00000 0.00000 0.00000 0.00000 0.00000

79 5D+2 0.00003 0.00000 0.00014 0.00000 -0.00001

80 5D-2 0.00000 0.00000 0.00000 0.00006 -0.00018

81 5 C 1S 0.00000 0.00000 0.00000 0.00000 0.00000

82 2S 0.00000 0.00000 0.00000 0.00000 0.00000

83 2PX 0.00000 0.00000 0.00000 0.00005 0.00000

84 2PY 0.00000 0.00000 0.00000 0.00001 0.00000

85 2PZ 0.00000 0.00000 0.00000 0.00000 0.00000

86 3S 0.00000 0.00000 0.00002 0.00026 0.00000

87 3PX 0.00000 0.00001 0.00003 0.00082 0.00000

88 3PY 0.00000 0.00000 0.00003 0.00007 0.00000

89 3PZ 0.00004 0.00003 0.00000 0.00000 0.00000

90 4S -0.00001 0.00000 -0.00012 0.00064 -0.00005

91 4PX 0.00000 0.00002 -0.00016 0.00095 0.00008

92 4PY 0.00000 0.00000 -0.00028 0.00006 -0.00011

93 4PZ 0.00008 0.00013 0.00001 0.00001 0.00000

94 5D 0 0.00000 0.00000 0.00000 0.00001 0.00000

95 5D+1 0.00000 0.00002 0.00000 0.00000 0.00000

96 5D-1 0.00001 0.00000 0.00000 0.00000 0.00000

97 5D+2 0.00000 0.00000 0.00003 0.00003 0.00000

98 5D-2 0.00000 0.00000 0.00000 -0.00002 0.00000

99 6 C 1S 0.00000 0.00000 0.00000 0.00000 0.00000

100 2S 0.00000 0.00000 0.00000 0.00000 0.00000

101 2PX 0.00000 0.00000 0.00000 0.00000 0.00000

102 2PY 0.00000 0.00000 0.00000 0.00000 0.00000

103 2PZ 0.00000 0.00000 0.00000 0.00000 0.00000

104 3S 0.00000 0.00000 0.00000 0.00000 0.00000

105 3PX 0.00000 0.00000 0.00000 0.00000 0.00000

106 3PY 0.00000 0.00000 0.00000 0.00000 0.00000

107 3PZ 0.00000 0.00000 0.00000 0.00000 0.00000

108 4S 0.00000 0.00000 -0.00004 0.00000 0.00005

109 4PX 0.00000 0.00000 -0.00010 -0.00001 0.00001

110 4PY 0.00000 0.00000 -0.00003 0.00000 0.00000

111 4PZ -0.00001 0.00000 0.00000 0.00000 0.00001

112 5D 0 0.00000 0.00000 0.00000 0.00000 0.00000

113 5D+1 0.00000 0.00000 0.00000 0.00000 0.00000

114 5D-1 0.00000 0.00000 0.00000 0.00000 0.00000

115 5D+2 0.00000 0.00000 0.00000 0.00000 0.00000

116 5D-2 0.00000 0.00000 0.00000 0.00000 0.00000

117 7 C 1S 0.00000 0.00000 0.00000 0.00000 0.00000

118 2S 0.00000 0.00000 0.00000 0.00000 0.00000

119 2PX 0.00000 0.00000 0.00000 0.00000 0.00000

120 2PY 0.00000 0.00000 0.00000 0.00000 0.00000

121 2PZ 0.00000 0.00000 0.00000 0.00000 0.00000

122 3S 0.00000 0.00000 0.00000 0.00000 0.00000

123 3PX 0.00000 0.00000 0.00000 0.00000 0.00000

124 3PY 0.00000 0.00000 0.00000 0.00000 0.00000

125 3PZ 0.00000 0.00000 0.00000 0.00000 0.00000

126 4S 0.00000 0.00000 0.00000 0.00000 0.00000

127 4PX 0.00000 0.00000 0.00000 0.00000 0.00000

128 4PY 0.00000 0.00000 0.00000 0.00000 0.00000

129 4PZ 0.00000 0.00000 0.00000 0.00000 0.00000

130 5D 0 0.00000 0.00000 0.00000 0.00000 0.00000

131 5D+1 0.00000 0.00000 0.00000 0.00000 0.00000

132 5D-1 0.00000 0.00000 0.00000 0.00000 0.00000

133 5D+2 0.00000 0.00000 0.00000 0.00000 0.00000

134 5D-2 0.00000 0.00000 0.00000 0.00000 0.00000

135 8 C 1S 0.00000 0.00000 0.00000 0.00000 0.00000

136 2S 0.00000 0.00000 0.00000 0.00000 0.00000

137 2PX 0.00000 0.00000 0.00000 0.00000 0.00000

138 2PY 0.00000 0.00000 0.00000 0.00000 0.00000

139 2PZ 0.00000 0.00000 0.00000 0.00000 0.00000

140 3S 0.00000 0.00000 0.00000 0.00000 0.00000

141 3PX 0.00000 0.00000 0.00000 0.00000 0.00000

142 3PY 0.00000 0.00000 0.00000 0.00000 0.00000

143 3PZ 0.00000 0.00000 0.00000 0.00000 0.00000

144 4S 0.00000 0.00000 0.00000 0.00000 0.00000

145 4PX 0.00000 0.00000 0.00000 0.00000 0.00000

146 4PY 0.00000 0.00000 0.00000 0.00000 0.00000

147 4PZ 0.00000 0.00000 0.00000 0.00000 0.00000

148 5D 0 0.00000 0.00000 0.00000 0.00000 0.00000

149 5D+1 0.00000 0.00000 0.00000 0.00000 0.00000

150 5D-1 0.00000 0.00000 0.00000 0.00000 0.00000

151 5D+2 0.00000 0.00000 0.00000 0.00000 0.00000

152 5D-2 0.00000 0.00000 0.00000 0.00000 0.00000

153 9 C 1S 0.00000 0.00000 0.00000 0.00000 0.00000

154 2S 0.00000 0.00000 0.00000 0.00000 0.00000

155 2PX 0.00000 0.00000 0.00000 0.00000 0.00000

156 2PY 0.00000 0.00000 0.00000 0.00000 0.00000

157 2PZ 0.00000 0.00000 0.00000 0.00000 0.00000

158 3S 0.00000 0.00000 0.00000 0.00000 0.00000

159 3PX 0.00000 0.00000 0.00000 0.00000 0.00000

160 3PY 0.00000 0.00000 0.00000 0.00000 0.00000

161 3PZ 0.00000 0.00000 0.00000 0.00000 0.00000

162 4S 0.00000 0.00000 0.00001 -0.00001 0.00000

163 4PX 0.00000 0.00000 0.00000 -0.00002 0.00000

164 4PY 0.00000 0.00000 0.00001 -0.00001 0.00000

165 4PZ 0.00000 -0.00001 0.00000 0.00000 0.00000

166 5D 0 0.00000 0.00000 0.00000 0.00000 0.00000

167 5D+1 0.00000 0.00000 0.00000 0.00000 0.00000

168 5D-1 0.00000 0.00000 0.00000 0.00000 0.00000

169 5D+2 0.00000 0.00000 0.00000 0.00000 0.00000

170 5D-2 0.00000 0.00000 0.00000 0.00000 0.00000

171 10 H 1S 0.00005 0.00002 0.00200 0.00079 0.00000

172 2S 0.00005 0.00003 0.00219 0.00087 0.00004

173 3S 0.00000 0.00000 0.00028 0.00010 0.00015

174 4PX 0.00000 0.00000 0.00000 -0.00001 0.00000

175 4PY 0.00000 0.00000 -0.00016 -0.00003 0.00000

176 4PZ -0.00016 -0.00001 0.00000 0.00000 0.00000

177 11 H 1S 0.00000 0.00000 0.00000 0.00000 0.00000

178 2S 0.00000 0.00000 0.00000 0.00000 0.00000

179 3S 0.00000 0.00000 -0.00001 0.00000 -0.00002

180 4PX 0.00000 0.00000 0.00000 0.00000 0.00000

181 4PY 0.00000 0.00000 0.00000 0.00000 0.00000

182 4PZ 0.00000 0.00000 0.00000 0.00000 0.00000

183 12 H 1S 0.00000 0.00000 0.00000 0.00000 0.00000

184 2S 0.00000 0.00000 0.00000 0.00000 0.00000

185 3S 0.00000 0.00000 0.00000 0.00000 0.00000

186 4PX 0.00000 0.00000 0.00000 0.00000 0.00000

187 4PY 0.00000 0.00000 0.00000 0.00000 0.00000

188 4PZ 0.00000 0.00000 0.00000 0.00000 0.00000

189 13 H 1S 0.00000 0.00000 0.00000 0.00000 0.00000

190 2S 0.00000 0.00000 0.00000 0.00000 0.00000

191 3S 0.00000 0.00000 0.00000 0.00000 0.00000

192 4PX 0.00000 0.00000 0.00000 0.00000 0.00000

193 4PY 0.00000 0.00000 0.00000 0.00000 0.00000

194 4PZ 0.00000 0.00000 0.00000 0.00000 0.00000

195 14 H 1S 0.00000 0.00000 0.00000 0.00000 0.00000

196 2S 0.00000 0.00000 0.00000 0.00000 0.00000

197 3S 0.00000 0.00000 0.00000 0.00000 0.00000

198 4PX 0.00000 0.00000 0.00000 0.00000 0.00000

199 4PY 0.00000 0.00000 0.00000 0.00000 0.00000

200 4PZ 0.00000 0.00000 0.00000 0.00000 0.00000

46 47 48 49 50

46 2S 0.50865

47 2PX 0.00000 0.09616

48 2PY 0.00000 0.00000 0.13042

49 2PZ 0.00000 0.00000 0.00000 0.09390

50 3S -0.07974 0.00000 0.00000 0.00000 0.58670

51 3PX 0.00000 0.08781 0.00000 0.00000 0.00000

52 3PY 0.00000 0.00000 0.11451 0.00000 0.00000

53 3PZ 0.00000 0.00000 0.00000 0.08744 0.00000

54 4S -0.03311 0.00000 0.00000 0.00000 0.36680

55 4PX 0.00000 0.01622 0.00000 0.00000 0.00000

56 4PY 0.00000 0.00000 0.03595 0.00000 0.00000

57 4PZ 0.00000 0.00000 0.00000 0.02456 0.00000

58 5D 0 0.00000 0.00000 0.00000 0.00000 0.00000

59 5D+1 0.00000 0.00000 0.00000 0.00000 0.00000

60 5D-1 0.00000 0.00000 0.00000 0.00000 0.00000

61 5D+2 0.00000 0.00000 0.00000 0.00000 0.00000

62 5D-2 0.00000 0.00000 0.00000 0.00000 0.00000

63 4 C 1S 0.00000 -0.00001 0.00000 0.00000 -0.00004

64 2S 0.00000 0.00000 0.00000 0.00000 -0.00005

65 2PX 0.00000 0.00040 0.00016 0.00000 0.00143

66 2PY 0.00000 0.00021 0.00005 0.00000 0.00076

67 2PZ 0.00000 0.00000 0.00000 0.00002 -0.00001

68 3S -0.00011 0.00268 0.00105 0.00002 0.00231

69 3PX -0.00066 0.00597 0.00305 0.00001 0.01169

70 3PY -0.00033 0.00365 0.00056 0.00000 0.00610

71 3PZ 0.00000 0.00001 -0.00001 0.00085 -0.00006

72 4S 0.00130 0.00387 0.00157 -0.00004 -0.01626

73 4PX 0.00019 0.00096 -0.00010 0.00010 0.00349

74 4PY -0.00039 0.00071 0.00038 -0.00001 0.00573

75 4PZ 0.00001 0.00004 0.00000 0.00212 -0.00016

76 5D 0 -0.00005 0.00017 0.00011 0.00000 0.00077

77 5D+1 0.00000 0.00002 0.00001 0.00039 0.00001

78 5D-1 0.00000 0.00001 0.00000 0.00013 0.00000

79 5D+2 -0.00001 -0.00001 0.00103 0.00000 0.00007

80 5D-2 -0.00030 0.00098 -0.00005 0.00000 0.00439

81 5 C 1S 0.00000 0.00000 0.00000 0.00000 0.00000

82 2S 0.00000 0.00000 0.00000 0.00000 0.00000

83 2PX 0.00000 0.00000 0.00000 0.00000 0.00000

84 2PY 0.00000 0.00000 0.00000 0.00000 0.00000

85 2PZ 0.00000 0.00000 0.00000 0.00000 0.00000

86 3S 0.00000 0.00000 -0.00002 0.00000 -0.00006

87 3PX 0.00000 0.00000 -0.00001 0.00000 -0.00009

88 3PY 0.00000 -0.00001 -0.00004 0.00000 -0.00010

89 3PZ 0.00000 0.00000 0.00000 0.00000 -0.00001

90 4S -0.00007 -0.00015 -0.00120 -0.00001 0.00113

91 4PX 0.00017 -0.00001 0.00063 0.00002 -0.00340

92 4PY -0.00012 -0.00051 -0.00062 -0.00002 0.00037

93 4PZ 0.00000 -0.00002 -0.00004 -0.00024 -0.00006

94 5D 0 0.00000 0.00000 0.00000 0.00000 -0.00001

95 5D+1 0.00000 0.00000 0.00000 0.00000 0.00000

96 5D-1 0.00000 0.00000 0.00000 0.00000 0.00000

97 5D+2 0.00000 0.00000 0.00000 0.00000 0.00001

98 5D-2 0.00000 0.00000 0.00001 0.00000 0.00001

99 6 C 1S 0.00000 0.00000 0.00000 0.00000 0.00000

100 2S 0.00000 0.00000 0.00000 0.00000 0.00000

101 2PX 0.00000 0.00000 0.00000 0.00000 0.00000

102 2PY 0.00000 0.00000 0.00000 0.00000 0.00000

103 2PZ 0.00000 0.00000 0.00000 0.00000 0.00000

104 3S 0.00000 0.00000 0.00000 0.00000 -0.00006

105 3PX 0.00000 -0.00002 0.00000 0.00000 -0.00017

106 3PY 0.00000 0.00000 0.00000 0.00000 0.00000

107 3PZ 0.00000 0.00000 0.00000 0.00000 -0.00001

108 4S 0.00007 -0.00037 0.00000 -0.00005 -0.00138

109 4PX 0.00002 -0.00054 -0.00002 -0.00012 -0.00046

110 4PY 0.00001 0.00002 -0.00011 0.00000 -0.00014

111 4PZ 0.00001 -0.00001 0.00000 -0.00005 -0.00012

112 5D 0 0.00000 0.00000 0.00000 0.00000 0.00000

113 5D+1 0.00000 0.00000 0.00000 0.00000 0.00000

114 5D-1 0.00000 0.00000 0.00000 0.00000 0.00000

115 5D+2 0.00000 0.00000 0.00000 0.00000 0.00002

116 5D-2 0.00000 0.00000 0.00000 0.00000 0.00000

117 7 C 1S 0.00000 0.00000 0.00000 0.00000 0.00000

118 2S 0.00000 0.00000 0.00000 0.00000 0.00000

119 2PX 0.00000 0.00000 0.00000 0.00000 0.00000

120 2PY 0.00000 0.00000 0.00000 0.00000 0.00000

121 2PZ 0.00000 0.00000 0.00000 0.00000 0.00000

122 3S 0.00000 0.00000 0.00000 0.00000 0.00000

123 3PX 0.00000 0.00000 0.00000 0.00000 0.00000

124 3PY 0.00000 0.00000 0.00000 0.00000 0.00000

125 3PZ 0.00000 0.00000 0.00000 0.00000 0.00000

126 4S 0.00000 0.00001 0.00000 0.00000 0.00002

127 4PX 0.00000 0.00000 0.00000 -0.00001 0.00000

128 4PY 0.00000 0.00001 0.00000 0.00000 0.00002

129 4PZ 0.00000 0.00001 0.00000 0.00000 0.00003

130 5D 0 0.00000 0.00000 0.00000 0.00000 0.00000

131 5D+1 0.00000 0.00000 0.00000 0.00000 0.00000

132 5D-1 0.00000 0.00000 0.00000 0.00000 0.00000

133 5D+2 0.00000 0.00000 0.00000 0.00000 0.00000

134 5D-2 0.00000 0.00000 0.00000 0.00000 0.00000

135 8 C 1S 0.00000 0.00000 0.00000 0.00000 0.00000

136 2S 0.00000 0.00000 0.00000 0.00000 0.00000

137 2PX 0.00000 0.00000 0.00000 0.00000 0.00000

138 2PY 0.00000 0.00000 0.00000 0.00000 0.00000

139 2PZ 0.00000 0.00000 0.00000 0.00000 0.00000

140 3S 0.00000 0.00000 0.00000 0.00000 0.00000

141 3PX 0.00000 0.00000 0.00000 0.00000 0.00000

142 3PY 0.00000 0.00000 0.00000 0.00000 0.00000

143 3PZ 0.00000 0.00000 0.00000 0.00000 0.00000

144 4S 0.00000 0.00000 0.00000 0.00000 0.00000

145 4PX 0.00000 0.00000 0.00000 0.00000 0.00000

146 4PY 0.00000 0.00000 0.00000 0.00000 0.00000

147 4PZ 0.00000 0.00000 0.00000 0.00000 0.00000

148 5D 0 0.00000 0.00000 0.00000 0.00000 0.00000

149 5D+1 0.00000 0.00000 0.00000 0.00000 0.00000

150 5D-1 0.00000 0.00000 0.00000 0.00000 0.00000

151 5D+2 0.00000 0.00000 0.00000 0.00000 0.00000

152 5D-2 0.00000 0.00000 0.00000 0.00000 0.00000

153 9 C 1S 0.00000 0.00000 0.00000 0.00000 0.00000

154 2S 0.00000 0.00000 0.00000 0.00000 0.00000

155 2PX 0.00000 0.00000 0.00000 0.00000 0.00000

156 2PY 0.00000 0.00000 0.00000 0.00000 0.00000

157 2PZ 0.00000 0.00000 0.00000 0.00000 0.00000

158 3S 0.00000 0.00000 0.00000 0.00000 0.00000

159 3PX 0.00000 0.00000 0.00000 0.00000 0.00000

160 3PY 0.00000 0.00000 0.00000 0.00000 0.00000

161 3PZ 0.00000 0.00000 0.00000 0.00000 0.00000

162 4S 0.00000 0.00000 0.00002 0.00000 -0.00004

163 4PX 0.00000 0.00000 0.00002 0.00000 0.00000

164 4PY 0.00000 0.00001 0.00003 0.00000 -0.00005

165 4PZ 0.00000 0.00000 0.00000 0.00000 0.00000

166 5D 0 0.00000 0.00000 0.00000 0.00000 0.00000

167 5D+1 0.00000 0.00000 0.00000 0.00000 0.00000

168 5D-1 0.00000 0.00000 0.00000 0.00000 0.00000

169 5D+2 0.00000 0.00000 0.00000 0.00000 0.00000

170 5D-2 0.00000 0.00000 0.00000 0.00000 0.00000

171 10 H 1S 0.00000 0.00000 0.00000 0.00000 -0.00006

172 2S 0.00006 -0.00024 0.00005 0.00000 -0.00243

173 3S 0.00026 -0.00049 0.00007 0.00000 -0.00456

174 4PX 0.00000 0.00001 0.00000 0.00000 0.00000

175 4PY 0.00000 0.00000 0.00000 0.00000 0.00001

176 4PZ 0.00000 0.00000 0.00000 0.00000 0.00000

177 11 H 1S 0.00000 0.00000 0.00000 0.00000 0.00000

178 2S 0.00000 0.00000 0.00000 0.00000 -0.00002

179 3S -0.00004 -0.00010 0.00008 0.00000 0.00083

180 4PX 0.00000 0.00000 0.00000 0.00000 0.00000

181 4PY 0.00000 0.00000 0.00000 0.00000 0.00000

182 4PZ 0.00000 0.00000 0.00000 0.00000 0.00000

183 12 H 1S 0.00000 0.00000 0.00000 0.00000 0.00000

184 2S 0.00000 0.00000 0.00000 0.00000 0.00000

185 3S 0.00000 0.00000 0.00000 0.00000 0.00000

186 4PX 0.00000 0.00000 0.00000 0.00000 0.00000

187 4PY 0.00000 0.00000 0.00000 0.00000 0.00000

188 4PZ 0.00000 0.00000 0.00000 0.00000 0.00000

189 13 H 1S 0.00000 0.00000 0.00000 0.00000 0.00000

190 2S 0.00000 0.00000 0.00000 0.00000 0.00000

191 3S 0.00000 0.00000 0.00000 0.00000 0.00000

192 4PX 0.00000 0.00000 0.00000 0.00000 0.00000

193 4PY 0.00000 0.00000 0.00000 0.00000 0.00000

194 4PZ 0.00000 0.00000 0.00000 0.00000 0.00000

195 14 H 1S 0.00000 0.00000 0.00000 0.00000 0.00000

196 2S 0.00000 0.00000 0.00000 0.00000 0.00000

197 3S 0.00000 0.00000 0.00000 0.00000 0.00000

198 4PX 0.00000 0.00000 0.00000 0.00000 0.00000

199 4PY 0.00000 0.00000 0.00000 0.00000 0.00000

200 4PZ 0.00000 0.00000 0.00000 0.00000 0.00000

51 52 53 54 55

51 3PX 0.20876

52 3PY 0.00000 0.26180

53 3PZ 0.00000 0.00000 0.21196

54 4S 0.00000 0.00000 0.00000 0.42400

55 4PX 0.07321 0.00000 0.00000 0.00000 0.06413

56 4PY 0.00000 0.15549 0.00000 0.00000 0.00000

57 4PZ 0.00000 0.00000 0.11267 0.00000 0.00000

58 5D 0 0.00000 0.00000 0.00000 0.00000 0.00000

59 5D+1 0.00000 0.00000 0.00000 0.00000 0.00000

60 5D-1 0.00000 0.00000 0.00000 0.00000 0.00000

61 5D+2 0.00000 0.00000 0.00000 0.00000 0.00000

62 5D-2 0.00000 0.00000 0.00000 0.00000 0.00000

63 4 C 1S -0.00044 -0.00016 0.00000 -0.00039 -0.00110

64 2S -0.00071 -0.00026 0.00000 -0.00052 -0.00200

65 2PX 0.00647 0.00290 0.00003 0.00271 0.00315

66 2PY 0.00381 0.00060 0.00000 0.00093 0.00329

67 2PZ 0.00001 -0.00001 0.00076 -0.00001 0.00001

68 3S 0.01944 0.00725 0.00012 0.00277 0.02074

69 3PX 0.02654 0.01542 0.00005 0.01348 0.00956

70 3PY 0.01858 0.00118 0.00001 0.00445 0.01356

71 3PZ 0.00005 -0.00007 0.00609 -0.00003 0.00004

72 4S 0.02244 0.00830 -0.00025 -0.02886 0.02815

73 4PX 0.00342 -0.00027 0.00051 0.01154 -0.00128

74 4PY 0.00349 0.00241 -0.00008 0.00866 0.00413

75 4PZ 0.00020 0.00001 0.01216 -0.00011 0.00024

76 5D 0 0.00056 0.00033 0.00002 0.00036 0.00004

77 5D+1 0.00006 0.00002 0.00227 0.00001 0.00001

78 5D-1 0.00003 0.00000 0.00079 -0.00001 0.00001

79 5D+2 0.00000 0.00456 0.00000 -0.00039 -0.00010

80 5D-2 0.00339 -0.00008 0.00001 0.00268 0.00060

81 5 C 1S 0.00000 0.00000 0.00000 -0.00002 0.00001

82 2S 0.00000 0.00000 0.00000 -0.00002 0.00002

83 2PX 0.00000 0.00000 0.00000 -0.00008 0.00001

84 2PY -0.00001 -0.00002 0.00000 -0.00003 -0.00030

85 2PZ 0.00000 0.00000 0.00000 -0.00001 -0.00001

86 3S -0.00003 -0.00042 0.00000 0.00069 -0.00035

87 3PX 0.00003 -0.00023 0.00000 -0.00049 -0.00001

88 3PY -0.00029 -0.00079 -0.00002 0.00008 -0.00186

89 3PZ -0.00001 -0.00001 -0.00007 -0.00008 -0.00004

90 4S -0.00113 -0.00799 -0.00003 0.00897 -0.00251

91 4PX -0.00020 0.00370 0.00014 -0.00793 -0.00139

92 4PY -0.00319 -0.00327 -0.00014 0.00227 -0.00579

93 4PZ -0.00010 -0.00023 -0.00172 -0.00025 -0.00015

94 5D 0 0.00000 0.00003 0.00001 -0.00002 0.00001

95 5D+1 0.00000 0.00000 0.00000 0.00000 0.00000

96 5D-1 0.00000 0.00001 0.00002 -0.00001 -0.00001

97 5D+2 0.00003 0.00008 0.00000 -0.00002 0.00005

98 5D-2 0.00004 0.00014 0.00000 0.00004 0.00001

99 6 C 1S 0.00000 0.00000 0.00000 0.00002 0.00004

100 2S 0.00000 0.00000 0.00000 0.00004 0.00007

101 2PX -0.00001 0.00000 0.00000 -0.00032 -0.00048

102 2PY 0.00000 0.00000 0.00000 0.00000 0.00000

103 2PZ 0.00000 0.00000 0.00000 -0.00002 -0.00003

104 3S -0.00016 0.00000 0.00000 -0.00148 -0.00174

105 3PX -0.00039 0.00001 0.00000 -0.00260 -0.00293

106 3PY 0.00001 -0.00001 0.00000 -0.00004 -0.00003

107 3PZ -0.00005 0.00000 -0.00002 -0.00010 -0.00021

108 4S -0.00268 0.00003 -0.00033 -0.00564 -0.00690

109 4PX -0.00325 -0.00011 -0.00078 -0.00311 -0.00648

110 4PY 0.00015 -0.00076 0.00002 -0.00042 0.00018

111 4PZ -0.00005 0.00001 -0.00034 -0.00030 0.00011

112 5D 0 0.00001 0.00000 0.00000 0.00004 0.00003

113 5D+1 0.00000 0.00000 0.00001 0.00001 0.00001

114 5D-1 0.00000 0.00000 0.00000 0.00000 0.00000

115 5D+2 0.00013 0.00001 0.00001 0.00012 0.00045

116 5D-2 0.00001 -0.00001 0.00000 0.00002 0.00007

117 7 C 1S 0.00000 0.00000 0.00000 0.00000 0.00000

118 2S 0.00000 0.00000 0.00000 0.00000 0.00000

119 2PX 0.00000 0.00000 0.00000 0.00000 0.00000

120 2PY 0.00000 0.00000 0.00000 0.00000 0.00000

121 2PZ 0.00000 0.00000 0.00000 0.00000 0.00000

122 3S 0.00000 0.00000 0.00000 0.00002 0.00004

123 3PX 0.00000 0.00000 0.00000 0.00000 -0.00001

124 3PY 0.00000 0.00000 0.00000 0.00001 0.00002

125 3PZ 0.00000 0.00000 0.00000 0.00001 0.00004

126 4S 0.00009 0.00001 -0.00001 0.00019 0.00064

127 4PX 0.00000 0.00004 -0.00007 -0.00022 -0.00014

128 4PY 0.00005 0.00000 0.00000 0.00016 0.00031

129 4PZ 0.00008 0.00000 0.00000 0.00028 0.00050

130 5D 0 0.00000 0.00000 0.00000 0.00000 0.00000

131 5D+1 0.00000 0.00000 0.00000 0.00000 0.00000

132 5D-1 0.00000 0.00000 0.00000 0.00000 0.00000

133 5D+2 0.00000 0.00000 0.00000 0.00000 0.00000

134 5D-2 0.00000 0.00000 0.00000 0.00000 0.00000

135 8 C 1S 0.00000 0.00000 0.00000 0.00000 0.00000

136 2S 0.00000 0.00000 0.00000 0.00000 0.00000

137 2PX 0.00000 0.00000 0.00000 0.00000 0.00000

138 2PY 0.00000 0.00000 0.00000 0.00000 0.00000

139 2PZ 0.00000 0.00000 0.00000 0.00000 0.00000

140 3S 0.00000 0.00000 0.00000 0.00000 0.00000

141 3PX 0.00000 0.00000 0.00000 0.00000 0.00001

142 3PY 0.00000 0.00000 0.00000 0.00000 0.00000

143 3PZ 0.00000 0.00000 0.00000 0.00000 0.00000

144 4S 0.00000 0.00000 0.00000 -0.00002 -0.00002

145 4PX 0.00000 -0.00001 0.00000 0.00010 0.00005

146 4PY 0.00001 0.00000 0.00000 0.00007 0.00006

147 4PZ 0.00000 0.00000 0.00000 0.00000 0.00000

148 5D 0 0.00000 0.00000 0.00000 0.00000 0.00000

149 5D+1 0.00000 0.00000 0.00000 0.00000 0.00000

150 5D-1 0.00000 0.00000 0.00000 0.00000 0.00000

151 5D+2 0.00000 0.00000 0.00000 0.00000 0.00000

152 5D-2 0.00000 0.00000 0.00000 0.00000 0.00000

153 9 C 1S 0.00000 0.00000 0.00000 0.00000 0.00000

154 2S 0.00000 0.00000 0.00000 0.00000 0.00000

155 2PX 0.00000 0.00000 0.00000 0.00000 0.00000

156 2PY 0.00000 0.00000 0.00000 0.00000 0.00000

157 2PZ 0.00000 0.00000 0.00000 0.00000 0.00000

158 3S 0.00000 0.00000 0.00000 0.00000 0.00002

159 3PX 0.00000 0.00000 0.00000 0.00001 0.00001

160 3PY 0.00000 0.00000 0.00000 0.00003 0.00005

161 3PZ 0.00000 0.00000 0.00000 0.00000 0.00000

162 4S 0.00005 0.00023 0.00000 -0.00059 0.00035

163 4PX -0.00002 0.00018 0.00000 -0.00018 -0.00005

164 4PY 0.00013 0.00029 0.00000 -0.00031 0.00072

165 4PZ 0.00000 0.00000 0.00001 0.00000 0.00001

166 5D 0 0.00000 0.00000 0.00000 0.00000 0.00000

167 5D+1 0.00000 0.00000 0.00000 0.00000 0.00000

168 5D-1 0.00000 0.00000 0.00000 0.00000 0.00000

169 5D+2 0.00000 0.00000 0.00000 0.00000 0.00000

170 5D-2 0.00000 0.00000 0.00000 0.00000 0.00000

171 10 H 1S -0.00012 0.00000 0.00000 -0.00110 -0.00111

172 2S -0.00223 0.00039 0.00002 -0.00810 -0.00529

173 3S -0.00304 0.00041 0.00000 -0.00844 -0.00529

174 4PX 0.00012 0.00001 0.00001 -0.00006 0.00022

175 4PY 0.00003 -0.00001 0.00000 0.00001 0.00006

176 4PZ 0.00002 0.00000 0.00003 0.00000 0.00005

177 11 H 1S 0.00000 0.00000 0.00000 -0.00004 -0.00012

178 2S -0.00008 0.00001 0.00000 -0.00033 -0.00099

179 3S -0.00073 0.00055 -0.00002 0.00310 -0.00228

180 4PX 0.00000 0.00000 0.00000 -0.00001 -0.00003

181 4PY 0.00000 0.00000 0.00000 0.00000 0.00000

182 4PZ 0.00000 0.00000 0.00000 0.00000 0.00000

183 12 H 1S 0.00000 0.00000 0.00000 0.00000 0.00000

184 2S 0.00000 0.00000 0.00000 0.00000 0.00000

185 3S -0.00001 0.00000 0.00000 -0.00003 -0.00006

186 4PX 0.00000 0.00000 0.00000 0.00000 0.00000

187 4PY 0.00000 0.00000 0.00000 0.00000 0.00000

188 4PZ 0.00000 0.00000 0.00000 0.00000 0.00000

189 13 H 1S 0.00000 0.00000 0.00000 0.00000 0.00000

190 2S 0.00000 0.00000 0.00000 0.00000 0.00000

191 3S 0.00000 0.00000 0.00000 0.00000 -0.00001

192 4PX 0.00000 0.00000 0.00000 0.00000 0.00000

193 4PY 0.00000 0.00000 0.00000 0.00000 0.00000

194 4PZ 0.00000 0.00000 0.00000 0.00000 0.00000

195 14 H 1S 0.00000 0.00000 0.00000 0.00000 0.00000

196 2S 0.00000 0.00000 0.00000 0.00000 0.00000

197 3S 0.00000 -0.00002 0.00000 0.00001 -0.00005

198 4PX 0.00000 0.00000 0.00000 0.00000 0.00000

199 4PY 0.00000 0.00000 0.00000 0.00000 0.00000

200 4PZ 0.00000 0.00000 0.00000 0.00000 0.00000

56 57 58 59 60

56 4PY 0.23568

57 4PZ 0.00000 0.15115

58 5D 0 0.00000 0.00000 0.00087

59 5D+1 0.00000 0.00000 0.00000 0.00071

60 5D-1 0.00000 0.00000 0.00000 0.00000 0.00160

61 5D+2 0.00000 0.00000 0.00000 0.00000 0.00000

62 5D-2 0.00000 0.00000 0.00000 0.00000 0.00000

63 4 C 1S -0.00090 -0.00001 -0.00002 0.00000 0.00000

64 2S -0.00169 -0.00001 -0.00003 0.00000 0.00000

65 2PX 0.00374 0.00000 0.00016 0.00000 0.00000

66 2PY -0.00065 -0.00001 0.00006 0.00000 0.00000

67 2PZ -0.00003 0.00232 0.00000 0.00016 0.00007

68 3S 0.01856 0.00012 0.00050 0.00001 0.00001

69 3PX 0.01698 -0.00009 0.00043 -0.00001 0.00000

70 3PY -0.00612 -0.00004 0.00018 -0.00001 0.00000

71 3PZ -0.00011 0.01177 0.00001 0.00103 0.00046

72 4S 0.02774 -0.00076 0.00011 0.00001 0.00000

73 4PX 0.00085 0.00066 0.00000 0.00000 0.00002

74 4PY 0.00609 -0.00014 0.00000 0.00000 0.00000

75 4PZ 0.00001 0.02725 0.00001 0.00070 0.00033

76 5D 0 0.00003 0.00001 0.00003 0.00001 0.00000

77 5D+1 0.00001 0.00232 -0.00001 -0.00005 0.00017

78 5D-1 -0.00001 0.00080 0.00000 -0.00005 0.00002

79 5D+2 0.00363 0.00000 0.00003 0.00000 0.00000

80 5D-2 0.00012 0.00000 0.00000 0.00000 0.00000

81 5 C 1S 0.00011 0.00000 0.00000 0.00000 0.00000

82 2S 0.00021 0.00000 0.00000 0.00000 0.00000

83 2PX -0.00005 0.00000 0.00000 0.00000 0.00000

84 2PY -0.00113 -0.00002 0.00000 0.00000 0.00000

85 2PZ -0.00002 -0.00012 0.00000 0.00000 0.00000

86 3S -0.00529 0.00002 0.00000 0.00000 0.00000

87 3PX -0.00092 -0.00001 0.00000 0.00000 0.00000

88 3PY -0.00653 -0.00016 0.00000 0.00001 0.00000

89 3PZ -0.00012 -0.00102 0.00000 0.00001 0.00000

90 4S -0.02889 -0.00011 -0.00007 0.00000 -0.00001

91 4PX 0.01146 0.00038 0.00004 0.00000 0.00001

92 4PY -0.01304 -0.00057 0.00000 0.00002 0.00000

93 4PZ -0.00071 -0.00600 -0.00002 0.00010 -0.00004

94 5D 0 0.00008 0.00003 0.00000 0.00000 0.00000

95 5D+1 0.00001 -0.00007 0.00000 0.00000 0.00000

96 5D-1 0.00004 0.00017 0.00000 0.00000 0.00000

97 5D+2 0.00021 0.00001 0.00000 0.00000 0.00000

98 5D-2 0.00048 0.00000 0.00000 0.00000 0.00000

99 6 C 1S 0.00000 0.00000 0.00000 0.00000 0.00000

100 2S 0.00000 0.00000 0.00000 0.00000 0.00000

101 2PX 0.00001 -0.00002 0.00000 0.00000 0.00000

102 2PY 0.00000 0.00000 0.00000 0.00000 0.00000

103 2PZ 0.00000 -0.00005 0.00000 0.00000 0.00000

104 3S 0.00013 0.00002 0.00000 0.00000 0.00000

105 3PX 0.00014 -0.00016 0.00001 0.00001 0.00000

106 3PY -0.00003 -0.00001 0.00000 0.00000 0.00000

107 3PZ 0.00000 -0.00045 0.00000 0.00001 0.00000

108 4S 0.00014 -0.00131 0.00001 -0.00001 0.00000

109 4PX -0.00032 -0.00241 0.00002 0.00003 0.00000

110 4PY -0.00294 0.00005 0.00000 0.00000 -0.00001

111 4PZ 0.00005 -0.00197 0.00000 0.00007 0.00000

112 5D 0 -0.00001 0.00004 0.00000 0.00000 0.00000

113 5D+1 0.00000 0.00007 0.00000 0.00000 0.00000

114 5D-1 -0.00001 0.00000 0.00000 0.00000 0.00000

115 5D+2 0.00006 0.00006 0.00000 0.00000 0.00000

116 5D-2 -0.00013 0.00000 0.00000 0.00000 0.00000

117 7 C 1S 0.00000 0.00000 0.00000 0.00000 0.00000

118 2S 0.00000 0.00000 0.00000 0.00000 0.00000

119 2PX 0.00000 0.00000 0.00000 0.00000 0.00000

120 2PY 0.00000 0.00000 0.00000 0.00000 0.00000

121 2PZ 0.00000 0.00000 0.00000 0.00000 0.00000

122 3S 0.00000 0.00000 0.00000 0.00000 0.00000

123 3PX 0.00000 -0.00005 0.00000 0.00000 0.00000

124 3PY 0.00000 0.00001 0.00000 0.00000 0.00000

125 3PZ 0.00000 0.00000 0.00000 0.00000 0.00000

126 4S 0.00014 -0.00009 0.00000 0.00000 0.00000

127 4PX 0.00028 -0.00066 0.00000 0.00000 0.00000

128 4PY -0.00002 0.00007 0.00000 0.00000 0.00000

129 4PZ -0.00002 -0.00011 0.00000 0.00000 0.00000

130 5D 0 0.00000 0.00000 0.00000 0.00000 0.00000

131 5D+1 0.00000 0.00000 0.00000 0.00000 0.00000

132 5D-1 0.00000 0.00000 0.00000 0.00000 0.00000

133 5D+2 0.00000 0.00000 0.00000 0.00000 0.00000

134 5D-2 0.00000 0.00000 0.00000 0.00000 0.00000

135 8 C 1S 0.00000 0.00000 0.00000 0.00000 0.00000

136 2S 0.00000 0.00000 0.00000 0.00000 0.00000

137 2PX 0.00000 0.00000 0.00000 0.00000 0.00000

138 2PY 0.00000 0.00000 0.00000 0.00000 0.00000

139 2PZ 0.00000 0.00000 0.00000 0.00000 0.00000

140 3S 0.00000 0.00000 0.00000 0.00000 0.00000

141 3PX 0.00000 0.00000 0.00000 0.00000 0.00000

142 3PY 0.00000 0.00000 0.00000 0.00000 0.00000

143 3PZ 0.00000 0.00000 0.00000 0.00000 0.00000

144 4S 0.00000 0.00000 0.00000 0.00000 0.00000

145 4PX -0.00015 0.00001 0.00000 0.00000 0.00000

146 4PY -0.00008 0.00001 0.00000 0.00000 0.00000

147 4PZ 0.00000 0.00006 0.00000 0.00000 0.00000

148 5D 0 0.00000 0.00000 0.00000 0.00000 0.00000

149 5D+1 0.00000 0.00000 0.00000 0.00000 0.00000

150 5D-1 0.00000 0.00000 0.00000 0.00000 0.00000

151 5D+2 0.00000 0.00000 0.00000 0.00000 0.00000

152 5D-2 0.00000 0.00000 0.00000 0.00000 0.00000

153 9 C 1S 0.00000 0.00000 0.00000 0.00000 0.00000

154 2S 0.00000 0.00000 0.00000 0.00000 0.00000

155 2PX 0.00000 0.00000 0.00000 0.00000 0.00000

156 2PY 0.00001 0.00000 0.00000 0.00000 0.00000

157 2PZ 0.00000 0.00000 0.00000 0.00000 0.00000

158 3S 0.00005 0.00000 0.00000 0.00000 0.00000

159 3PX 0.00005 0.00000 0.00000 0.00000 0.00000

160 3PY 0.00012 0.00000 0.00000 0.00000 0.00000

161 3PZ 0.00000 0.00000 0.00000 0.00000 0.00000

162 4S 0.00217 0.00003 0.00000 0.00000 0.00000

163 4PX 0.00104 -0.00001 0.00000 0.00000 0.00000

164 4PY 0.00233 0.00004 0.00000 0.00000 0.00000

165 4PZ -0.00002 0.00017 0.00000 0.00000 0.00000

166 5D 0 0.00000 0.00000 0.00000 0.00000 0.00000

167 5D+1 0.00000 0.00000 0.00000 0.00000 0.00000

168 5D-1 0.00000 0.00000 0.00000 0.00000 0.00000

169 5D+2 -0.00001 0.00000 0.00000 0.00000 0.00000

170 5D-2 0.00000 0.00000 0.00000 0.00000 0.00000

171 10 H 1S 0.00060 0.00005 0.00000 0.00000 0.00000

172 2S 0.00346 0.00043 0.00006 0.00001 0.00000

173 3S 0.00235 0.00026 0.00002 0.00000 0.00000

174 4PX 0.00008 0.00005 0.00000 0.00000 0.00000

175 4PY -0.00011 0.00001 0.00000 0.00000 0.00000

176 4PZ 0.00001 0.00023 0.00000 0.00000 0.00000

177 11 H 1S 0.00002 0.00000 0.00000 0.00000 0.00000

178 2S 0.00018 -0.00002 0.00000 0.00000 0.00000

179 3S 0.00258 -0.00010 -0.00001 0.00000 0.00000

180 4PX 0.00002 0.00000 0.00000 0.00000 0.00000

181 4PY 0.00000 0.00000 0.00000 0.00000 0.00000

182 4PZ 0.00000 -0.00001 0.00000 0.00000 0.00000

183 12 H 1S 0.00000 0.00000 0.00000 0.00000 0.00000

184 2S 0.00000 0.00000 0.00000 0.00000 0.00000

185 3S 0.00000 0.00003 0.00000 0.00000 0.00000

186 4PX 0.00000 0.00000 0.00000 0.00000 0.00000

187 4PY 0.00000 0.00000 0.00000 0.00000 0.00000

188 4PZ 0.00000 0.00000 0.00000 0.00000 0.00000

189 13 H 1S 0.00000 0.00000 0.00000 0.00000 0.00000

190 2S 0.00000 0.00000 0.00000 0.00000 0.00000

191 3S -0.00001 0.00000 0.00000 0.00000 0.00000

192 4PX 0.00000 0.00000 0.00000 0.00000 0.00000

193 4PY 0.00000 0.00000 0.00000 0.00000 0.00000

194 4PZ 0.00000 0.00000 0.00000 0.00000 0.00000

195 14 H 1S 0.00000 0.00000 0.00000 0.00000 0.00000

196 2S -0.00001 0.00000 0.00000 0.00000 0.00000

197 3S -0.00023 -0.00001 0.00000 0.00000 0.00000

198 4PX 0.00000 0.00000 0.00000 0.00000 0.00000

199 4PY 0.00000 0.00000 0.00000 0.00000 0.00000

200 4PZ 0.00000 0.00000 0.00000 0.00000 0.00000

61 62 63 64 65

61 5D+2 0.00141

62 5D-2 0.00000 0.00260

63 4 C 1S 0.00000 -0.00006 0.65856

64 2S -0.00001 -0.00009 0.49230 0.48990

65 2PX -0.00006 0.00083 0.00000 0.00000 0.08210

66 2PY 0.00013 0.00011 0.00000 0.00000 0.00000

67 2PZ 0.00000 0.00000 0.00000 0.00000 0.00000

68 3S 0.00010 0.00179 -0.02830 -0.06247 0.00000

69 3PX -0.00001 0.00233 0.00000 0.00000 0.07671

70 3PY 0.00052 0.00018 0.00000 0.00000 0.00000

71 3PZ 0.00000 0.00000 0.00000 0.00000 0.00000

72 4S 0.00009 0.00077 -0.01184 -0.02053 0.00000

73 4PX -0.00003 0.00015 0.00000 0.00000 0.01234

74 4PY 0.00023 -0.00002 0.00000 0.00000 0.00000

75 4PZ 0.00000 0.00000 0.00000 0.00000 0.00000

76 5D 0 0.00000 0.00003 0.00000 0.00000 0.00000

77 5D+1 0.00000 0.00001 0.00000 0.00000 0.00000

78 5D-1 0.00000 0.00000 0.00000 0.00000 0.00000

79 5D+2 0.00016 -0.00012 0.00000 0.00000 0.00000

80 5D-2 0.00005 0.00012 0.00000 0.00000 0.00000

81 5 C 1S 0.00000 0.00000 0.00000 0.00000 0.00000

82 2S 0.00000 0.00000 0.00000 0.00000 0.00000

83 2PX 0.00000 0.00000 0.00000 0.00000 0.00002

84 2PY 0.00000 0.00000 -0.00003 -0.00002 0.00007

85 2PZ 0.00000 0.00000 0.00000 0.00000 0.00000

86 3S 0.00000 0.00001 -0.00037 -0.00061 0.00027

87 3PX -0.00002 0.00001 -0.00003 -0.00005 0.00038

88 3PY 0.00002 0.00005 -0.00144 -0.00247 0.00056

89 3PZ 0.00000 0.00000 -0.00008 -0.00015 0.00003

90 4S -0.00011 -0.00001 0.00038 0.00038 0.00000

91 4PX 0.00001 -0.00001 0.00019 0.00029 0.00155

92 4PY 0.00007 -0.00005 -0.00094 -0.00195 0.00028

93 4PZ 0.00001 0.00000 -0.00010 -0.00019 -0.00001

94 5D 0 0.00000 0.00000 -0.00004 -0.00007 0.00001

95 5D+1 0.00000 0.00000 0.00000 0.00000 0.00001

96 5D-1 0.00000 0.00000 -0.00005 -0.00008 0.00001

97 5D+2 0.00000 0.00001 -0.00022 -0.00035 0.00023

98 5D-2 0.00001 0.00000 -0.00004 -0.00007 0.00040

99 6 C 1S 0.00000 0.00000 0.00000 0.00000 -0.00003

100 2S 0.00000 0.00000 0.00000 0.00000 -0.00002

101 2PX 0.00000 0.00000 -0.00003 -0.00002 0.00106

102 2PY 0.00000 0.00000 -0.00001 -0.00001 0.00039

103 2PZ 0.00000 0.00000 0.00000 0.00000 0.00024

104 3S 0.00001 0.00000 -0.00038 -0.00062 0.00511

105 3PX 0.00004 -0.00001 -0.00116 -0.00198 0.00785

106 3PY 0.00000 0.00000 -0.00032 -0.00056 0.00346

107 3PZ 0.00000 0.00000 -0.00020 -0.00036 0.00225

108 4S 0.00000 -0.00002 0.00012 -0.00005 0.00316

109 4PX 0.00008 -0.00003 -0.00021 -0.00056 0.00103

110 4PY -0.00001 -0.00005 -0.00008 -0.00030 0.00190

111 4PZ 0.00000 0.00000 -0.00027 -0.00050 0.00088

112 5D 0 0.00000 0.00000 -0.00003 -0.00005 0.00003

113 5D+1 0.00000 0.00000 -0.00005 -0.00009 0.00032

114 5D-1 0.00000 0.00000 -0.00002 -0.00004 0.00020

115 5D+2 0.00000 0.00000 -0.00010 -0.00015 0.00007

116 5D-2 0.00000 0.00000 -0.00015 -0.00023 0.00082

117 7 C 1S 0.00000 0.00000 0.00000 0.00000 0.00000

118 2S 0.00000 0.00000 0.00000 0.00000 0.00000

119 2PX 0.00000 0.00000 0.00000 0.00000 0.00000

120 2PY 0.00000 0.00000 0.00000 0.00000 0.00000

121 2PZ 0.00000 0.00000 0.00000 0.00000 0.00000

122 3S 0.00000 0.00000 0.00000 0.00000 -0.00001

123 3PX 0.00000 0.00000 0.00000 0.00000 -0.00002

124 3PY 0.00000 0.00000 0.00000 0.00000 0.00000

125 3PZ 0.00000 0.00000 0.00000 0.00000 -0.00001

126 4S 0.00000 0.00000 0.00002 0.00005 -0.00055

127 4PX 0.00000 0.00000 0.00009 0.00019 -0.00059

128 4PY 0.00000 0.00000 0.00000 0.00000 0.00001

129 4PZ 0.00000 0.00000 0.00001 0.00003 -0.00024

130 5D 0 0.00000 0.00000 0.00000 0.00000 0.00000

131 5D+1 0.00000 0.00000 0.00000 0.00000 0.00001

132 5D-1 0.00000 0.00000 0.00000 0.00000 0.00000

133 5D+2 0.00000 0.00000 0.00000 0.00000 0.00001

134 5D-2 0.00000 0.00000 0.00000 0.00000 0.00000

135 8 C 1S 0.00000 0.00000 0.00000 0.00000 0.00000

136 2S 0.00000 0.00000 0.00000 0.00000 0.00000

137 2PX 0.00000 0.00000 0.00000 0.00000 0.00000

138 2PY 0.00000 0.00000 0.00000 0.00000 0.00000

139 2PZ 0.00000 0.00000 0.00000 0.00000 0.00000

140 3S 0.00000 0.00000 0.00000 0.00000 0.00000

141 3PX 0.00000 0.00000 0.00000 0.00000 0.00000

142 3PY 0.00000 0.00000 0.00000 0.00000 0.00000

143 3PZ 0.00000 0.00000 0.00000 0.00000 0.00000

144 4S 0.00000 0.00000 0.00002 0.00003 -0.00008

145 4PX 0.00000 0.00000 0.00003 0.00005 0.00005

146 4PY 0.00000 0.00000 0.00005 0.00008 -0.00019

147 4PZ 0.00000 0.00000 0.00000 0.00000 0.00000

148 5D 0 0.00000 0.00000 0.00000 0.00000 0.00000

149 5D+1 0.00000 0.00000 0.00000 0.00000 0.00000

150 5D-1 0.00000 0.00000 0.00000 0.00000 0.00000

151 5D+2 0.00000 0.00000 0.00000 0.00000 0.00000

152 5D-2 0.00000 0.00000 0.00000 0.00000 0.00000

153 9 C 1S 0.00000 0.00000 0.00000 0.00000 0.00000

154 2S 0.00000 0.00000 0.00000 0.00000 0.00000

155 2PX 0.00000 0.00000 0.00000 0.00000 0.00000

156 2PY 0.00000 0.00000 0.00000 0.00000 0.00000

157 2PZ 0.00000 0.00000 0.00000 0.00000 0.00000

158 3S 0.00000 0.00000 0.00000 0.00000 0.00000

159 3PX 0.00000 0.00000 0.00000 0.00000 0.00000

160 3PY 0.00000 0.00000 0.00000 0.00000 0.00000

161 3PZ 0.00000 0.00000 0.00000 0.00000 0.00000

162 4S 0.00000 0.00000 -0.00006 -0.00007 -0.00015

163 4PX 0.00001 0.00000 0.00001 0.00002 0.00003

164 4PY 0.00000 0.00000 0.00001 0.00006 -0.00010

165 4PZ 0.00000 0.00000 0.00001 0.00002 -0.00001

166 5D 0 0.00000 0.00000 0.00000 0.00000 0.00000

167 5D+1 0.00000 0.00000 0.00000 0.00000 0.00000

168 5D-1 0.00000 0.00000 0.00000 0.00000 0.00000

169 5D+2 0.00000 0.00000 0.00000 0.00000 0.00000

170 5D-2 0.00000 0.00000 0.00000 0.00000 0.00000

171 10 H 1S 0.00002 -0.00001 0.00000 0.00000 0.00000

172 2S 0.00026 -0.00008 0.00000 0.00000 0.00000

173 3S 0.00008 -0.00005 -0.00001 -0.00002 0.00016

174 4PX 0.00002 0.00001 0.00000 0.00000 0.00000

175 4PY 0.00000 0.00000 0.00000 0.00000 0.00000

176 4PZ 0.00000 0.00000 0.00000 0.00000 0.00000

177 11 H 1S 0.00000 0.00000 0.00000 0.00000 0.00000

178 2S 0.00000 0.00000 0.00001 0.00002 -0.00011

179 3S 0.00001 0.00001 0.00016 0.00028 -0.00022

180 4PX 0.00000 0.00000 0.00000 0.00000 0.00000

181 4PY 0.00000 0.00000 0.00000 0.00000 0.00000

182 4PZ 0.00000 0.00000 0.00000 0.00000 0.00000

183 12 H 1S 0.00000 0.00000 0.00000 0.00000 0.00000

184 2S 0.00000 0.00000 0.00000 0.00000 0.00000

185 3S 0.00000 0.00000 -0.00001 -0.00001 0.00005

186 4PX 0.00000 0.00000 0.00000 0.00000 0.00000

187 4PY 0.00000 0.00000 0.00000 0.00000 0.00000

188 4PZ 0.00000 0.00000 0.00000 0.00000 0.00000

189 13 H 1S 0.00000 0.00000 0.00000 0.00000 0.00000

190 2S 0.00000 0.00000 0.00000 0.00000 0.00000

191 3S 0.00000 0.00000 0.00000 0.00000 0.00001

192 4PX 0.00000 0.00000 0.00000 0.00000 0.00000

193 4PY 0.00000 0.00000 0.00000 0.00000 0.00000

194 4PZ 0.00000 0.00000 0.00000 0.00000 0.00000

195 14 H 1S 0.00000 0.00000 0.00000 0.00000 0.00000

196 2S 0.00000 0.00000 0.00000 0.00000 0.00000

197 3S 0.00000 0.00000 0.00000 0.00000 0.00000

198 4PX 0.00000 0.00000 0.00000 0.00000 0.00000

199 4PY 0.00000 0.00000 0.00000 0.00000 0.00000

200 4PZ 0.00000 0.00000 0.00000 0.00000 0.00000

66 67 68 69 70

66 2PY 0.08293

67 2PZ 0.00000 0.07508

68 3S 0.00000 0.00000 0.45587

69 3PX 0.00000 0.00000 0.00000 0.18179

70 3PY 0.07661 0.00000 0.00000 0.00000 0.17943

71 3PZ 0.00000 0.07318 0.00000 0.00000 0.00000

72 4S 0.00000 0.00000 0.20958 0.00000 0.00000

73 4PX 0.00000 0.00000 0.00000 0.05236 0.00000

74 4PY 0.00834 0.00000 0.00000 0.00000 0.03500

75 4PZ 0.00000 0.02241 0.00000 0.00000 0.00000

76 5D 0 0.00000 0.00000 0.00000 0.00000 0.00000

77 5D+1 0.00000 0.00000 0.00000 0.00000 0.00000

78 5D-1 0.00000 0.00000 0.00000 0.00000 0.00000

79 5D+2 0.00000 0.00000 0.00000 0.00000 0.00000

80 5D-2 0.00000 0.00000 0.00000 0.00000 0.00000

81 5 C 1S -0.00004 0.00000 -0.00037 -0.00007 -0.00165

82 2S -0.00003 0.00000 -0.00061 -0.00011 -0.00284

83 2PX 0.00006 0.00000 0.00010 0.00040 0.00057

84 2PY 0.00198 0.00016 0.00604 0.00062 0.01548

85 2PZ 0.00012 0.00009 0.00038 0.00001 0.00107

86 3S 0.00661 0.00017 0.01546 0.00155 0.04014

87 3PX 0.00053 0.00005 0.00067 0.00259 0.00192

88 3PY 0.01523 0.00140 0.03532 0.00207 0.04927

89 3PZ 0.00111 0.00235 0.00225 0.00005 0.00408

90 4S 0.00547 -0.00009 -0.00100 -0.00015 0.02968

91 4PX 0.00055 0.00000 -0.00226 0.00893 0.00243

92 4PY 0.00499 0.00105 0.02346 0.00114 0.01590

93 4PZ 0.00064 0.00528 0.00227 -0.00009 0.00262

94 5D 0 0.00013 0.00016 0.00050 0.00002 0.00023

95 5D+1 0.00000 0.00000 0.00000 0.00007 0.00002

96 5D-1 0.00026 0.00045 0.00065 0.00002 0.00057

97 5D+2 0.00077 0.00012 0.00291 0.00069 0.00115

98 5D-2 0.00014 0.00000 0.00057 0.00186 0.00031

99 6 C 1S -0.00001 0.00000 -0.00038 -0.00120 -0.00031

100 2S 0.00000 0.00000 -0.00062 -0.00208 -0.00053

101 2PX 0.00037 0.00020 0.00473 0.00799 0.00336

102 2PY 0.00002 -0.00001 0.00139 0.00349 -0.00004

103 2PZ 0.00004 0.00002 0.00094 0.00213 0.00029

104 3S 0.00133 0.00022 0.01619 0.02964 0.00784

105 3PX 0.00321 0.00194 0.02765 0.02401 0.01251

106 3PY -0.00004 -0.00014 0.00810 0.01308 -0.00106

107 3PZ 0.00030 0.00097 0.00546 0.00836 0.00103

108 4S 0.00055 -0.00060 0.00489 0.01671 0.00282

109 4PX 0.00101 0.00055 0.00798 0.00255 0.00443

110 4PY -0.00027 -0.00027 0.00546 0.00820 -0.00118

111 4PZ 0.00017 0.00220 0.00575 0.00355 0.00065

112 5D 0 -0.00001 0.00028 0.00033 0.00003 -0.00002

113 5D+1 0.00016 0.00026 0.00075 0.00069 0.00042

114 5D-1 0.00000 0.00004 0.00032 0.00051 0.00000

115 5D+2 0.00071 0.00005 0.00122 0.00003 0.00221

116 5D-2 -0.00002 0.00019 0.00191 0.00173 0.00025

117 7 C 1S 0.00000 0.00000 0.00000 0.00000 0.00000

118 2S 0.00000 0.00000 0.00000 0.00000 0.00000

119 2PX 0.00000 0.00000 -0.00001 -0.00002 0.00000

120 2PY 0.00000 0.00000 0.00000 0.00000 0.00000

121 2PZ 0.00000 0.00000 0.00000 -0.00001 0.00000

122 3S 0.00000 0.00000 -0.00014 -0.00035 0.00001

123 3PX 0.00000 -0.00001 -0.00032 -0.00028 0.00001

124 3PY 0.00000 0.00000 0.00000 -0.00003 -0.00002

125 3PZ 0.00000 0.00000 -0.00010 -0.00034 0.00001

126 4S -0.00001 0.00001 -0.00155 -0.00363 -0.00007

127 4PX 0.00000 -0.00028 -0.00371 -0.00280 -0.00002

128 4PY 0.00004 -0.00002 0.00009 0.00000 0.00019

129 4PZ 0.00000 0.00001 -0.00067 -0.00151 0.00001

130 5D 0 0.00000 0.00000 -0.00001 -0.00001 0.00000

131 5D+1 0.00000 0.00000 0.00004 0.00014 0.00000

132 5D-1 0.00000 0.00000 0.00000 0.00000 -0.00001

133 5D+2 0.00000 0.00000 0.00005 0.00017 -0.00001

134 5D-2 0.00000 0.00000 0.00000 -0.00001 -0.00001

135 8 C 1S 0.00000 0.00000 0.00000 0.00000 0.00000

136 2S 0.00000 0.00000 0.00000 0.00000 0.00000

137 2PX 0.00000 0.00000 0.00000 0.00000 0.00000

138 2PY 0.00000 0.00000 0.00000 0.00000 0.00000

139 2PZ 0.00000 0.00000 0.00000 0.00000 0.00000

140 3S 0.00000 0.00000 -0.00002 -0.00004 -0.00003

141 3PX 0.00000 0.00000 -0.00002 -0.00002 -0.00006

142 3PY 0.00000 0.00000 -0.00006 -0.00009 -0.00001

143 3PZ 0.00000 0.00000 0.00000 0.00000 0.00000

144 4S 0.00000 0.00000 -0.00072 -0.00061 -0.00002

145 4PX -0.00006 0.00000 -0.00080 0.00022 -0.00034

146 4PY 0.00001 -0.00001 -0.00173 -0.00126 0.00000

147 4PZ 0.00000 -0.00021 0.00001 0.00000 0.00001

148 5D 0 0.00000 0.00000 0.00000 0.00000 0.00000

149 5D+1 0.00000 0.00000 0.00000 0.00000 0.00000

150 5D-1 0.00000 0.00000 0.00000 0.00000 0.00000

151 5D+2 0.00000 0.00000 0.00000 0.00000 0.00000

152 5D-2 0.00000 0.00000 0.00000 0.00001 -0.00001

153 9 C 1S 0.00000 0.00000 0.00000 0.00000 0.00000

154 2S 0.00000 0.00000 0.00000 0.00000 0.00000

155 2PX 0.00000 0.00000 0.00000 0.00000 -0.00001

156 2PY 0.00000 0.00000 -0.00001 0.00000 -0.00004

157 2PZ 0.00000 0.00000 0.00000 0.00000 0.00000

158 3S -0.00001 0.00000 -0.00015 0.00000 -0.00041

159 3PX -0.00001 0.00000 -0.00010 0.00000 -0.00023

160 3PY -0.00004 0.00000 -0.00037 0.00000 -0.00081

161 3PZ 0.00000 0.00000 -0.00001 -0.00001 -0.00001

162 4S -0.00027 0.00004 0.00077 -0.00115 -0.00189

163 4PX 0.00007 0.00002 -0.00038 0.00023 0.00030

164 4PY -0.00089 -0.00005 -0.00179 -0.00072 -0.00476

165 4PZ -0.00002 -0.00025 -0.00032 -0.00006 -0.00015

166 5D 0 0.00000 0.00000 0.00001 0.00000 0.00002

167 5D+1 0.00000 0.00000 0.00000 0.00000 0.00001

168 5D-1 0.00000 0.00000 0.00000 0.00000 0.00002

169 5D+2 0.00001 0.00000 0.00005 -0.00001 0.00025

170 5D-2 0.00000 0.00000 0.00002 0.00001 0.00004

171 10 H 1S 0.00000 0.00000 0.00000 0.00000 0.00000

172 2S 0.00000 0.00000 0.00002 0.00009 0.00001

173 3S 0.00001 0.00000 0.00057 0.00120 0.00006

174 4PX 0.00000 0.00000 0.00000 0.00000 0.00000

175 4PY 0.00000 0.00000 0.00000 0.00000 0.00000

176 4PZ 0.00000 0.00000 0.00000 0.00000 0.00000

177 11 H 1S 0.00000 0.00000 -0.00004 -0.00008 -0.00007

178 2S -0.00011 0.00000 -0.00102 -0.00106 -0.00115

179 3S -0.00062 0.00002 -0.00447 -0.00107 -0.00366

180 4PX 0.00000 0.00000 0.00003 0.00006 0.00007

181 4PY 0.00000 0.00000 0.00001 0.00001 0.00005

182 4PZ 0.00000 0.00000 0.00000 0.00001 0.00000

183 12 H 1S 0.00000 0.00000 0.00000 0.00000 0.00000

184 2S 0.00000 0.00000 0.00001 0.00002 0.00000

185 3S 0.00000 0.00001 0.00023 0.00036 0.00000

186 4PX 0.00000 0.00000 0.00000 0.00000 0.00000

187 4PY 0.00000 0.00000 0.00000 0.00000 0.00000

188 4PZ 0.00000 0.00000 0.00000 0.00000 0.00000

189 13 H 1S 0.00000 0.00000 0.00000 0.00000 0.00000

190 2S 0.00000 0.00000 0.00000 0.00000 0.00000

191 3S 0.00000 0.00000 0.00003 0.00009 0.00002

192 4PX 0.00000 0.00000 0.00000 0.00000 0.00000

193 4PY 0.00000 0.00000 0.00000 0.00000 0.00000

194 4PZ 0.00000 0.00000 0.00000 0.00000 0.00000

195 14 H 1S 0.00000 0.00000 0.00000 0.00000 0.00000

196 2S 0.00000 0.00000 0.00000 0.00000 0.00001

197 3S 0.00008 0.00001 0.00011 -0.00001 0.00058

198 4PX 0.00000 0.00000 0.00000 0.00000 0.00000

199 4PY 0.00000 0.00000 0.00000 0.00000 0.00000

200 4PZ 0.00000 0.00000 0.00000 0.00000 0.00000

71 72 73 74 75

71 3PZ 0.18036

72 4S 0.00000 0.26718

73 4PX 0.00000 0.00000 0.06195

74 4PY 0.00000 0.00000 0.00000 0.02910

75 4PZ 0.10287 0.00000 0.00000 0.00000 0.14713

76 5D 0 0.00000 0.00000 0.00000 0.00000 0.00000

77 5D+1 0.00000 0.00000 0.00000 0.00000 0.00000

78 5D-1 0.00000 0.00000 0.00000 0.00000 0.00000

79 5D+2 0.00000 0.00000 0.00000 0.00000 0.00000

80 5D-2 0.00000 0.00000 0.00000 0.00000 0.00000

81 5 C 1S -0.00004 0.00016 0.00002 -0.00012 -0.00003

82 2S -0.00007 0.00014 0.00001 -0.00056 -0.00005

83 2PX 0.00004 0.00025 -0.00055 0.00031 0.00003

84 2PY 0.00150 0.00111 0.00013 0.00204 0.00073

85 2PZ 0.00242 0.00005 0.00002 0.00040 0.00446

86 3S 0.00099 0.00042 0.00020 0.01014 0.00064

87 3PX 0.00017 0.00142 -0.00293 0.00149 0.00015

88 3PY 0.00552 0.00637 0.00051 0.00640 0.00295

89 3PZ 0.01606 0.00030 0.00008 0.00175 0.02377

90 4S -0.00047 -0.03389 -0.00043 -0.00259 -0.00239

91 4PX 0.00002 -0.00126 -0.00471 0.00117 -0.00006

92 4PY 0.00463 -0.02403 -0.00018 -0.00051 0.00443

93 4PZ 0.02909 -0.00106 0.00000 0.00156 0.05182

94 5D 0 0.00054 0.00001 0.00001 0.00000 0.00027

95 5D+1 0.00000 -0.00001 0.00004 0.00000 0.00001

96 5D-1 0.00227 0.00018 0.00001 0.00004 0.00173

97 5D+2 0.00031 0.00098 0.00026 -0.00025 0.00010

98 5D-2 -0.00002 0.00011 0.00064 0.00000 -0.00001

99 6 C 1S -0.00006 -0.00004 -0.00086 -0.00024 0.00007

100 2S -0.00009 -0.00022 -0.00172 -0.00045 0.00009

101 2PX 0.00188 0.00227 0.00126 0.00071 0.00094

102 2PY -0.00013 0.00115 0.00125 -0.00026 -0.00018

103 2PZ 0.00102 0.00105 0.00117 0.00010 0.00162

104 3S 0.00122 0.00565 0.02066 0.00499 -0.00067

105 3PX 0.00763 0.01136 0.00297 0.00289 0.00426

106 3PY -0.00062 0.00580 0.00526 -0.00145 -0.00083

107 3PZ 0.00764 0.00538 0.00502 0.00042 0.00892

108 4S -0.00321 -0.01602 0.00661 0.00137 -0.00796

109 4PX 0.00247 -0.01013 -0.00141 0.00116 0.00141

110 4PY -0.00124 0.00501 0.00454 -0.00333 -0.00173

111 4PZ 0.01246 0.00962 0.00460 0.00044 0.01495

112 5D 0 0.00097 0.00005 -0.00002 0.00000 0.00053

113 5D+1 0.00155 0.00026 0.00003 0.00005 0.00134

114 5D-1 0.00024 0.00013 0.00012 -0.00002 0.00011

115 5D+2 0.00014 0.00069 -0.00027 0.00041 0.00007

116 5D-2 0.00048 0.00032 0.00011 0.00003 0.00011

117 7 C 1S 0.00000 0.00004 0.00004 0.00000 0.00004

118 2S 0.00000 0.00007 0.00011 -0.00001 0.00006

119 2PX -0.00001 -0.00070 -0.00088 0.00002 -0.00058

120 2PY 0.00000 0.00003 0.00001 0.00007 -0.00001

121 2PZ 0.00000 -0.00001 -0.00020 0.00001 0.00001

122 3S -0.00009 -0.00134 -0.00270 0.00007 -0.00117

123 3PX -0.00014 -0.00466 -0.00449 0.00008 -0.00310

124 3PY -0.00002 0.00020 0.00008 0.00049 -0.00007

125 3PZ 0.00001 0.00000 -0.00106 0.00004 -0.00008

126 4S 0.00010 -0.00329 -0.00772 0.00020 0.00053

127 4PX -0.00154 -0.01459 -0.00858 0.00021 -0.00799

128 4PY -0.00010 0.00101 0.00046 0.00222 -0.00014

129 4PZ -0.00015 0.00138 -0.00050 0.00001 -0.00314

130 5D 0 0.00003 -0.00005 -0.00001 0.00000 0.00004

131 5D+1 -0.00001 0.00016 0.00026 -0.00001 0.00000

132 5D-1 0.00000 0.00000 0.00000 0.00000 0.00000

133 5D+2 0.00006 0.00014 0.00018 -0.00001 0.00011

134 5D-2 0.00000 -0.00003 -0.00003 -0.00003 0.00000

135 8 C 1S 0.00000 0.00002 0.00004 0.00002 0.00000

136 2S 0.00000 0.00004 0.00005 0.00003 0.00000

137 2PX 0.00000 -0.00007 0.00014 -0.00011 0.00000

138 2PY 0.00000 -0.00015 -0.00016 0.00008 -0.00001

139 2PZ 0.00000 0.00000 0.00000 0.00000 -0.00018

140 3S 0.00000 -0.00088 -0.00057 -0.00044 -0.00001

141 3PX 0.00000 -0.00051 0.00083 -0.00069 -0.00003

142 3PY 0.00000 -0.00125 -0.00111 0.00051 -0.00005

143 3PZ -0.00005 0.00000 -0.00001 0.00000 -0.00144

144 4S 0.00000 -0.00530 -0.00220 0.00022 -0.00001

145 4PX -0.00003 -0.00280 0.00167 -0.00127 -0.00010

146 4PY -0.00006 -0.00571 -0.00356 0.00114 -0.00020

147 4PZ -0.00174 0.00000 -0.00002 0.00000 -0.00858

148 5D 0 0.00000 0.00001 0.00002 0.00000 0.00000

149 5D+1 0.00000 0.00000 0.00000 0.00000 -0.00006

150 5D-1 0.00000 0.00000 0.00000 0.00000 0.00002

151 5D+2 0.00000 0.00000 -0.00001 -0.00003 0.00000

152 5D-2 0.00000 -0.00002 -0.00001 -0.00003 0.00000

153 9 C 1S 0.00000 -0.00003 0.00004 0.00001 0.00000

154 2S 0.00000 -0.00005 0.00005 0.00002 -0.00001

155 2PX 0.00000 0.00002 0.00004 0.00009 0.00001

156 2PY 0.00000 -0.00028 0.00010 -0.00065 -0.00004

157 2PZ 0.00000 -0.00004 0.00001 0.00002 -0.00016

158 3S 0.00000 0.00122 -0.00048 -0.00054 0.00011

159 3PX 0.00000 0.00020 0.00033 0.00050 0.00004

160 3PY -0.00005 -0.00155 0.00054 -0.00353 -0.00022

161 3PZ -0.00005 -0.00028 0.00003 0.00014 -0.00107

162 4S 0.00026 0.00518 -0.00063 0.00238 0.00114

163 4PX 0.00014 -0.00176 0.00105 0.00248 0.00038

164 4PY -0.00033 0.00027 0.00048 -0.00508 -0.00066

165 4PZ -0.00185 -0.00114 -0.00005 0.00042 -0.00763

166 5D 0 -0.00001 0.00002 0.00000 0.00001 -0.00002

167 5D+1 0.00002 0.00000 0.00000 0.00001 0.00014

168 5D-1 0.00002 0.00000 0.00000 0.00003 0.00008

169 5D+2 0.00003 0.00004 -0.00013 0.00026 0.00008

170 5D-2 -0.00002 0.00012 -0.00001 -0.00004 -0.00005

171 10 H 1S 0.00000 0.00024 0.00028 0.00000 0.00001

172 2S 0.00000 0.00197 0.00165 0.00001 0.00009

173 3S 0.00004 0.00497 0.00328 -0.00001 0.00028

174 4PX 0.00000 0.00004 -0.00001 0.00000 0.00000

175 4PY 0.00000 0.00000 0.00001 0.00000 0.00000

176 4PZ 0.00000 0.00000 0.00000 0.00000 -0.00001

177 11 H 1S 0.00000 -0.00145 -0.00096 -0.00039 0.00004

178 2S 0.00003 -0.00720 -0.00378 -0.00189 0.00018

179 3S 0.00013 -0.01514 -0.00420 -0.00290 0.00034

180 4PX 0.00001 0.00003 0.00007 0.00010 0.00002

181 4PY -0.00001 -0.00001 0.00003 0.00006 -0.00002

182 4PZ 0.00001 0.00003 0.00003 0.00000 0.00009

183 12 H 1S 0.00000 0.00004 0.00011 0.00000 0.00000

184 2S 0.00000 0.00039 0.00093 0.00001 0.00012

185 3S 0.00008 0.00137 0.00205 0.00002 0.00059

186 4PX 0.00000 -0.00001 -0.00001 0.00000 -0.00002

187 4PY 0.00000 0.00000 0.00000 0.00000 0.00000

188 4PZ 0.00000 0.00001 0.00001 0.00000 0.00001

189 13 H 1S 0.00000 0.00000 0.00000 0.00000 0.00000

190 2S 0.00000 0.00006 -0.00004 -0.00001 0.00003

191 3S 0.00003 0.00040 0.00008 -0.00007 0.00020

192 4PX 0.00000 0.00000 0.00000 0.00000 0.00000

193 4PY 0.00000 0.00000 0.00000 0.00000 0.00000

194 4PZ 0.00000 0.00000 0.00000 0.00000 0.00000

195 14 H 1S 0.00000 0.00002 -0.00001 0.00011 0.00001

196 2S 0.00000 0.00022 -0.00009 0.00080 0.00010

197 3S 0.00006 -0.00024 -0.00026 0.00224 0.00029

198 4PX 0.00000 0.00000 0.00000 0.00000 0.00000

199 4PY 0.00000 0.00002 0.00000 0.00000 0.00000

200 4PZ 0.00000 0.00000 0.00000 0.00000 0.00000

76 77 78 79 80

76 5D 0 0.00084

77 5D+1 0.00000 0.00186

78 5D-1 0.00000 0.00000 0.00155

79 5D+2 0.00000 0.00000 0.00000 0.00345

80 5D-2 0.00000 0.00000 0.00000 0.00000 0.00301

81 5 C 1S -0.00005 0.00000 -0.00005 -0.00029 -0.00003

82 2S -0.00007 0.00000 -0.00008 -0.00046 -0.00004

83 2PX 0.00000 0.00001 0.00003 0.00009 0.00042

84 2PY 0.00003 0.00003 0.00044 0.00099 0.00031

85 2PZ 0.00015 0.00002 0.00049 0.00012 0.00004

86 3S 0.00047 0.00004 0.00071 0.00394 0.00033

87 3PX 0.00000 0.00004 0.00006 0.00014 0.00201

88 3PY 0.00005 0.00007 0.00096 0.00156 0.00064

89 3PZ 0.00052 0.00009 0.00248 0.00029 0.00010

90 4S 0.00002 0.00001 0.00023 0.00160 0.00020

91 4PX 0.00001 0.00006 0.00003 0.00025 0.00129

92 4PY 0.00001 0.00002 0.00013 -0.00033 0.00012

93 4PZ 0.00023 0.00008 0.00220 0.00010 0.00004

94 5D 0 0.00001 0.00000 0.00003 0.00000 0.00000

95 5D+1 0.00000 0.00001 -0.00001 0.00001 -0.00002

96 5D-1 0.00007 0.00001 -0.00011 -0.00001 0.00000

97 5D+2 0.00000 0.00000 0.00000 0.00000 -0.00009

98 5D-2 0.00000 0.00003 0.00000 0.00003 -0.00031

99 6 C 1S -0.00003 -0.00006 -0.00002 -0.00009 -0.00014

100 2S -0.00004 -0.00009 -0.00003 -0.00015 -0.00022

101 2PX -0.00003 0.00038 0.00019 0.00004 0.00087

102 2PY 0.00000 0.00014 0.00000 0.00083 -0.00003

103 2PZ 0.00023 0.00021 0.00005 0.00010 0.00013

104 3S 0.00028 0.00076 0.00022 0.00128 0.00185

105 3PX -0.00005 0.00080 0.00046 0.00003 0.00182

106 3PY 0.00001 0.00034 0.00003 0.00250 0.00037

107 3PZ 0.00078 0.00122 0.00028 0.00027 0.00031

108 4S 0.00006 0.00033 0.00007 0.00024 0.00060

109 4PX 0.00005 0.00011 0.00012 0.00007 0.00010

110 4PY 0.00000 0.00007 0.00006 0.00069 0.00039

111 4PZ 0.00034 0.00108 0.00032 0.00012 0.00000

112 5D 0 0.00000 -0.00008 -0.00003 0.00000 0.00000

113 5D+1 0.00007 -0.00007 -0.00003 0.00003 -0.00002

114 5D-1 0.00003 0.00006 0.00000 0.00001 0.00000

115 5D+2 0.00000 -0.00002 -0.00003 -0.00020 -0.00021

116 5D-2 0.00000 0.00000 0.00001 -0.00015 -0.00006

117 7 C 1S 0.00000 0.00000 0.00000 0.00000 0.00000

118 2S 0.00000 0.00000 0.00000 0.00000 0.00000

119 2PX 0.00000 0.00001 0.00000 0.00000 0.00000

120 2PY 0.00000 0.00000 0.00000 0.00000 0.00000

121 2PZ 0.00000 0.00000 0.00000 0.00000 0.00000

122 3S 0.00000 0.00003 0.00000 0.00006 0.00001

123 3PX -0.00002 0.00031 -0.00002 0.00007 0.00000

124 3PY 0.00000 -0.00001 -0.00001 0.00001 -0.00004

125 3PZ 0.00002 -0.00005 0.00000 0.00009 0.00001

126 4S 0.00000 0.00009 -0.00001 0.00012 0.00003

127 4PX -0.00001 0.00069 -0.00005 0.00002 0.00000

128 4PY 0.00000 -0.00003 -0.00011 0.00005 -0.00026

129 4PZ 0.00006 0.00002 0.00000 0.00018 0.00004

130 5D 0 0.00000 0.00001 0.00000 0.00000 0.00000

131 5D+1 0.00000 0.00000 0.00000 0.00002 0.00000

132 5D-1 0.00000 0.00000 0.00000 0.00000 0.00000

133 5D+2 0.00000 0.00001 0.00000 0.00001 0.00000

134 5D-2 0.00000 0.00000 0.00000 0.00000 0.00000

135 8 C 1S 0.00000 0.00000 0.00000 0.00000 0.00000

136 2S 0.00000 0.00000 0.00000 0.00000 0.00000

137 2PX 0.00000 0.00000 0.00000 0.00000 0.00000

138 2PY 0.00000 0.00000 0.00000 0.00000 0.00000

139 2PZ 0.00000 0.00000 0.00000 0.00000 0.00000

140 3S 0.00000 0.00000 0.00000 0.00000 0.00000

141 3PX 0.00000 0.00000 0.00000 0.00000 0.00000

142 3PY 0.00000 0.00000 0.00000 0.00000 0.00000

143 3PZ 0.00000 0.00000 0.00000 0.00000 0.00000

144 4S 0.00001 0.00000 0.00000 -0.00001 -0.00011

145 4PX 0.00000 0.00000 0.00000 0.00000 0.00001

146 4PY 0.00000 0.00000 0.00000 -0.00007 0.00000

147 4PZ 0.00000 0.00002 -0.00004 0.00000 0.00000

148 5D 0 0.00000 0.00000 0.00000 0.00000 0.00000

149 5D+1 0.00000 0.00000 0.00000 0.00000 0.00000

150 5D-1 0.00000 0.00000 0.00000 0.00000 0.00000

151 5D+2 0.00000 0.00000 0.00000 0.00000 0.00000

152 5D-2 0.00000 0.00000 0.00000 0.00000 0.00000

153 9 C 1S 0.00000 0.00000 0.00000 0.00000 0.00000

154 2S 0.00000 0.00000 0.00000 0.00000 0.00000

155 2PX 0.00000 0.00000 0.00000 0.00000 0.00000

156 2PY 0.00000 0.00000 0.00000 0.00000 0.00001

157 2PZ 0.00000 0.00000 0.00000 0.00000 0.00000

158 3S 0.00001 0.00000 0.00000 0.00000 0.00006

159 3PX 0.00000 0.00000 -0.00001 0.00001 0.00001

160 3PY 0.00003 0.00000 0.00001 0.00004 0.00015

161 3PZ 0.00000 0.00000 0.00005 0.00001 -0.00001

162 4S 0.00003 -0.00001 0.00000 -0.00016 0.00007

163 4PX 0.00001 0.00000 -0.00002 -0.00002 -0.00001

164 4PY 0.00004 0.00000 0.00000 -0.00007 0.00022

165 4PZ 0.00000 -0.00003 0.00026 0.00002 -0.00001

166 5D 0 0.00000 0.00000 0.00000 0.00000 0.00000

167 5D+1 0.00000 0.00000 0.00000 0.00000 0.00000

168 5D-1 0.00000 0.00000 0.00001 0.00000 0.00000

169 5D+2 0.00000 0.00000 0.00000 0.00001 0.00000

170 5D-2 0.00000 0.00000 0.00000 0.00001 0.00000

171 10 H 1S 0.00000 0.00000 0.00000 0.00000 0.00000

172 2S 0.00000 0.00000 0.00000 0.00000 0.00000

173 3S 0.00000 0.00000 0.00000 0.00001 0.00000

174 4PX 0.00000 0.00000 0.00000 0.00000 0.00000

175 4PY 0.00000 0.00000 0.00000 0.00000 0.00000

176 4PZ 0.00000 0.00000 0.00000 0.00000 0.00000

177 11 H 1S 0.00000 0.00000 0.00000 0.00001 0.00004

178 2S 0.00003 -0.00001 0.00001 0.00007 0.00043

179 3S -0.00002 0.00000 0.00000 0.00006 0.00013

180 4PX 0.00000 0.00000 0.00000 0.00000 0.00001

181 4PY 0.00001 0.00000 0.00000 0.00000 0.00002

182 4PZ 0.00000 0.00000 0.00000 0.00000 0.00000

183 12 H 1S 0.00000 0.00000 0.00000 0.00000 0.00000

184 2S 0.00000 0.00000 0.00000 0.00000 0.00000

185 3S 0.00000 -0.00002 0.00000 0.00000 0.00000

186 4PX 0.00000 0.00000 0.00000 0.00000 0.00000

187 4PY 0.00000 0.00000 0.00000 0.00000 0.00000

188 4PZ 0.00000 0.00000 0.00000 0.00000 0.00000

189 13 H 1S 0.00000 0.00000 0.00000 0.00000 0.00000

190 2S 0.00000 0.00000 0.00000 0.00000 0.00000

191 3S 0.00000 0.00000 0.00000 0.00000 0.00001

192 4PX 0.00000 0.00000 0.00000 0.00000 0.00000

193 4PY 0.00000 0.00000 0.00000 0.00000 0.00000

194 4PZ 0.00000 0.00000 0.00000 0.00000 0.00000

195 14 H 1S 0.00000 0.00000 0.00000 0.00000 0.00000

196 2S 0.00000 0.00000 0.00000 0.00000 0.00000

197 3S 0.00000 0.00000 0.00000 0.00002 -0.00001

198 4PX 0.00000 0.00000 0.00000 0.00000 0.00000

199 4PY 0.00000 0.00000 0.00000 0.00000 0.00000

200 4PZ 0.00000 0.00000 0.00000 0.00000 0.00000

81 82 83 84 85

81 5 C 1S 0.65858

82 2S 0.49240 0.49017

83 2PX 0.00000 0.00000 0.06928

84 2PY 0.00000 0.00000 0.00000 0.09058

85 2PZ 0.00000 0.00000 0.00000 0.00000 0.07931

86 3S -0.02813 -0.06210 0.00000 0.00000 0.00000

87 3PX 0.00000 0.00000 0.06723 0.00000 0.00000

88 3PY 0.00000 0.00000 0.00000 0.08118 0.00000

89 3PZ 0.00000 0.00000 0.00000 0.00000 0.07659

90 4S -0.01372 -0.02418 0.00000 0.00000 0.00000

91 4PX 0.00000 0.00000 0.01674 0.00000 0.00000

92 4PY 0.00000 0.00000 0.00000 0.02226 0.00000

93 4PZ 0.00000 0.00000 0.00000 0.00000 0.02695

94 5D 0 0.00000 0.00000 0.00000 0.00000 0.00000

95 5D+1 0.00000 0.00000 0.00000 0.00000 0.00000

96 5D-1 0.00000 0.00000 0.00000 0.00000 0.00000

97 5D+2 0.00000 0.00000 0.00000 0.00000 0.00000

98 5D-2 0.00000 0.00000 0.00000 0.00000 0.00000

99 6 C 1S 0.00000 0.00000 0.00000 0.00000 0.00000

100 2S 0.00000 0.00000 0.00000 0.00000 0.00000

101 2PX 0.00000 0.00000 0.00000 0.00000 0.00000

102 2PY 0.00000 0.00000 0.00000 0.00000 0.00000

103 2PZ 0.00000 0.00000 0.00000 0.00000 0.00000

104 3S 0.00000 0.00000 0.00000 -0.00001 0.00000

105 3PX 0.00000 0.00000 0.00000 -0.00001 0.00000

106 3PY 0.00000 0.00000 0.00000 -0.00002 0.00000

107 3PZ 0.00000 0.00000 0.00000 0.00000 0.00000

108 4S 0.00005 0.00010 -0.00006 -0.00035 -0.00002

109 4PX 0.00009 0.00015 -0.00007 -0.00035 -0.00002

110 4PY 0.00008 0.00016 0.00006 -0.00026 0.00001

111 4PZ 0.00000 0.00000 0.00000 -0.00001 -0.00025

112 5D 0 0.00000 0.00000 0.00000 0.00000 0.00000

113 5D+1 0.00000 0.00000 0.00000 0.00000 0.00000

114 5D-1 0.00000 0.00000 0.00000 0.00000 0.00000

115 5D+2 0.00000 0.00000 0.00000 0.00000 0.00000

116 5D-2 0.00000 0.00000 0.00000 0.00001 0.00000

117 7 C 1S 0.00000 0.00000 0.00000 0.00000 0.00000

118 2S 0.00000 0.00000 0.00000 0.00000 0.00000

119 2PX 0.00000 0.00000 0.00000 0.00000 0.00000

120 2PY 0.00000 0.00000 0.00000 0.00000 0.00000

121 2PZ 0.00000 0.00000 0.00000 0.00000 0.00000

122 3S 0.00000 0.00000 0.00000 0.00000 0.00000

123 3PX 0.00000 0.00000 0.00000 0.00000 0.00000

124 3PY 0.00000 0.00000 0.00000 0.00000 0.00000

125 3PZ 0.00000 0.00000 0.00000 0.00000 0.00000

126 4S 0.00002 0.00003 -0.00014 -0.00003 -0.00004

127 4PX 0.00002 0.00003 -0.00012 -0.00019 -0.00030

128 4PY 0.00001 0.00002 -0.00014 0.00001 -0.00011

129 4PZ 0.00001 0.00001 -0.00001 0.00004 -0.00006

130 5D 0 0.00000 0.00000 0.00000 0.00000 0.00000

131 5D+1 0.00000 0.00000 0.00000 0.00000 0.00000

132 5D-1 0.00000 0.00000 0.00000 0.00000 0.00000

133 5D+2 0.00000 0.00000 0.00000 0.00000 0.00000

134 5D-2 0.00000 0.00000 0.00000 0.00000 0.00000

135 8 C 1S 0.00000 0.00000 0.00000 0.00000 0.00000

136 2S 0.00000 0.00000 0.00000 0.00000 0.00000

137 2PX 0.00000 0.00000 0.00000 0.00000 0.00000

138 2PY 0.00000 0.00000 0.00000 0.00000 0.00000

139 2PZ 0.00000 0.00000 0.00000 0.00000 0.00000

140 3S 0.00000 0.00000 -0.00001 0.00000 0.00000

141 3PX 0.00000 0.00000 -0.00002 -0.00001 0.00000

142 3PY 0.00000 0.00000 0.00000 0.00000 0.00000

143 3PZ 0.00000 0.00000 0.00000 0.00000 0.00000

144 4S 0.00002 0.00004 -0.00059 -0.00004 0.00003

145 4PX 0.00010 0.00020 -0.00065 -0.00015 -0.00007

146 4PY 0.00001 0.00002 -0.00002 0.00008 -0.00001

147 4PZ -0.00001 -0.00001 -0.00003 0.00001 -0.00026

148 5D 0 0.00000 0.00000 0.00000 0.00000 0.00000

149 5D+1 0.00000 0.00000 0.00000 0.00000 0.00000

150 5D-1 0.00000 0.00000 0.00000 0.00000 0.00000

151 5D+2 0.00000 0.00000 0.00001 0.00000 0.00000

152 5D-2 0.00000 0.00000 0.00000 0.00000 0.00000

153 9 C 1S 0.00000 0.00000 -0.00002 -0.00002 0.00000

154 2S 0.00000 0.00000 -0.00001 -0.00001 0.00000

155 2PX -0.00002 -0.00002 0.00052 0.00072 0.00000

156 2PY -0.00002 -0.00001 0.00065 0.00036 0.00000

157 2PZ 0.00000 0.00000 0.00000 0.00000 0.00012

158 3S -0.00036 -0.00059 0.00368 0.00324 -0.00001

159 3PX -0.00097 -0.00167 0.00363 0.00639 0.00002

160 3PY -0.00074 -0.00127 0.00558 0.00230 -0.00002

161 3PZ 0.00000 0.00000 0.00003 0.00000 0.00233

162 4S -0.00006 -0.00021 0.00167 0.00088 -0.00001

163 4PX -0.00051 -0.00104 0.00009 0.00102 0.00002

164 4PY -0.00037 -0.00084 0.00211 -0.00042 -0.00001

165 4PZ -0.00001 -0.00001 0.00002 0.00000 0.00288

166 5D 0 -0.00006 -0.00010 0.00013 0.00010 0.00000

167 5D+1 0.00000 0.00000 0.00000 0.00000 0.00050

168 5D-1 0.00000 0.00000 0.00000 0.00000 0.00030

169 5D+2 0.00000 -0.00001 0.00016 0.00062 0.00000

170 5D-2 -0.00031 -0.00047 0.00083 0.00056 0.00000

171 10 H 1S 0.00000 0.00000 0.00000 0.00000 0.00000

172 2S 0.00000 0.00000 0.00000 0.00000 0.00000

173 3S 0.00000 0.00000 0.00008 0.00000 0.00000

174 4PX 0.00000 0.00000 0.00000 0.00000 0.00000

175 4PY 0.00000 0.00000 0.00000 0.00000 0.00000

176 4PZ 0.00000 0.00000 0.00000 0.00000 0.00000

177 11 H 1S 0.00000 0.00000 0.00000 0.00000 0.00000

178 2S 0.00000 0.00000 0.00000 0.00000 0.00000

179 3S -0.00001 -0.00001 0.00001 0.00008 0.00000

180 4PX 0.00000 0.00000 0.00000 0.00000 0.00000

181 4PY 0.00000 0.00000 0.00000 0.00000 0.00000

182 4PZ 0.00000 0.00000 0.00000 0.00000 0.00000

183 12 H 1S 0.00000 0.00000 0.00000 0.00000 0.00000

184 2S 0.00000 0.00000 0.00000 0.00000 0.00000

185 3S 0.00000 0.00000 0.00001 0.00000 0.00001

186 4PX 0.00000 0.00000 0.00000 0.00000 0.00000

187 4PY 0.00000 0.00000 0.00000 0.00000 0.00000

188 4PZ 0.00000 0.00000 0.00000 0.00000 0.00000

189 13 H 1S 0.00000 0.00000 0.00000 0.00000 0.00000

190 2S 0.00000 0.00000 0.00000 0.00000 0.00000

191 3S -0.00001 -0.00001 0.00005 0.00001 0.00000

192 4PX 0.00000 0.00000 0.00000 0.00000 0.00000

193 4PY 0.00000 0.00000 0.00000 0.00000 0.00000

194 4PZ 0.00000 0.00000 0.00000 0.00000 0.00000

195 14 H 1S 0.00000 0.00000 0.00000 0.00000 0.00000

196 2S 0.00001 0.00001 -0.00005 -0.00018 0.00000

197 3S 0.00011 0.00018 -0.00008 -0.00072 -0.00002

198 4PX 0.00000 0.00000 0.00000 0.00000 0.00000

199 4PY 0.00000 0.00000 0.00000 0.00000 0.00000

200 4PZ 0.00000 0.00000 0.00000 0.00000 0.00000

86 87 88 89 90

86 3S 0.44786

87 3PX 0.00000 0.16638

88 3PY 0.00000 0.00000 0.18425

89 3PZ 0.00000 0.00000 0.00000 0.18696

90 4S 0.24875 0.00000 0.00000 0.00000 0.30727

91 4PX 0.00000 0.07633 0.00000 0.00000 0.00000

92 4PY 0.00000 0.00000 0.09329 0.00000 0.00000

93 4PZ 0.00000 0.00000 0.00000 0.12259 0.00000

94 5D 0 0.00000 0.00000 0.00000 0.00000 0.00000

95 5D+1 0.00000 0.00000 0.00000 0.00000 0.00000

96 5D-1 0.00000 0.00000 0.00000 0.00000 0.00000

97 5D+2 0.00000 0.00000 0.00000 0.00000 0.00000

98 5D-2 0.00000 0.00000 0.00000 0.00000 0.00000

99 6 C 1S 0.00000 0.00000 0.00000 0.00000 0.00005

100 2S 0.00000 0.00000 0.00000 0.00000 0.00008

101 2PX -0.00001 0.00000 -0.00002 0.00000 -0.00033

102 2PY -0.00001 0.00000 -0.00002 0.00000 -0.00031

103 2PZ 0.00000 0.00000 0.00000 0.00000 -0.00001

104 3S -0.00017 -0.00006 -0.00031 0.00001 -0.00143

105 3PX -0.00026 -0.00001 -0.00032 0.00001 -0.00219

106 3PY -0.00031 -0.00003 -0.00032 0.00002 -0.00189

107 3PZ -0.00001 0.00001 -0.00001 -0.00009 -0.00003

108 4S -0.00218 -0.00030 -0.00217 -0.00013 -0.00198

109 4PX -0.00237 -0.00028 -0.00197 -0.00013 -0.00466

110 4PY -0.00272 0.00026 -0.00107 0.00006 -0.00183

111 4PZ -0.00005 0.00003 -0.00005 -0.00173 -0.00010

112 5D 0 0.00001 0.00001 0.00007 0.00001 -0.00001

113 5D+1 0.00000 0.00000 -0.00001 0.00002 0.00000

114 5D-1 0.00000 0.00000 0.00000 0.00000 0.00000

115 5D+2 0.00002 0.00000 0.00002 0.00000 0.00006

116 5D-2 0.00007 0.00006 0.00021 0.00001 0.00017

117 7 C 1S 0.00000 0.00000 0.00000 0.00000 0.00002

118 2S 0.00000 0.00000 0.00000 0.00000 0.00003

119 2PX 0.00000 0.00000 0.00000 0.00000 0.00002

120 2PY 0.00000 0.00000 0.00000 0.00000 -0.00007

121 2PZ 0.00000 0.00000 0.00000 0.00000 -0.00002

122 3S -0.00001 -0.00005 -0.00001 -0.00002 -0.00059

123 3PX -0.00002 -0.00007 -0.00008 -0.00014 0.00019

124 3PY -0.00002 -0.00006 0.00000 -0.00004 -0.00057

125 3PZ -0.00001 -0.00002 0.00001 0.00000 -0.00017

126 4S -0.00060 -0.00108 -0.00024 -0.00029 -0.00394

127 4PX -0.00050 -0.00079 -0.00120 -0.00208 0.00067

128 4PY -0.00044 -0.00090 0.00001 -0.00077 -0.00198

129 4PZ -0.00006 -0.00009 0.00025 -0.00063 -0.00093

130 5D 0 0.00000 0.00000 0.00000 0.00000 0.00002

131 5D+1 0.00000 0.00000 0.00000 0.00000 -0.00002

132 5D-1 0.00000 0.00000 0.00000 0.00000 -0.00002

133 5D+2 0.00000 0.00000 0.00000 0.00000 -0.00004

134 5D-2 0.00000 0.00001 0.00000 0.00001 -0.00002

135 8 C 1S 0.00000 0.00000 0.00000 0.00000 0.00002

136 2S 0.00000 0.00000 0.00000 0.00000 0.00004

137 2PX -0.00002 -0.00002 -0.00001 0.00000 -0.00063

138 2PY 0.00000 0.00000 0.00000 0.00000 -0.00010

139 2PZ 0.00000 0.00000 0.00000 0.00000 0.00003

140 3S -0.00015 -0.00032 -0.00004 -0.00001 -0.00101

141 3PX -0.00046 -0.00039 -0.00014 -0.00006 -0.00406

142 3PY 0.00000 0.00004 -0.00001 0.00000 -0.00073

143 3PZ -0.00001 -0.00006 0.00000 -0.00007 0.00018

144 4S -0.00110 -0.00397 -0.00021 0.00023 -0.00012

145 4PX -0.00364 -0.00353 -0.00077 -0.00043 -0.00837

146 4PY -0.00034 -0.00006 0.00044 -0.00004 -0.00304

147 4PZ 0.00010 -0.00016 0.00006 -0.00185 0.00129

148 5D 0 0.00000 0.00001 0.00000 0.00000 -0.00004

149 5D+1 0.00001 0.00003 0.00000 0.00003 0.00005

150 5D-1 0.00000 0.00000 0.00000 0.00000 0.00000

151 5D+2 0.00005 0.00016 0.00003 0.00000 0.00009

152 5D-2 0.00003 0.00006 -0.00002 0.00000 0.00010

153 9 C 1S -0.00036 -0.00087 -0.00074 0.00000 0.00036

154 2S -0.00059 -0.00151 -0.00128 0.00000 0.00046

155 2PX 0.00400 0.00360 0.00634 0.00002 0.00166

156 2PY 0.00306 0.00580 0.00226 -0.00002 0.00208

157 2PZ 0.00001 0.00002 0.00000 0.00233 0.00002

158 3S 0.01517 0.02171 0.01846 -0.00004 -0.00349

159 3PX 0.02349 0.00974 0.02375 0.00007 0.00884

160 3PY 0.01798 0.02108 0.00510 -0.00008 0.01065

161 3PZ 0.00006 0.00010 -0.00002 0.01435 0.00011

162 4S 0.00387 0.00939 0.00449 -0.00003 -0.03550

163 4PX 0.01229 -0.00047 0.00441 0.00007 -0.00227

164 4PY 0.01118 0.00908 -0.00366 -0.00004 0.00648

165 4PZ 0.00011 0.00006 0.00000 0.01538 0.00026

166 5D 0 0.00079 0.00023 0.00018 0.00000 0.00016

167 5D+1 0.00000 0.00000 -0.00001 0.00216 0.00000

168 5D-1 0.00001 0.00000 0.00000 0.00134 0.00000

169 5D+2 0.00004 0.00097 0.00227 0.00000 0.00008

170 5D-2 0.00380 0.00150 0.00087 0.00000 0.00122

171 10 H 1S 0.00000 0.00000 0.00000 0.00000 0.00005

172 2S 0.00001 0.00003 -0.00001 0.00000 0.00036

173 3S 0.00009 0.00067 0.00002 0.00001 0.00058

174 4PX 0.00000 0.00000 0.00000 0.00000 0.00000

175 4PY 0.00000 0.00000 0.00000 0.00000 0.00000

176 4PZ 0.00000 0.00000 0.00000 0.00000 0.00000

177 11 H 1S 0.00000 0.00000 0.00000 0.00000 0.00002

178 2S 0.00000 0.00000 0.00001 0.00000 0.00024

179 3S 0.00030 0.00006 0.00062 0.00001 0.00146

180 4PX 0.00000 0.00000 0.00000 0.00000 0.00000

181 4PY 0.00000 0.00000 0.00000 0.00000 0.00000

182 4PZ 0.00000 0.00000 0.00000 0.00000 0.00000

183 12 H 1S 0.00000 0.00000 0.00000 0.00000 0.00000

184 2S 0.00000 0.00000 0.00000 0.00000 0.00002

185 3S 0.00001 0.00008 0.00001 0.00010 0.00003

186 4PX 0.00000 0.00000 0.00000 0.00000 0.00000

187 4PY 0.00000 0.00000 0.00000 0.00000 0.00000

188 4PZ 0.00000 0.00000 0.00000 0.00000 0.00000

189 13 H 1S 0.00000 0.00000 0.00000 0.00000 0.00004

190 2S 0.00001 0.00001 0.00000 0.00000 0.00042

191 3S 0.00025 0.00037 0.00007 -0.00004 0.00141

192 4PX 0.00000 0.00000 0.00000 0.00000 0.00000

193 4PY 0.00000 0.00000 0.00000 0.00000 0.00000

194 4PZ 0.00000 0.00000 0.00000 0.00000 0.00000

195 14 H 1S -0.00004 -0.00004 -0.00013 0.00000 -0.00146

196 2S -0.00088 -0.00050 -0.00182 -0.00001 -0.00664

197 3S -0.00268 -0.00043 -0.00410 -0.00013 -0.00797

198 4PX 0.00002 0.00001 0.00007 0.00000 0.00004

199 4PY 0.00002 0.00000 0.00014 0.00001 -0.00004

200 4PZ 0.00000 0.00000 0.00000 0.00002 -0.00002

91 92 93 94 95

91 4PX 0.10876

92 4PY 0.00000 0.13212

93 4PZ 0.00000 0.00000 0.19760

94 5D 0 0.00000 0.00000 0.00000 0.00073

95 5D+1 0.00000 0.00000 0.00000 0.00000 0.00117

96 5D-1 0.00000 0.00000 0.00000 0.00000 0.00000

97 5D+2 0.00000 0.00000 0.00000 0.00000 0.00000

98 5D-2 0.00000 0.00000 0.00000 0.00000 0.00000

99 6 C 1S 0.00011 0.00005 0.00000 0.00000 0.00000

100 2S 0.00016 0.00012 0.00000 0.00000 0.00000

101 2PX -0.00010 -0.00025 -0.00001 0.00000 0.00000

102 2PY 0.00018 -0.00070 0.00002 0.00000 0.00000

103 2PZ 0.00000 -0.00002 -0.00022 0.00000 0.00000

104 3S -0.00182 -0.00281 -0.00001 0.00000 0.00000

105 3PX -0.00046 -0.00114 -0.00001 0.00002 0.00000

106 3PY 0.00098 -0.00363 0.00012 0.00001 0.00000

107 3PZ 0.00003 -0.00009 -0.00149 0.00000 0.00001

108 4S -0.00084 -0.00507 -0.00056 -0.00006 0.00000

109 4PX -0.00020 -0.00133 -0.00042 0.00000 0.00000

110 4PY 0.00457 -0.00431 0.00020 -0.00002 0.00000

111 4PZ 0.00007 -0.00026 -0.00816 0.00000 0.00003

112 5D 0 0.00001 0.00015 0.00004 0.00000 0.00000

113 5D+1 0.00000 -0.00004 0.00016 0.00000 0.00000

114 5D-1 0.00001 0.00000 -0.00007 0.00000 0.00000

115 5D+2 0.00004 -0.00009 -0.00002 0.00000 0.00000

116 5D-2 0.00010 0.00039 0.00001 0.00000 0.00000

117 7 C 1S 0.00007 0.00000 0.00001 0.00000 0.00000

118 2S 0.00010 0.00000 0.00002 0.00000 0.00000

119 2PX -0.00007 -0.00017 -0.00037 0.00000 0.00000

120 2PY -0.00015 0.00002 -0.00011 0.00000 0.00000

121 2PZ -0.00001 0.00001 -0.00006 0.00000 0.00000

122 3S -0.00149 -0.00022 -0.00057 0.00000 0.00000

123 3PX -0.00044 -0.00107 -0.00239 0.00000 0.00001

124 3PY -0.00105 0.00004 -0.00077 0.00000 0.00000

125 3PZ -0.00008 0.00008 -0.00054 0.00000 0.00000

126 4S -0.00612 -0.00026 -0.00150 0.00000 0.00000

127 4PX -0.00211 -0.00322 -0.00836 -0.00002 0.00007

128 4PY -0.00333 -0.00052 -0.00305 0.00000 0.00001

129 4PZ -0.00017 0.00063 -0.00488 0.00004 0.00001

130 5D 0 0.00000 0.00001 -0.00005 0.00000 0.00000

131 5D+1 0.00002 -0.00002 -0.00003 0.00000 0.00000

132 5D-1 -0.00003 0.00000 0.00001 0.00000 0.00000

133 5D+2 0.00002 -0.00006 0.00003 0.00000 0.00000

134 5D-2 0.00003 0.00000 0.00007 0.00000 0.00000

135 8 C 1S 0.00016 0.00002 0.00000 0.00000 0.00000

136 2S 0.00029 0.00004 0.00000 0.00000 0.00000

137 2PX -0.00103 -0.00022 -0.00013 0.00000 0.00000

138 2PY -0.00006 0.00012 -0.00001 0.00000 0.00000

139 2PZ -0.00007 0.00001 -0.00031 0.00000 0.00000

140 3S -0.00495 -0.00056 -0.00006 0.00000 0.00000

141 3PX -0.00517 -0.00122 -0.00075 0.00003 0.00005

142 3PY -0.00043 0.00085 -0.00004 0.00001 0.00001

143 3PZ -0.00038 0.00005 -0.00214 -0.00001 0.00005

144 4S -0.01295 -0.00146 0.00097 0.00000 0.00002

145 4PX -0.00803 -0.00264 -0.00197 0.00003 0.00014

146 4PY -0.00112 0.00498 -0.00028 0.00002 0.00003

147 4PZ -0.00035 0.00027 -0.01175 -0.00003 0.00034

148 5D 0 -0.00001 -0.00001 0.00000 0.00000 0.00000

149 5D+1 0.00008 0.00001 0.00018 0.00000 0.00001

150 5D-1 0.00000 0.00000 -0.00002 0.00000 0.00000

151 5D+2 0.00017 0.00009 -0.00001 0.00000 0.00000

152 5D-2 0.00016 -0.00020 0.00001 0.00000 0.00000

153 9 C 1S -0.00015 -0.00094 0.00001 -0.00006 0.00000

154 2S -0.00068 -0.00184 0.00001 -0.00009 0.00000

155 2PX 0.00021 0.00336 0.00001 0.00013 0.00000

156 2PY 0.00223 -0.00041 -0.00001 0.00008 0.00000

157 2PZ 0.00002 0.00001 0.00336 0.00000 0.00036

158 3S 0.01274 0.02171 -0.00012 0.00067 0.00000

159 3PX -0.00105 0.01406 0.00005 0.00024 0.00000

160 3PY 0.00885 -0.00370 -0.00006 0.00016 0.00000

161 3PZ 0.00010 0.00004 0.01816 0.00000 0.00155

162 4S -0.00200 0.00245 -0.00012 0.00020 0.00000

163 4PX -0.00182 0.00135 0.00008 -0.00002 0.00000

164 4PY 0.00488 -0.01069 -0.00004 -0.00001 0.00000

165 4PZ 0.00011 0.00006 0.01986 0.00000 0.00119

166 5D 0 -0.00001 -0.00002 0.00000 0.00002 0.00000

167 5D+1 0.00000 0.00000 0.00184 0.00000 -0.00009

168 5D-1 0.00000 0.00000 0.00085 0.00000 0.00007

169 5D+2 0.00077 0.00122 0.00000 0.00000 0.00000

170 5D-2 -0.00003 -0.00025 0.00000 0.00001 0.00000

171 10 H 1S 0.00017 -0.00015 0.00000 0.00000 0.00000

172 2S 0.00156 -0.00110 0.00003 0.00000 0.00000

173 3S 0.00401 -0.00133 0.00008 0.00000 0.00000

174 4PX 0.00000 -0.00006 0.00000 0.00000 0.00000

175 4PY -0.00002 -0.00001 0.00000 0.00000 0.00000

176 4PZ 0.00000 0.00000 -0.00001 0.00000 0.00000

177 11 H 1S -0.00002 0.00015 0.00000 0.00000 0.00000

178 2S -0.00007 0.00122 0.00001 0.00000 0.00000

179 3S 0.00068 0.00438 0.00005 0.00000 0.00000

180 4PX 0.00000 0.00000 0.00000 0.00000 0.00000

181 4PY -0.00001 0.00000 0.00000 0.00000 0.00000

182 4PZ 0.00000 0.00000 0.00000 0.00000 0.00000

183 12 H 1S 0.00000 0.00000 0.00001 0.00000 0.00000

184 2S 0.00006 0.00002 0.00015 0.00000 0.00000

185 3S 0.00039 0.00003 0.00082 0.00000 0.00000

186 4PX 0.00000 0.00000 -0.00002 0.00000 0.00000

187 4PY 0.00000 0.00000 0.00000 0.00000 0.00000

188 4PZ 0.00000 0.00000 0.00000 0.00000 0.00000

189 13 H 1S 0.00011 0.00003 -0.00001 0.00000 0.00000

190 2S 0.00107 0.00022 -0.00011 0.00000 0.00000

191 3S 0.00257 0.00055 -0.00017 0.00000 -0.00001

192 4PX 0.00001 0.00000 -0.00002 0.00000 0.00000

193 4PY 0.00000 0.00000 0.00000 0.00000 0.00000

194 4PZ 0.00000 0.00000 0.00000 0.00000 0.00000

195 14 H 1S -0.00035 -0.00165 0.00000 0.00000 0.00000

196 2S -0.00108 -0.00626 0.00005 0.00002 0.00000

197 3S -0.00004 -0.01061 -0.00029 0.00000 0.00000

198 4PX -0.00004 0.00021 0.00000 0.00000 0.00000

199 4PY -0.00003 0.00043 0.00002 0.00000 0.00000

200 4PZ -0.00002 -0.00001 0.00019 0.00000 0.00000

96 97 98 99 100

96 5D-1 0.00123

97 5D+2 0.00000 0.00278

98 5D-2 0.00000 0.00000 0.00187

99 6 C 1S 0.00000 0.00000 0.00000 0.65850

100 2S 0.00000 0.00000 0.00000 0.49200 0.48912

101 2PX 0.00000 0.00000 0.00000 0.00000 0.00000

102 2PY 0.00000 0.00000 0.00001 0.00000 0.00000

103 2PZ 0.00000 0.00000 0.00000 0.00000 0.00000

104 3S 0.00000 -0.00001 0.00007 -0.02737 -0.06047

105 3PX 0.00001 -0.00001 0.00011 0.00000 0.00000

106 3PY 0.00000 0.00000 0.00016 0.00000 0.00000

107 3PZ 0.00003 0.00000 0.00000 0.00000 0.00000

108 4S -0.00001 -0.00002 0.00014 -0.01076 -0.01901

109 4PX 0.00002 -0.00003 0.00008 0.00000 0.00000

110 4PY -0.00001 0.00002 0.00027 0.00000 0.00000

111 4PZ 0.00020 0.00000 0.00000 0.00000 0.00000

112 5D 0 0.00000 0.00000 0.00000 0.00000 0.00000

113 5D+1 0.00000 0.00000 0.00000 0.00000 0.00000

114 5D-1 0.00000 0.00000 0.00000 0.00000 0.00000

115 5D+2 0.00000 0.00000 0.00000 0.00000 0.00000

116 5D-2 0.00000 0.00000 0.00002 0.00000 0.00000

117 7 C 1S 0.00000 0.00000 0.00000 0.00000 0.00000

118 2S 0.00000 0.00000 0.00000 0.00000 0.00000

119 2PX 0.00000 0.00000 0.00000 -0.00002 -0.00001

120 2PY 0.00000 0.00000 0.00000 -0.00001 -0.00001

121 2PZ 0.00000 0.00000 0.00000 -0.00001 -0.00001

122 3S 0.00000 0.00000 0.00000 -0.00035 -0.00057

123 3PX 0.00000 0.00000 0.00000 -0.00079 -0.00137

124 3PY 0.00000 0.00000 0.00000 -0.00049 -0.00086

125 3PZ 0.00000 0.00000 0.00000 -0.00031 -0.00052

126 4S 0.00001 -0.00004 -0.00004 0.00017 0.00024

127 4PX -0.00001 0.00000 0.00000 -0.00045 -0.00097

128 4PY 0.00000 -0.00008 0.00000 -0.00032 -0.00068

129 4PZ -0.00001 -0.00001 -0.00002 -0.00012 -0.00023

130 5D 0 0.00000 0.00000 0.00000 -0.00002 -0.00002

131 5D+1 0.00000 0.00000 0.00000 -0.00007 -0.00011

132 5D-1 0.00000 0.00000 0.00000 -0.00008 -0.00012

133 5D+2 0.00000 0.00000 0.00000 0.00000 -0.00001

134 5D-2 0.00000 0.00000 0.00000 -0.00014 -0.00023

135 8 C 1S 0.00000 0.00000 0.00000 0.00000 0.00000

136 2S 0.00000 0.00000 0.00000 0.00000 0.00000

137 2PX 0.00000 0.00001 0.00000 0.00000 0.00000

138 2PY 0.00000 0.00000 0.00000 0.00000 0.00000

139 2PZ 0.00000 0.00000 0.00000 0.00000 0.00000

140 3S 0.00000 0.00010 0.00000 0.00000 0.00000

141 3PX 0.00001 0.00027 0.00001 0.00000 0.00000

142 3PY 0.00000 -0.00003 -0.00001 0.00000 0.00000

143 3PZ 0.00001 0.00002 0.00000 0.00000 0.00000

144 4S 0.00001 0.00020 -0.00004 0.00004 0.00006

145 4PX 0.00003 0.00032 -0.00003 0.00001 0.00001

146 4PY -0.00004 -0.00013 -0.00011 0.00013 0.00021

147 4PZ 0.00009 0.00005 -0.00001 0.00000 0.00000

148 5D 0 0.00000 0.00000 0.00000 0.00000 0.00000

149 5D+1 0.00000 0.00000 0.00000 0.00000 0.00000

150 5D-1 0.00000 0.00000 0.00000 0.00000 0.00000

151 5D+2 0.00000 0.00002 0.00001 0.00000 0.00000

152 5D-2 0.00000 0.00000 0.00000 0.00000 0.00000

153 9 C 1S 0.00000 -0.00001 -0.00023 0.00000 0.00000

154 2S 0.00000 -0.00001 -0.00037 0.00000 0.00000

155 2PX 0.00000 0.00019 0.00070 0.00000 0.00000

156 2PY 0.00000 0.00059 0.00035 0.00000 0.00000

157 2PZ 0.00031 0.00000 0.00000 0.00000 0.00000

158 3S 0.00000 0.00005 0.00298 0.00000 0.00000

159 3PX 0.00000 0.00098 0.00129 0.00000 0.00000

160 3PY 0.00000 0.00213 0.00055 0.00000 0.00000

161 3PZ 0.00134 0.00000 0.00000 0.00000 0.00000

162 4S 0.00000 -0.00004 0.00092 -0.00001 -0.00001

163 4PX 0.00000 0.00065 -0.00001 0.00000 0.00000

164 4PY 0.00000 0.00086 -0.00011 -0.00003 -0.00004

165 4PZ 0.00109 0.00000 0.00000 0.00000 0.00000

166 5D 0 0.00000 0.00000 0.00001 0.00000 0.00000

167 5D+1 -0.00014 0.00000 0.00000 0.00000 0.00000

168 5D-1 0.00002 0.00000 0.00000 0.00000 0.00000

169 5D+2 0.00000 -0.00069 -0.00002 0.00000 0.00000

170 5D-2 0.00000 0.00000 0.00004 0.00000 0.00000

171 10 H 1S 0.00000 0.00000 0.00000 0.00000 0.00000

172 2S 0.00000 0.00000 0.00000 0.00000 0.00000

173 3S 0.00000 -0.00001 -0.00003 0.00000 0.00000

174 4PX 0.00000 0.00000 0.00000 0.00000 0.00000

175 4PY 0.00000 0.00000 0.00000 0.00000 0.00000

176 4PZ 0.00000 0.00000 0.00000 0.00000 0.00000

177 11 H 1S 0.00000 0.00000 0.00000 -0.00022 -0.00031

178 2S 0.00000 0.00000 0.00000 -0.00227 -0.00392

179 3S 0.00000 0.00001 -0.00001 -0.00005 -0.00016

180 4PX 0.00000 0.00000 0.00000 -0.00002 -0.00002

181 4PY 0.00000 0.00000 0.00000 -0.00052 -0.00079

182 4PZ 0.00000 0.00000 0.00000 -0.00002 -0.00003

183 12 H 1S 0.00000 0.00000 0.00000 0.00000 0.00000

184 2S 0.00000 0.00000 0.00000 0.00001 0.00001

185 3S 0.00000 0.00000 0.00000 0.00008 0.00013

186 4PX 0.00000 0.00000 0.00000 0.00000 0.00000

187 4PY 0.00000 0.00000 0.00000 0.00000 0.00000

188 4PZ 0.00000 0.00000 0.00000 0.00000 0.00000

189 13 H 1S 0.00000 0.00000 0.00000 0.00000 0.00000

190 2S 0.00000 0.00000 0.00000 0.00000 0.00000

191 3S 0.00000 -0.00001 0.00000 0.00000 0.00000

192 4PX 0.00000 0.00000 0.00000 0.00000 0.00000

193 4PY 0.00000 0.00000 0.00000 0.00000 0.00000

194 4PZ 0.00000 0.00000 0.00000 0.00000 0.00000

195 14 H 1S 0.00000 0.00003 0.00000 0.00000 0.00000

196 2S 0.00000 0.00040 0.00004 0.00000 0.00000

197 3S -0.00001 0.00022 -0.00002 0.00000 0.00000

198 4PX 0.00000 0.00000 0.00000 0.00000 0.00000

199 4PY 0.00000 0.00001 0.00001 0.00000 0.00000

200 4PZ 0.00000 0.00000 0.00000 0.00000 0.00000

101 102 103 104 105

101 2PX 0.08447

102 2PY 0.00000 0.08757

103 2PZ 0.00000 0.00000 0.07152

104 3S 0.00000 0.00000 0.00000 0.43582

105 3PX 0.07636 0.00000 0.00000 0.00000 0.17469

106 3PY 0.00000 0.07814 0.00000 0.00000 0.00000

107 3PZ 0.00000 0.00000 0.06743 0.00000 0.00000

108 4S 0.00000 0.00000 0.00000 0.19504 0.00000

109 4PX 0.01498 0.00000 0.00000 0.00000 0.06379

110 4PY 0.00000 0.01668 0.00000 0.00000 0.00000

111 4PZ 0.00000 0.00000 0.01909 0.00000 0.00000

112 5D 0 0.00000 0.00000 0.00000 0.00000 0.00000

113 5D+1 0.00000 0.00000 0.00000 0.00000 0.00000

114 5D-1 0.00000 0.00000 0.00000 0.00000 0.00000

115 5D+2 0.00000 0.00000 0.00000 0.00000 0.00000

116 5D-2 0.00000 0.00000 0.00000 0.00000 0.00000

117 7 C 1S -0.00002 -0.00001 -0.00001 -0.00035 -0.00080

118 2S -0.00001 -0.00001 -0.00001 -0.00057 -0.00137

119 2PX 0.00015 0.00031 0.00068 0.00347 0.00092

120 2PY 0.00057 0.00014 0.00003 0.00220 0.00495

121 2PZ 0.00051 0.00023 -0.00010 0.00132 0.00460

122 3S 0.00342 0.00241 0.00160 0.01463 0.01960

123 3PX 0.00074 0.00269 0.00603 0.01958 0.00170

124 3PY 0.00509 0.00063 0.00026 0.01284 0.01873

125 3PZ 0.00447 0.00192 -0.00026 0.00707 0.01698

126 4S 0.00123 0.00119 0.00125 -0.00315 0.00625

127 4PX 0.00004 0.00053 0.00372 0.01231 0.00058

128 4PY 0.00261 -0.00096 -0.00063 0.00903 0.01074

129 4PZ 0.00210 0.00101 0.00166 0.00244 0.00886

130 5D 0 0.00000 0.00000 0.00042 0.00019 0.00003

131 5D+1 0.00015 0.00051 -0.00008 0.00085 0.00027

132 5D-1 0.00059 0.00006 -0.00002 0.00100 0.00147

133 5D+2 0.00010 0.00043 0.00003 0.00008 0.00062

134 5D-2 0.00024 0.00003 0.00059 0.00183 0.00040

135 8 C 1S 0.00000 0.00000 0.00000 0.00000 0.00000

136 2S 0.00000 0.00000 0.00000 0.00000 0.00000

137 2PX 0.00000 0.00000 0.00000 0.00000 0.00000

138 2PY 0.00000 0.00000 0.00000 0.00000 0.00000

139 2PZ 0.00000 0.00000 0.00000 0.00000 0.00000

140 3S 0.00000 0.00000 0.00000 -0.00004 -0.00003

141 3PX 0.00000 0.00000 0.00000 0.00000 0.00000

142 3PY 0.00000 -0.00001 0.00000 -0.00018 -0.00015

143 3PZ 0.00000 0.00000 0.00000 -0.00001 0.00001

144 4S -0.00007 -0.00030 0.00000 -0.00123 -0.00049

145 4PX 0.00000 0.00014 0.00001 0.00002 -0.00005

146 4PY -0.00025 -0.00066 0.00004 -0.00405 -0.00153

147 4PZ 0.00001 -0.00004 0.00000 -0.00003 0.00006

148 5D 0 0.00000 0.00000 0.00000 0.00000 0.00001

149 5D+1 0.00000 0.00000 0.00000 0.00000 0.00000

150 5D-1 0.00000 0.00000 0.00000 0.00000 0.00000

151 5D+2 0.00000 0.00000 0.00000 0.00000 0.00001

152 5D-2 0.00000 0.00000 0.00000 0.00001 0.00000

153 9 C 1S 0.00000 0.00000 0.00000 0.00000 0.00000

154 2S 0.00000 0.00000 0.00000 0.00000 0.00000

155 2PX 0.00000 0.00000 0.00000 0.00000 0.00000

156 2PY 0.00000 0.00000 0.00000 0.00000 0.00000

157 2PZ 0.00000 0.00000 0.00000 0.00000 0.00000

158 3S 0.00000 0.00000 0.00000 0.00000 0.00000

159 3PX 0.00000 0.00000 0.00000 0.00000 0.00000

160 3PY 0.00000 0.00000 0.00000 -0.00001 -0.00001

161 3PZ 0.00000 0.00000 0.00000 0.00000 0.00000

162 4S 0.00000 -0.00013 0.00000 0.00010 0.00002

163 4PX 0.00002 0.00003 0.00000 0.00003 0.00020

164 4PY -0.00001 -0.00013 0.00000 0.00039 -0.00008

165 4PZ 0.00000 0.00001 -0.00008 0.00002 -0.00002

166 5D 0 0.00000 0.00000 0.00000 0.00000 0.00000

167 5D+1 0.00000 0.00000 0.00000 0.00000 0.00000

168 5D-1 0.00000 0.00000 0.00000 0.00000 0.00000

169 5D+2 0.00000 0.00000 0.00000 -0.00001 0.00000
[truncated: 1,191,344 more chars]
